# Fluorspar to fluorochemicals upon low-temperature activation in water

**DOI:** 10.1038/s41586-024-08125-1

**Published:** 2024-11-13

**Authors:** Immo Klose, Calum Patel, Anirban Mondal, Andrew Schwarz, Gabriele Pupo, Véronique Gouverneur

**Affiliations:** 1 University of Oxford, Chemistry Research Laboratory, Oxford, UK; 2 FluoRok, Oxford, UK

**Keywords:** Synthetic chemistry methodology, Organic chemistry, Inorganic chemistry, Materials chemistry

## Abstract

The dangerous chemical hydrogen fluoride sits at the apex of the fluorochemical industry, but the substantial hazards linked to its production under harsh conditions (above 300 degrees Celsius) and transport are typically contracted to specialists. All fluorochemicals for applications, including refrigeration, electric transportation, agrochemicals and pharmaceuticals, are prepared from fluorspar (CaF_2_) through a procedure that generates highly dangerous hydrogen fluoride^1–5^. Here we report a mild method to obtain fluorochemicals directly from fluorspar, bypassing the necessity to manufacture hydrogen fluoride. Acid-grade fluorspar (more than 97 per cent CaF_2_) is treated with the fluorophilic Lewis acid boric acid (B(OH)_3_) or silicon dioxide (SiO_2_), in the presence of oxalic acid, a Brønsted acid that is highly effective for Ca^2+^ sequestration. This scalable process carried out in water at low temperature (below 50 degrees Celsius) enables access to widely used fluorochemicals, including tetrafluoroboric acid, alkali metal fluorides, tetraalkylammonium fluorides and fluoro(hetero)arenes. The replacement of oxalic acid with sulfuric acid gave comparable results for B(OH)_3_, but was not as effective when the fluorophilic Lewis acid was SiO_2_. A similar process also works with the lower-purity metspar. The production of fluorochemicals directly from fluorspar offers the possibility of decentralized manufacturing—an attractive model for the fluorochemical industry. With the renewed interest in innovative methods to synthesize oxalic acid via carbon dioxide capture and biomass^6,7^, and the challenges posed by our dependence on fossil fuels for sulfur and therefore sulfuric acid supply^8,9^, our technology may represent a departure towards a sustainable fluorochemical industry.

## Abstract (web summary)

Fluorochemicals are obtained directly from fluorspar activated in water at low temperature, without the requirement to manufacture hydrogen fluoride, a toxic and hazardous gas that is central to the current industrial process.

## Main

**Fig. 1:**
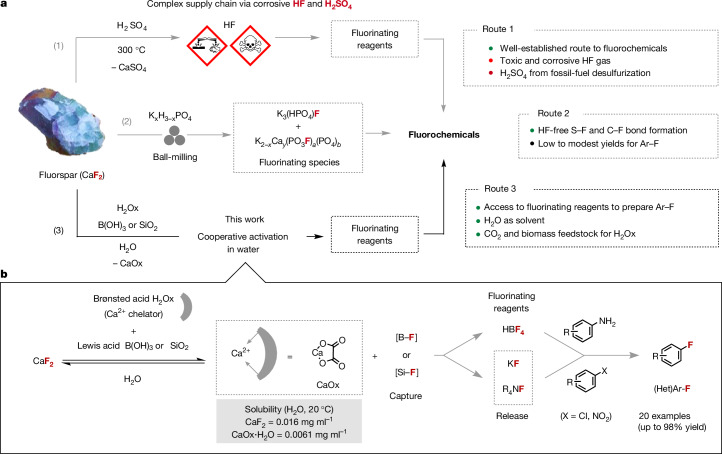
Strategies to access fluorochemicals from fluorspar. **a**, Overview of routes to fluorochemicals: (1) current industrial route, (2) preparation of fluorochemicals using phosphate-activated fluorspar, (3) synthesis of fluorinating reagents from fluorspar activated in water with oxalic acid, and B(OH)_3_ or SiO_2_. (Het)Ar, heteroaromatic. **b**, Cooperative activation of fluorspar using both a Brønsted acid and a fluorophilic Lewis acid (B(OH)_3_ or SiO_2_) to prepare fluorinating reagents for the synthesis of fluoroarenes (this work).

Innovations with an impact on the large-scale production of chemicals are in demand considering the pressing global challenges faced by the manufacturing industry. These include the availability and management of raw materials, transportation disruptions, complex supply chains and climate change pressure^10^. This urgency applies to fluorochemicals, a class of molecules that remain in increasing demand owing to their critical role as pharmaceuticals to cure diseases, agrochemicals for food security and in the production of lithium-ion batteries, which is expected to soar over the coming decade^1–3,11^. At present, the starting point of the entire fluorine industry is the treatment of fluorspar (fluorite, CaF_2_) with concentrated sulfuric acid (H_2_SO_4_) at high temperature (>300 °C) to produce dangerous hydrogen fluoride (HF)^4,5^. Subsequently, HF is used as is, or converted into various fluorinating reagents for the synthesis of fluorochemicals (Fig. 1a). For decades, other routes to HF have been studied, all featuring inorganic acids, such as hydrochloric acid (HCl), and requiring harsh reaction conditions^12^. We recently disclosed an activation strategy whereby fluorochemicals as diverse as sulfonyl, benzyl, allyl and alkyl fluorides can be accessed with a fluorinating reagent directly prepared from acid-grade fluorspar (AGF; >97% CaF_2_)^13^. This solid reagent obtained by ball-milling CaF_2_ with dipotassium phosphate (K_2_HPO_4_) consists of two crystalline components identified as K_3_(HPO_4_)F and K_2−*x*_Ca_*y*_(PO_3_F)_*a*_(PO_4_)_*b*_. This mechanochemical reaction stands out because it bypasses the production, storage and complex transport chain of HF. The reactivity of this fluorinating reagent for well-established transformations unavoidably requires independent investigation, and preliminary work has revealed some limitations. One of the challenges that we encountered is its broad application to the synthesis of fluoroarenes frequently used for the production of pharmaceuticals and agrochemicals. Also, mechanochemical reactions require specialized equipment that is available in only a few laboratories. This state of play encouraged the development of an alternative strategy to prepare fluorochemicals directly from fluorspar applying mild conditions for its activation. Departing from solid-state chemistry induced by mechanical energy, we surmise that activation of fluorspar in solution, and specifically in nature’s solvent water, would represent a highly attractive approach (Fig. 1b).

**Fig. 2:**
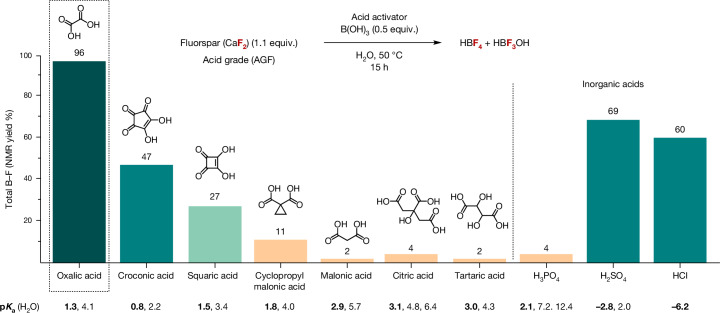
Identification of oxalic acid for cooperative activation of fluorspar. Screening of Brønsted acids for cooperative activation of AGF with B(OH)_3_ (2.0 mmol) at 50 °C in water. The yields of HBF_4_ and HBF_3_OH were determined by ^19^F NMR spectroscopy (details in Supplementary Tables 3–6). The first p*K*_a_ value (bold) refers to the dissociation of the first proton of the acid, and subsequent p*K*_a_ values (non-bold) refer to the dissociation of successive protons. p*K*_a_, negative logarithm of the acid dissociation constant.

**Fig. 3:**
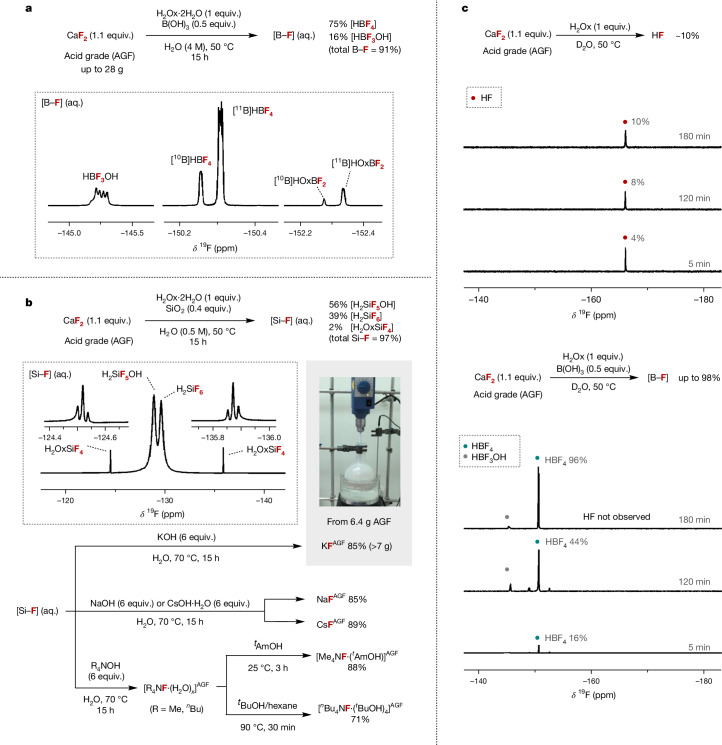
Fluorinating reagents from fluorspar and mechanistic insight. **a**, Preparation of HBF_4_ from AGF, B(OH)_3_ and H_2_Ox·2H_2_O with ^19^F NMR (D_2_O) spectra of reaction mixture containing [B–F] products HBF_4_, HBF_3_OH and HOxBF_2_. **b**, Preparation of KF, NaF, CsF, Me_4_NF and ^*n*^Bu_4_NF from AGF, SiO_2_ and H_2_Ox·2H_2_O with ^19^F NMR (D_2_O) spectra of reaction mixture containing [Si–F] products H_2_SiF_6_, H_2_SiF_5_OH and H_2_OxSiF_4_. The yields of H_2_SiF_6_, H_2_SiF_5_OH and H_2_OxSiF_4_ were determined by ^19^F NMR spectroscopy (further details provided in Supplementary Fig. 18). **c**, Monitoring the reaction of AGF (0.5 mmol) with anhydrous H_2_Ox with and without B(OH)_3_ by ^19^F NMR spectroscopy in D_2_O (HF, HBF_4_ and HBF_3_OH yield quantified by ^19^F NMR spectroscopy; details in Supplementary Figs. 6 and 7).

In search for a suitable manifold to activate CaF_2_ in water, we considered mild acidic conditions that provide direct access to frequently used fluorinating reagents other than HF. We propose that the synergistic use of a Brønsted acid and a fluorophilic Lewis acid would activate CaF_2_, with Ca^2+^ capture as an insoluble salt and simultaneous HF capture in a form suitable for subsequent fluorination processes. A report by Lennox and Lloyd-Jones provided guidance by highlighting the beneficial influence of tartaric acid on the equilibrium in solution between aryl boronic acids, potassium fluoride and potassium trifluoroborate salts through precipitation of potassium bitartrate^14^. This knowledge prompted us to select a suitable Brønsted acid for AGF activation that enables concurrent Ca^2+^ sequestration as an insoluble salt. Oxalic acid (C_2_H_2_O_4_, H_2_Ox) stood out as a suitable candidate because calcium oxalate (CaOx) is poorly soluble in water (0.0061 mg ml^−1^ (H_2_O, 20 °C))^15,16^. This choice was also driven by the appearance of economically viable and environmentally friendly methods for its synthesis, such as bio-based production in fast-growing microorganisms, or through carbon dioxide (CO_2_) capture^6,7^. The upsurge of interest in oxalic acid results from the ability of this solid organic acid to extract rare-earth elements from primary or waste ores and possibly replace existing recovery processes that depend on inorganic acids such as H_2_SO_4_ (ref. ^17^). Mechanistically, we surmise that the dissolution of AGF in water with oxalic acid in the presence of fluorophilic boric acid (B(OH)_3_) may be driven by precipitation of CaOx and immediate capture of HF as a strong B−F bond (732 kJ mol^−1^) with water as a by-product^15^. We prioritized B(OH)_3_ as the Lewis acid prompted by the kinetic studies of Wamser on the reaction of hydrofluoric acid and B(OH)_3_ in water^18^, and the target Balz–Schiemann reaction for the synthesis of fluoroarenes. Fluoride capture from AGF with B(OH)_3_ was found to be highly effective in the presence of H_2_Ox when the reaction was carried out in water for 15 h at 50 °C (Fig. 2). The [B−F] products were identified by ^19^F and ^11^B nuclear magnetic resonance (NMR) spectroscopy in deuterium oxide (D_2_O; Fig. 3a). By ^19^F NMR spectroscopy, HBF_4_ (quartet, chemical shift (*δ*) = −150.3 parts per million (ppm), coupling constant ^1^*J*_B–F_ = 1.1 Hz) and HBF_3_OH (quartet, *δ* = −145.3 ppm, ^1^*J*_B–F_ = 10.7 Hz) were identified as the major products^19^. In addition, a broad singlet (*δ* = −152.3 ppm) characteristic of difluoro(oxalate)borate species HOxBF_2_ was observed in trace amount (<1%)^20^. Notably, H_2_Ox afforded a higher yield (96%) of [B−F] products than tartaric acid (2%), H_2_SO_4_ (69%) or HCl (60%) under these reaction conditions (Fig. 2). An investigation on fluorspar activation with a range of Brønsted acids gave insight on the interplay between acidity, denticity and solubility of the Ca^2+^ salt by-product. For monoacids (2 equiv. versus CaF_2_), we noted a correlation between acidity and fluoride release in a p*K*_a_ (negative logarithm of the acid dissociation constant *K*_a_) range between 5 and –0.5 (Supplementary Table 3). Multifunctional activators (1 equiv. versus CaF_2_) were also investigated (Supplementary Table 4). Organic acids leading to five-membered Ca^2+^ chelates stood out with H_2_Ox (p*K*_a1_ = 1.3 and p*K*_a2_ = 4.1 (where p*K*_a1_ and p*K*_a2_ are the p*K*_a_ values associated with the dissociation of the first and second proton of the diacid, respectively)) being the most effective activator followed by croconic acid (p*K*_a1_ = 0.8 and p*K*_a2_ = 2.2) and squaric acid (p*K*_a1_ = 1.5 and p*K*_a2_ = 3.4). Oxalic acid dihydrate (H_2_Ox·2H_2_O), which is more cost-effective than H_2_Ox, gave the [B–F] products HBF_4_ and HBF_3_OH with efficacy similar to H_2_Ox (total yield of 98%) upon treatment of AGF with B(OH)_3_ at 50 °C for 15 h (Supplementary Table 12). Subsequent studies were therefore performed with H_2_Ox·2H_2_O.

It was also found that H_2_Ox·2H_2_O was a suitable activator for AGF when combined with silicon dioxide (SiO_2_) in water at 50 °C for 15 h, with fluoride release as [Si–F] products (Si–F bond 577 kJ mol^−1^) in 97% total yield^15^ (Fig. 3b). The formation of H_2_SiF_6_ (broad singlet, *δ* = −129.6 ppm), which in water exists in equilibrium with H_2_SiF_5_(OH) (broad singlet, *δ* = −128.8 ppm), was evidenced by ^19^F NMR spectroscopy of the reaction mixture^21^. Two triplets were also observed (*δ* = −124.5 ppm and −135.9 ppm, ^2^*J*_F–F_ = 8.9 Hz) and assigned to H_2_OxSiF_4_ (supported by ^29^Si NMR spectroscopy; Supplementary Figs. 14 and 15)^22^. Subsequent studies focused on the synthesis of frequently used fluorinating reagents from these [Si–F] products (Fig. 3b). For this purpose, AGF (1.1 equiv.) was reacted with H_2_Ox·2H_2_O (1 equiv.) and SiO_2_ (0.4 equiv.) in water at 50 °C for 15 h. The reaction mixture was filtered and treated with potassium hydroxide (KOH). The insoluble by-product of this filtration was unambiguously characterized as CaOx·H_2_O by powder X-ray diffraction (Supplementary Fig. 17). Neutralization with KOH (2 equiv.) led to the formation of K_2_SiF_6_ (Supplementary Fig. 20), whereas treatment with excess KOH (6 equiv.) afforded KF^AGF^ (85% yield, calculated from AGF; purity analysis is included in Supplementary Information)^23^. A similar protocol gave NaF^AGF^ (85% yield, 94% purity) and CsF^AGF^ (89% yield, 96% purity) from NaOH and CsOH·H_2_O, respectively. Alternatively, treatment of the filtered reaction mixture with tetramethylammonium hydroxide (6 equiv.) afforded tetramethylammonium fluoride hydrate, which was converted to tetramethylammonium *tert*-amyl alcohol fluoride [Me_4_NF·(^*t*^AmOH)] ^AGF^ (88% yield), a fluorinating reagent well documented for nucleophilic aromatic fluorination (S_N_Ar)^24^. This strategy also enabled the preparation of tetrabutylammonium fluoride hydrate, which was converted to the bench-stable reagent tetrabutylammonium tetra(*tert*-butyl alcohol) fluoride [Bu_4_NF·(^*t*^BuOH)_4_]^AGF^ (71% yield calculated from AGF)^25^. Mechanistic investigations by ^19^F NMR spectroscopy in D_2_O established whether HF is formed upon dissolution of CaF_2_ with H_2_Ox (Supplementary Fig. 6). In the absence of a fluorophilic Lewis acid, HF is indeed observed (singlet, *δ* = −166.0 ppm), and an equilibrium is established with the amount of HF plateauing after 3 h at approximately 10% (ref. ^26^; Fig. 3c). In the presence of either B(OH)_3_ or SiO_2_, the equilibrium is displaced via the precipitation of highly insoluble CaOx and immediate HF capture by the Lewis acid. Under these conditions, the singlet diagnostic of HF was not detected by ^19^F NMR spectroscopy during the entire course of the reaction. As anticipated, the reaction of AGF with fluorophilic Lewis acid in the absence of H_2_Ox resulted in no fluoride release (Supplementary Information). These data highlight how Brønsted and Lewis acid cooperativity allows for fluorspar activation under mild conditions, prevents HF from building up, and enables access to HBF_4_ (aq.), KF, NaF, CsF, Me_4_NF·^*t*^AmOH and ^*n*^Bu_4_NF·(^*t*^BuOH)_4_, directly from fluorspar.

Further studies compared the reactivity of H_2_Ox·2H_2_O and H_2_SO_4_ with AGF. At 50 °C, the combination of AGF, concentrated H_2_SO_4_ and B(OH)_3_ increased the yield of [B−F] products to 97% when the reaction time was extended to 24 h (versus 69% yield after 15 h). This reaction was also effective at 25 °C, offering [B−F] products in 94% yield (average of 2 runs) after a reaction time of 48 h. For comparison, H_2_Ox·2H_2_O afforded [B−F] products in 98% yield under these conditions (25 °C, 48 h). When SiO_2_ served as the fluorophilic Lewis acid, activation of AGF with H_2_Ox·2H_2_O was effective at 25 °C, albeit requiring a prolonged reaction time of 72 h. After neutralization with KOH, KF was indeed isolated in 74% yield. Replacement of oxalic acid for H_2_SO_4_ did not give satisfactory results. At 50 °C for 24 h, the reaction of AGF, concentrated H_2_SO_4_ and SiO_2_ gave only partial fluoride release as H_2_SiF_6_ and H_2_SiF_5_OH with a total [Si−F] product yield of 48% (quantified by ^19^F NMR spectroscopy). At 25 °C for 72 h, the total [Si−F] product yield was reduced to 29%. Notably, the treatment of the [Si−F] solutions with KOH afforded a solid material containing both KF and K_2_SO_4_ (46% KF (50 °C) and 36% KF (25 °C)) as evidenced by powder X-ray diffraction analysis, two salts that are challenging to separate (KF, 1,020 mg ml^−1^; K_2_SO_4_ 120 mg ml^−1^ (H_2_O, 25 °C))^15^. The formation of K_2_SO_4_ is indicative of the incomplete reaction between AGF, H_2_SO_4_ and SiO_2_. The overall superior reactivity of H_2_Ox·2H_2_O compared with H_2_SO_4_ correlates with the solubility of the calcium by-product formed upon AGF activation (CaOx.H_2_O, 0.0061 mg ml^−1^ versus CaSO_4_.2H_2_O, 2.1 mg ml^−1^ (H_2_O, 20 °C))^15^.

**Fig. 4:**
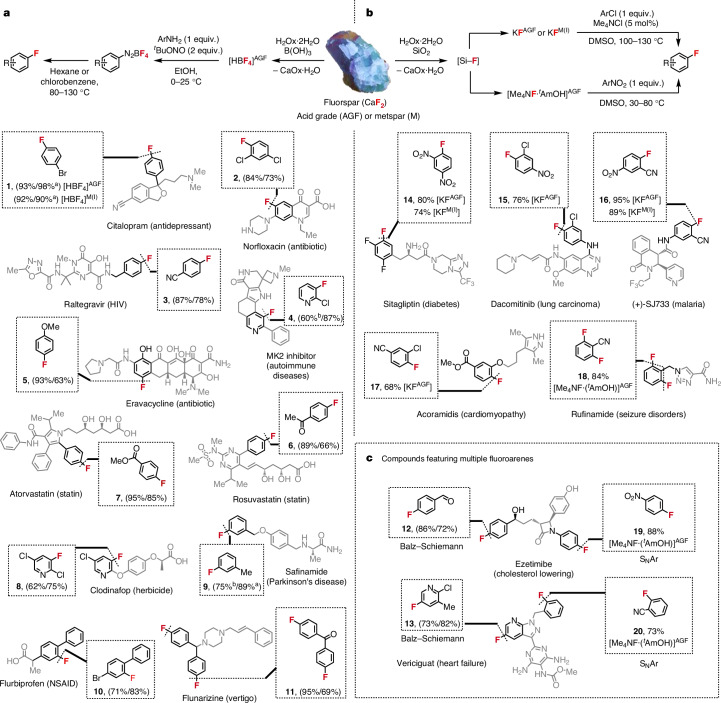
Scope of fluoroarenes prepared from fluorspar. **a**, Fluoroarenes prepared via Balz–Schiemann reaction using AGF-derived HBF_4_ [HBF_4_]^AGF^ or metspar-derived HBF_4_ [HBF_4_]^M(I)^ (yield of diazotization/yield of dediazoniation). Diazotization reactions were carried out with 5.0 mmol of aryl amine, excluding compounds **3**, **4**, **6**, **8**, **9** and **11**, which were carried out on 1.0 mmol of aryl amine based on differential scanning calorimetry analysis of the diazonium precursor (Supplementary Tables 29 and 30). Dediazoniation reactions were carried out on a 1.0-mmol scale (unless otherwise stated). All yields are for isolated products (unless otherwise stated). NSAID, nonsteroidal anti-inflammatory drug. **b**, Fluoroarenes prepared via halogen exchange (S_N_Ar) of chloroarenes using AGF-derived KF [KF]^AGF^ or metspar-derived KF [KF]^M(I)^, or fluorodenitration reactions of nitroarenes using AGF-derived Me_4_NF·^*t*^AmOH. Reactions were carried out on a 1.0-mmol scale (unless otherwise stated). All yields are for isolated products. **c**, Fluoroarenes prepared using AGF-derived HBF_4_ for Balz–Schiemann reaction and AGF-derived Me_4_NF·^*t*^AmOH for S_N_Ar reactions. M, metspar; MK, MAP-activated protein kinase. ^a19^F NMR yields using 4-fluoroanisole as internal standard. ^b^Diazonium salt precursors of **4** and **9** are prepared at 10 °C (maximum process temperature <25 °C). Alternatively, **4** and **9** can be prepared via a one-pot protocol to bypass the isolation of the diazonium salt intermediate (Supplementary Information).

With an effective strategy to convert AGF into HBF_4_ (aq.), KF and Me_4_NF·^*t*^AmOH, we had a duty to demonstrate that these AGF-derived reagents react as expected, with the synthesis of industrially valuable fluoroarenes and a focus on those not accessible via mechanochemical activation of AGF using a phosphate salt. For Balz–Schiemann chemistry, a two-step procedure followed the preparation of HBF_4_ (aq.); addition of *tert*-butyl nitrite to a solution of aryl amine and aqueous HBF_4_ led to the precipitation of the corresponding aryl diazonium tetrafluoroborate salt, which was isolated and subsequently heated to liberate the desired fluoroarene^27^ (Fig. 4a). The protocol was validated first with 4-bromoaniline, AGF-derived HBF_4_ (HBF_4_^AGF^; 1.1 equiv.) and *tert*-butyl nitrite (2 equiv.). Heating the resulting diazonium salt at 90 °C in chlorobenzene gave 4-bromofluorobenzene in 98% yield (as measured by ^19^F NMR spectroscopy), a key intermediate featured in the synthesis of the antidepressant citalopram^28^. This chemistry was subsequently applied to prepare multiple fluoroarenes frequently used as building blocks in the synthesis of various organo-fluorine-containing drugs in up to 87% yield (dediazoniation yield). Examples include the precursors of lipitor (cholesterol lowering), norfloxacin (antibiotic), raltegravir (HIV), eravacycline (antibiotic), rosuvastatin (cardiovascular disease), flurbiprofen (anti-inflammatory), flunarizine (vertigo) and ezetimibe (cholesterol-lowering drug)^29–36^. The methodology was also suitable for the preparation of fluoropyridines (**4**, **8** and **13**) that are building blocks for drugs such as MK2 inhibitors (autoimmune diseases) and vericiguat (heart failure), and agrochemicals including the herbicide clodinafop^37–39^. For diazonium salts prone to decomposition, we developed a one-pot protocol using *tert*-butyl nitrite and LiBF_4_ enabling access to fluoroarenes (**4** and **9**) without the necessity to isolate the diazonium salt. For this purpose, LiBF_4_ was prepared from fluorspar upon treatment of HBF_4_^AGF^ with Li_2_CO_3_ (Supplementary Information).

Next, AGF-derived KF (KF^AGF^) (90% purity) was found to achieve high-yielding fluorination of chloroarene substrates using Me_4_NCl (5 mol%) and DMSO as solvent to provide fluoroarenes **14** to **17** (Fig. 4b). We noted that the performance of KF^AGF^ was comparable to commercial KF (99% purity; Supplementary Table 22). Aromatic fluorodenitration using AGF-derived Me_4_NF·^*t*^AmOH proceeded in DMSO (30−80 °C) to access 2,6-difluorobenzonitrile (**18**), 4-fluoronitrobenzene (**19**) and 2-fluorobenzonitrile (**20**) in high yield.

The successful cooperative activation of AGF in water and its application to the synthesis of fluoroarenes encouraged an investigation on the reactivity of lower-grade metallurgical fluorspar (metspar). These studies were performed with materials sourced from China (Metspar^I^ CaF_2_ (85%), SiO_2_ (10%), CaCO_3_ (<5%), S (0.12%), P (0.1%)), and Mexico (Metspar^II^ from CaF_2_ (88.98%), SiO_2_ (5.43%), CaCO_3_ (4.02%), Al_2_O_3_ (0.41%), Fe_2_O_3_ (0.24%), S (0.011%), P (0.023%), Pb (<0.001%)). Both metspar^I^ and metspar^II^ yielded the [B–F] products HBF_4_ and HBF_3_OH with an overall yield of 83% each, upon activation with B(OH)_3_ and H_2_Ox·2H_2_O at 50 °C for 15 h (Supplementary Information). The reaction of metspar with H_2_Ox·2H_2_O and SiO_2_ also enabled the preparation of metspar-derived KF (KF^M^) (53% (KF^M(I)^) and 63% (KF^M(II)^) yield, calculated from metspar I or II, respectively). Fluoroarenes **1**, **14** and **16** were prepared in good yield using HBF_4_^M(I)^ or KF^M(I)^ despite the reduced purity of metspar.

## Online content

Any methods, additional references, Nature Portfolio reporting summaries, source data, extended data, supplementary information, acknowledgements, peer review information; details of author contributions and competing interests; and statements of data and code availability are available at 10.1038/s41586-024-08125-1.

## Supplementary information

Supplementary Information Supplementary Sections 1–19 including Supplementary Text, Figures, Tables and Data – see Contents for details.

## Data Availability

All data supporting this work are included in Supplementary Information.
