# Supplementary material for: Fluorspar to fluorochemicals upon low-temperature activation in water
Source: Nature. 2024 Nov 13;635(8038):359–64. doi: 10.1038/s41586-024-08125-1 (PMC11560839; doi:10.1038/s41586-024-08125-1)
Supplement: Supplementary file 1 — Supplementary Sections 1–19 including Supplementary Text, Figures, Tables and Data – see Contents for details. [file 41586_2024_8125_MOESM1_ESM.pdf]

nature portfolio

https://doi.org/10.1038/s41586-024-08125-1

Supplementary information

Fluorspar to fluorochemicals upon low-temperature activation in water

In the format provided by the
authors and unedited

Fluorspar to fluorochemicals upon low-temperature activation in water

Supplementary Information

Immo Klose,[1,3] Calum Patel,[1,3] Anirban Mondal,[1,3] Andrew Schwarz,[2] Gabriele Pupo,[2] Véronique Gouverneur[1]*

[1] University of Oxford, Chemistry Research Laboratory, Oxford, OX1 3TA, UK; [2] FluoRok, The Quorum Alec Issigonis Way Oxford Business Park North, Oxford OX4 2JZ, UK.

[3] These authors contributed equally.

Correspondence to: veronique.gouverneur@chem.ox.ac.uk

Contents

1. Materials and Methods 5
2. Safety Statement 7
2.1. Aryl diazonium tetrafluoroborate salts 7
2.2. Hydrogen Fluoride (HF) 8
2.3. Hexafluorosilic acid (H_2SiF_6) 8
2.4. Dimethyl sulfoxide (Me_2SO, DMSO) 8
2.5. Calcium Oxalate (CaOx) and Boric acid [$B(OH)_3$] 8
3. Preliminary Experiments 9
3.1. Preliminary experiment using L-(+)-tartaric acid to prepare organotrifluoroborate salt 9
3.2. Preliminary ball milling experiments 9
3.2.1. Ball milling acid grade fluorspar with potassium oxalate 9
3.2.2. Ball milling potassium fluoride with calcium oxalate 10
3.3. Preliminary experiment using oxalic acid to prepare organotrifluoroborate salt 10
3.4. Activation of acid grade fluorspar with potassium oxalate in water 11
4. Brønsted acid evaluation for CaF_2 activation 12
5. Reaction Monitoring and Characterization 15
5.1. HF limit of detection 15
5.2. Reaction monitoring in the absence of Lewis Acid 15
5.3. Reaction monitoring in presence of Lewis Acid 17
5.3.1. Reaction monitoring in presence of $B(OH)_3$ 17
5.3.2. Reaction monitoring in presence of SiO_2 18
5.4. Reaction monitoring in absence of H_2Ox 18
5.5. Characterization of aqueous boron fluorine products (HBF_4, HBF_3OH and $HOxBF_2$) 19
5.6. Characterization of aqueous silicon fluorine products (H_2SiF_6, H_2SiF_5OH and H_2OxSiF_4) 21
5.7. Characterization of insoluble by-product formed in the reaction of acid grade fluorspar with H_2Ox and Lewis acid in water 25
6. Reaction Optimization 26
6.1. Optimization of HBF_4 preparation 26
6.1.1. Ratios of reagents 26
6.1.2. Reaction temperature 26
6.1.3. Reaction concentration 27
6.1.4. Use of oxalic acid dihydrate 27
6.2. Optimization of KF preparation 28
6.2.1. Reaction temperature 29
6.2.2. Reaction time 29
6.2.3. Reaction concentration 30
6.2.4. Equivalents of SiO_2 30
6.2.5. Use of oxalic acid dihydrate 31
6.2.6. Reassessment of acids for fluorspar activation using SiO_2 under optimized conditions 31
6.2.7. Quantification of [Si-F] species under optimized conditions ($H_2Ox{\cdot}H_2O$ as Brønsted acid) 32
7. Characterization of K_2SiF_6 from acid grade fluorspar 34

8. Synthesis of Fluorinating Reagents 35
8.1. Preparation of HBF_4 from acid grade fluorspar 35
8.2. Preparation of HBF_4 from metspar I (85% CaF_2) 35
8.3. Preparation of HBF_4 from metspar II (89% CaF_2) 36
8.4. Performance of prepared HBF_4 36
8.5. Preparation of KF from acid grade fluorspar 37
8.6. Preparation of KF from metspar I (85% CaF_2) 38
8.7. Preparation of KF from metspar II (89% CaF_2) 39
8.8. Purity Analysis of KF 41
8.8.1. Purity analysis by ^{19}F NMR 41
8.8.2. ICP OES 43
8.8.3. TGA-MS 43
8.9. Performance of prepared KF 44
8.10. Preparation of $R_4NF{\cdot}(ROH)_x$ from acid grade fluorspar 44
8.11. Performance of acid grade fluorspar derived $Me_4NF{\cdot}{}^t$AmOH 46
8.12. Preparation of NaF from acid grade fluorspar 46
8.13. Preparation of CsF from acid grade fluorspar 48
8.14. Performance of prepared CsF 50
9. Preparation of HBF_4 using oxalic acid dihydrate at 25 °C 50
10. Preparation of KF using oxalic acid dihydrate at 25 °C 50
11. Preparation of HBF_4 using sulfuric acid at 50 °C and 25 °C 52
12. Preparation of KF using sulfuric acid at 50 °C and 25 °C 53
12.1. Quantification of [Si-F] species formed (H_2SO_4 as Brønsted acid) 55
13. Synthesis of Aryldiazonium Tetrafluoroborate Salts 57
13.1. Safety Statement 57
13.2. Differential Scanning Calorimetry of Aryl Diazonium Tetrafluoroborate Salts 57
13.3. Recommended Process Temperature (T_{D24}) of Aryl Diazonium Tetrafluoroborate Salts 63
13.4. Impact Sensitivity and Explosive Propagation of Aryl Diazonium Tetrafluoroborate Salts 64
13.5. General procedure for the synthesis of aryl diazonium tetrafluoroborate (Method A) 65
13.6. Example procedure for the synthesis of aryl diazonium tetrafluoroborate using 1 equivalent of *tert*-amyl nitrite 69
13.7. Example procedure for the synthesis of aryl diazonium tetrafluoroborate with the addition of PVC as a phlegmatizing agent. 70
14. Synthesis of Aryl Fluorides by Balz-Schiemann Reaction 70
14.1. Safety Statement 70
14.2. General procedure for the dediazotization (Method B) 70
14.3. Example procedure for the dediazotization of aryl diazonium tetrafluoroborate in the presence of PVC as a phlegmatizing agent. 77
15. Synthesis of Aryl Fluorides by S_NAr Reaction 77
15.1. Safety Statement 77
15.2. S_NAr reaction of chloroarenes using KF (Method C) 77
15.3. S_NAr reactions using acid grade fluorspar derived $Me_4NF{\cdot}{}^t$AmOH (Method D) 79
16. One-pot fluoro-dediazotization using acid grade fluorspar derived $LiBF_4$ 81

16.1. Synthesis of $LiBF_4$ from acid grade fluorspar 81
16.2. Application of $LiBF_4$ in one-pot Balz Schiemann reaction 82
17. Stability of aqueous [B-F] and [Si-F] solutions prepared from acid grade fluorspar 83
18. NMR Spectra 84
18.1. Spectra for isolated products 84
18.2. Yields determined by internal standard 122
19. References 124

1. Materials and Methods

Unless otherwise stated, all reagents were purchased from commercial suppliers, used without further purification and stored under ambient conditions unless otherwise stated. Solvents were purchased from commercial suppliers and used as provided without further purification. Anhydrous oxalic acid ($C_2H_2O_4$, Sigma Aldrich, CAS 144-62-7), oxalic acid dihydrate ($C_2H_6O_6$, Sigma Aldrich, CAS 6153-56-6), boric acid (H_3BO_3, ≥ 99.5%, Sigma Aldrich, CAS 10043-35-3), silica gel (SiO_2, silica gel 60 particle size 0.040-0.063 mm, Merck, CAS 7631-86-9), potassium oxalate monohydrate ($C_2H_2K_2O_5$, 99%, Sigma Aldrich, CAS 6487-48-5), calcium oxalate monohydrate ($C_2H_2CaO_5$, 99%, Thermo Fisher Scientific, CAS 5794-28-5), potassium hexafluorosilicate (K_2SiF_6, ≥99.0%, Sigma Aldrich, CAS 16871-90-2), potassium hydroxide (KOH, 85% pellets, Sigma Aldrich, CAS 1310-58-3), cesium hydroxide monohydrate ($CsOH{\cdot}H_2O$, 95%, Fluorochem, CAS 35103-79-8), sodium hydroxide (NaOH, 97%, Sigma Aldrich, CAS 1310-73-2), lithium tetrafluoroborate ($LiBF_4$, 96%, Fluorochem, CAS 14283-07-9) and sodium fluoride (NaF, >98%, Honeywell, 7681-49-4) were used without drying and stored under ambient conditions. Anhydrous potassium fluoride (KF, 99%, Thermo Scientific Chemicals, CAS 7789-23-3) and anhydrous cesium fluoride (CsF, 99%, Sigma Aldrich, 13400-13-0) were used without drying and stored in a desiccator. Commercial tetrafluoroboric acid solution (48 wt. % in H_2O, Sigma Aldrich, CAS 16872-11-0) was used as received. Tetramethylammonium chloride ($C_4H_{12}NCl$, ≥ 99.0%, Sigma Aldrich, 75-57-0) was stored in a desiccator. Fluorspar (acid grade) was purchased from Mistral Industrial Chemicals (UK), sourced from Minersa group (Asturias region, Spain) and contains CaF_2 (> 97%), total carbonates (< 1.50%), SiO_2 (<1.00%), $BaSO_4$ (<0.50 %), Pb (< 0.10%), Fe_2O_3 (< 0.10%), S (< 0.15%), H_2O (< 1.0%).

Metspar was sourced from China (Luoyang Aurora Minechem. Co. Ltd, gifted by FluoRok) [Metspar^{I} CaF_2 (85%), SiO_2 (10%), $CaCO_3$ (<5%), S (0.12%), P (0.1%)], and Mexico (Mexichem Fluor S.A de C.V., gifted by Glencore UK) [Metspar^{II} from CaF_2 (88.98%), SiO_2 (5.43%), $CaCO_3$ (4.02%), Al_2O_3 (0.41%), Fe_2O_3 (0.24%), S (0.011%), P (0.023%), Pb (<0.001%)].

Deuterated solvents were purchased from VWR Chemicals or Sigma Aldrich and used as received.

Thin layer chromatography (TLC) was carried out on silica gel pre-coated aluminum sheets (Merck Kieselgel 60 F254 plates) and visualized using ultraviolet light of wavelength 254 nm or potassium permanganate stain. Flash column chromatography (FFC) was performed on Merck silica gel (60, particle size 0.040-0.063 mm).

Reactions using fluorspar and Brønsted acids were performed in polypropylene (PP) vessels sealed with a low-density polyethylene cap or polytetrafluoroethylene (PTFE) round bottom flask (RBF) under an atmosphere of air, unless otherwise stated. Reported concentrations refer to solution volumes at room temperature. Fluorination reactions using KF, $Me_4NF{\cdot}{}^{t}AmOH$ or HBF_4 were stirred at 1000 rpm using an IKA heating plate, aluminum block and borosilicate glass vials.

^{1}H NMR, ^{13}C NMR, ^{19}F NMR, ^{11}B NMR and ^{29}Si NMR spectra were recorded on Bruker AVIIIHD 400, AVIIIHD 500, AVII 500 or AV NEO 600. ^{1}H NMR spectra were recorded at 400 or 500 MHz. ^{13}C NMR spectra were recorded at 101 or 126 MHz with ^{1}H decoupling, ^{19}F NMR spectra were recorded at 377 or 471 MHz, ^{11}B NMR spectra were recorded at 128 MHz, ^{29}Si NMR spectra were recorded at 99 MHz. ^{1}H NMR, ^{13}C NMR,^{19}F NMR, ^{11}B NMR and ^{29}Si NMR spectral data are reported as chemical shifts (δ) in parts per million (ppm) relative to the solvent peak using the Bruker internal referencing procedure (edlock). Coupling constants, *J*, are reported in Hz to the nearest 0.1 Hz. Unless otherwise stated, ^{13}C spectra are ^{1}H decoupled and reported coupling constants for ^{13}C spectra correspond to ^{19}F–^{13}C heteronuclear coupling. Data are reported as follows: chemical shift, multiplicity (s = singlet, d = doublet, t = triplet, q = quartet, pent = pentet, hept = heptet, br = broad, m = multiplet), coupling constants (Hz) and integration. NMR (nuclear magnetic resonance) spectra were processed with MestReNova 14.1.2. Quantitative NMR analysis was determined using 4-fluoroanisole or sodium triflate (NaOTf) as an internal standard. The standard was added to the crude reaction mixture and an aliquot was taken to be analyzed by quantitative ^{19}F NMR and ^{1}H NMR.

High resolution mass spectra (HRMS) were determined on a Thermo Exactive High-Resolution Orbitrap FTMS mass spectrometer (ESI+ or ESI-). Agilent 5977B was used for GC-MS (EI+). Some compounds were found to be unstable under a variety of MS ionization methods (CI, EI, ESI, GC-MS) and therefore no HRMS could be obtained for them.

X-ray powder diffraction (PXRD) data was collected at room temperature using a Bruker D8 Advance X-ray diffractometer (Bragg−Brentano geometry); the radiations Cu Kα1,2 were used.

TGA-MS data was recorded using a PerkinElmer TGA 8000 and Hiden Analytical HPR-20 EGA mass spectrometer for MS. Inductively Coupled Plasma Optical Emission spectroscopy (ICP-OES) for microanalyses was carried out by MEDAC Ltd.

Teflon NMR thin wall (5 mm) liner for reaction monitoring experiments was purchased from NEW ERA.

All DSC analysis were performed on a DSC 2910 Differential Scanning Calorimeter (TA Instruments) equipped with a liquid nitrogen cooling accessory. All Data analysis was conducted using the software TA Instruments Universal Analysis 2000 version 4.5.0.5. Sample preparation used approximately 3−4 mg of the arenediazonium salt. After equilibration, the DSC cells were heated in a ramp from 25 to 250 °C with a heating rate of 20 °C /min under an inert atmosphere of nitrogen. Differential Scanning Calorimetry (DSC) measurements were performed on a DSC25 (TA Instruments) under a nitrogen flow (80 mL min^{-1}). Samples were prepared in hermetic aluminum pans using 2-4 mg of each salt. The samples were equilibrated at 25°C and then heated to 250 °C at a rate of 20 °C min-1. Peak integrations, peak temperatures ($T_{initial}$ and T_{onset}) and enthalpy (Q) were calculated using the TRIOS™ software. The DSC was calibrated using indium and zinc standards.

The energy change was monitored via a constant temperature ramp, providing information on the initial temperature of decomposition ($T_{initial}$, defined by the temperature where the heat flow is >0.01 W/g from the baseline). This decomposition process is defined as exothermic and appears as a positive peak in DSC data (decreased heat flow to the sample). Conversely, if any melting event occurs, which corresponds to an endothermic process in the sample, the energy supplied to the sample must increase in order to maintain the temperature, hence a negative peak appears.

2. Safety Statement

The procedures reported in this work are intended for use only by individuals with proper training in experimental chemistry. Reaction set-up, and chemical-specific hazards are highlighted in bold with "**CAUTION!**" notes in the procedures reported in this supplementary information. It is important to note that the absence of a caution note does not imply that no significant hazards are associated with the chemicals involved in that procedure.

2.1. Aryl diazonium tetrafluoroborate salts

Aryl diazonium salts are considered highly energetic compounds and are presumed to be thermally unstable, sensitive to friction, and shock-prone. It cannot be generalized that these salts are always stable due to the presence of the tetrafluoroborate counter-ion. Potential **detonation** can be caused by heat, friction or shock. In their solid state, some diazonium salts pose a risk of **violent decomposition** and can rapidly release tremendous amounts of destructive energy. Numerous incidents in both industrial and laboratory settings have been attributed to this class of chemicals[40].

For safety, we assessed the **thermal stability** of each diazonium salt (**S1-S13**) to assess **thermal stability** including **DSC analysis, recommended process temperature (T_{D24}), impact sensitivity (IS)** and **explosive propagation (EP)**.

Using the thermal analysis results obtained by Differential Scanning Calorimetry (DSC) (included in **Section 13.2**), the thermal stability, in particular the safe process temperature of diazonium salts **S1**-**S13** was evaluated by determining the **T_{D24} value**. The T_{D24} consists in an estimated temperature (°C) at which the maximum rate of decomposition of a compound, under adiabatic conditions (without energy exchanges) is achieved after 24 h. Compounds with T_{D24} below ambient temperature must be stored at -18 °C (in the dark), otherwise thermal runaway may occur, and explosions may be observed. Full T_{D24} data can be found in **Section 13.3**.

Impact sensitivity (IS) and the potential for **explosive propagation** (EP) has been determined for all the diazonium salts prepared using correlations outline by Yoshida and Pfizer[41]. Full IS and EP data can be found in **Section 13.4**.

All aryl diazonium tetrafluoroborate salts must be prepared in the presence of a **blast shield**, independent of the protocol applied.

The presence of nitrous acid can lower the decomposition temperature of aryl tetrafluoroborate diazonium salt [42]. During diazotization, minimize the presence of nitrous acid by combining amine and acid first, before adding the *tert*-butyl nitrite. Check for the excess of nitrous acid by starch–potassium iodide paper and neutralization using sulfamic acid.

All diazonium salts were immediately stored in the **freezer (-18 °C)** following isolation, unless mentioned otherwise (see specific procedures in **Section 13** for **S3**, **S4**, **S6**, **S8**, **S9** and **S10**). A **plastic spatula** should be used when handling the aryl diazonium tetrafluoroborate salt and **never a metal spatula**. Aryl diazonium salts can be dried under a stream of N_2 gas but never under reduced pressure or by heating.

Residual diazonium compounds in filtrates formed during diazotizations should be analyzed by ^{1}H and ^{19}F NMR spectroscopy. Any **residual diazonium compound should be quenched** using triethylamine. After fluoro-dediazotisation of the diazonium salt, the crude reaction mixture is treated with saturated aqueous $NaHCO_3$ solution to neutralize unreacted diazonium salt.

Diazonium salts **S3, S6, S8,** and **S11** exhibit high impact sensitivity as calculated by the Pfizer correlation and should only be prepared on ≤1.0 mmol scale and used immediately. For these salts, an inert material (phlegmatizing agent) can be added to stabilize the diazonium salt and reduce the risk of spontaneous decomposition (see **Section 13.7**).

For safety purposes, please refer to the guidelines for the preparation of aryl tetrafluoroborate diazonium salts outlined by Gorman and co-workers in ""**Reactive chemical hazards of diazonium salts**" [43].

2.2. Hydrogen Fluoride (HF)

Hydrogen fluoride (HF) is a highly corrosive and toxic substance that will corrode glassware. Safe handling should be conducted with plastic syringes and metal needles, with KOH (aq.) employed to quench excess HF. Always handle HF while wearing gloves and in a fume hood. Always have calcium gluconate gel nearby and apply immediately and liberally on skin when exposed to HF. Reactions should be conducted in polypropylene vessels or directly in Teflon NMR thin wall (5 mm) liners, as demonstrated in this work.

2.3. Hexafluorosilic acid (H_2SiF_6)

Hexafluorsilicic acid (H_2SiF_6) can release **HF** upon evaporation, therefore aqueous solutions of H_2SiF_6 should not be concentrated. H_2SiF_6 is corrosive. Safe handling should be conducted with plastic syringes and metal needles, with KOH (aq.) employed to quench excess H_2SiF_6.

2.4. Dimethyl sulfoxide (Me_2SO, DMSO)

DMSO undergoes thermal decomposition at temperatures around its boiling point of 189 °C[44]. The presence of impurities and/or acidic substances can cause it to decompose at significantly lower temperature and potentially result in uncontrollable autocatalytic decomposition of DMSO, leading to thermal runaway or even explosions. All S_NAr reactions using KF and DMSO as solvent (5 mL) were carried out ≤ 130 °C (1 mmol scale). All S_NAr reactions using $Me_4NF{\cdot}t$AmOH and DMSO as solvent (5 mL) were carried out ≤ 80 °C (1 mmol scale). All S_NAr reactions using DMSO with heating should be conducted in the presence of a **blast shield**. For large scale experimentation, DMSO should be avoided and a suitable alternative should be used.

2.5. Calcium Oxalate (CaOx) and Boric acid [$B(OH)_3$]

Calcium oxalate can produce sores and numbing on ingestion and may even be fatal. Boric acid is poisonous if taken internally or inhaled in large quantities.

All hazardous materials (solid, liquid, or gaseous) should be handled using the standard work procedures described in the "Prudent Practices in the Laboratory"[45]. All chemical waste should be disposed of in accordance with local regulations. For general guidelines for the management of chemical waste, see Chapter 8 of "Prudent Practices in the Laboratory".

3. Preliminary Experiments

3.1. Preliminary experiment using L-(+)-tartaric acid to prepare organotrifluoroborate salt

The synthesis of organotrifluoroborate salts using potassium fluoride (KF) as the nucleophilic fluoride source was reported by Lloyd-Jones and Lennox [14]. L-(+)-Tartaric acid serves as an alkali-metal sponge and shifts the equilibrium towards the organotrifluoroborate salt with precipitation of potassium bitartrate. As a case study, we chose to investigate the fluorination of 4-fluorophenylboronic acid using acid grade fluorspar (CaF_2, > 97.0%, Minersa Group) and L-(+)-tartaric acid.

To a glass reaction vessel was added 4-fluorophenylboronic acid (14 mg, 0.1 mmol, 1.0 equiv), MF (4.0 mmol, 4.0 equiv) and MeCN (0.4 mL). H_2O (40 µL) was added and the reaction was stirred for 15 min. L-(+)-tartaric acid (31 mg, 0.21 mmol, 2.05 equiv) was dissolved in THF (150 µL) and added dropwise to the reaction mixture. The reaction was stirred at 25 °C. After 20 minutes, NaOTf (13.6 mg, 0.1 mmol) was added as an internal standard and an aliquot of the reaction mixture was diluted in D_2O for NMR analysis. Reaction yield was determined by quantitative ^{19}F NMR spectroscopy.

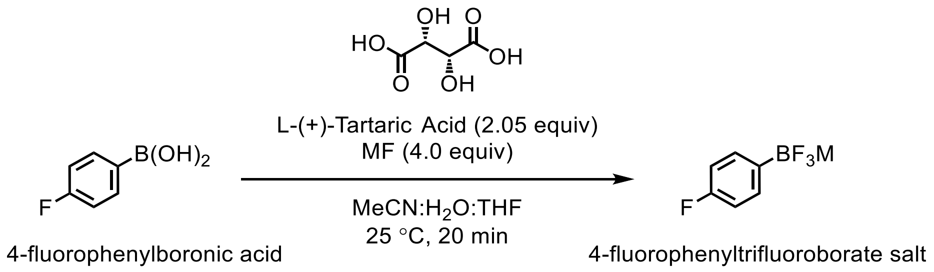

Table S1. Comparison of fluoride sources in fluorination of 4-fluorophenylboronic acid

Entry	MF	$ArB(OH)_3$ (%)	$ArBF_3M$ (%)
1	KF	0	100
2	CaF_2 (acid grade fluorspar)	99	0

Quantitative conversion of 4-fluorophenylboronic acid to potassium 4-fluorophenyltrifluoroborate was observed using KF as the fluoride source. Acid grade fluorspar did not react under identical reaction conditions.

3.2. Preliminary ball milling experiments

3.2.1. Ball milling acid grade fluorspar with potassium oxalate

The reaction between acid grade fluorspar (CaF_2, > 97.0%, Minersa Group) with potassium oxalate monohydrate (99%, Sigma Aldrich) was investigated under mechanochemical conditions (ball milling). We considered that ion metathesis between CaF_2 and $K_2C_2O_4$ (K_2Ox) might occur to afford calcium oxalate (CaC_2O_4, CaOx) and KF, or derivatives thereof. To a 15 mL stainless-steel milling jar was added a stainless-steel ball (7 g), acid grade fluorspar (160.1 mg, 2.05 mmol, 1.0 equiv) and potassium oxalate monohydrate (378 mg, 2.05 mmol, 1.0 equiv). The jar was closed and securely fitted to the mill which was set for 3 h at the frequency of 30 Hz. Upon completion, the jar was opened and the white powder was collected and stored in a borosilicate glass vial in a desiccator. The powder was analyzed by PXRD. Crystalline phases were identified as CaF_2 and $K_2C_2O_4 \cdot H_2O$.

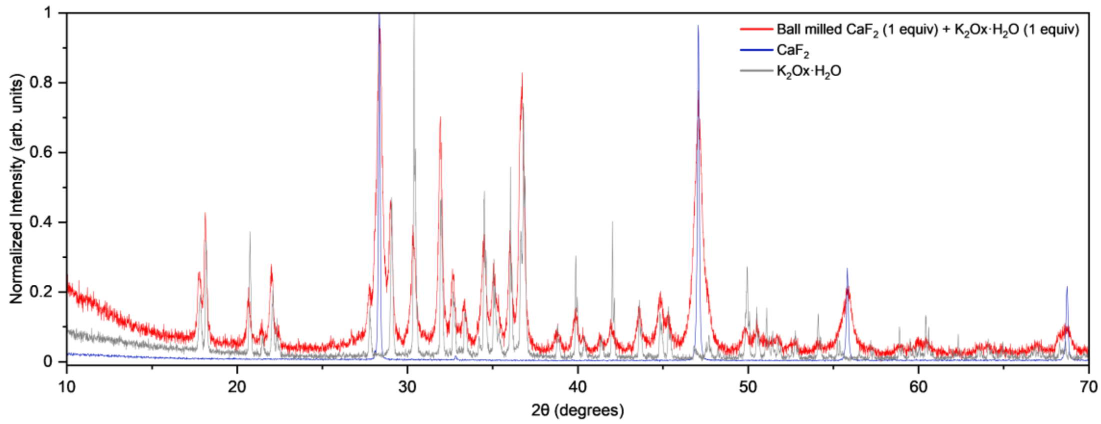

Figure S1. Measured X-ray powder diffraction data collected at room temperature of ball milled acid grade fluorspar (CaF_2) with $K_2C_2O_4{\cdot}H_2O$ (red), acid grade fluorspar (CaF_2) (blue) and $K_2C_2O_4{\cdot}H_2O$ (grey).

3.2.2. Ball milling potassium fluoride with calcium oxalate

The reaction between anhydrous KF (99%, Thermo Scientific Chemicals) with calcium oxalate monohydrate (99%, Thermo Fisher Scientific) was investigated under mechanochemical conditions (ball milling). To a 15 mL stainless-steel milling jar was added a stainless-steel ball (7 g), anhydrous KF (220 mg, 3.78 mmol, 2.0 equiv) and calcium oxalate monohydrate (276 mg, 1.89 mmol, 1.0 mmol). The jar was closed and securely fitted to the mill which was set for 3 h at the frequency of 30 Hz. Upon completion, the jar was opened and the white powder was collected and stored in a borosilicate glass vial in a desiccator. The powder was analyzed by PXRD. Crystalline phases were identified as CaF_2 and $K_2C_2O_4{\cdot}H_2O$.

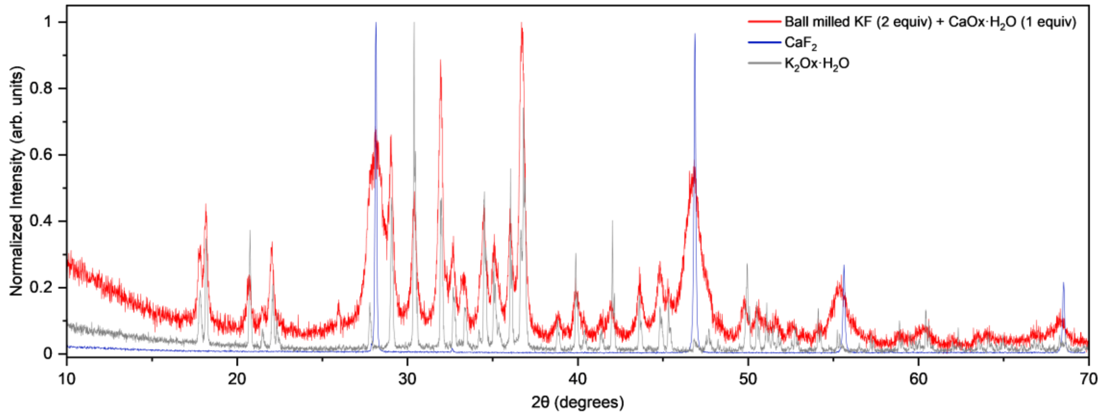

Figure S2. Measured X-ray powder diffraction data collected at room temperature of ball milled anhydrous KF with CaOx·H_2O (red), acid grade fluorspar (CaF_2) (blue) and $K_2C_2O_4{\cdot}H_2O$ (grey).

Under mechanochemical conditions, the formation of CaF_2 and $K_2C_2O_4$ is favored.

3.3. Preliminary experiment using oxalic acid to prepare organotrifluoroborate salt

To a glass reaction vessel was added 4-fluorophenylboronic acid (17.5 mg, 0.125 mmol, 1.0 equiv), acid grade fluorspar (39 mmol, 0.5 mmol, 4.0 equiv) and anhydrous oxalic acid (45 mg, 0.5 mmol, 4.0 equiv) or L-(+)-tartaric acid (75 mg, 0.5 mmol, 4.0 equiv). H_2O (0.4 mL) was added and the reaction was stirred for 15 min. The reaction was stirred at 70 °C. After 1 h, sodium triflate (13.6 mg, 0.1 mmol) was as an internal standard and an aliquot of the reaction mixture was diluted in D_2O for NMR analysis. Reaction yield was determined by quantitative ^{19}F NMR spectroscopy.

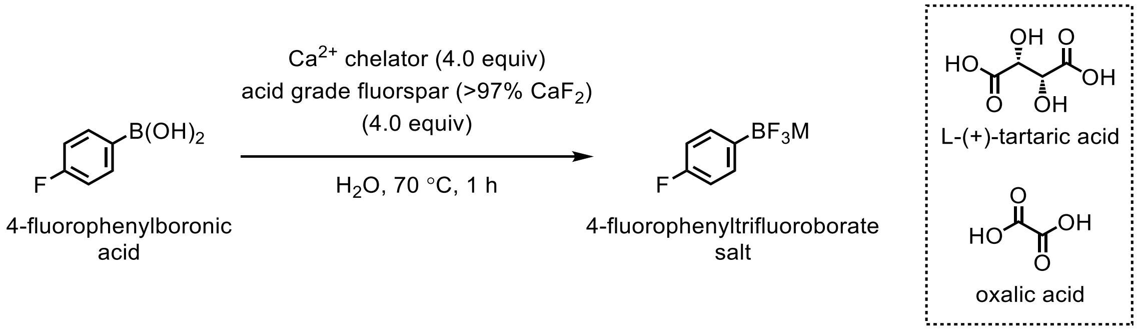

Table S2. Comparison of L-(+)-tartaric acid and oxalic acid as Ca^{2+} chelators

Entry	Ca^{2+} chelator	$ArBF_3M$ (%)
1	L-(+)-tartaric acid	trace
2	Oxalic acid	10

Potassium 4-fluorophenyltrifluoroborate was afforded in 10 % NMR yield using anhydrous oxalic acid as the Ca^{2+} chelator. L-(+)-tartaric acid was found to be a less effective Ca^{2+} chelator under these conditions.

3.4. Activation of acid grade fluorspar with potassium oxalate in water

The reaction between acid grade fluorspar (CaF_2, > 97.0%, Minersa Group) with potassium oxalate monohydrate (99%, Sigma Aldrich) was investigated in H_2O at 70 °C. We considered that ion metathesis may occur to afford calcium oxalate (CaOx) and KF.

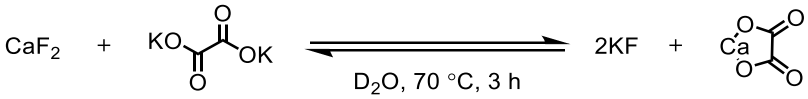

To a reaction vial was added acid grade fluorspar (10 mg, 0.125 mmol, 1.0 equiv), potassium oxalate monohydrate (21 mg, 0.125 mmol, 1.0 equiv) and D_2O (0.4 mL). The reaction was stirred at 70 °C for 3 h. NaOTf (4 mg, 0.023 mmol) was as an internal standard and an aliquot of the reaction mixture was diluted in D_2O for NMR analysis (quantitative ^{19}F NMR spectroscopy). KF was not detected by ^{19}F NMR spectroscopy.

To a reaction vial was added potassium fluoride (15 mg, 0.25 mmol, 2.0 equiv), calcium oxalate monohydrate (18.3 mg, 0.125 mmol, 1.0 equiv) and D_2O (0.4 mL). The reaction was stirred at 70 °C for 3 h. NaOTf (4 mg, 0.023 mmol) was as an internal standard and an aliquot of the reaction mixture was diluted in D_2O for NMR analysis (quantitative ^{19}F NMR spectroscopy). Under these reaction conditions, only 42% of KF remained by ^{19}F NMR spectroscopy.

4. Brønsted acid evaluation for CaF_2 activation

To a 15 mL conical sterile polypropylene (PP) tube was added acid grade fluorspar (344 mg, 4.40 mmol, 1.1 equiv.), $B(OH)_3$ (124 mg, 2.0 mmol, 0.5 equiv), acid activator (8.0 mmol, 2.0 equiv. for 'mono-acids', 4.0 mmol, 1.0 equiv. for 'di- and oligo-acids') and H_2O (1 mL). The tubes were capped. Each reaction was stirred at 50 °C for 15 h. The suspension was allowed to settle and the total amount of HBF_4 and HBF_3OH was assessed by quantitative ^{19}F NMR spectroscopy (D_2O) using sodium triflate (NaOTf) as internal standard (as shown in **Figure S3**). The yield of B–F products [HBF_4 + HBF_3OH] are given in **Table S3** to **Table S6**.

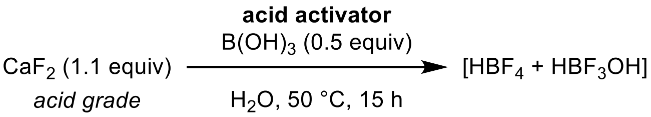

Table S3. Yields of B-F products [HBF_4 + HBF_3OH] from acid grade fluorspar using monoacids

	HO_4Cl	HCl	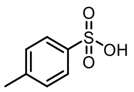	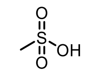	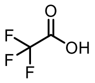
	perchloric acid	hydrochloric acid	tosic acid	methanesulfonic acid	trifluoroacetic acid
[HBF_4 + HBF_3OH]	26% + 13%	40% + 20%	18% + 10%	38% + 27%	0%
pK_a	-15.0	-6.2	-2.8	-1.9	-0.3
	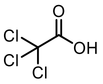	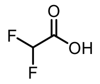	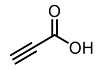	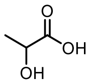	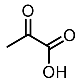
	trichloroacetic acid	difluoroacetic acid	propiolic acid	lactic acid	pyruvic acid
[HBF_4 + HBF_3OH]	8% + 1%	3% + 2%	2% + 0%	2% + 2%	0%
pK_a	0.7	1.3	1.9	2.5	2.5
	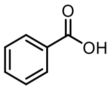	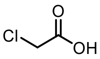	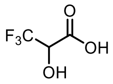	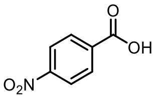	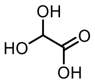
	benzoic acid	chloroacetic acid	trifluorolactic acid	4-nitrobenzoic acid	2,2-dihydroxyacetic acid
[HBF_4 + HBF_3OH]	0%	0%	0%	0%	4% + 2%
pK_a	2.5	2.9	2.9	2.9	3.2
	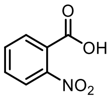	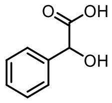	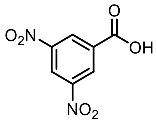	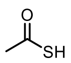	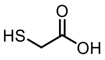
	2-nitrobenzoic acid	mandelic acid	3,5-nitrobenzoic acid	thioacetic acid	mercaptoacetic acid
[HBF_4 + HBF_3OH]	0%	3% + 4%	0%	0%	1% + 0%
pK_a	3.2	3.4	3.4	3.4	3.8
	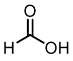	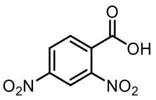	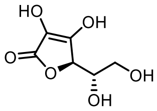	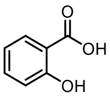	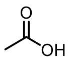
	formic acid	2,4-nitrobenzoic acid	ascorbic acid	salicylic acid	acetic acid
[HBF_4 + HBF_3OH]	1% + 0%	1% + 0%	1% + 0%	0%	0%
pK_a	3.7	3.9	4.2	4.2	4.7

Table S4. Yields of B-F products [HBF_4 + HBF_3OH] from acid grade fluorspar using diacids

	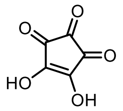	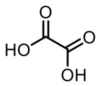	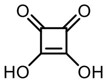	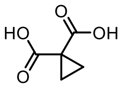	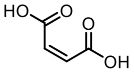
	croconic acid	oxalic acid	squaric acid	cyclopropyl malonic acid	*(Z)*-butenedioic acid
[HBF_4 + HBF_3OH]	36% + 11%	76% + 20%	18% + 9%	5 % + 6%	2% + 2%
pK_a	0.8, 2.2	1.3, 4.1	1.5, 3.4	1.8, 4.0	1.9, 6.0
	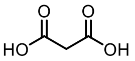	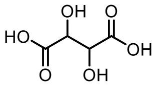	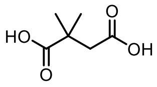	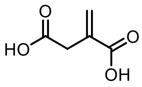	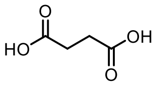
	malonic acid	tartaric acid	2,2-dimethylsuccinic acid	2-methylenesuccinic acid	succinic acid
[HBF_4 + HBF_3OH]	1% + 1%	1% + 1%	0%	0%	0%
pK_a	2.9, 5.7	3.0, 4.3	4.1, 5.4	3.8, 5.6	4.2, 5.6
	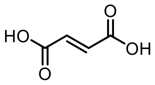	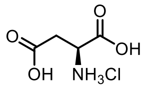	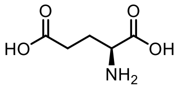	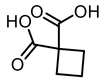	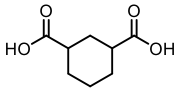
	E-butenedioic acid	L-aspartic acid HCl	L-Aspartic acid	cyclobutane-1,1-dicarboxylic acid	cyclohexane-1,3-dicarboxylic acid
[HBF_4 + HBF_3OH]	0%	0% + 1%	0%	0% + 1%	0%
pK_a	3.0, 4.4	2.0, 3.9	4.3, 10.0	3.1, 5.9	4.3, 6.0

Table S5. Yields of B-F products [HBF_4 + HBF_3OH] from acid grade fluorspar using inorganic di-acids and oligo-acids.

	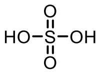	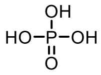
	sulfuric acid	phosphoric acid
[HBF_4 + HBF_3OH]	49% + 20%	0% + 4%
pK_a	-2.8, 2	2.1, 7.2

Table S6. Yields of B-F products [HBF_4 + HBF_3OH] from acid grade fluorspar using oligo-acids or aromatic acids

	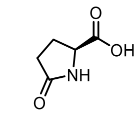 L-pyroglutamic acid	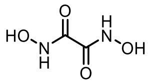 oxalylhydroxamic acid	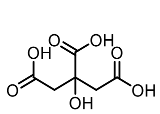 citric acid	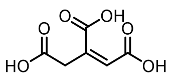 *cis*-aconitic acid	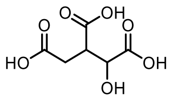 isocitric acid
[HBF_4 + HBF_3OH]	0% + 1%	0%	3% + 1%	0% + 1%	0% + 1%
pK_a	3.5	9.0	3.1, 4.7	3.8, 5.6	3.4, 4.6
	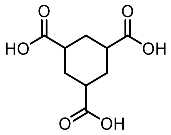 1,3,5-cyclohexane-tricarboxylic acid	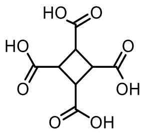 1,2,3,4-cyclobutanetetra-carboxylic acid	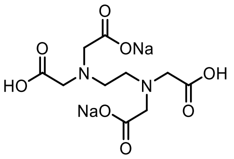 disodium ethylenediamine -tetraacetic acid	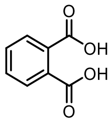 phthalic acid	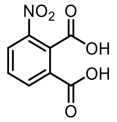 3-nitrophthalic acid
[HBF_4 + HBF_3OH]	0%	2% + 1%	0%	0%	0% + 1%
pK_a	4.0, 6.0	1.8, 4.4	6.2, 10.2	3.0, 5.4	1.9
	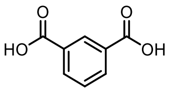 isophthalic acid	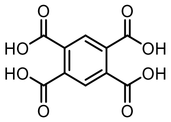 pyromellitic acid			
[HBF_4 + HBF_3OH]	0% + 1%	8% + 5%			
pK_a	3.5, 4.6	1.9, 2.9			

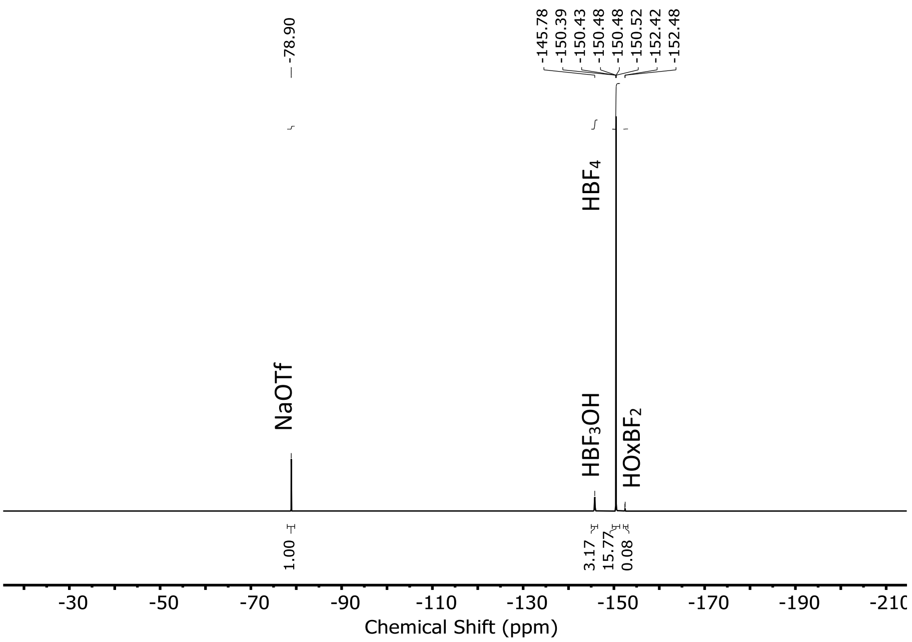

Figure S3 ^{19}F quantitative NMR (D_2O) of crude reaction mixture of acid grade fluorspar (CaF_2), H_2Ox and $B(OH)_3$ (2.0 mmol). HBF_4 (-150.3 ppm), HBF_3OH (-145.6 ppm) and trace $HOxBF_2$ (-152.4 ppm) produced. 0.13 mmol of NaOTf used as internal standard (-78.9 ppm). ^{19}F NMR Yields of HBF_4 and HBF_3OH are 76% and 20%, respectively.

5. Reaction Monitoring and Characterization

CAUTION! *HF is a corrosive and toxic substance that will corrode glassware. Safe handling can be conducted with plastic syringes and metal needles, with KOH (aq.) employed to quench excess HF. Always handle HF while wearing gloves and in a fume hood. Always have calcium gluconate gel nearby and apply immediately and liberally on skin exposed to HF. The following reactions were conducted in polypropylene vessels or directly in Teflon NMR thin wall (5 mm) liners.*

5.1. HF limit of detection

To evaluate the threshold at which sensitivity limits the capability of NMR to detect HF, a series of experiments were undertaken to determine the limit of detection (LOD) of HF in H_2O:D_2O 90%:10% at (470 MHz, 32 scans, d1 = 50 s). A series of samples were created with concentrations of HF (48% aq. HF) ranging from 0.005 M to 0.5 M. Signals for HF within the range of -152.2 to -159.9 ppm are observed across all concentrations measured. For integration errors < 1%, a signal-to-noise ratio (SNR) ≥150 is acceptable [46]. SNR was calculated from the spectra using an MNova software tool [47].

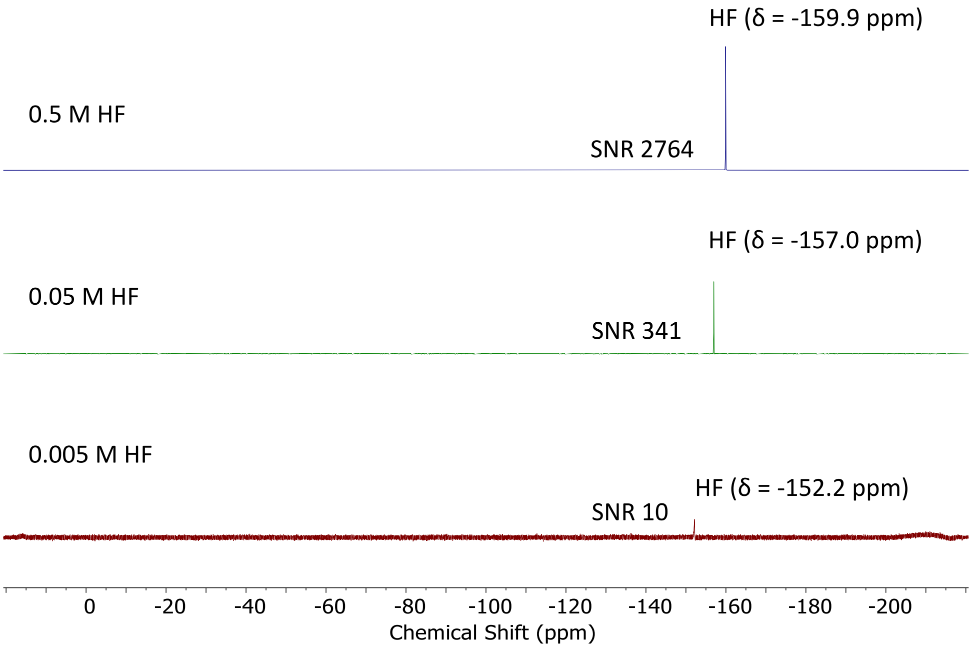

Figure S4. ^{19}F NMR spectra (stacked, 470 MHz, D_2O:H_2O 10%:90%) of HF (0.005 to 0.5 M) with SNR.

5.2. Reaction monitoring in the absence of Lewis Acid

The reaction between acid grade fluorspar (CaF_2, > 97.0%, Minersa Group) with anhydrous oxalic acid in D_2O was monitored at 50 °C over a period 8 h. Into a Teflon NMR thin wall (5 mm) liner was added acid grade fluorspar (97% CaF_2, 40 mg, 0.5 mmol, 1.1 equiv), anhydrous oxalic acid (41 mg, 0.45 mmol, 1.0 equiv), sodium triflate (10 mg) and D_2O (0.5 mL). The Teflon liner was plugged tightly with a Teflon cap and inserted through a custom-built holder (filled with ice) and then placed an ultrasonic bath filled with water heated to 50 ˚C (see **Figure S5**). The sample was sonicated and ^{19}F NMR experiments (470 MHz, 64 scans, d1 = 70 s) were conducted at various time points (**Figure S6**). At these time points, the Teflon liner was removed from the water bath and placed into an ice bath (0 ˚C). The suspension was allowed to settle before inserting the Teflon liner into a glass NMR tube. The samples were then analyzed by ^{19}F NMR spectroscopy and once completed, heating at 50 °C with sonication was resumed until the next time point.

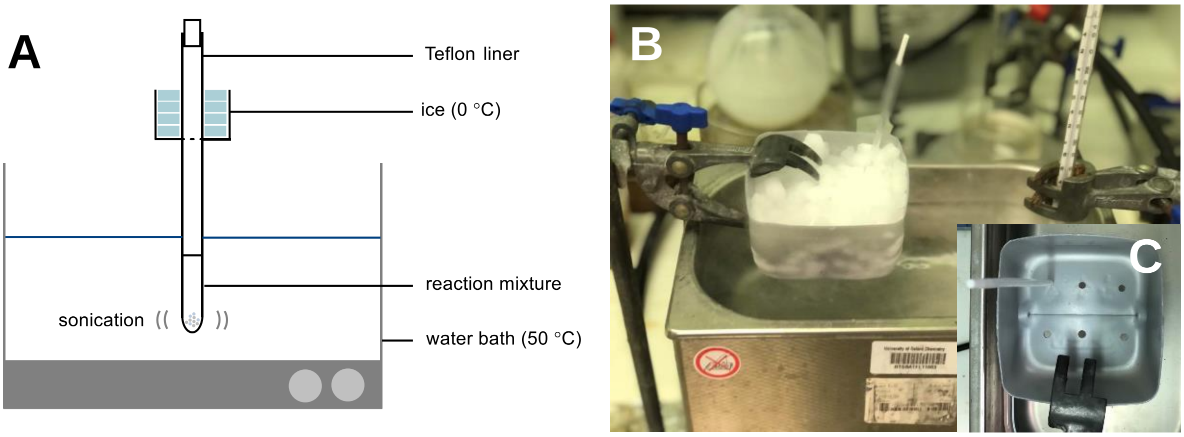

Figure S5. Experimental setup for HF monitoring experiment by ^{19}F NMR. (A) Schematic of experiment setup; (B) photograph of experimental set up; (C) Teflon NMR holder.

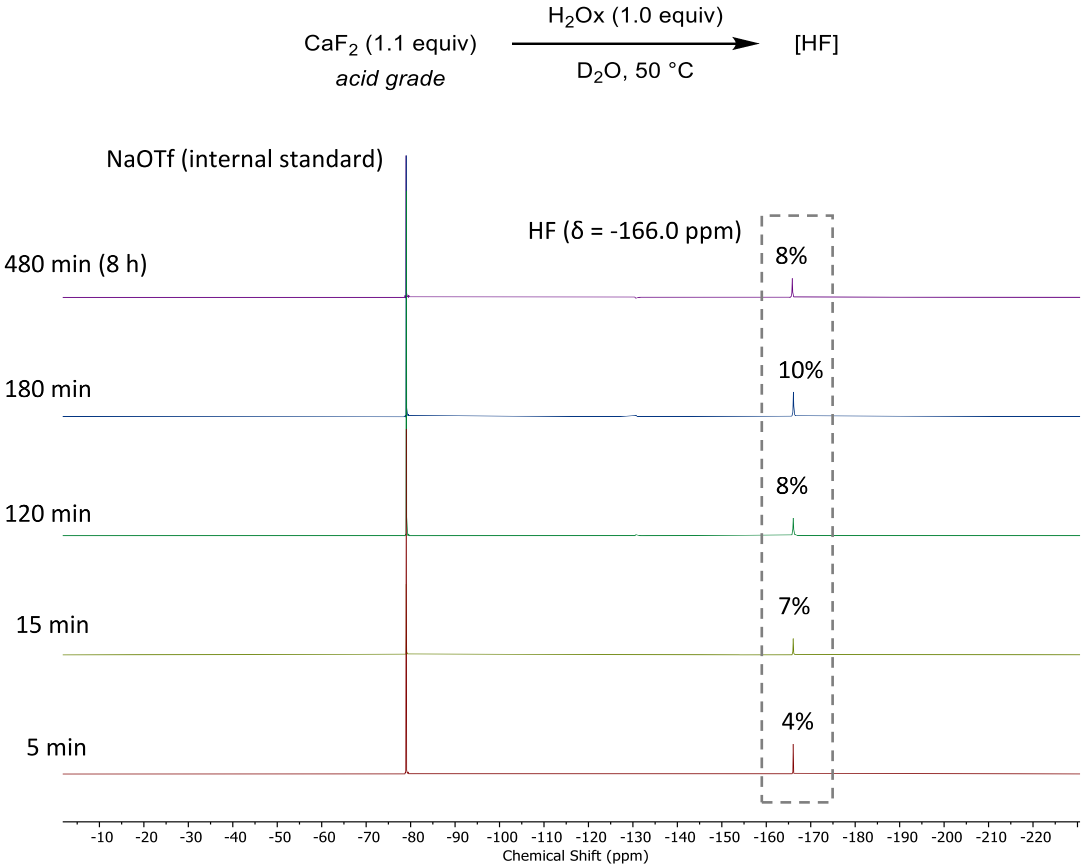

Figure S6. Reaction of acid grade fluorspar (CaF_2) with H_2Ox in D_2O at 50 °C monitored by ^{19}F NMR. Singlet diagnostic of HF observed at -166.0 ppm. Sodium triflate internal standard at -78.0 ppm.

Table S7. HF (%) quantified over course of reaction between acid grade fluorspar (CaF_2) with H_2Ox in D_2O at 50 °C. Signal-to-noise ratio (SNR) provided.

Entry	Time (min)	HF (%)	HF mmol	HF conc. (M)	SNR
1	5	4	0.033	0.07	280
2	15	7	0.066	0.13	178
3	120	8	0.070	0.14	159
4	180	10	0.089	0.18	338
5	480	8	0.075	0.15	219

5.3. Reaction monitoring in presence of Lewis Acid

The reaction between acid grade fluorspar (CaF_2, > 97.0%, Minersa Group) with anhydrous oxalic acid in D_2O in the presence of $B(OH)_3$ (or SiO_2) was monitored at 50 °C over a period of 3 hours. Into a Teflon NMR thin wall (5 mm) liner was added acid grade fluorspar (97% CaF_2, 40 mg, 0.5 mmol, 1.1 equiv), anhydrous oxalic acid (41 mg, 0.45 mmol, 1.0 equiv), sodium triflate (10 mg), $B(OH)_3$ (14 mg, 0.23 mmol, 0.5 equiv) or SiO_2 (18 mg, 0.32 mmol, 0.7 equiv) and D_2O (0.5 mL). The Teflon liner was plugged tightly with a Teflon cap and inserted through a custom-built holder (filled with ice) and then placed a water bath heated at 50 ˚C. The sample was sonicated and ^{19}F NMR experiments (470 MHz, 64 scans, d1 = 70 s) were conducted at various time points (**Figure S7** and **Figure S8**). The suspension was allowed to settle before inserting the Teflon liner into a glass NMR tube. The samples were then analyzed by ^{19}F NMR spectroscopy and once completed, heating at 50 °C with sonication was resumed until the next time point.

5.3.1. Reaction monitoring in presence of $B(OH)_3$

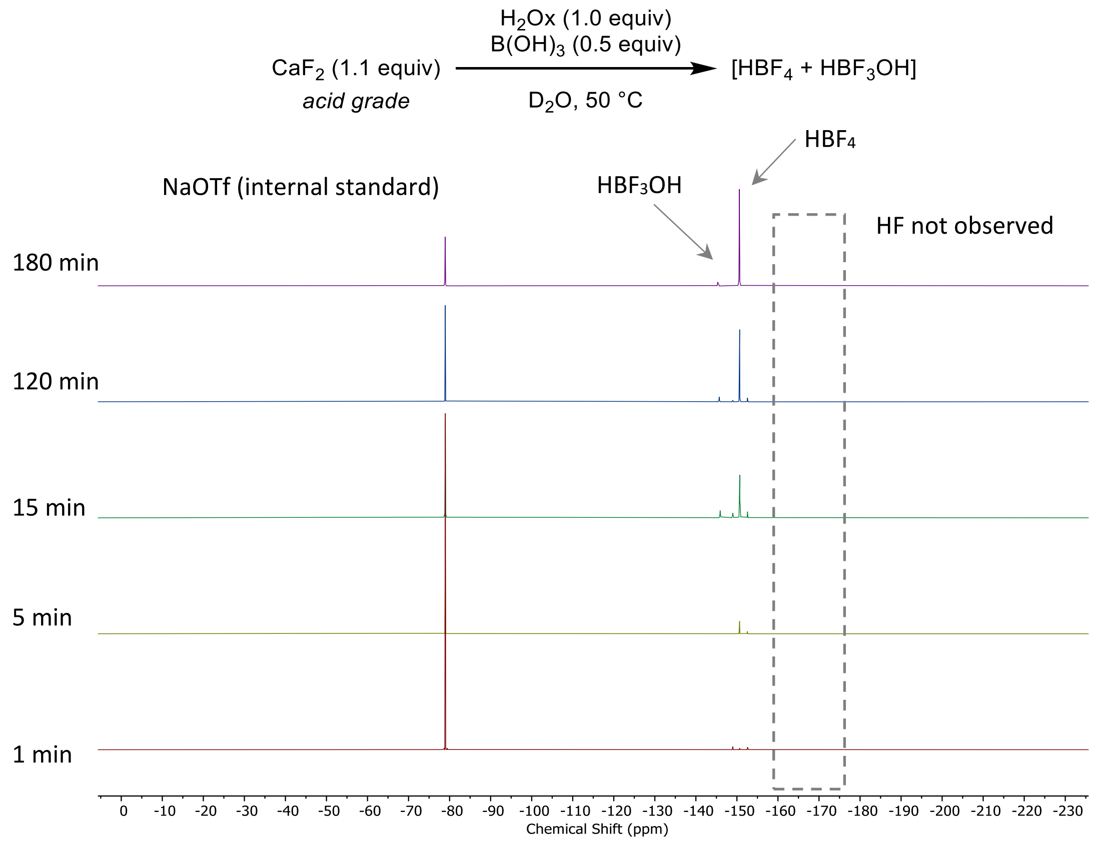

Figure S7. Reaction of acid grade fluorspar (CaF_2) with H_2Ox and $B(OH)_3$ in D_2O at 50 °C monitored by ^{19}F NMR (D_2O). HBF_4 at -151.0 ppm and HBF_3OH at -145.3 [19]. Sodium triflate internal standard at -78.0 ppm.

Table S8. HBF_4 (%) and HBF_3OH (%) quantified over course of reaction between acid grade fluorspar (CaF_2) with H_2Ox and $B(OH)_3$ in D_2O at 50 °C.

Entry	Time (min)	HBF_4 (%)	HBF_3OH (%)
1	1	<1	1
2	5	16	6
3	15	22	10
4	120	44	12
5	180	96	3

5.3.2. Reaction monitoring in presence of SiO_2

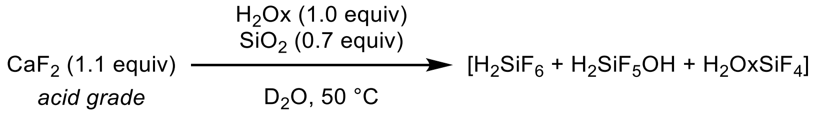

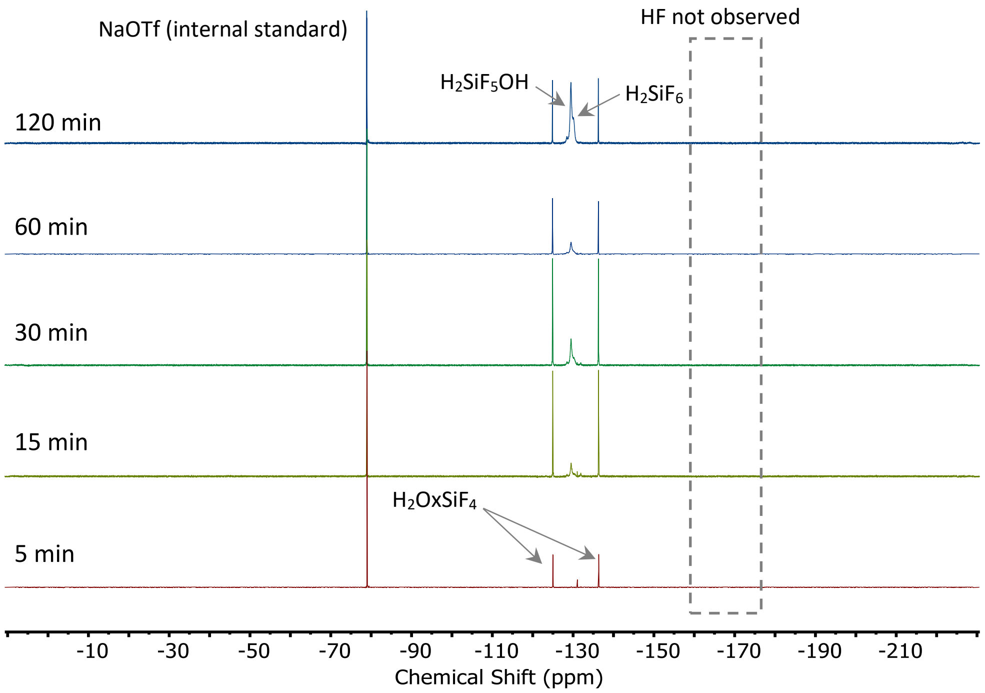

Figure S8. Reaction of acid grade fluorspar (CaF_2) with H_2Ox and SiO_2 in D_2O at 50 °C monitored by ^{19}F NMR (D_2O). H_2SiF_6 at -131.2 ppm, H_2SiF_5OH at -128.9 ppm (broadening of signals due to fast intramolecular fluoride exchange) and H_2OxSiF_4 resonances at -124.5 ppm and -135.9 ppm [21,22]. Sodium triflate standard at -78.0 ppm.

5.4. Reaction monitoring in absence of H_2Ox

Acid grade fluorspar (97% CaF_2, 40 mg, 0.5 mmol, 1.1 equiv), $B(OH)_3$ (14 mg, 0.23 mmol, 0.5 equiv) or SiO_2 (18 mg, 0.32 mmol, 0.7 equiv) was stirred in D_2O (0.5 mL) at 50 °C for 15 h. The suspension was transferred to a 1 mL centrifuge tube. Centrifugation with an acceleration time of 120 seconds (13,500 rpm) (included in the overall centrifugation time) at 25 °C followed and the supernatant was analyzed by quantitative ^{19}F NMR spectroscopy (D_2O). No signals were detected in the ^{19}F NMR spectra of the supernatant of either reaction mixture.

5.5. Characterization of aqueous boron fluorine products (HBF_4, HBF_3OH and $HOxBF_2$)

The reaction between acid grade fluorspar (CaF_2, > 97.0%, Minersa Group) with anhydrous oxalic acid and $B(OH)_3$ in D_2O was monitored at 50 °C. Monitoring of the reaction by ^{19}F NMR enabled the identification of HBF_4, HBF_3OH and $HOxBF_2$ in solution.

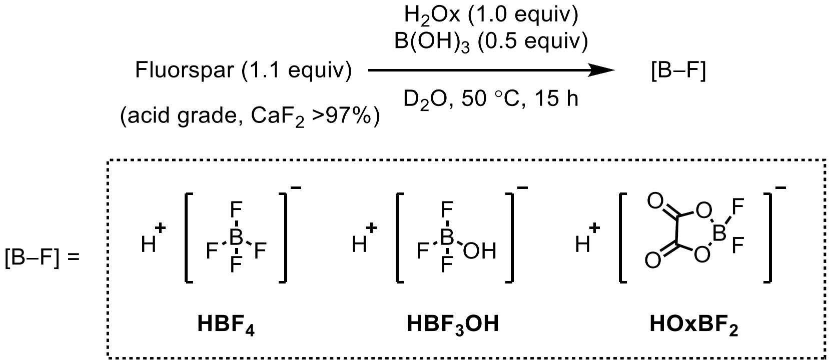

Characterization of the species was achieved using ^{11}B NMR (**Figure S9**) and ^{19}F NMR (**Figure S10**) analysis. All spectra were collected at 25 °C. Coupling between ^{19}F and ^{11}B leads to a quintet of peaks in the ^{11}B NMR spectrum for the BF_4 ion at -1.46 ppm, which cannot be resolved with the instrument, and a quartet for the HBF_3OH at -0.03 ppm.

HBF_4:
^{19}F NMR (377 MHz, D_2O) δ -150.3 ([^{10}B]HBF_4]), -150.3 ppm ([^{11}B]HBF_4], quartet, J = 1.1 Hz)
^{11}B NMR (128 MHz, D_2O) δ -1.46 (br s).

HBF_3OH:
^{19}F NMR (377 MHz, D_2O) δ -145.26 (overlapping br s and q, J = 10.7 Hz)
^{11}B NMR (128 MHz, D_2O) δ -0.03 (q, J = 10.7 Hz)

$HOxBF_2$:
^{19}F NMR (377 MHz, D_2O) δ -152.27 ([^{10}B]$HOxBF_2$], br s), -152.34 ([^{11}B]$HOxBF_2$], br s)
^{11}B NMR (D_2O) δ 2.84 (br s)

Data is in accordance with literature values [19]. The peak at -2.84 ppm in the ^{11}B NMR spectrum of the reaction mixture was assigned to a difluoro(oxalato)borate species ($HOxBF_2$) [20].

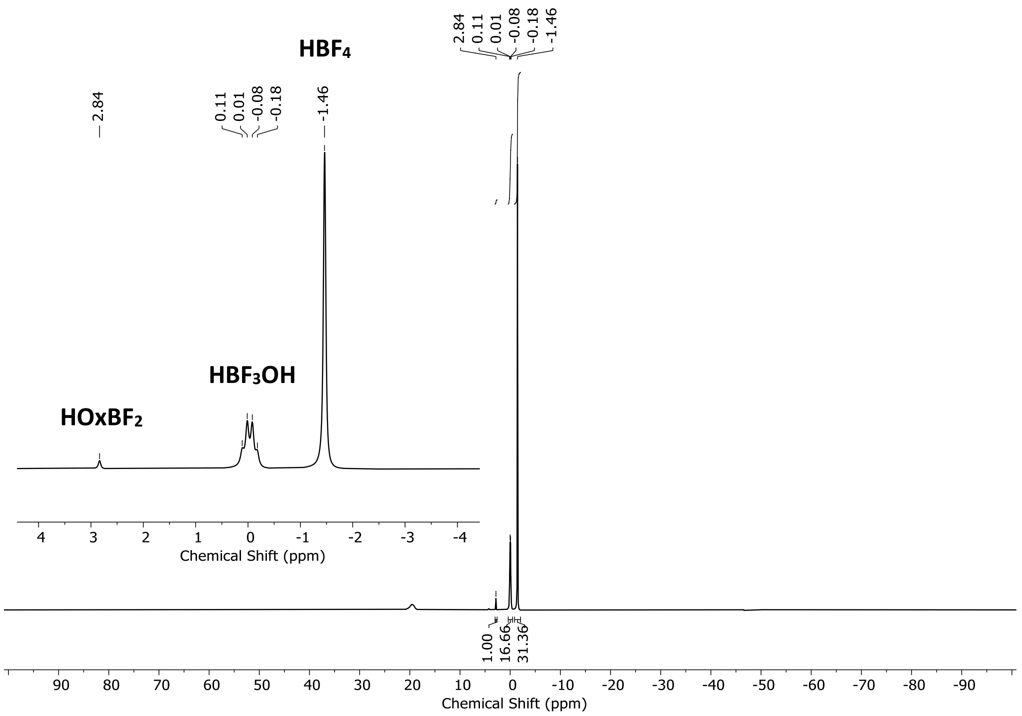

Figure S9. ^{11}B NMR (D_2O) of reaction between acid grade fluorspar (CaF_2), H_2Ox and $B(OH)_3$ at 50 °C (15 h). The species observed are HBF_4 at -1.46 ppm, HBF_3OH at -0.03 ppm and $HOxBF_2$ at 2.84 ppm.

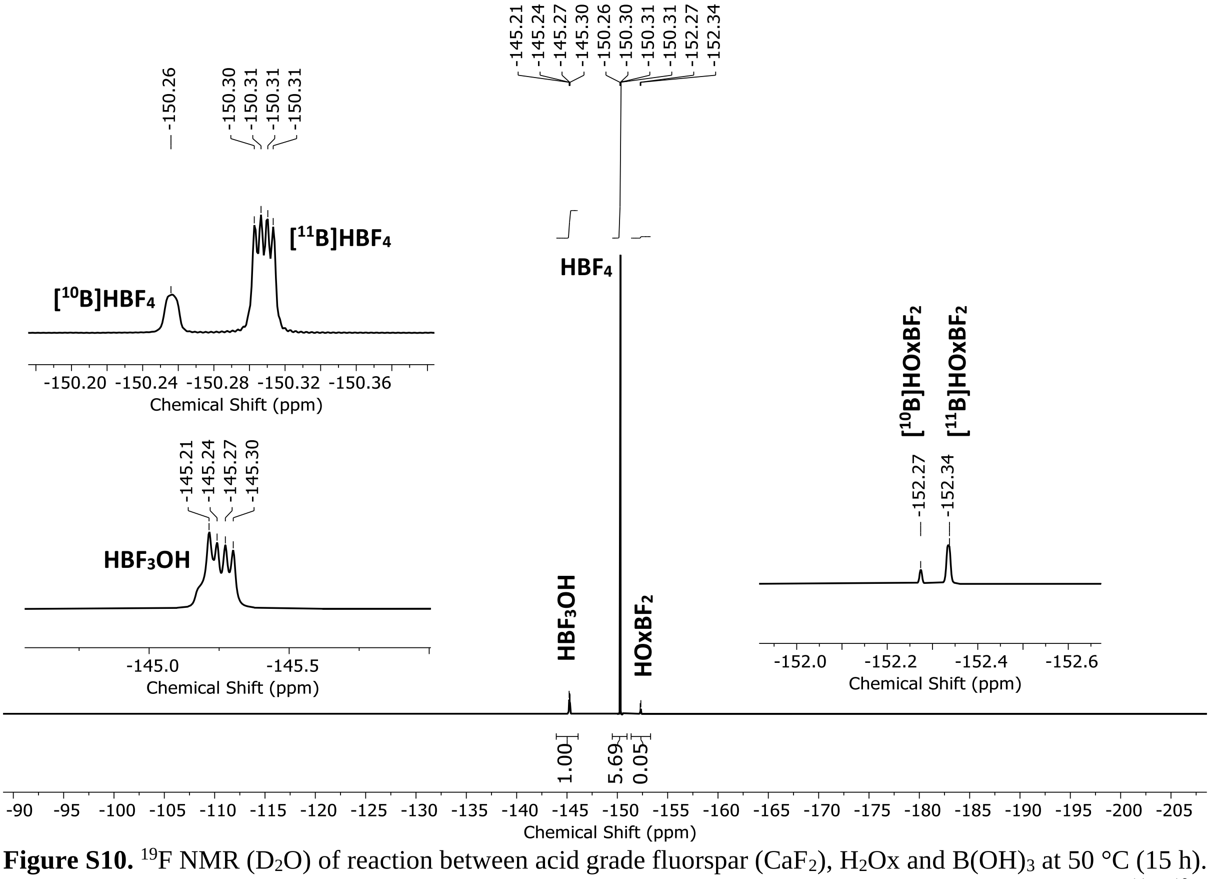

Figure S10. ^{19}F NMR (D_2O) of reaction between acid grade fluorspar (CaF_2), H_2Ox and $B(OH)_3$ at 50 °C (15 h). The species observed are HBF_4 at -150.3 ppm, HBF_3OH at -145.3 ppm and $HOxBF_2$ at -152.3 ppm. $^{11}B/^{10}B$ isotope effects observed.

5.6. Characterization of aqueous silicon fluorine products (H_2SiF_6, H_2SiF_5OH and H_2OxSiF_4)

The reaction between acid grade fluorspar (CaF_2, > 97.0%, Minersa Group) with anhydrous oxalic acid and SiO_2 in D_2O was monitored at 50 °C by ^{19}F NMR. All spectra were collected at 25 °C.

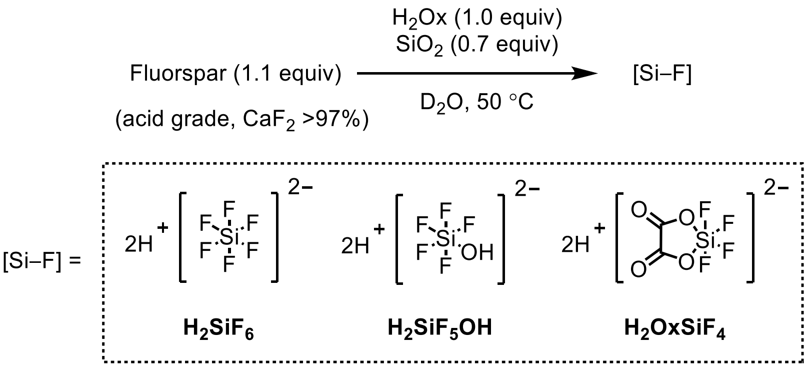

The formation of H_2SiF_6 was observed after 5 min by ^{19}F NMR, in addition to two fluorine resonances (triplets) at -124.9 ppm and -136.2 ppm with $J_{F\text{-}F}$ coupling values of 8.9 Hz (**Figure S12**). These resonances are assigned to the oxalate fluorosilicate species H_2OxSiF_4 [22]. $^{29}Si\{^{19}F\}$ NMR spectra was acquired using a ^{29}Si-^{19}F rINEPT NMR method [48,49]. $^{29}Si\{^{19}F\}$ NMR spectra of the reaction mixture after 5 min displayed 2 signals at -178.2 ppm and -182.8 ppm (**Figure S14**). The ^{19}F-^{29}Si bond of H_2SiF_6 and H_2OxSiF_4 was confirmed by a ^{19}F-^{29}Si HMBC experiment optimized for a short-range coupling (**Figure S15**). The ^{19}F to ^{29}Si rINEPT experiment was used to confirm *J* couplings (**Figure S13**).

H_2SiF_6:
^{19}F NMR (470 MHz, D_2O) δ -129.6 (br s).
^{29}Si{^{19}F} NMR (100 MHz, D_2O) δ -178.2 (s).

H_2OxSiF_4:
^{19}F NMR (470 MHz, D_2O) δ -124.9 ppm (t, $J_{F\text{-}F}$ = 8.9 Hz), -136.2 ppm (t, $J_{F\text{-}F}$ = 8.9 Hz).
^{29}Si {^{19}F} NMR (100 MHz, D_2O) δ -182.8 (s).

After 15 h, a second broad singlet is observed in the ^{19}F NMR spectrum of the reaction mixture at -128.9 ppm which is assigned to the hydrolysis intermediate H_2SiF_5OH [21] (**Figure S16**). The broad singlet at -129.6 ppm is assigned to H_2SiF_6.

H_2SiF_5OH:
^{19}F NMR (470 MHz, D_2O) δ -128.9 (br s)

Peaks corresponding to H_2SiF_6 and H_2SiF_5OH are broad and overlap due to fast fluoride exchange. Basic hydrolysis of the [Si-F] species using KOH (6 equiv) affords an aqueous solution of KF as the sole aqueous fluorine containing species, evidenced by ^{19}F NMR (see **Section 8** in **Supplementary Information**).

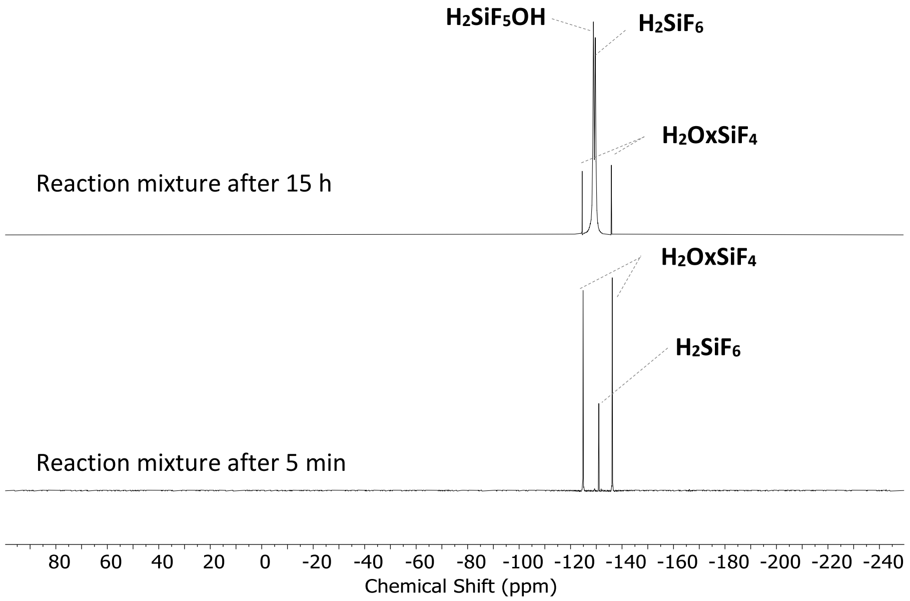

Figure S11. ^{19}F NMR (D_2O) of reaction between acid grade fluorspar (CaF_2), H_2Ox and SiO_2 at 50 °C. Species observed are H_2SiF_6, $H_2SiF_5(OH)$ and H_2OxSiF_4. Reaction after 5 min (bottom); reaction after 15 h (top).

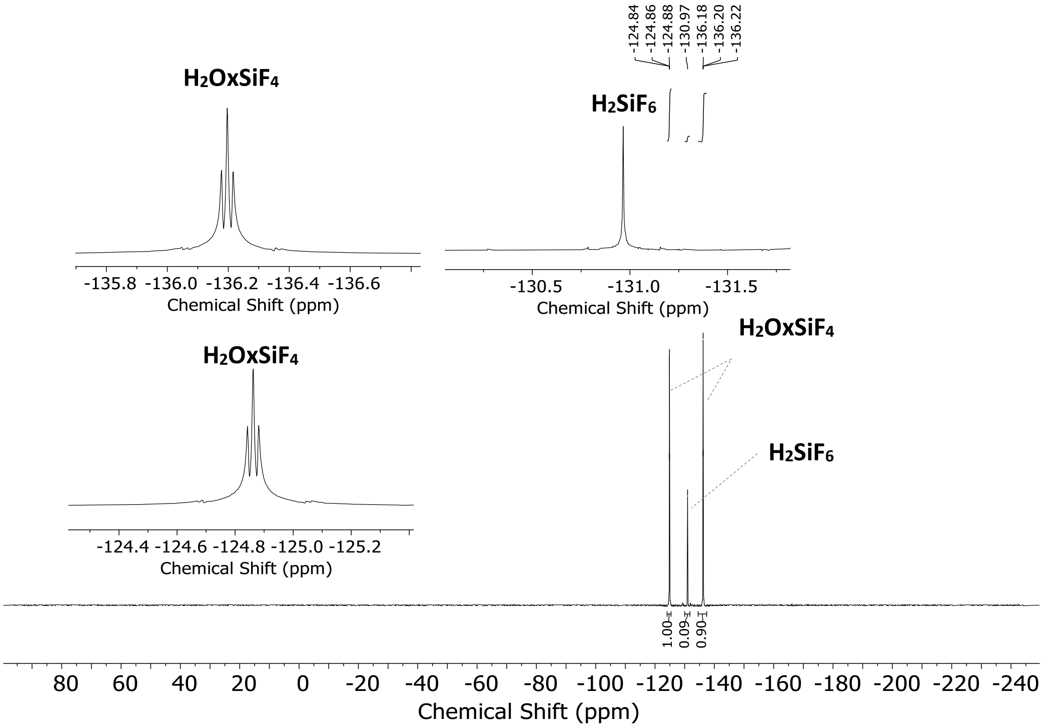

Figure S12. ^{19}F NMR (D_2O) of reaction between acid grade fluorspar (CaF_2), H_2Ox and SiO_2 (5 min, 50 °C). Aqueous species H_2SiF_6 (s, -131.2 ppm) and H_2OxSiF_4 (triplets at -124.9 ppm and -136.2 ppm with $^2J_{F\text{-}F}$ = 8.9 Hz) observed.

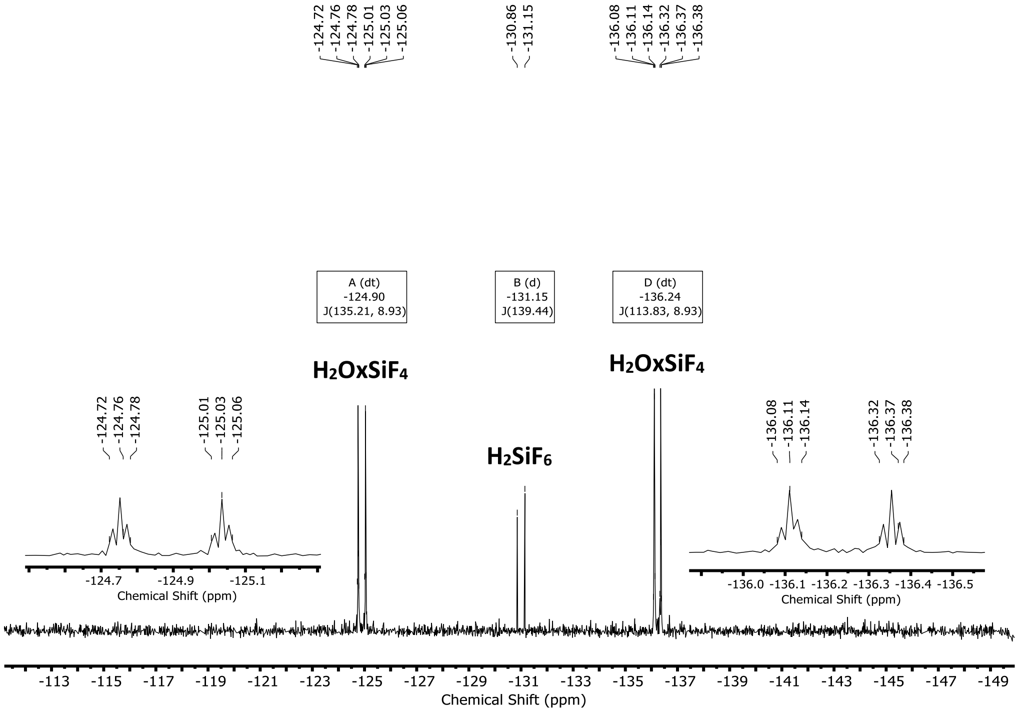

Figure S13. ^{19}F NMR (D_2O) of reaction between acid grade fluorspar (CaF_2), H_2Ox and SiO_2 (5 min, 50 °C), acquired using ^{29}Si-^{19}F rINEPT NMR method. Aqueous species H_2SiF_6 (d, -131.2 ppm with $^1J_{Si\text{-}F}$ = 139.4 Hz) and H_2OxSiF_4 (two dt at -124.9 ppm with $^1J_{Si\text{-}F}$ = 135.2 Hz and $^2J_{F\text{-}F}$ = 8.9 Hz and at -136.2 ppm with $^1J_{Si\text{-}F}$ = 113.8 Hz and $^2J_{F\text{-}F}$ = 8.9 Hz) were observed.

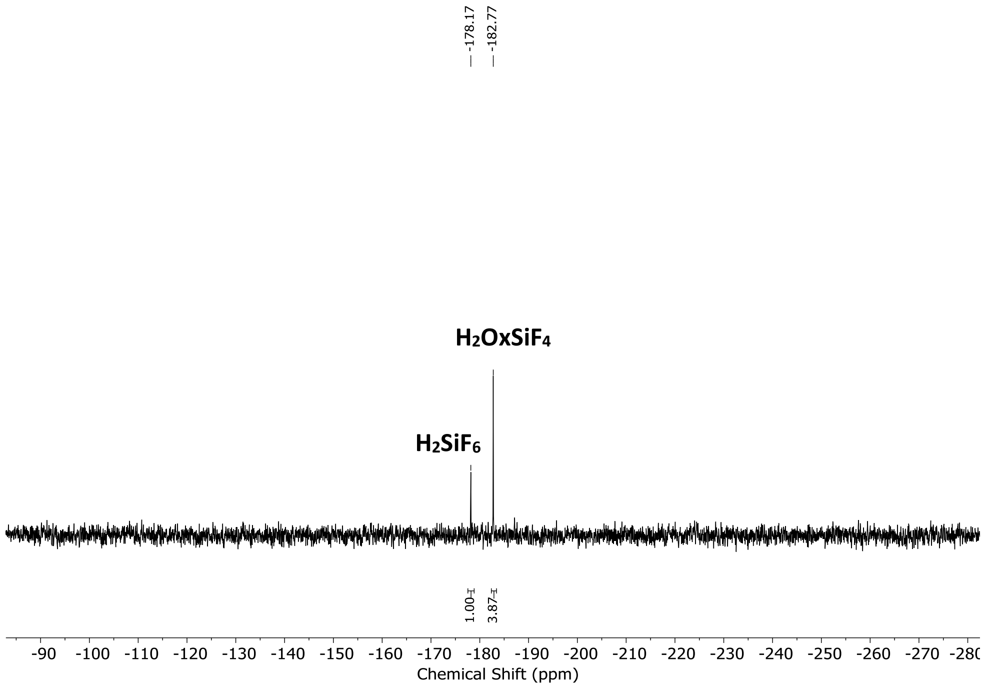

Figure S14. $^{29}Si\{^{19}F\}$ NMR (D_2O) of reaction between acid grade fluorspar (CaF_2), H_2Ox and SiO_2 (5 min, 50 °C). Aqueous species H_2SiF_6 (-178.2 ppm) and H_2OxSiF_4 (-182.8 ppm) observed. $^{29}Si\{^{19}F\}$ NMR spectra was acquired using ^{29}Si-^{19}F rINEPT NMR method.

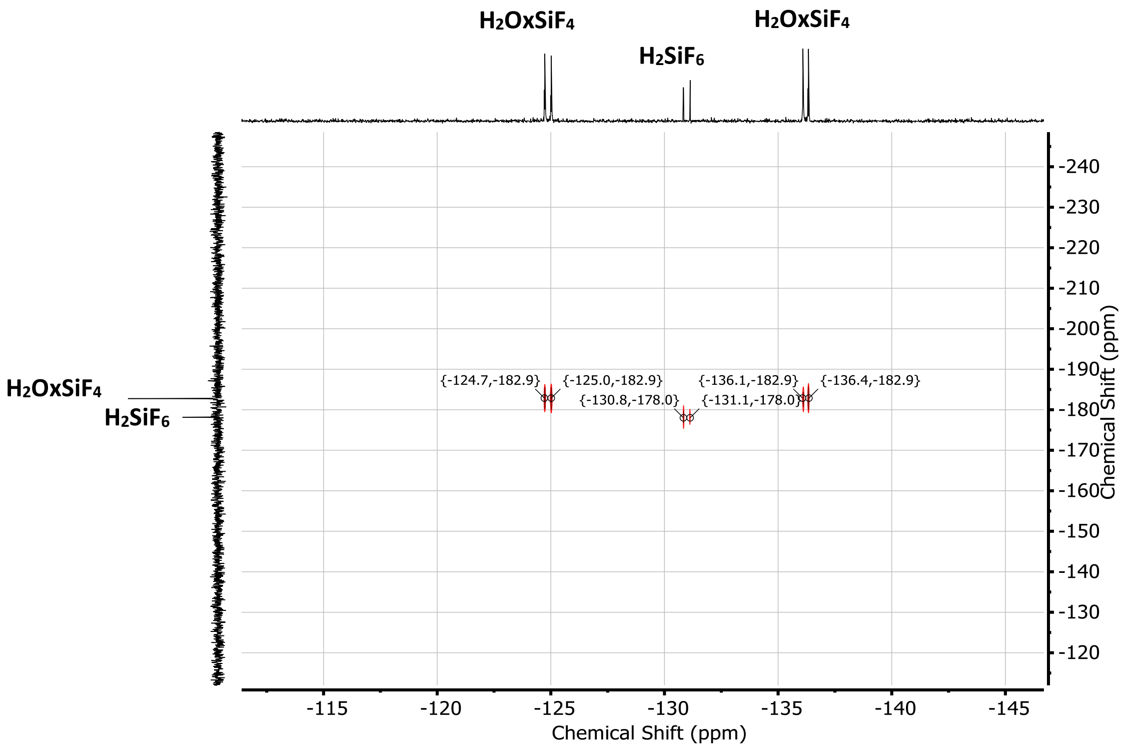

Figure S15. ^{29}Si-^{19}F HMBC NMR experiment (D_2O) of reaction between acid grade fluorspar (CaF_2), H_2Ox and SiO_2 (5 min, 50 °C) confirming ^{19}F-^{29}Si bonds and $^{1}J_{Si-F}$ couplings. The top trace is ^{19}F NMR spectrum (internal projection). Left trace is $^{29}Si\{^{19}F\}$ NMR spectrum (external projection).

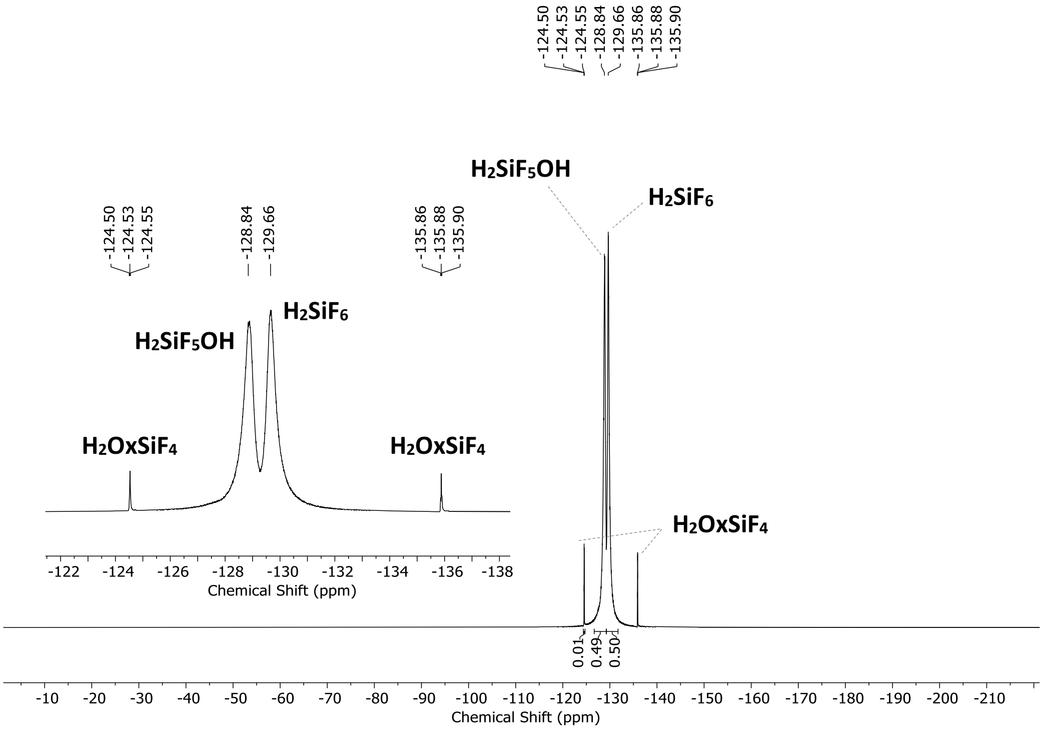

Figure S16. ^{19}F NMR (D_2O) of reaction between acid grade fluorspar (CaF_2), H_2Ox and SiO_2 (15 h, 50 °C). Aqueous species H_2SiF_6 (br s, -129.6 ppm), H_2SiF_5OH (br s, -128.8 ppm) and H_2OxSiF_4 (two triplets at -124.5 ppm and -135.9 ppm with $^{2}J_{F-F}$ = 8.9 Hz) observed.

5.7. Characterization of insoluble by-product formed in the reaction of acid grade fluorspar with H_2Ox and Lewis acid in water

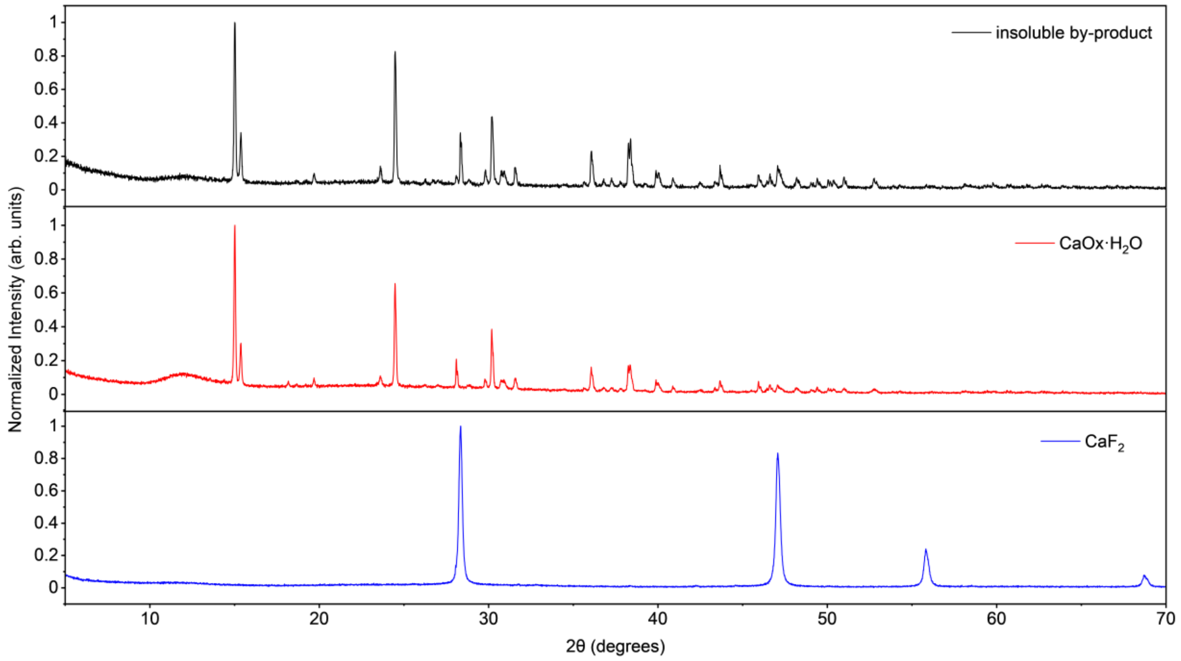

Figure S17. Powder X-ray diffraction patterns of insoluble by-product formed in the reaction of acid grade fluorspar (CaF_2) with H_2Ox and $B(OH)_3$ after 15 h at 50 °C (top), commercial $CaOx \cdot H_2O$ (middle) and acid grade fluorspar (CaF_2) (bottom). All powder X-ray diffraction patterns recorded at room temperature.

6. Reaction Optimization

6.1. Optimization of HBF_4 preparation

Acid grade fluorspar (1.0 to 1.1 equiv), boric acid (0.5 g, 8.08 mmol, 0.5 equiv), water (4 mL) and anhydrous oxalic acid (1.46 g, 16.2 mmol, 1.0 equiv) were weighed into a 50 mL conical sterile polypropylene (PP) centrifuge tube. Each reaction was stirred at corresponding temperature for 16 h. Then, NaOTf (348 mg, 2.02 mmol) was added as an internal standard. The resulting crude mixture was then diluted with 2 mL of water and stirred for an additional 2 min to ensure the complete dissolution of NaOTf. The suspension was allowed to settle and the total amount of HBF_4 and HBF_3OH was assessed by quantitative ^{19}F NMR spectroscopy (D_2O).

This procedure was optimized for temperature, reaction time, concentration and equivalents of reagents.

6.1.1. Ratios of reagents

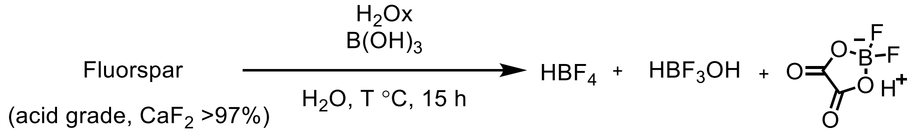

Table S9. Optimization of reagent ratio for B-F product formation.

Entry	fluorspar : H_2Ox : $B(OH)_3$	temp. (° C)	H_2Ox concentration (M)	HBF_4 (%)	HBF_3OH (%)	$HOxBF_2$ (%)
1	1.1 : 1.0 : 0.5	50	4	81	16	<1
2	1.0 : 1.0 : 0.5	50	4	71	16	<1

6.1.2. Reaction temperature

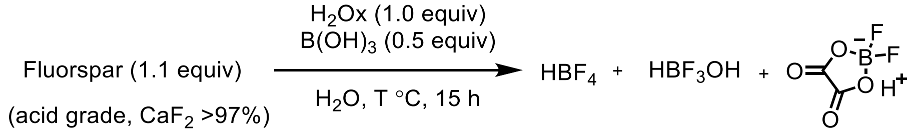

Table S10. Optimization of reaction temperature for B-F product formation.

Entry	temp. (° C)	H_2Ox concentration (M)	HBF_4 (%)	HBF_3OH (%)	$HOxBF_2$ (%)
1	40	4	72	16	<1%
2	50	4	81	16	<1%
3	25	4	51	16	<1%
4	70	4	81	16	<1%

6.1.3. Reaction concentration

The effect of concentration (respect to oxalic acid) in the activation of acid grade fluorspar using oxalic acid and $B(OH)_3$ was studied.

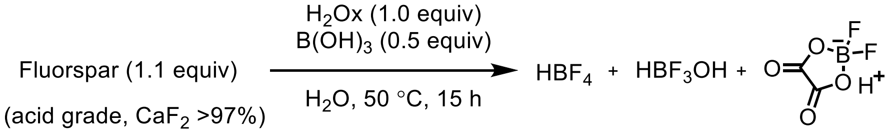

Table S11. Optimization of reaction concentration for B-F product formation.

Entry	H_2Ox concentration (M)	HBF_4 (%)	HBF_3OH (%)	$HOxBF_2$ (%)
1	4	81	16	<1%
2	5.4	81	16	<1%
3	3	81	16	<1%

6.1.4. Use of oxalic acid dihydrate

Oxalic acid dihydrate is a cheaper alternative to anhydrous oxalic acid. Anhydrous oxalic acid was replaced for oxalic acid dihydrate.

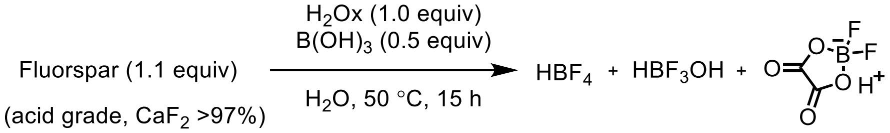

Table S12. Replacement of anhydrous oxalic acid for oxalic acid dihydrate for B-F product formation.

Entry	Oxalic acid	HBF_4	HBF_3OH	$HOxBF_2$ (%)
1	anhydrous	81	16	<1%
2	dihydrate	82	16	<1%

High HBF_4 yield (82%) was retained when anhydrous oxalic acid was replaced for oxalic acid dihydrate.

6.2. Optimization of KF preparation

Activation of acid grade fluorspar (> 97% CaF_2) using anhydrous oxalic acid (H_2Ox) and silica gel (SiO_2) was investigated. Filtration of the suspension followed by basification with KOH affords KF.

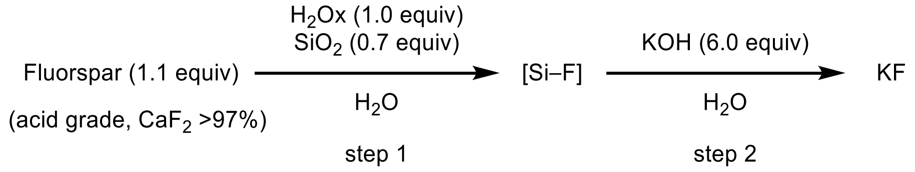

Step 1 - Cooperative activation of fluorspar

Acid grade fluorspar (644.0 mg, 8.0 mmol, 1.1 equiv), silica gel (306.0 mg, 5.1 mmol, 0.7 equiv) and oxalic acid (655 mg, 7.3 mmol, 1.0 equiv) were weighed into a 50 mL conical sterile polypropylene (PP) centrifuge tube. H_2O (2.5 mL) was added and the mixture was heated with stirring at the indicated temperature for the indicated reaction time. The resulting suspension was cooled to ambient temperature, diluted with H_2O (5 mL) and filtered using a Büchner funnel into a 50 mL PP tube. Filtered solids were washed with H_2O (2.5 mL) to give an aqueous solution (~ 10 mL) containing H_2SiF_6, H_2SiF_5OH and $OxSiF_4$.

Step 2 - Basic hydrolysis of Si–F species

The first step of this reaction affords a theoretical maximum of 2.4 mmol of H_2SiF_6. Accordingly, KOH (85%, 951 mg, 14.4 mmol) was added portion-wise to the aqueous solution (containing H_2SiF_6, H_2SiF_5OH and $OxSiF_4$). The reaction was heated with stirring at the indicated temperature for the indicated reaction time. The resulting suspension was filtered using a fritted glass filter to separate insoluble by-products from the solution and washed with water (2 x 5 mL). The filtrate was concentrated *in vacuo* (50 °C) and dried under high vacuum (< 0.1 mbar). Gentle heating (100 °C) of the flask under vacuum for 5 min followed by drying overnight under high vacuum provided a white solid product.

KF analysis

To assess the quantity of KF in the solid product, a sample of the solid product (14 mg) and NaOTf as an internal standard was dissolved in D_2O. The purity of KF in the solid was assessed by quantitative ^{19}F NMR as described in **Section 8.8.1** in **Supplementary Information**[46].

This 2-step procedure was optimized for temperature, reaction time, concentration and equivalents of SiO_2. The KF yield (%) was calculated for each reaction using equation (1).

$$\text{purity of KF} \times \text{mass of solid product} = \text{KF yield (\%)} \quad (1)$$

6.2.1. Reaction temperature

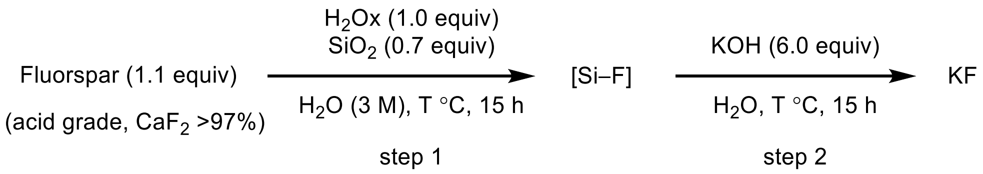

Table S13. Optimization of reaction temperature for KF preparation.

Entry	step 1 T (° C)	step 2 T (° C)	product (KF) mass	KF purity	KF yield
1	25	50	98	24	24
2	25	70	99	33	33
3	50	25	87	46	40
4	50	50	87	81	71
5	50	70	99	87	86
6	70	25	94	28	26
7	70	50	93	77	72
8	70	70	96	76	73

Reaction temperatures of 50 °C for step 1 and 70 °C for step 2 proved to be optimal for KF yield (86%).

6.2.2. Reaction time

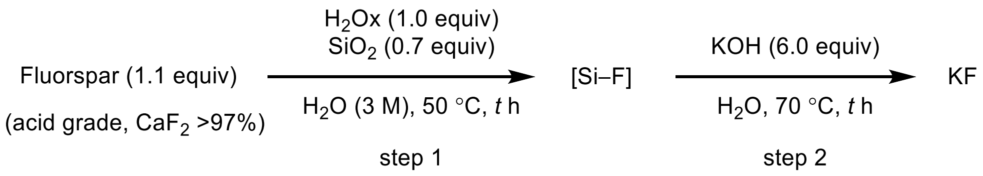

Table S14. Optimization of reaction time for KF preparation.

Entry	step 1 time (h)	step 2 time (h)	product (KF) mass	KF purity	KF yield
1	1	1	100	54	54
2	1	15	100	46	45
3	3	1	99	53	52
4	3	15	99	38	38
5	15	1	97	81	80
6	15	3	96	92	89
7	15	15	99	87	86

Reaction times of 15 h for step 1 and 1 h (or above) for step 2 proved to be optimal for KF yield (up to 89%).

6.2.3. Reaction concentration

The effect of concentration (respect to oxalic acid) in step 1 (activation of acid grade fluorspar using oxalic acid and SiO_2) was studied.

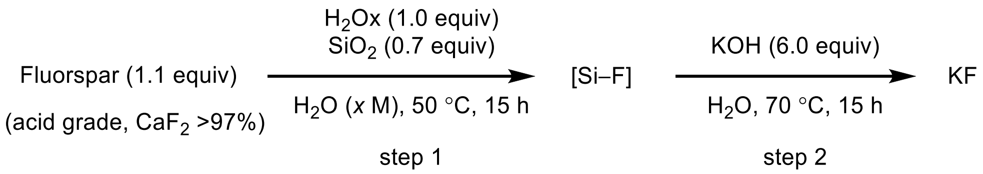

Table S15. Optimization of reaction concentration for KF preparation.

Entry	H_2Ox concentration (M)	Step 1 H_2O volume (mL)	product (KF) mass	KF purity (%)	KF yield (%)
1	0.5	14.5	95	96	91
2	2.0	3.7	92	81	74
3	3.0	2.4	99	87	86
4	4.0	1.8	97	83	80

Oxalic acid concentration of 0.5 M was found be optimal for KF yield (91%). These conditions also ensure complete dissolution of oxalic acid in water.

6.2.4. Equivalents of SiO_2

The effect of reducing SiO_2 equivalents (respect to oxalic acid) in step 1 was studied.

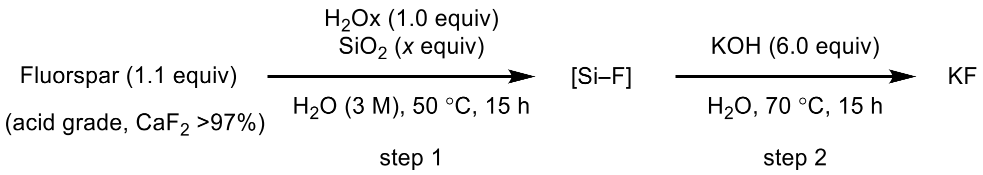

Table S16. Optimization of SiO_2 equivalents for KF preparation.

Entry	equivalents of SiO_2	product (KF) mass	KF purity (%)	KF yield (%)
1	0.7	99	87	86
2	0.4	98	84	82
3	0.3	98	97	95

High yields of KF (purity > 97%) were retained when the equivalents of SiO_2 was lowered to 0.3 equivalents. We chose to use 0.4 equivalents of SiO_2 for our final conditions.

6.2.5. Use of oxalic acid dihydrate

Oxalic acid dihydrate is a cheaper alternative to anhydrous oxalic acid. Anhydrous oxalic acid was replaced for oxalic acid dihydrate in step 1.

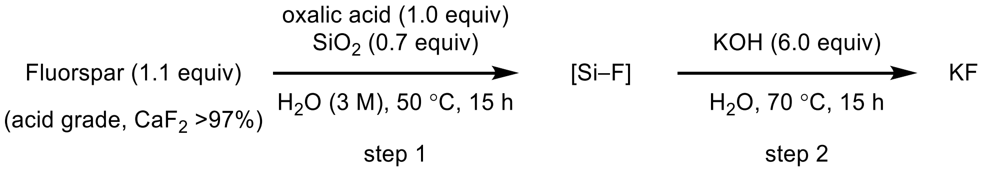

Table S17. Replacement of anhydrous oxalic acid for oxalic acid dihydrate for KF preparation.

Entry	Oxalic acid	product (KF) mass	KF purity	KF yield
1	anhydrous	99	87	86
2	dihydrate	98	87	85

High KF yield (85%) was retained when anhydrous oxalic acid was replaced for oxalic acid dihydrate in step 1.

6.2.6. Reassessment of acids for fluorspar activation using SiO_2 under optimized conditions

The total fluoride release from acid grade fluorspar (>97%, CaF_2) using SiO_2 under optimized conditions was reinvestigated with Brønsted acids aq. HCl (37%), aq. H_2SO_4 (95%-98%) and oxalic acid dihydrate.

Acid grade fluorspar (644.0 mg, 8.0 mmol, 1.1 equiv), silica gel (175.0 mg, 2.9 mmol, 0.4 equiv), H_2O (14.5 mL) and Brønsted acid activator (1.0 equiv for "diacids", 2.0 equiv for "monoacid") were added into a 50 mL conical sterile polypropylene (PP) centrifuge tube. The reaction mixture was heated with stirring at the 50 °C for 15 h. The resulting suspension was cooled to ambient temperature and filtered with a Büchner funnel into a 50 mL PP tube. Filtered solids were washed with H_2O (5 mL) to give an aqueous solution (~ 20 mL) containing H_2SiF_6, H_2SiF_5OH and $OxSiF_4$.

The first step of this reaction affords a theoretical maximum of 2.4 mmol of H_2SiF_6. Accordingly, KOH (85%, 808 mg, 14.4 mmol) was added portion-wise to the aqueous solution (containing H_2SiF_6, H_2SiF_5OH and $OxSiF_4$). The reaction mixture was heated with stirring at the 50 °C for 15 h. NaOTf (86 mg, 0.5 mmol) was added as an internal standard to the reaction mixture. The reaction mixture was stirred for 2 minutes and an aliquot of the reaction was analyzed by quantitative ^{19}F NMR spectroscopy (D_2O). The total amount of fluoride in solution was quantified.

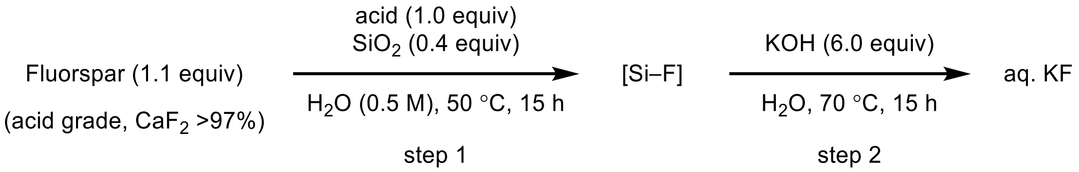

Table S18. Reassessment of Brønsted acids for activation of acid grade fluorspar in the presence of SiO_2 under optimized conditions.

Entry	Brønsted acid	aq. KF (%)
1	$H_2Ox\cdot 2H_2O$	96
2	HCl	0
3	H_2SO_4	51

6.2.7. Quantification of [Si-F] species under optimized conditions ($H_2Ox{\cdot}H_2O$ as Brønsted acid)

Acid grade fluorspar (644.0 mg, 8.0 mmol, 1.1 equiv), silica gel (175.0 mg, 2.9 mmol, 0.4 equiv), H_2O (14.5 mL) and oxalic acid dihydrate (917 mg, 7.3 mmol, 1 equiv) were added into a 50 mL conical sterile polypropylene (PP) centrifuge tube. The reaction mixture was heated with stirring at the 50 °C for 15 h. The reaction mixture was heated with stirring at 50 °C for 15 h. NaOTf (45 mg, 0.262 mmol) was added as an internal standard to the reaction mixture. The reaction mixture was stirred for 2 minutes and an aliquot of the reaction was analyzed by quantitative ^{19}F NMR spectroscopy (D_2O) (32 scans, d1 = 30 s, op1 = 103.5 ppm).

The total amount of [Si-F] species was quantified using the global spectrum deconvolution (GSD) tool available on MestReNova which enables integration of partially overlapping peaks [47].

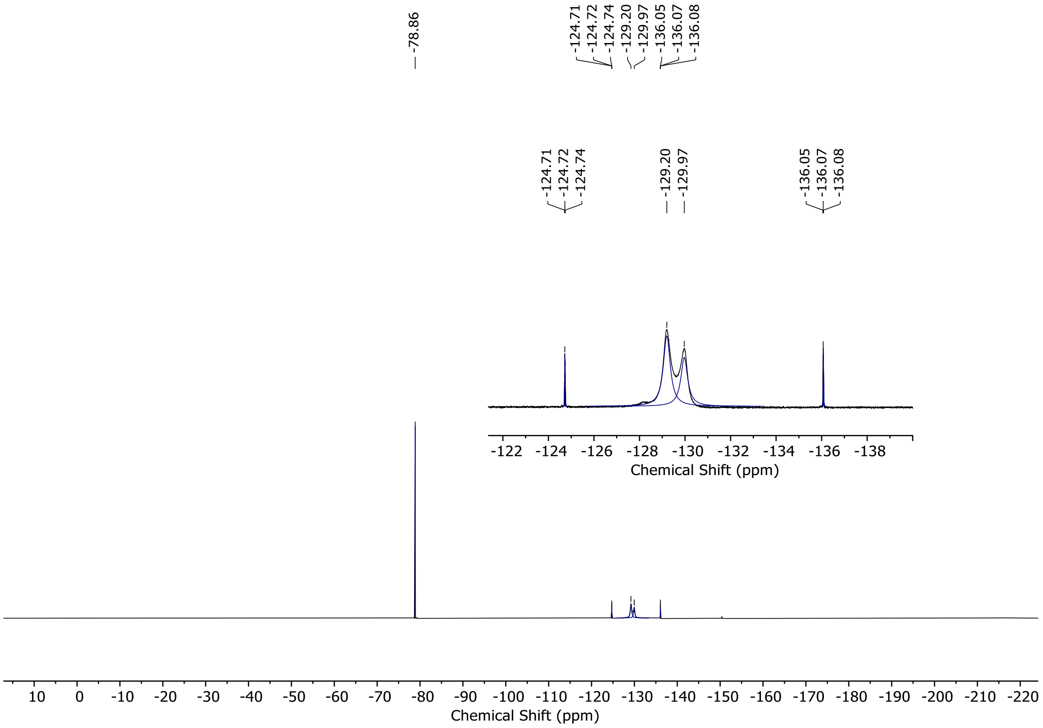

Figure S18. ^{19}F NMR (D_2O) spectrum of reaction between acid grade fluorspar (CaF_2), H_2Ox and SiO_2 (15 h, 50 °C). Aqueous species H_2SiF_5OH (br s, -129.2 ppm), H_2SiF_6 (br s, -130.0 ppm) and H_2OxSiF_4 (two triplets at -124.7 ppm and -136.1 ppm with $^2J_{F\text{-}F}$ = 8.9 Hz) observed.

	ppm	Intensity	Width	Area
1	-78.86	3737.7	1.18	7717.36
2	-78.87	13.6	1.33	32.71
3	-129.20	260.7	170.66	80429.78
4	-129.97	181.4	166.94	55439.13
5	-136.05	77.3	3.07	393.22
6	-136.07	194.3	3.16	1202.51
7	-136.08	138.0	6.76	1872.93

Figure S19. Global Spectrum Deconvolution (GSD) peak list characterized by chemical shift (ppm), peak intensity, peak width and peak area. Data extracted from the ^{19}F NMR spectrum in **Figure S18**.

Table S19. Calculated [Si–F] % values from total peak area values obtained using GSD

Entry	Compound	Total peak area	Normalized area	[Si-F] %
1	NaOTf	7717.36	1	-
2	H_2SiF_6	55439.14	7.18	39
3	$H_2SiF_5(OH)$	80429.78	10.42	56
4	H_2OxSiF_4	3468.66	0.45	2
			Total [Si-F]	97

7. Characterization of K_2SiF_6 from acid grade fluorspar

Treatment of the aqueous solution of H_2SiF_6, H_2SiF_5OH and H_2OxSiF_4 with 2 equivalents of KOH at 25 °C affords K_2SiF_6.

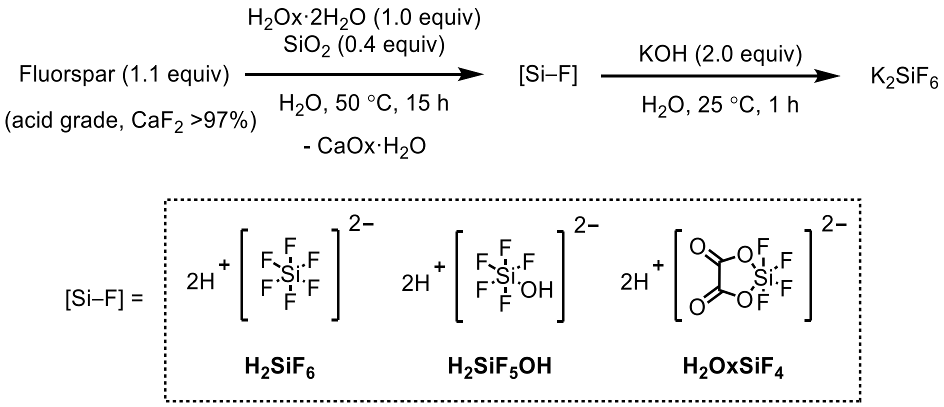

Oxalic acid dihydrate (9.17 g, 72.7 mmol, 1.0 equiv) was dissolved in H_2O (145.6 mL) in a 500 mL PTFE round bottom flask. A mixture of acid grade fluorspar (97% CaF_2, 6.44 g, 80.0 mmol, 1.1 equiv) and silica gel (1.75 g, 29.0 mmol, 0.4 equiv) was added in 3 portions to the solution of oxalic acid dihydrate at 50 °C. The resulting suspension was stirred using a mechanical stirrer at 50 °C. After 15 h, the suspension was cooled to room temperature, and filtered using a Büchner funnel into a 500 mL PTFE round bottom flask. Filtered solids were washed with H_2O (3 x 5 mL) to give a ~160 mL aqueous solution containing H_2SiF_6, H_2SiF_5OH and $OxSiF_4$. The first step of this reaction affords a theoretical maximum of 24 mmol of H_2SiF_6. Accordingly, KOH (85%, 3.17 g, 48 mmol) was added to the filtrate (until pH 7 was reached). The suspension was stirred at 25 °C for 1 h before filtration. The filtered solid was allowed to dry at room temperature and then under high vacuum to give K_2SiF_6 as a white powder (5.08 g, 23 mmol, 96%).

K_2SiF_6 is sparingly soluble in water. The formation of K_2SiF_6 was confirmed by PXRD.

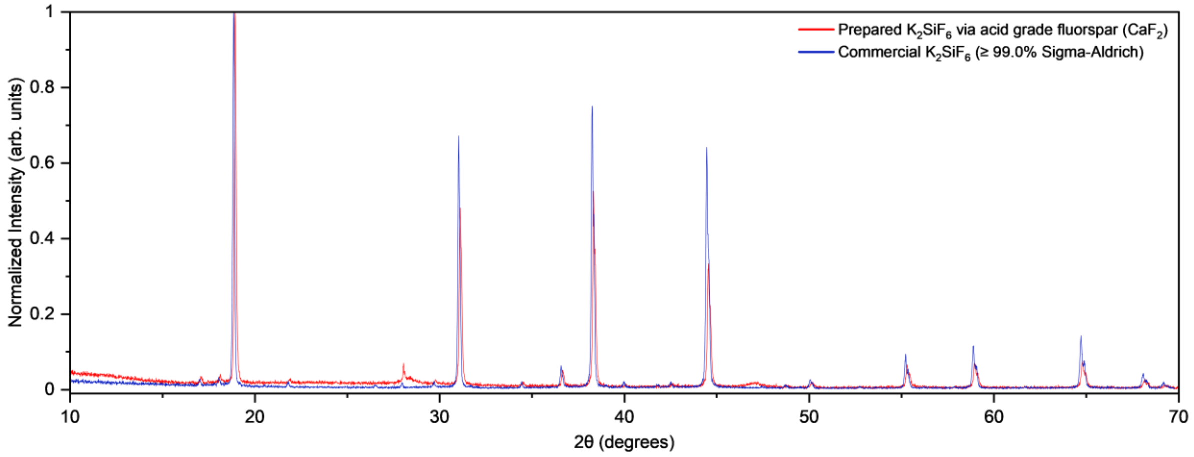

Figure S20. Powder X-ray diffraction patterns of K_2SiF_6 prepared via acid grade fluorspar (red) and commercial K_2SiF_6 (blue) collected at room temperature.

Treatment of the aqueous solution of H_2SiF_6, H_2SiF_5OH and H_2OxSiF_4 with 6 equivalents of KOH at 70 °C affords KF (see **Section 8.8** of **Supplementary Materials**).

8. Synthesis of Fluorinating Reagents

Detailed procedures to prepare common fluorinating reagents (HBF_4, KF, Me_4NF and *n*Bu_4NF reagents) from acid grade fluorspar (CaF_2 >97%) or metspar (CaF_2 ~ 85%) using our optimized reaction conditions are outlined below.

8.1. Preparation of HBF_4 from acid grade fluorspar

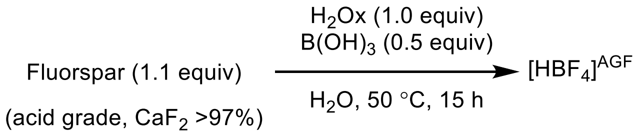

Acid grade fluorspar (> 97% CaF_2, 27.8 g, 355.8 mmol, 1.1 equiv), boric acid (10 g, 161.7 mmol, 0.5 equiv) and oxalic acid dihydrate (40.78 g, 323.5 mmol, 1equiv) were weighed into a 250 mL PTFE round bottom flask. A stir bar and water (60 mL) were added and the mixture was heated in a preheated oil bath to 50 °C for 15 h. After cooling the resulting suspension to room temperature, the solids were filtered off using a Büchner funnel and further washed with water (2 x 5 mL) to obtain a solution mainly containing tetrafluoroborate anion along with a minor amount of oxalic acid. Upon concentration to ~5.5-6.5 M oxalic acid precipitated out of solution. The aqueous solution was decanted to give $[HBF_4]^{AGF}$. Aliquot analysis by ^{19}F NMR using NaOTf as internal standard revealed a yield of 75% HBF_4 and 16% HBF_3OH mixture. The concentration of HBF_4 was determined as ~5.5-6.5 M. The aqueous solution of $[HBF_4]^{AGF}$ was used for Balz-Schiemann reactions without further purification.

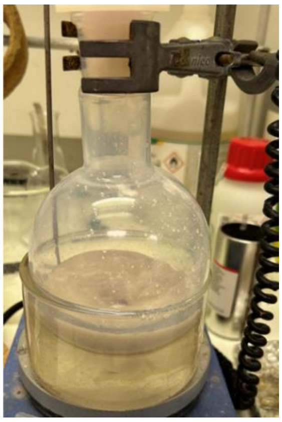

Figure S21. Large scale reaction setup employed in the preparation of $[HBF_4]^{AGF}$

8.2. Preparation of HBF_4 from metspar I (85% CaF_2)

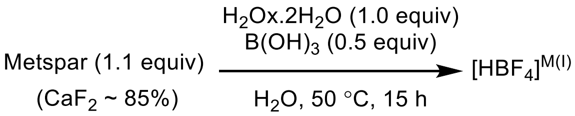

Metspar I sourced from Luoyang Aurora Minechem Co., Ltd. (China) has the following purity profile CaF_2 (85%), SiO_2 (10%), $CaCO_3$ (<5%), S (0.12%), P (0.1%).

Powdered metspar (~85 % CaF_2, 9.8 g, 106.74 mmol, 1.1 equiv), boric acid (3.0 g, 48.52 mmol, 0.5 equiv) and oxalic acid dihydrate (12.23 g, 97.04 mmol, 1 equiv) were weighed into a 100 mL PTFE round bottom flask. A stir bar and water (18 mL) were added and the mixture was heated in a preheated oil bath to 50 °C for 15 h. After cooling the resulting suspension to room temperature, the solids were filtered off using a Büchner funnel and further washed with water (2 x 2 mL) to obtain a solution mainly containing tetrafluoroborate anion along with a minor amount of oxalic acid. Upon concentration to ~5.5-6.5 M oxalic acid precipitated out of solution. The aqueous solution was decanted to give $[HBF_4]^{M(I)}$. Aliquot analysis by ^{19}F NMR using NaOTf as internal standard revealed a yield of 66 % HBF_4 and 17% HBF_3OH mixture. The concentration of HBF_4 was determined as ~5.5-6.5 M. The aqueous solution of $[HBF_4]^{M(I)}$ was used for Balz-Schiemann reactions without further purification.

8.3. Preparation of HBF_4 from metspar II (89% CaF_2)

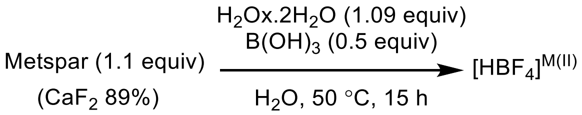

Metspar II was sourced from Mexico (Mexichem Fluor S.A de C.V.) **Metspar^{II}** with the following purity profile: CaF_2 (88.98%), SiO_2 (5.43%), $CaCO_3$ (4.02%), Al_2O_3 (0.41%), Fe_2O_3 (0.24%), S (0.011%), P (0.023%), Pb (<0.001%).

Excess of $H_2Ox{\cdot}2H_2O$ was added to the reaction to neutralize 4% $CaCO_3$ found in metspar II.

Powdered metspar (89% CaF_2, 1.56 g, 17.8 mmol, 1.1 equiv), boric acid (0.50 g, 8.09 mmol, 0.5 equiv), water (4 mL) and oxalic acid dihydrate (2.22 g, 17.6 mmol, 1.09 equiv) were weighed into a 50 mL conical sterile polypropylene (PP) centrifuge tube. The reaction was stirred at 50 °C for 15h. NaOTf (347.8 mg, 2.02 mmol) was added as an internal standard and the crude mixture was diluted with 2 mL of water and stirred for an additional 2 min to ensure the complete dissolution of NaOTf. Aliquot analysis by ^{19}F NMR using NaOTf as internal standard revealed a yield of 67 % HBF_4 and 16% HBF_3OH mixture.

8.4. Performance of prepared HBF_4

The performance of the HBF_4, prepared using either acid grade fluorspar ($[HBF_4]^{AGF}$) or metspar ($[HBF_4]^{M(I)}$) was compared with commercial HBF_4 ($[HBF_4]^{C}$, 48 wt. % in H_2O, Sigma Aldrich) in the Balz-Schiemann reaction of 4-bromoaniline.

In a 25 mL round-bottom flask, 4-bromoaniline (5 mmol, 1.0 equiv) was suspended in 5 mL of ethanol. Subsequently, an aqueous solution containing $[HBF_4]$ (1.1 to 2.0 equiv) was added and the mixture was stirred for 5 minutes at 25 °C. Next, the solution was cooled to 0 °C using an ice bath, and ^{t}BuONO (1.19 mL, 10 mmol, 2 equiv) was added dropwise over a 5-minute period. Immediate formation of a precipitate was observed and reaction was subsequently stirred at 25 °C. After 1 h, cold diethyl ether (Et_2O, 20 mL) was added to ensure complete precipitation. The precipitate was isolated by filtration and washed with an additional Et_2O (2 x 5 mL). The resulting precipitate (**S1**) was dried under high vacuum for 10 minutes and the yield was subsequently determined. Under an air atmosphere, a screwcap sealed tube was charged with 4-bromobenzenediazonium tetrafluoroborate **S1** (135 mg, 0.5 mmol) and PhCl (2 mL). The mixture was heated to 90 °C for 16 h, after which the lid was unscrewed and white smoke emerged the vial. Reaction yield was determined from the crude reaction mixture by quantitative ^{19}F NMR spectroscopy using 4-fluoroanisole as internal standard.

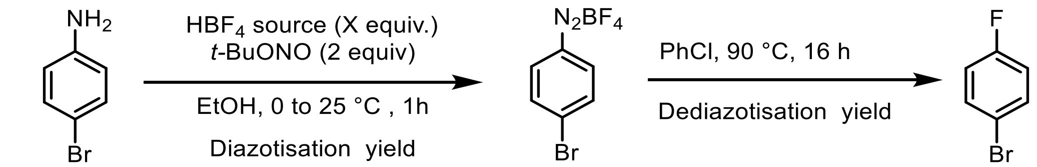

4-bromoaniline **S1** **1**

Table S20. Performance of HBF_4 in the Balz-Schiemann reaction of 4-bromoaniline

Entry	HBF_4	HBF_4 source	HBF_4 (equiv.)	Diazotisation yield (%)	Dediazotisation yield (%)
1	$[HBF_4]^{C}$	Commercial	1.1	93	97
2	$[HBF_4]^{AGF}$	AGF	1.1	90	94
3	$[HBF_4]^{M(I)}$	Metspar	1.1	70	68
4	$[HBF_4]^{M(I)}$	Metspar	1.5	89	78
5	$[HBF_4]^{M(I)}$	Metspar	2.0	92	90

8.5. Preparation of KF from acid grade fluorspar

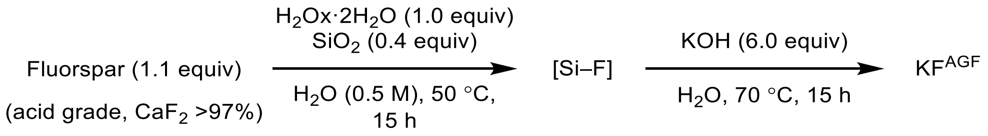

This reaction was performed using 6.44 g of acid grade fluorspar. KF^{AGF} was prepared in a mechanically stirred vessel (500 mL PTFE round bottom flask) using a Teflon stirring shaft (6 mm) with a diamond shaped button (500 mm) purchased from Sigma-Aldrich. The reaction setup can be seen in **Figure S23**.

Oxalic acid dihydrate (9.17 g, 72.7 mmol, 1.0 equiv) was dissolved in H_2O (145.6 mL) in a 500 mL PTFE round bottom flask. A mixture of acid grade fluorspar (97% CaF_2, 6.44 g, 80.0 mmol, 1.1 equiv) and silica gel (1.75 g, 29.0 mmol, 0.4 equiv) was added in 3 portions to the solution of oxalic acid dihydrate at 50 °C. The resulting suspension was stirred using a mechanical stirrer at 50 °C. After 15 h, the suspension was cooled to room temperature, and filtered using a Büchner funnel into a 500 mL PTFE round bottom flask. Filtered solids were washed with H_2O (3 x 5 mL) to give a ~ 160 mL aqueous solution containing H_2SiF_6, H_2SiF_5OH and $OxSiF_4$.

The first step of this reaction affords a theoretical maximum of 24 mmol of H_2SiF_6. Accordingly, KOH (85%, 9.50 g, 144 mmol) was added portion-wise to the aqueous solution (containing H_2SiF_6, H_2SiF_5OH and $OxSiF_4$). After the addition is complete, the reaction mixture was heated with stirring at the 70 °C for 15 h (monitored by ^{19}F NMR spectroscopy). After 15 h a pH of 7 should be reached (acid grade fluorspar derived H_2SiF_6 can be used as a pH regulator). The resulting suspension was allowed to cool to room temperature and the solids were allowed to settle. Filtration using a fritted glass filter followed and insoluble by-products were washed with water (2 x 10 mL). The filtrate was concentrated *in vacuo* (50 °C, 20 mmbar) and dried under high vacuum (< 0.1 mbar). Gentle heating (100 °C) of the flask under vacuum for 5 min followed by drying overnight at room temperature under high vacuum provides acid grade fluorspar derived potassium fluoride (KF^{AGF}) as a white crystalline solid [7.84 g, 136 mmol, 94% (calculated from acid grade fluorspar)].

Purity of KF^{AGF} by quantitative ^{19}F NMR using NaOTf as an internal standard was calculated to be 90%. This gives a total KF^{AGF} yield of 85%. For full purity analysis see **Section 8.8** of **Supplementary Materials.**

PXRD

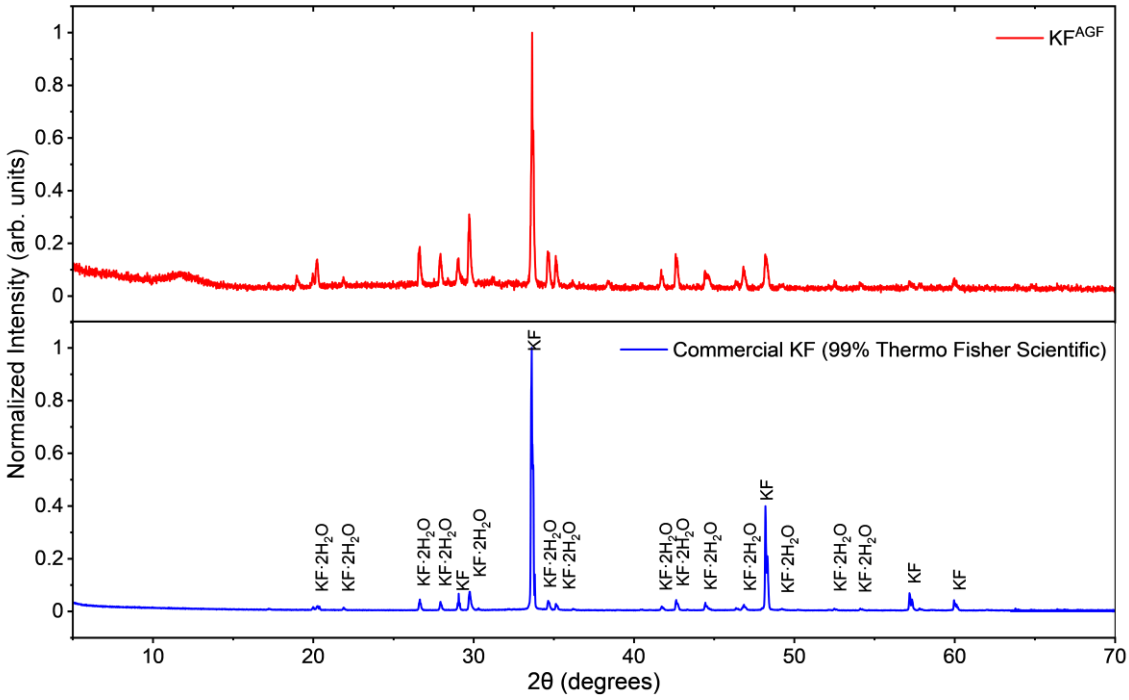

Figure S22. Powder X-ray diffraction pattern of KF^{AGF} (top trace, red) and commercial KF (bottom trace, blue) collected at room temperature. KF^{AGF} is comprised of crystalline anhydrous KF and KF dihydrate.

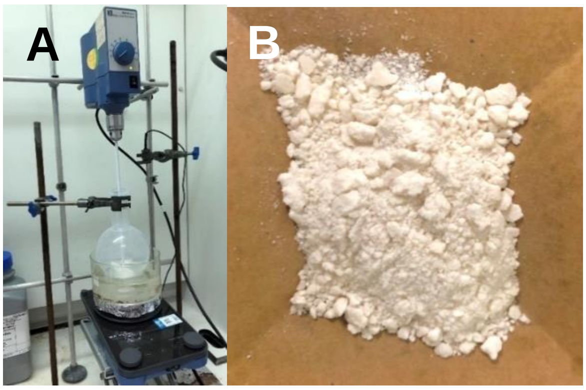

Figure S23. Large scale reaction setup employed in the preparation of KF^{AGF} from acid grade fluorspar using a mechanical stirrer (A) and 7.84 g of KF^{AGF} (B).

8.6. Preparation of KF from metspar I (85% CaF_2)

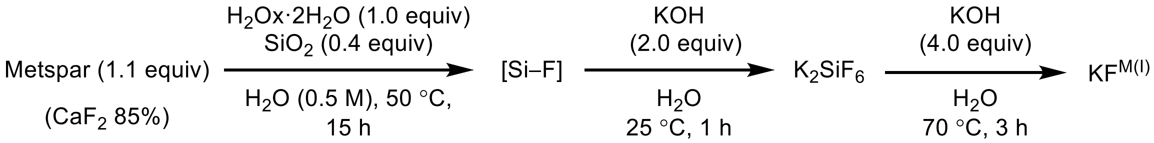

Metspar I sourced from Luoyang Aurora Minechem Co., Ltd. (China) has the following purity profile CaF_2 (85%), SiO_2 (10%), $CaCO_3$ (<5%), S (0.12%), P (0.1%).

Due to the reduced purity of metspar compared to acid grade fluorspar, K_2SiF_6 was first prepared and isolated prior to basic hydrolysis.

Powdered metspar (~ 85% CaF_2, 735 mg, 8.0 mmol, 1.1 equiv), silica gel (175 mg, 2.9 mmol, 0.4 equiv) and oxalic acid dihydrate (917 mg, 7.3 mmol, 1.0 equiv) were weighed into a 100 mL PTFE round bottom flask. H_2O (14.5 mL) was added and the mixture was heated with stirring at 50 °C for 15 h (monitored by ^{19}F NMR spectroscopy). The resulting suspension was cooled to room temperature and filtered with a Büchner funnel into a 50 mL PP tube. Filtered solids were washed with H_2O (5 mL) to give an aqueous solution (~ 20 mL) containing H_2SiF_6, H_2SiF_5OH and $OxSiF_4$.

The first step of this reaction affords a theoretical maximum of 2.4 mmol of H_2SiF_6. Accordingly, KOH (85%, 317 mg, 4.8 mmol) was added to the filtrate (until pH 7 was reached). After the addition is complete, the resultant suspension is stirred at 25 °C for 1 h (monitored by ^{19}F NMR spectroscopy) before filtration. The filtered solid was allowed to dry at room temperature overnight to give K_2SiF_6 as a white powder (393 mg, 1.33 mmol, 74%).

To the metspar derived K_2SiF_6 (393 mg, 1.78 mmol) was added a solution of KOH (85%, 471 mg, 7.41 mmol) in water (0.7 mL). The reaction mixture was stirred at 70 °C for 3 h. The resulting suspension was allowed to cool to room temperature and the solids were allowed to settle. Filtration using a fritted glass filter followed and insoluble by-products were washed with water (2 x 5 mL). The filtrate was concentrated *in vacuo* (50 °C, 20 mmbar) and dried under high vacuum (< 0.1 mbar). Gentle heating (100 °C) of the flask under vacuum for 5 min followed by drying overnight under high vacuum at room temperature provides metspar derived potassium fluoride [KF$^{M(I)}$] as a white crystalline solid [458 mg, 7.88 mmol, 74%].

Purity of KF^{M} by quantitative ^{19}F NMR using NaOTf as an internal standard was calculated to be 98%.

This gives a total KF$^{M(I)}$ yield of 72% (7.73 mmol, calculated from K_2SiF_6), 54% [calculated from metspar I where 1 equivalent CaF_2 (7.27 mmol) theoretically affords 2 equivalents KF (14.54 mmol)]. For full purity analysis see **Section 8.8** of **Supplementary Materials.**

PXRD

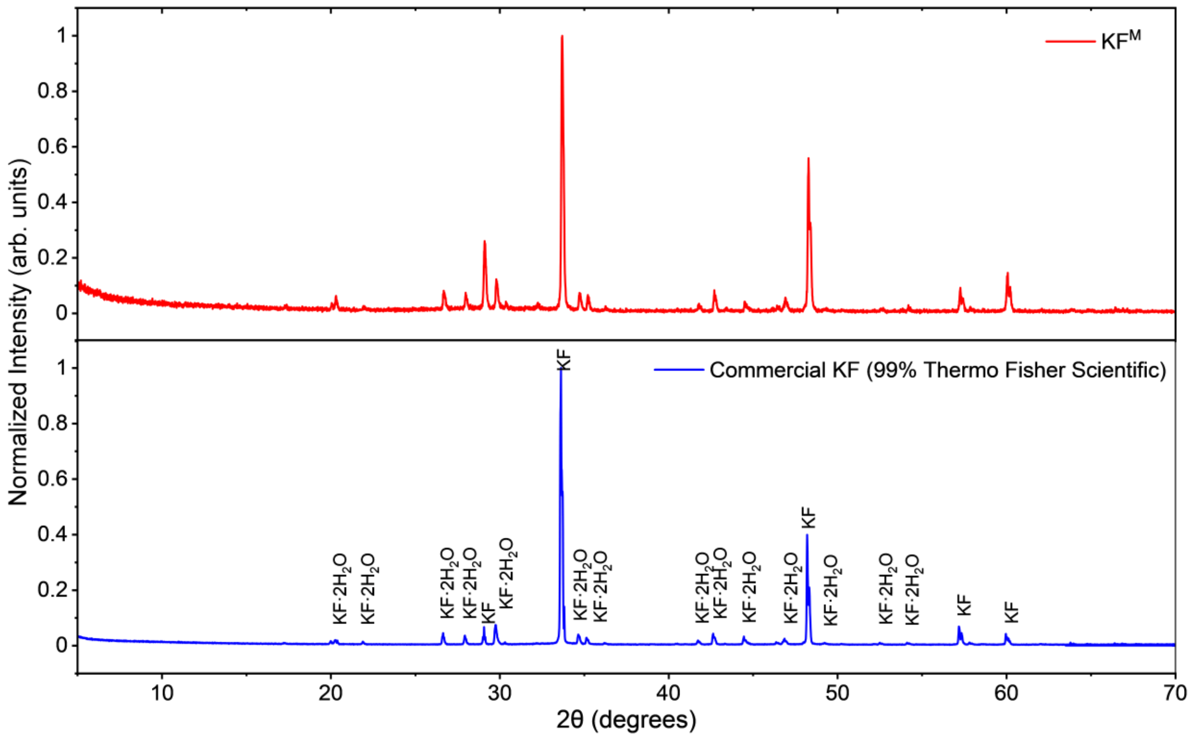

Figure S24. Powder X-ray diffraction pattern of KF$^{M(I)}$ (top trace, red) and commercial KF (bottom trace, blue). KF$^{M(I)}$ is comprised of crystalline anhydrous KF and KF dihydrate.

8.7. Preparation of KF from metspar II (89% CaF_2)

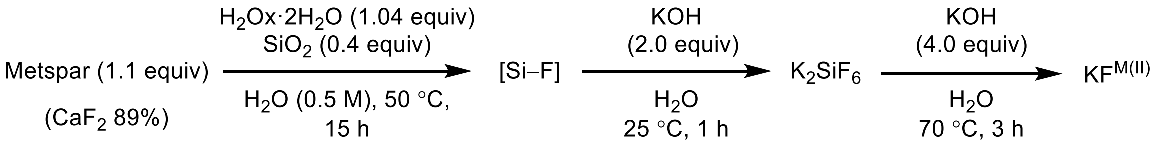

Metspar II was sourced from Mexico (Mexichem Fluor S.A de C.V.) **Metspar^{II}** with the following purity profile: CaF_2 (88.98%), SiO_2 (5.43%), $CaCO_3$ (4.02%), Al_2O_3 (0.41%), Fe_2O_3 (0.24%), S (0.011%), P (0.023%), Pb (<0.001%).

Due to the reduced purity of metspar compared to acid grade fluorspar, K_2SiF_6 was first prepared and isolated prior to basic hydrolysis. Additional 0.04 equiv. of $H_2Ox{\cdot}2H_2O$ was added to the reaction to neutralize 4% $CaCO_3$ found in metspar II.

Powdered metspar (89% CaF_2, 1.40 g, 16.0 mmol, 1.1 equiv), silica gel (350 mg, 5.82 mmol, 0.4 equiv) and oxalic acid dihydrate (1.90 g, 15.1 mmol, 1.04 equiv) were weighed into a 100 mL PTFE round bottom flask. H_2O (29 mL) was added and the mixture was heated with stirring at 50 °C for 15 h (monitored by ^{19}F NMR spectroscopy). The resulting suspension was cooled to room temperature and filtered with a Büchner funnel into a 50 mL PP tube. Filtered solids were washed with H_2O (5 mL) to give an aqueous solution (~ 35 mL) containing H_2SiF_6, H_2SiF_5OH and $OxSiF_4$.

The first step of this reaction affords a theoretical maximum of 4.8 mmol of H_2SiF_6. Accordingly, KOH (85%, 634 mg, 9.60 mmol) was added to the filtrate (until pH 7 was reached). After the addition is complete, the resultant suspension is stirred at 25 °C for 1 h (monitored by ^{19}F NMR spectroscopy) before filtration. The filtered solid was allowed to dry at room temperature overnight to give K_2SiF_6 as a white powder (873 mg, 3.96 mmol, 83%).

To the metspar derived K_2SiF_6 (873 mg, 3.96 mmol) was added a solution of KOH (85%, 1.05 g, 15.9 mmol) in water (10 mL). The reaction mixture was stirred at 70 °C for 3 h. The resulting suspension was allowed to cool to room temperature and the solids were allowed to settle. Filtration using a fritted glass filter followed and insoluble by-products were washed with water (2 x 5 mL). The filtrate was concentrated *in vacuo* (50 °C, 20 mmbar) and dried under high vacuum (< 0.1 mbar). Gentle heating (100 °C) of the flask under vacuum for 5 min followed by drying overnight under high vacuum at room temperature provides metspar^{II} derived potassium fluoride [$KF^{M(II)}$] as a white crystalline solid [1.18 g, 20.31 mmol, 85%].

Purity of $KF^{M(II)}$ by quantitative ^{19}F NMR using NaOTf as an internal standard was calculated to be 90%.

This gives a total $KF^{M(II)}$ yield of 77% (18.28 mmol, calculated from K_2SiF_6), 63% (calculated from metspar II where 1 equivalent CaF_2 (14.54 mmol) theoretically affords 2 equivalents KF (29.1 mmol)]). For full purity analysis see **Section 8.8** of **Supplementary Materials.**

8.8. Purity Analysis of KF

8.8.1. Purity analysis by ^{19}F NMR

To assess the quantity of KF in the solid product, a sample of the solid product and sodium triflate (>98 %) as an internal standard was dissolved in D_2O. The purity of KF in the solid was assessed by quantitative ^{19}F NMR (16 scans, d_1= 50 s, o1p = 100.5 ppm).

The purity of compound (x), as a percentage of its nominal weight, can be determined using the following formula as described in reference [46]:

$$P_x = (I_x/I_{is}) \times (N_{is}/N_x) \times (M_x/M_{is}) \times (W_{is}/W_x) \times P_{is} \quad (1)$$

where, *I*, *N*, *M*, *W* and *P* are the integrated area (*I*), number of nuclei (*N*), molecular mass (*M*), gravimetric weight (*W*) and purity (*P*) of the compound of interest (x) and the internal standard compound (is), respectively. Because this is a weight-based % purity, it allows you to evaluate the purity even if other components in the sample are invisible by NMR.

Using the following equation (2) a KF yield (%) was determined for KF^{AGF} and KF^{M}.

purity of KF x mass of solid product = KF yield (%) (2)

KF^{AGF}

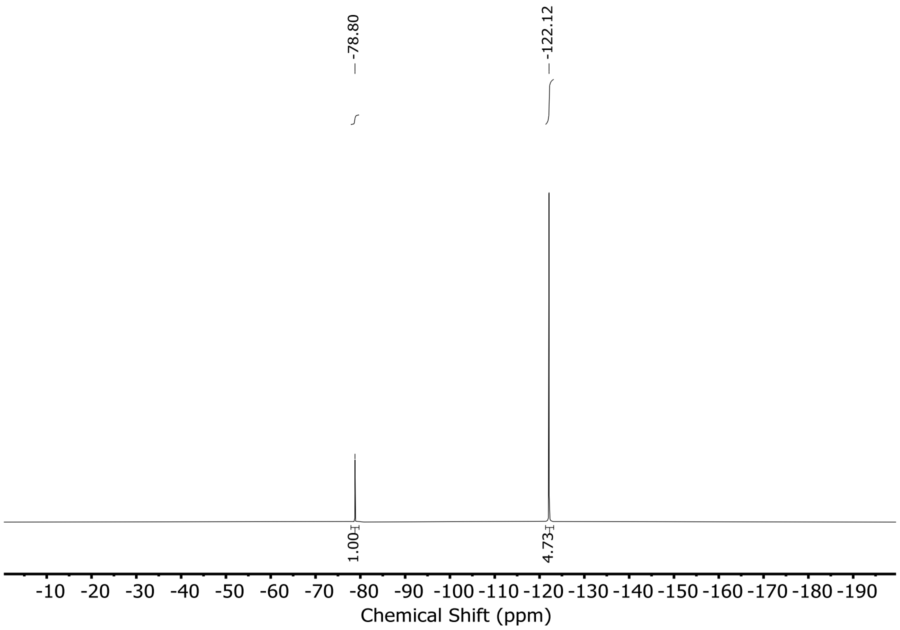

Figure S25. ^{19}F qNMR (D_2O) of KF^{AGF} dissolved in D_2O with NaOTf internal standard. KF at -122.12 ppm and NaOTf at -78.80 ppm.

A sample of KF^{AGF} (23.4 mg) and sodium triflate as an NaOTf (4.40 mg) was dissolved in D_2O. The purity of KF in the solid was assessed by quantitative ^{19}F NMR and calculated to be 90%.
^{19}F NMR (377 MHz, D_2O) δ -122.12 (KF, s). No signals were observed by ^{13}C NMR.

KF$^{M(I)}$

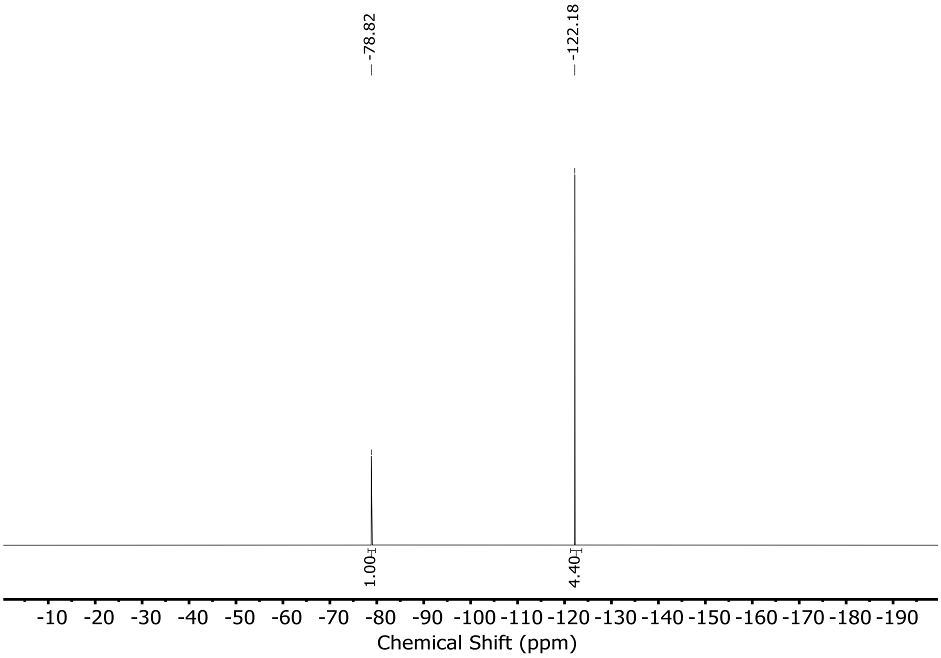

Figure S26. ^{19}F qNMR (D_2O) of KF$^{M(I)}$ dissolved in D_2O with NaOTf internal standard. KF at -122.12 ppm and NaOTf at -78.80 ppm.

A sample of KF$^{M(I)}$ (15.5 mg) and sodium triflate as an NaOTf (3.40 mg) was dissolved in D_2O. The purity of KF$^{M(I)}$ in the solid was assessed by quantitative ^{19}F NMR and calculated to be 98%.
^{19}F NMR (377 MHz, D_2O) δ -122.18 (KF, s). No signals were observed by ^{13}C NMR.

KF$^{M(II)}$

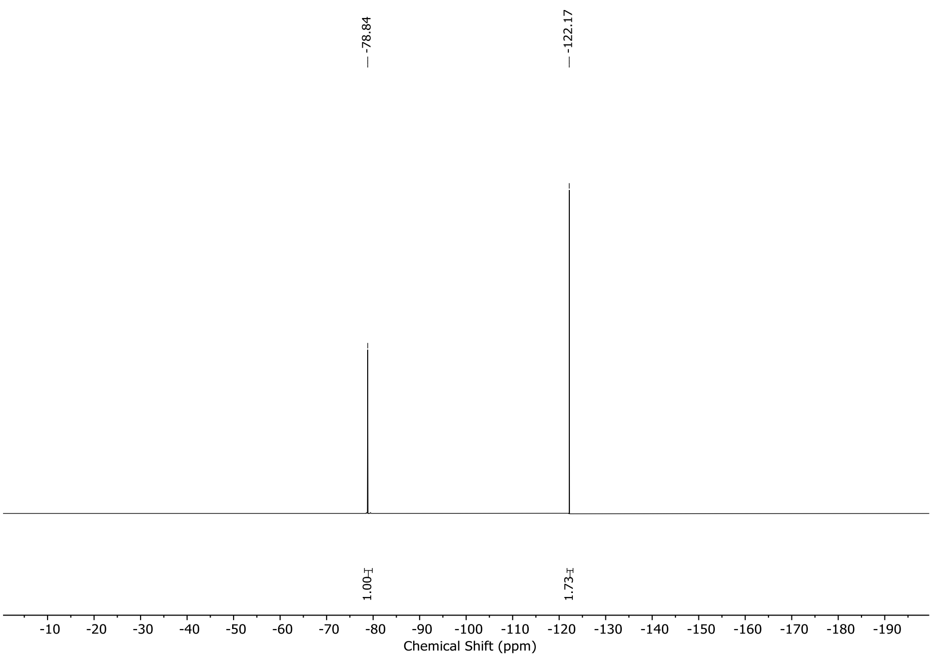

Figure S27. ^{19}F qNMR (D_2O) of KF$^{M(II)}$ dissolved in D_2O with NaOTf internal standard. KF at -122.12 ppm and NaOTf at -78.80 ppm.

A sample of KF$^{M(II)}$ (19.4 mg) and sodium triflate as an NaOTf (10.0 mg) was dissolved in D_2O. The purity of KF$^{M(II)}$ in the solid was assessed by quantitative ^{19}F NMR and calculated to be 90%.
^{19}F NMR (377 MHz, D_2O) δ -122.21 (KF, s). No signals were observed by ^{13}C NMR

8.8.2. ICP OES

Inductively Coupled Plasma Optical Emission spectroscopy (ICP-OES) for microanalyses (F, K, Si) of commercial KF samples, KF^{AGF} and $KF^{M(I)}$ was carried out by MEDAC Ltd.

Table S21. Microanalysis of KF samples

Element	KF (theoretical)	KF Thermo Scientific 99% (metals basis)	KF Sigma Aldrich >99.9% (metals basis)	KF^{AGF}	$KF^{M(I)}$
F (%)	32.70	29.88	31.48	23.76	28.30
K (%)	67.30	61.71	64.70	56.27	62.5
Si (%)	0	n/a	n/a	0.4	0.17

KF exists as anhydrous KF and $KF{\cdot}2H_2O$ [as observed in the PXRD patterns of commercial KF, KF^{AGF} and $KF^{M(I)}$].

8.8.3. TGA-MS

Thermogravimetric analysis coupled with mass spectroscopy (TGA-MS) was recorded for KF^{AGF} to identify impurities. The measurement was performed under flowing nitrogen. KF^{AGF} demonstrates a weight loss of 9% within 36 - 130 °C which is attributed to H_2O.

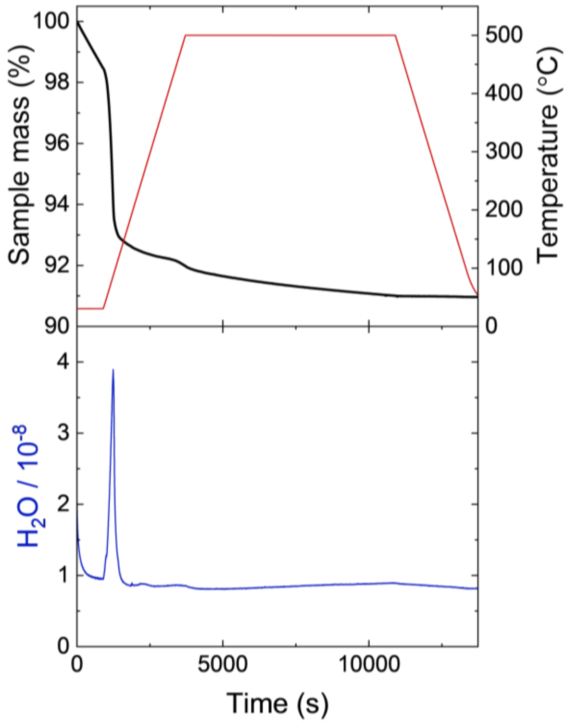

Figure S28. TGA of KF^{AGF} (top graph) with sample mass % (black), temperature (red) and m/e 18 ion trace (bottom graph, blue).

8.9. Performance of prepared KF

The performance of our KF prepared either using acid grade fluorspar (KF^{AGF}, 90% purity) or metspar ($KF^{M(I)}$, 98% purity) was examined in the fluorination of 2,4-dinitrochlorobenzene and compared against commercial anhydrous KF (KF^{C}, 99% purity, Thermo Scientific Chemicals, CAS 7789-23-3)

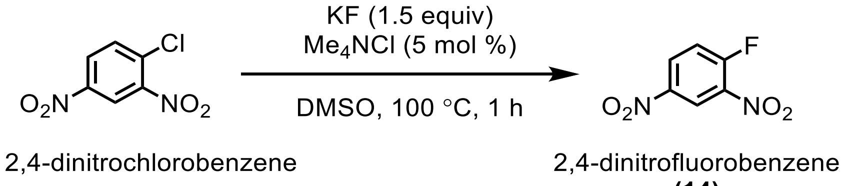

(14)

To an oven dried glass reaction vial was added 2,4-dinitrochlorobenzene (101 mg, 0.5 mmol, 1.0 equiv), KF (22 mg, 0.75 mmol, 1.5 equiv), Me_4NCl (2.7 mg, 0.025 mmol, 0.05 equiv) and anhydrous DMSO (2.5 mL). After stirring at 100 °C for 1 h in a heating block, the resulting suspension was cooled to room temperature. Reaction yield was determined from the crude reaction mixture by quantitative ^1H and ^{19}F NMR spectroscopy using 4-fluoroanisole as internal standard.

Table S22. Performance of KF^{AGF} and $KF^{M(I)}$

Entry	KF	source	ArF (%)
1	KF^{C}	commercial	95%
2	KF^{AGF}	this work	90%
3	$KF^{M(I)}$	this work	92%

8.10. Preparation of $R_4NF{\cdot}(ROH)_x$ from acid grade fluorspar

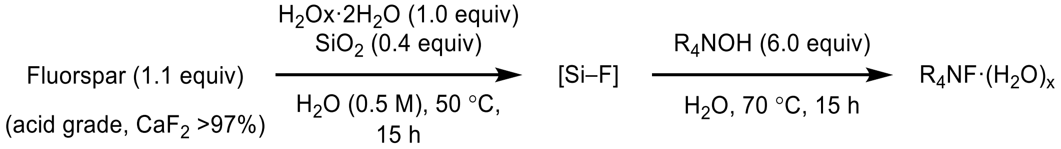

Acid grade fluorspar (644 mg, 8.0 mmol, 1.1 equiv), silica gel (175 mg, 2.9 mmol, 0.4 equiv) and oxalic acid dihydrate (917 mg, 7.3 mmol, 1.0 equiv) were weighed into a 100 mL PTFE round bottom flask. H_2O (14.5 mL) was added and the mixture was heated with stirring at 50 °C for 15 h (monitored by ^{19}F NMR spectroscopy). The resulting suspension was cooled to ambient temperature and filtered with a Büchner funnel into a 50 mL PP tube. Filtered solids were washed with H_2O (5 mL) to give an aqueous solution (~ 20 mL) containing H_2SiF_6, H_2SiF_5OH and $OxSiF_4$.

The first step of this reaction affords a theoretical maximum of 2.4 mmol of H_2SiF_6. Accordingly, Me_4NOH (25 wt. % in H_2O, 5.17 mL, 14.4 mmol) or $^nBu_4NOH$ (40 wt. % in H_2O, 9.44 mL, 14.4 mmol) was added portion wise to the aqueous solution (containing H_2SiF_6, H_2SiF_5OH and $OxSiF_4$). After the addition is complete, the reaction was heated with stirring at 70 °C for 15 h (monitored by ^{19}F NMR spectroscopy). Filtration using a Büchner funnel followed and insoluble by-products were washed with water (2 x 5 mL). The filtrate was concentrated *in vacuo* (50 °C, 20 mmbar) and dried under high vacuum (< 0.1 mbar). Drying overnight under high vacuum at room

temperature provides crude tetramethylammonium fluoride (TMAF, Me_4NF) or crude *n*-tetrabutylammonium fluoride (TBAF, $n$$Bu_4NF$) as hydrates.

$Me_4NF{\cdot}{}^tAmOH$

To the crude tetramethylammonium fluoride (TMAF, Me_4NF) hydrate (1.01 g) was added anhydrous *tert*-amyl alcohol (40 mL) and activated 3Å molecular sieves (powdered, 20 g). The slurry was stirred at 25 °C for 3 h. The slurry was then filtered to remove the solids and the solids were washed with *tert*-amyl alcohol (3 × 5 mL). The filtrate and alcohol washes were combined and concentrated *in vacuo* (40 °C, 12 mmbar) to yield $Me_4NF{\cdot}{}^tAmOH$ as a white solid (2.3 g, 12.7 mmol, 88%).

^{1}H NMR (400 MHz, $CDCl_3$) δ 3.30 – 3.21 (m, 8H), 1.60 (dq, *J* = 11.9, 8.0 Hz, 8H), 1.40 (dt, *J* = 14.7, 7.4 Hz, 8H), 1.20 (s, 36H), 0.95 (t, *J* = 7.3 Hz, 12H).
^{19}F NMR (376 MHz, $CDCl_3$) δ -120.19 (br s).
^{13}C NMR (101 MHz, $CDCl_3$) δ 68.5, 58.6, 31.2, 24.0, 19.7, 13.6.
Spectroscopic data are in accordance with those in literature [24].

$Bu_4NF{\cdot}({}^nBuOH)_4$

To the crude tetrabutylammonium fluoride (TBAF, $^nBu_4NF$) hydrate (3.86 g) was added anhydrous *tert*-butanol (200 mL) and hexane (50 mL). The slurry was stirred at 90 °C for 30 min. The slurry was then filtered whilst warm and the filtrate was cooled to room temperature before storing at 5 °C overnight. A white crystalline solid precipitated out of the filtrate which was filtered and washed with hexane. The crystalline precipitate was collected and dried under high vacuum for 15 min to give $^nBu_4NF{\cdot}({}^tBuOH)_4$ as a fluffy crystalline solid (5.79 g, 10.4 mmol, 71%).

^{1}H NMR (400 MHz, D_2O) δ 3.10 (s, 12H), 1.42 (q, *J* = 7.5 Hz, 2H), 1.10 (d, *J* = 1.2 Hz, 5H), 0.80 (dd, *J* = 8.2, 6.9 Hz, 2H).
^{19}F NMR (377 MHz, D_2O) δ -122.23 (s).
^{13}C NMR (101 MHz, D_2O) δ 72.1, 55.8 – 54.1 (m), 35.2, 27.1, 7.8.
Spectroscopic data are in accordance with those in literature [25].

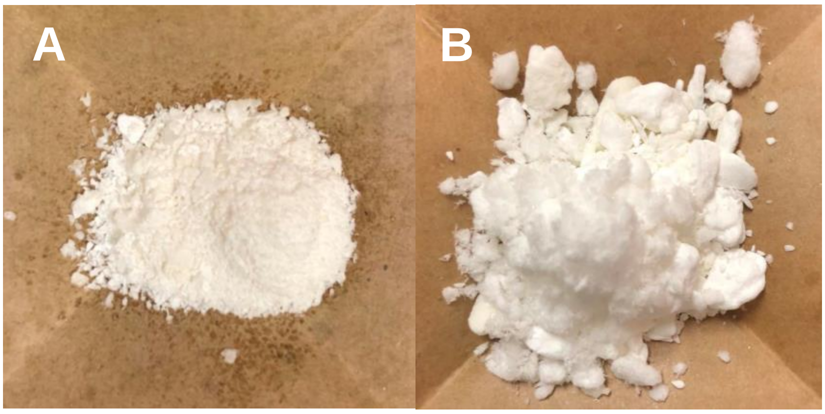

Figure S29. Acid grade fluorspar derived $Me_4NF{\cdot}{}^tAmOH$ (A) and $Bu_4NF{\cdot}({}^nBuOH)_4$ (B)

8.11. Performance of acid grade fluorspar derived $Me_4NF\cdot{}^tAmOH$

The performance of our acid grade fluorspar derived $Me_4NF\cdot{}^tAmOH$ was examined in the fluorination of 1,4-dinitrobenzene following a modified procedure by Sanford and co-workers [24].

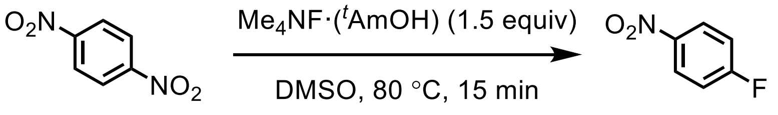

1,4-dinitrobenzene   4-fluoronitrobenzene (**19**)

To an oven dried glass reaction vial was added 1,4-dinitrobenzene (84 mg, 0.5 mmol, 1.0 equiv), acid grade fluorspar derived $Me_4NF\cdot(^tAmOH)$ (136 mg, 0.75 mmol, 1.5 equiv) and anhydrous DMSO (2.5 mL). After stirring at 80 °C for 15 minutes in a heating block, the resulting suspension was cooled to room temperature. Reaction yield was determined from the crude reaction mixture by quantitative ^1H and ^{19}F NMR spectroscopy using 4-fluoroanisole as internal standard. Yield of 97% (determined by ^{19}F NMR against 4-fluoroanisole as an internal standard).

8.12. Preparation of NaF from acid grade fluorspar

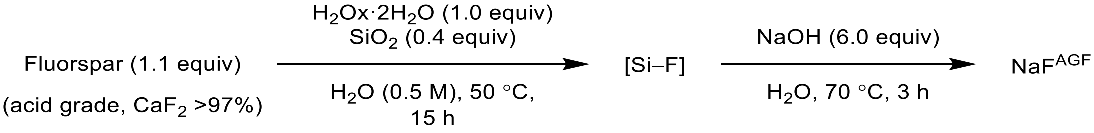

Acid grade fluorspar (644.0 mg, 8.0 mmol, 1.1 equiv), silica gel (306.0 mg, 5.1 mmol, 0.4 equiv) and oxalic acid (951 mg, 7.3 mmol, 1.0 equiv) were weighed into a 50 mL conical sterile polypropylene (PP) centrifuge tube. H_2O (14.5 mL) was added and the mixture was stirred at 50 °C for 15 h.

The resulting suspension was cooled to ambient temperature, diluted with H_2O (5 mL) and filtered using a Büchner funnel into a 50 mL PP tube. Filtered solids were washed with H_2O to give an aqueous solution (~ 20 mL) containing [Si-F] (H_2SiF_6, H_2SiF_5OH and $OxSiF_4$).

The first step of this reaction affords a theoretical maximum of 2.4 mmol of H_2SiF_6. Accordingly, NaOH (98%, 588 mg, 14.4 mmol) was added portion-wise to the aqueous solution (containing H_2SiF_6 and H_2SiF_5OH). The reaction was heated with stirring at the 70 °C for 3 h (monitored by ^{19}F NMR spectroscopy). After 15 h a pH of 7 should be reached (acid grade fluorspar derived H_2SiF_6 can be used as a pH regulator). The resulting suspension was filtered using a fritted glass filter to separate insoluble by-products from the solution and washed with water (2 x 5 mL). The filtrate was concentrated *in vacuo* (50 °C) and dried under high vacuum (< 0.1 mbar). Gentle heating (100 °C) of the flask under vacuum for 5 min followed by drying overnight at room temperature under high vacuum provided NaF^{AGF} as a white solid [541 mg, 12.9 mmol, 90% (calculated from acid grade fluorspar)].

Purity of NaF^{AGF} by quantitative ^{19}F NMR using NaOTf as an internal standard was calculated to be 94%. This gives a total NaF^{AGF} yield of 85%. For full purity analysis provided below.

PXRD

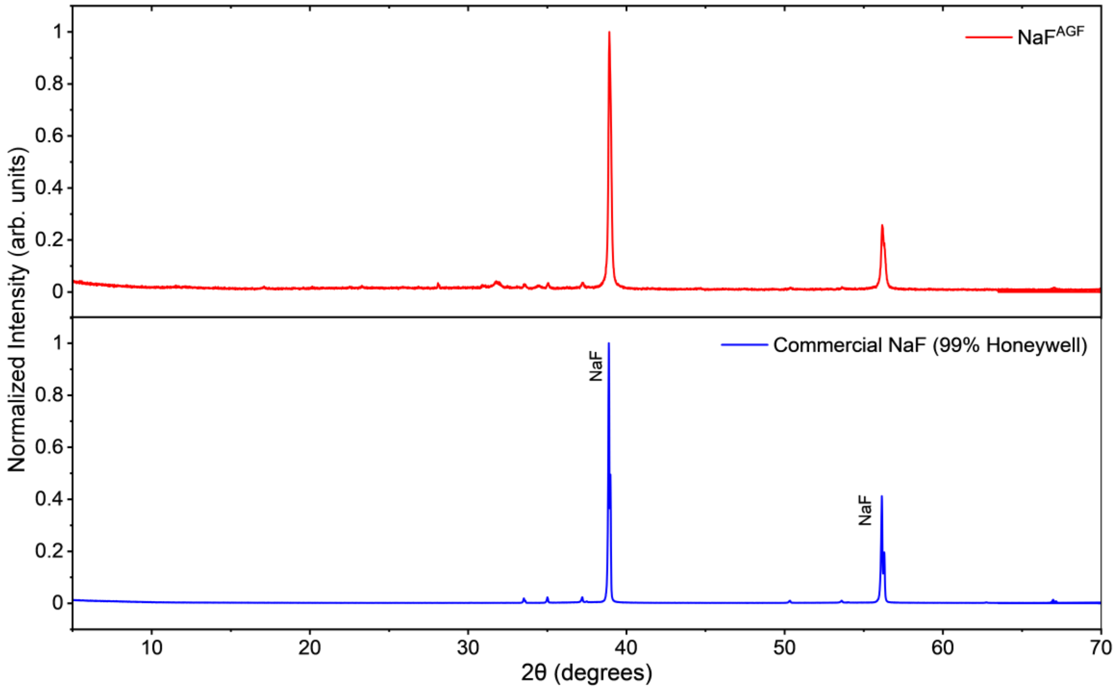

Figure S30. Powder X-ray diffraction pattern of NaF^{AGF} (top trace, red) and commercial NaF (bottom trace, blue). NaF^{AGF} is comprised of crystalline anhydrous NaF.

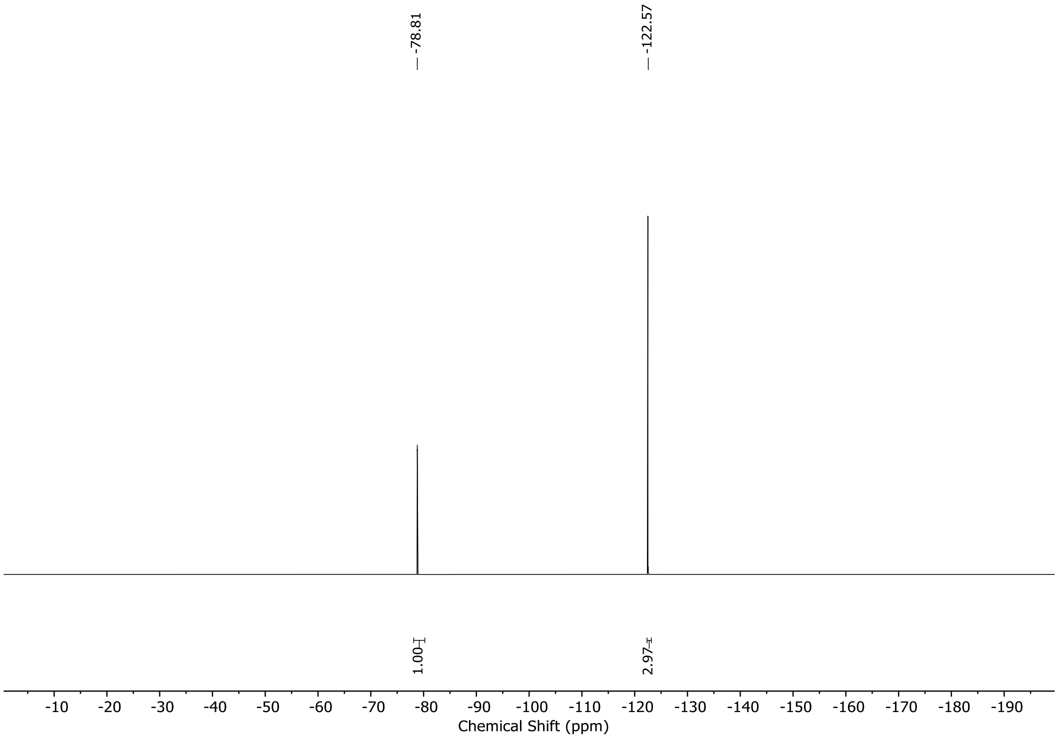

Figure S31. ^{19}F qNMR (D_2O) of NaF^{AGF} dissolved in D_2O with NaOTf internal standard. NaF at -122.57 ppm and NaOTf at -78.81 ppm.

A sample of NaF^{AGF} (21.0 mg) and sodium triflate as an NaOTf (9.1 mg) was dissolved in D_2O. The purity of NaF in the solid was assessed by quantitative ^{19}F NMR and calculated to be 94%.
^{19}F NMR (377 MHz, D_2O) δ -121.57 (NaF, s).

Elemental Analysis of NaF^{AGF}

Inductively Coupled Plasma Optical Emission spectroscopy (ICP-OES) for microanalyses of commercial NaF, and NaF^{AGF} was carried out by MEDAC Ltd.

Table S23. Microanalysis of NaF samples

Element	NaF (theoretical)	NaF Honeywell 99%	NaF^{AGF}
F (%)	45.25	46.52	45.23
Na (%)	54.75	52.81	54.63

8.13. Preparation of CsF from acid grade fluorspar

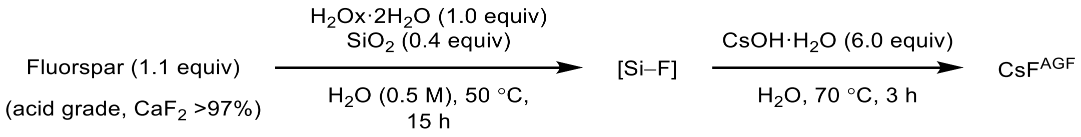

Acid grade fluorspar (644.0 mg, 8.0 mmol, 1.1 equiv), silica gel (306.0 mg, 5.1 mmol, 0.4 equiv) and oxalic acid (951 mg, 7.3 mmol, 1.0 equiv) were weighed into a 50 mL conical sterile polypropylene (PP) centrifuge tube. H_2O (14.5 mL) was added and the mixture was stirred at 50 °C for 15 h.

The resulting suspension was cooled to ambient temperature, diluted with H_2O (5 mL) and filtered using a Büchner funnel into a 50 mL PP tube. Filtered solids were washed with H_2O to give an aqueous solution (~ 20 mL) containing [Si-F] (H_2SiF_6, H_2SiF_5OH and $OxSiF_4$).

The first step of this reaction affords a theoretical maximum of 2.4 mmol of H_2SiF_6. Accordingly, $CsOH·H_2O$ (2.42 g, 14.4 mmol) was added portion-wise to the aqueous solution (containing H_2SiF_6 and H_2SiF_5OH). The reaction was heated with stirring at the 70 °C for 3 h (monitored by ^{19}F NMR spectroscopy). After 15 h a pH of 7 should be reached (acid grade fluorspar derived H_2SiF_6 can be used as a pH regulator). The resulting suspension was filtered using a fritted glass filter to separate insoluble by-products from the solution and washed with water (2 x 5 mL). The filtrate was concentrated *in vacuo* (50 °C) and dried under high vacuum (< 0.1 mbar). Gentle heating (100 °C) of the flask under vacuum for 5 min followed by drying overnight at room temperature under high vacuum provided CsF^{AGF} as a white solid [2.04 g, 13.9 mmol, 93% (calculated from acid grade fluorspar)].

Purity of CsF^{AGF} by quantitative ^{19}F NMR using NaOTf as an internal standard was calculated to be 96%. This gives a total CsF^{AGF} yield of 89%. For full purity analysis provided below.

A sample of CsF^{AGF} could not be analyzed by PXRD because the sample is highly hygroscopic and deliquesces.

CsF^AGF^

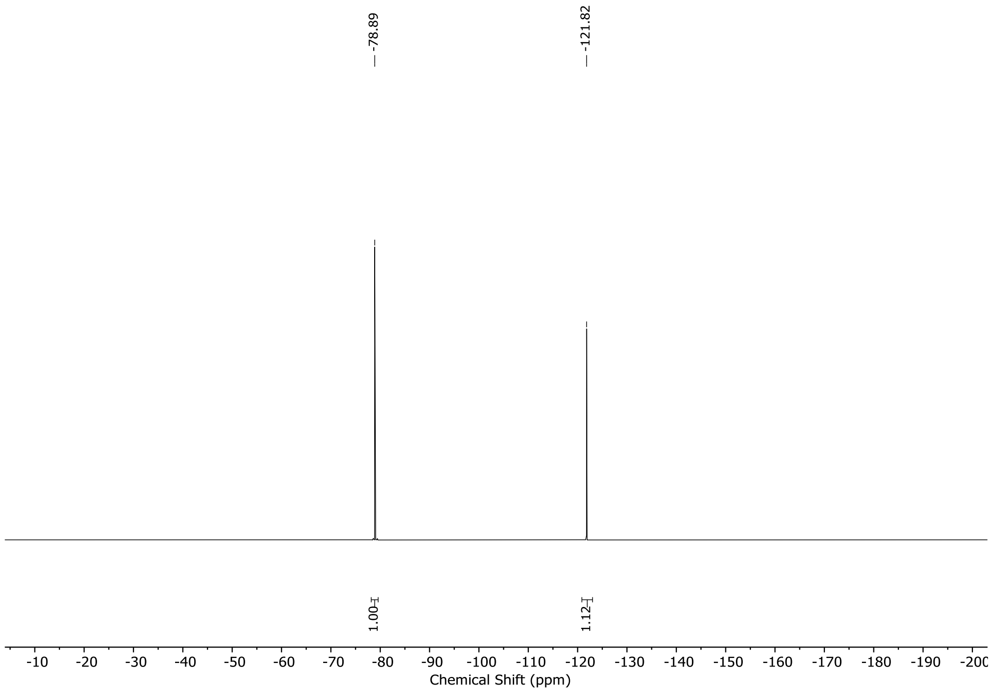

Figure S32. ^{19}F qNMR (D_2O) of CsF^{AGF} dissolved in D_2O with NaOTf internal standard. CsF at -121.82 ppm and NaOTf at -78.89 ppm.

A sample of CsF^{AGF} (24.8 mg) and sodium triflate as an NaOTf (8.0 mg) was dissolved in D_2O. The purity of CsF in the solid was assessed by quantitative ^{19}F NMR and calculated to be 96%.
^{19}F NMR (377 MHz, D_2O) δ -121.82 (CsF, s).

Elemental Analysis of CsF^{AGF}

Inductively Coupled Plasma Optical Emission spectroscopy (ICP-OES) for microanalyses of commercial CsF and CsF^{AGF} was carried out by MEDAC Ltd. Cs cannot be detected by ICP-OES.

Table S24. Microanalysis of CsF samples

Element	CsF (theoretical)	CsF Sigma-Aldrich 97%	CsF^{AGF}
F (%)	12.51	12.59	11.47

8.14. Performance of prepared CsF

The performance of our CsF prepared either using acid grade fluorspar (CsF^{AGF}) was examined in the fluorination of 2-chloroquinoline and compared against commercial anhydrous CsF (CsF^{C}, 99% purity, Sigma Aldrich CAS 13400-13-0) following a protocol outlined in ref[50].

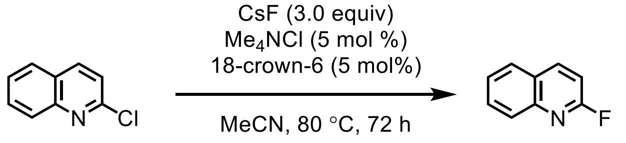

2-chloroquinoline 2-fluoroquinoline

The reactivity of CsF^{AGF} (3 equiv) was tested in the fluorination of 2-chloroquinoline (0.25 mmol, 1 equiv) in the presence of Me_4NCl (5 mol%) and 18-crown-6 (5 mol%) in anh. MeCN at 80 °C for 72 h. Reaction yield was determined from the crude reaction mixture by quantitative ^1H and ^{19}F NMR spectroscopy using 4-fluoroanisole as internal standard.

Table S25. Performance of CsF^{AGF} and CsF^{C}

Entry	CsF	source	ArF (%)	ArCl (%)
1	CsF^{C}	commercial	69	31
2	CsF^{AGF}	this work	73	27

9. Preparation of HBF_4 using oxalic acid dihydrate at 25 °C

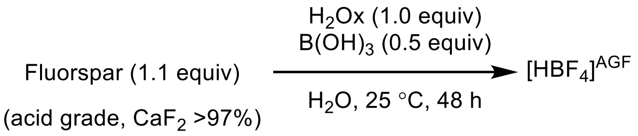

Acid grade fluorspar (1.39 g, 17.79 mmol, 1.1 equiv), boric acid (0.50 g, 8.09 mmol, 0.5 equiv), water (4 mL) and oxalic acid dihydrate (1.46 g, 16.2 mmol, 1.0 equiv) were weighed into a 50 mL conical sterile polypropylene (PP) centrifuge tube. The reaction was stirred at ambient temperature for 48 h. NaOTf (348 mg, 2.02 mmol) was added as an internal standard and the crude mixture was diluted with 2 mL of water and stirred for an additional 2 min to ensure the complete dissolution of NaOTf. The suspension was allowed to settle and an aliquot was withdrawn from the supernatant to determine the total amount of HBF_4 and HBF_3OH by quantitative ^{19}F NMR spectroscopy (D_2O). Aliquot analysis by ^{19}F NMR using NaOTf as internal standard revealed a yield of 82% HBF_4 and 16% HBF_3OH mixture (<1% $HOxBF_2$).

10. Preparation of KF using oxalic acid dihydrate at 25 °C

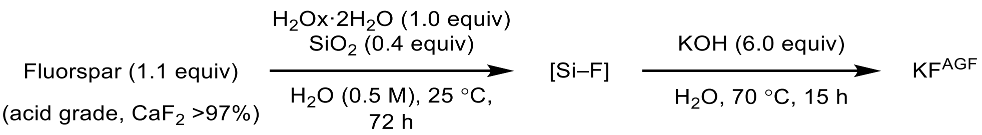

Acid grade fluorspar (644.0 mg, 8.0 mmol, 1.1 equiv), silica gel (306.0 mg, 5.1 mmol, 0.4 equiv) and oxalic acid (951 mg, 7.3 mmol, 1.0 equiv) were weighed into a 50 mL conical sterile polypropylene (PP) centrifuge tube. H_2O (14.5 mL) was added and the mixture was stirred at 25 °C for 72 h.

The resulting suspension was cooled to ambient temperature, diluted with H_2O (5 mL) and filtered using a Büchner funnel into a 50 mL PP tube. Filtered solids were washed with H_2O to give an aqueous solution (~ 20 mL) containing [Si-F] (H_2SiF_6, H_2SiF_5OH and $OxSiF_4$).

The first step of this reaction affords a theoretical maximum of 2.4 mmol of H_2SiF_6. Accordingly, KOH (85%, 951 mg, 14.4 mmol) was added portion-wise to the aqueous solution (containing H_2SiF_6 and H_2SiF_5OH). The reaction was heated with stirring at the 70 °C for 3 h. The resulting suspension was filtered using a fritted glass filter to separate insoluble by-products from the solution and washed with water (2 x 5 mL). The filtrate was concentrated *in vacuo* (50 °C) and dried under high vacuum (< 0.1 mbar). Gentle heating (100 °C) of the flask under vacuum for 5 min followed by drying overnight at room temperature under high vacuum provides acid-grade fluorspar derived potassium fluoride (KF^{AGF}) as a white crystalline solid (798 mg, 13.7 mmol, 95 %).

Purity of KF^{AGF} by quantitative ^{19}F NMR using NaOTf as an internal standard was calculated to be 78%. This gives a total KF^{AGF} yield of 74%. Purity analysis can be seen below.

KF^{AGF} (prepared at 25 °C)

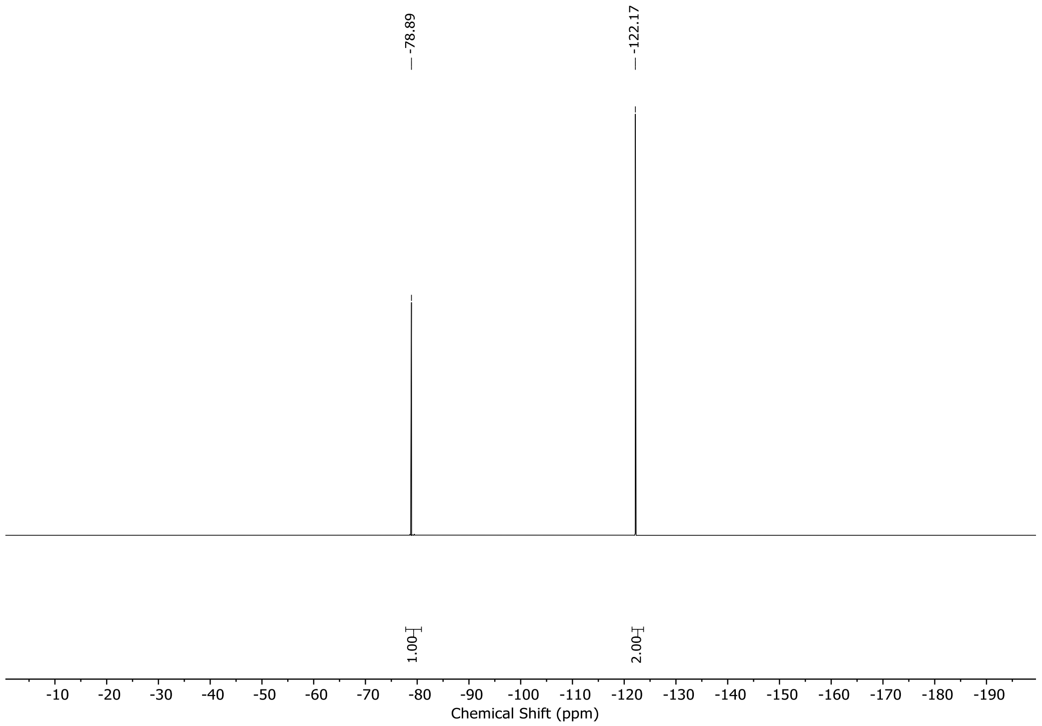

Figure S33. A sample of KF^{AGF} (20.9 mg) and sodium triflate as an NaOTf (8.0 mg) was dissolved in D_2O. The purity of KF in the solid was assessed by quantitative ^{19}F NMR and calculated to be 78%.

^{19}F NMR (377 MHz, D_2O) δ -121.17 (KF, s).

11. Preparation of HBF_4 using sulfuric acid at 50 °C and 25 °C

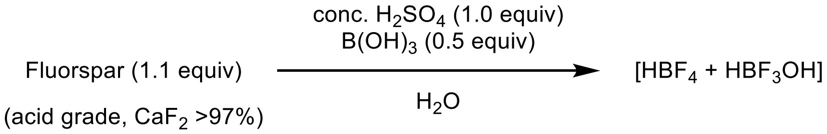

Acid grade fluorspar (1.39 g, 17.79 mmol, 1.1 equiv), boric acid (0.50 g, 8.09 mmol, 0.5 equiv), water (4 mL), concentrated 95% H_2SO_4 (0.898 mL, 17.7 M, 16.2 mmol, 1.0 equiv) were weighed into a 50 mL conical sterile polypropylene (PP) centrifuge tube. An exothermic reaction was observed. The reaction was stirred at 25 °C or 50 °C for specified time. NaOTf (347.8 mg, 2.02 mmol) was added as an internal standard and the crude mixture was diluted with 2 mL of H_2O and stirred for an additional 2 min to ensure the complete dissolution of NaOTf. The suspension was allowed to settle and an aliquot was withdrawn from the supernatant to determine the total amount of HBF_4 and HBF_3OH by quantitative ^{19}F NMR spectroscopy (D_2O).

Table S26. Yields of [B-F] products from reaction between acid-grade fluorspar, H_2SO_4 and $B(OH)_3$

Entry	Acid	Temperature (°C)	Time (h)	HBF_4 (%)	HBF_3OH (%)
1	conc. H_2SO_4	25	48	86	13
2*	conc. H_2SO_4	25	48	75	14
3	conc H_2SO_4	50	24	84	13

* repeat of entry 1

The reaction between acid-grade fluorspar (1.1 equiv), $B(OH)_3$ (0.5 equiv) and H_2SO_4 (1.0 equiv) at 25 °C afforded total [B–F] products in 94% (average of two repeats) after 48 h of stirring.

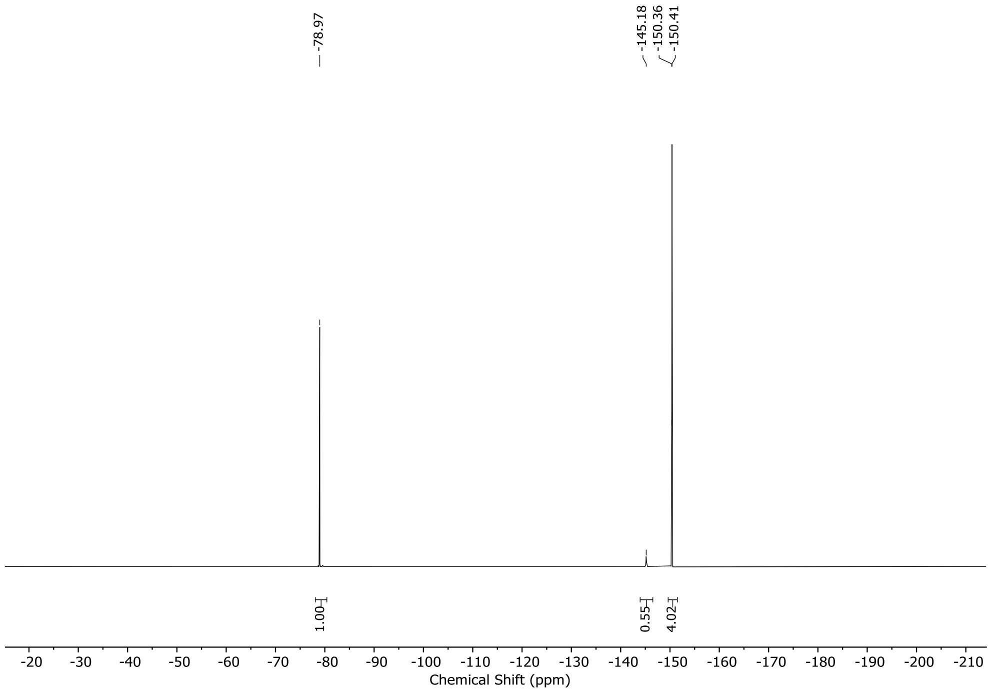

Figure S34. ^{19}F quantitative NMR (D_2O) of crude reaction mixture of acid grade fluorspar (CaF_2, 17.8 mmol), H_2SO_4 (16.2 mmol) and $B(OH)_3$ (8.1 mmol). HBF_4 (-150.4 ppm) and HBF_3OH (-145.2 ppm) produced. 2.02 mmol of NaOTf used as internal standard (-79.0 ppm). ^{19}F NMR Yields of HBF_4 and HBF_3OH are 75% and 14%, respectively.

12. Preparation of KF using sulfuric acid at 50 °C and 25 °C

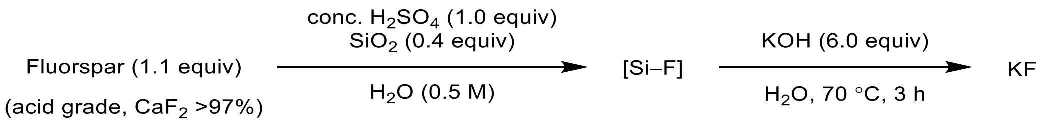

Acid grade fluorspar (644.0 mg, 8.0 mmol, 1.1 equiv), silica gel (306.0 mg, 5.1 mmol, 0.4 equiv) and 95-98% concentrated sulfuric acid (404 µL, 7.3 mmol, 1.0 equiv) were weighed into a 50 mL conical sterile polypropylene (PP) centrifuge tube. H_2O (14.5 mL) was added and the mixture was heated with stirring at either 50 °C or 25 °C for 24 h or 72 h, respectively. The resulting suspension was cooled to ambient temperature, diluted with H_2O (5 mL) and filtered using a Büchner funnel into a 50 mL PP tube. Filtered solids were washed with H_2O to give an aqueous solution (~ 20 mL) containing [Si-F] (H_2SiF_6, and H_2SiF_5OH).

The first step of this reaction affords a theoretical maximum of 2.4 mmol of H_2SiF_6. Accordingly, KOH (85%, 951 mg, 14.4 mmol) was added portion-wise to the aqueous solution (containing H_2SiF_6 and H_2SiF_5OH).

The reaction was heated with stirring at the 70 °C for 3 h. The resulting suspension was filtered using a fritted glass filter to separate insoluble by-products from the solution and washed with water (2 x 5 mL). The filtrate was concentrated *in vacuo* (50 °C) and dried under high vacuum (< 0.1 mbar). Gentle heating (100 °C) of the flask under vacuum for 5 min followed by drying overnight at room temperature under high vacuum provides acid-grade fluorspar derived potassium fluoride (KF^{AGF}) as a white crystalline solid.

KF^{AGF} prepared at 50 °C (24 h) using concentrated H_2SO_4: 817 mg, 14.1 mmol, 98%
Purity of KF^{AGF} by quantitative ^{19}F NMR using NaOTf as an internal standard was calculated to be 46%. This gives a total KF^{AGF} yield of 46%. Purity analysis can be seen below.

KF^{AGF} prepared at 25 °C (72 h) using concentrated H_2SO_4: 830 mg, 14.29 mmol, 99%
Purity of KF^{AGF} by quantitative ^{19}F NMR using NaOTf as an internal standard was calculated to be 36%. This gives a total KF^{AGF} yield of 36%. Purity analysis can be seen below.

Potassium sulfate (K_2SO_4) was identified as a major impurity in the samples of KF prepared at 50 °C (or 25 °C) by PXRD (see below), indicating an incomplete reaction between acid-grade fluorspar, H_2SO_4 and SiO_2.

KF^{AGF} (prepared at 50 °C using H_2SO_4)

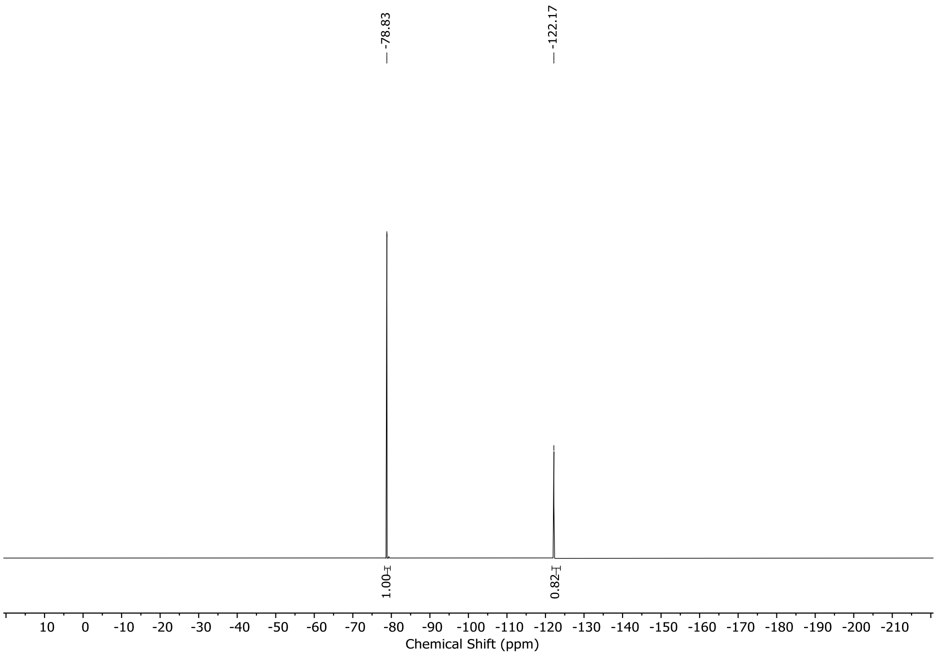

Figure S35. ^{19}F qNMR (D_2O) of KF (prepared using H_2SO_4 at 50 °C) dissolved in D_2O with NaOTf internal standard. KF at -122.17 ppm and NaOTf at -78.80 ppm.

A sample of KF^{AGF} (21.3 mg) and sodium triflate as an NaOTf (12.0 mg) was dissolved in D_2O. The purity of KF in the solid was assessed by quantitative ^{19}F NMR and calculated to be 46%.
^{19}F NMR (377 MHz, D_2O) δ -121.17 (KF, s).

PXRD of KF^{AGF} (prepared at 50 °C using H_2SO_4)

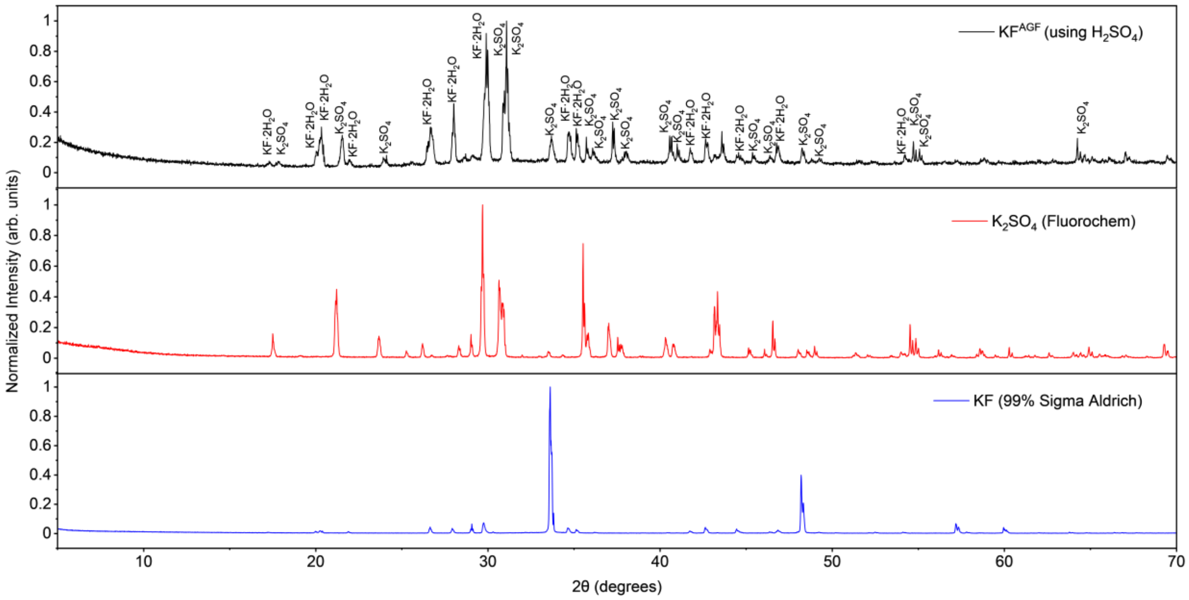

Figure S36. Powder X-ray diffraction patterns of KF formed in the reaction of acid-grade fluorspar (CaF_2) with H_2SO_4 and SiO_2 after 24 h at 50 °C (top), K_2SO_4 (middle) and KF (bottom). Data recorded at room temperature.

KF^{AGF} (prepared at 25 °C using H_2SO_4)

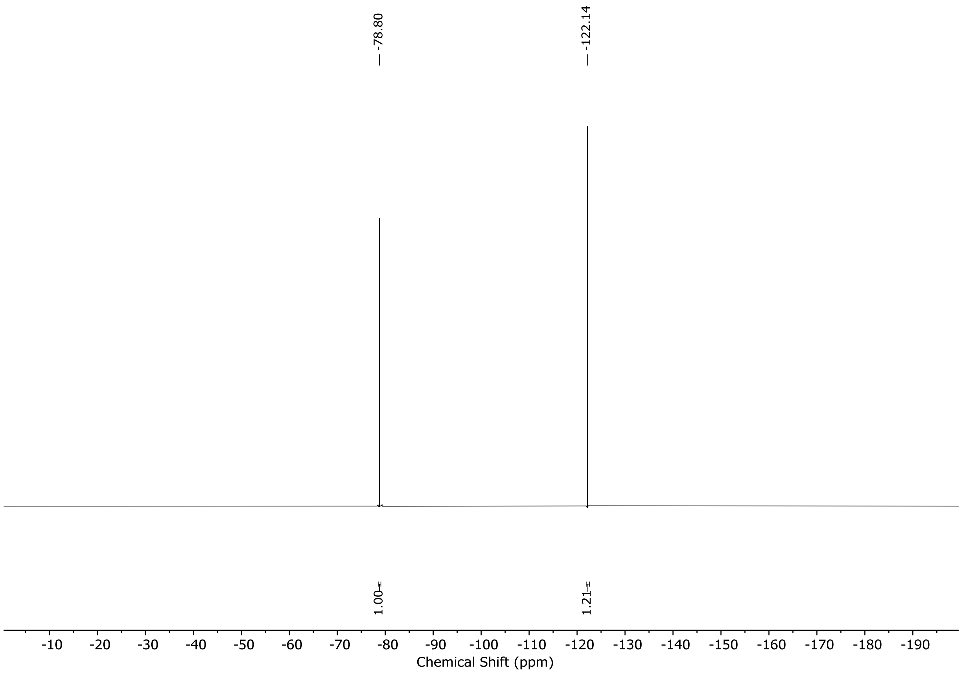

Figure S37. ^{19}F qNMR (D_2O) of KF (prepared using H_2SO_4 at 50 °C) dissolved in D_2O with NaOTf internal standard. KF at -122.17 ppm and NaOTf at -78.80 ppm.

A sample of KF^{AGF} (24.1 mg) and sodium triflate as an NaOTf (7.1 mg) was dissolved in D_2O. The purity of KF in the solid was assessed by quantitative ^{19}F NMR and calculated to be 36%.
^{19}F NMR (377 MHz, D_2O) δ -121.14 (KF, s).

PXRD of KF^{AGF} (prepared at 25 °C using H_2SO_4).

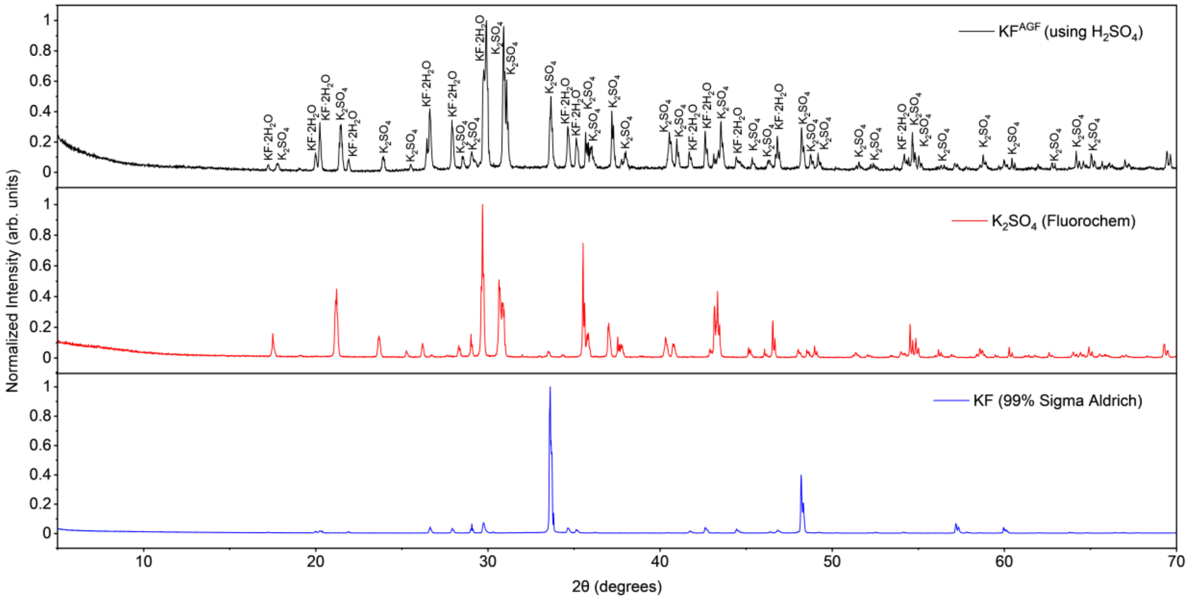

Figure S38. Powder X-ray diffraction patterns of KF formed in the reaction of acid-grade fluorspar (CaF_2) with H_2SO_4 and SiO_2 after 72 h at 25 °C (top), K_2SO_4 (middle) and KF (bottom). Data recorded at room temperature.

12.1. Quantification of [Si-F] species formed (H_2SO_4 as Brønsted acid)

Acid grade fluorspar (644.0 mg, 8.0 mmol, 1.1 equiv), silica gel (306.0 mg, 5.1 mmol, 0.4 equiv) and 95-98% concentrated sulfuric acid (404 µL, 7.3 mmol, 1.0 equiv) were weighed into a 50 mL conical sterile polypropylene (PP) centrifuge tube. H_2O (14.5 mL) was added and the mixture was heated with stirring at either 25 °C or 50 °C for 15 h. NaOTf (42 mg, 0.269 mmol) was added as an internal standard to the reaction mixture. The reaction mixture was stirred for 2 minutes and an aliquot of the reaction was analyzed by quantitative ^{19}F NMR spectroscopy (D_2O) (32 scans, d1 = 30 s, op1 = 103.5 ppm). The total amount of [Si-F] species was quantified using the global spectrum deconvolution (GSD) tool available on MestReNova which enables integration of partially overlapping peaks. This reaction theoretically affords 2.4 mmol of H_2SiF_6 and 2.91 mmol H_2SiF_5OH.

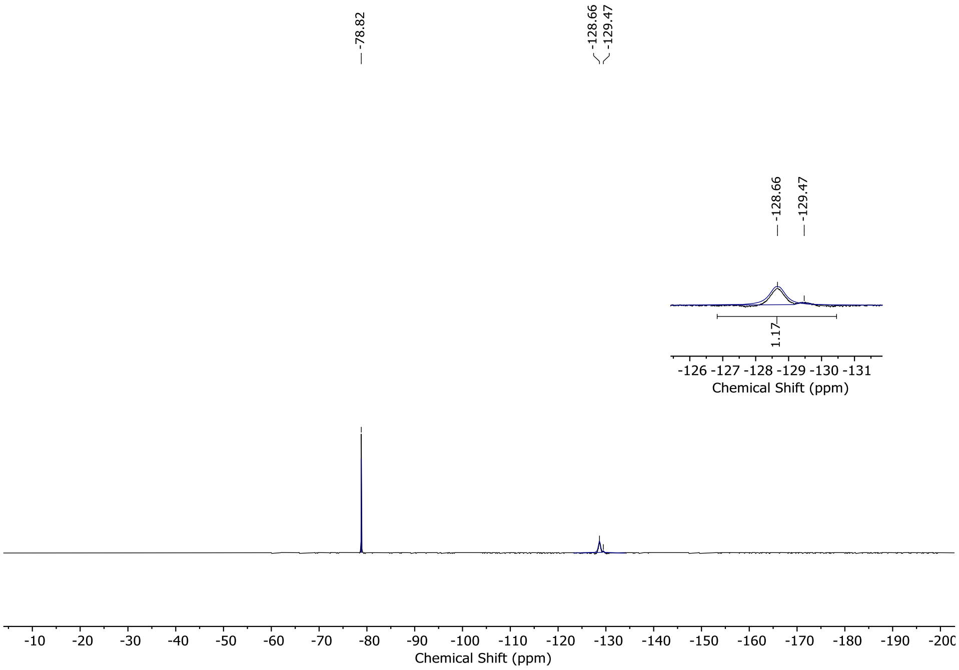

Figure S39.^{19}F NMR (D_2O) spectrum of reaction between acid grade fluorspar (CaF_2), H_2SO_4 and SiO_2 (24 h, 50 °C). Aqueous species H_2SiF_5OH (br s, -128.66 ppm), H_2SiF_6 (br s, -129.47 ppm) observed. Internal standard NaOTf (s, -78.82).

	ppm	Intensity	Width	Area
1	-78.82	284.7	2.94	2396.78
2	-128.66	35.2	207.84	18796.60
3	-129.47	5.4	175.25	1910.53

Figure S40. Global Spectrum Deconvolution (GSD) peak list characterized by chemical shift (ppm), peak intensity, peak width and peak area. Data extracted from the ^{19}F NMR spectrum in **Figure S39**.

Table S27. Calculated [Si–F] % values from total peak area values obtained using GSD. Data extracted from the ^{19}F NMR spectrum in **Figure S39**.

Entry	Compound	Total peak area	Normalized area	[Si-F] %
1	NaOTf	2396.78	1	-
2	H_2SiF_6	1910.53	0.80	4
3	$H_2SiF_5(OH)$	18796.60	7.84	44
			Total [Si-F]	48

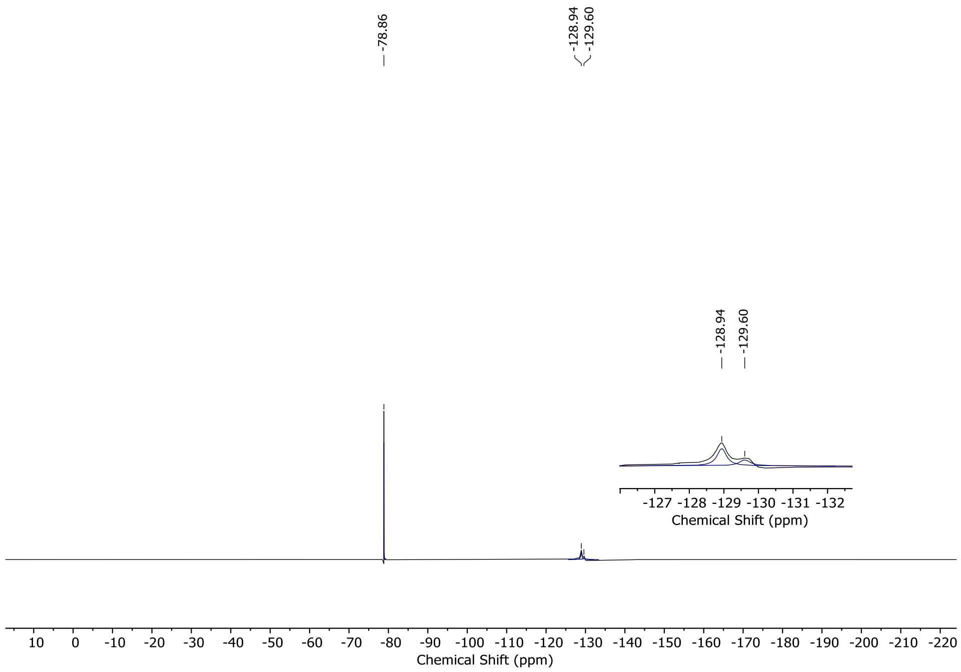

Figure S41.^{19}F NMR (D_2O) spectrum of reaction between acid grade fluorspar (CaF_2), H_2SO_4 and SiO_2 (72 h, 25 °C). Aqueous species H_2SiF_5OH (br s, -128.94 ppm), H_2SiF_6 (br s, -129.60 ppm) observed. Internal standard NaOTf (s, -78.86).

	ppm	Intensity	Width	Area
1	-78.86	3263.2	2.60	15377.56
2	-128.94	200.6	155.19	56397.81
3	-129.60	70.1	186.40	23684.13

Figure S42. Global Spectrum Deconvolution (GSD) peak list characterized by chemical shift (ppm), peak intensity, peak width and peak area. Data extracted from the ^{19}F NMR spectrum in **Figure S40.**

Table S28. Calculated [Si–F] % values from total peak area values obtained using GSD. Data extracted from the ^{19}F NMR spectrum in **Figure S41**.

Entry	Compound	Total peak area	Normalized area	[Si-F] %
1	NaOTf	15377.56	1	-
2	H_2SiF_6	23684.13	1.54	9
3	$H_2SiF_5(OH)$	56397.81	3.67	20
			Total [Si-F]	29

13. Synthesis of Aryldiazonium Tetrafluoroborate Salts

13.1. Safety Statement

CAUTION! **Aryl diazonium tetrafluoroborate salts (ArN_2BF_4)** are considered highly energetic compounds and are presumed to be thermally unstable, sensitive to friction, and shock-prone. It cannot be generalized that these salts are always stable due to the presence of the tetrafluoroborate counter-ion. Potential **detonation** can be caused by heat, friction or shock. In their solid state, some diazonium salts pose a risk of **violent decomposition** and can rapidly release tremendous amounts of destructive energy. Numerous incidents in both industrial and laboratory settings have been attributed to this class of chemicals.

For safety, we assessed the **thermal stability** of each diazonium salt (**S1-S13**), including **DSC analysis, recommended process temperature (T_{D24}), impact sensitivity (IS)** and **explosive propagation (EP)**.

All aryl diazonium tetrafluoroborate salts must be prepared in the presence of a **blast shield**, independent of the protocol applied.

The presence of nitrous acid can lower the decomposition temperature of aryl tetrafluoroborate diazonium salt [42]. During diazotization, minimize the presence of nitrous acid by combining amine and acid first, before adding the *tert*-butyl nitrite. Check for the excess of nitrous acid by starch–potassium iodide paper and neutralization using sulfamic acid.

All diazonium salts were immediately stored in the **freezer (-18 °C)** following isolation, unless mentioned otherwise (see specific procedures in **Section 13** for **S3**, **S4**, **S6**, **S8**, **S9** and **S10**). A **plastic spatula** should be used when handling the aryl diazonium tetrafluoroborate salt and **never a metal spatula**. Aryl diazonium salts can be dried under a stream of N_2 gas but never under reduced pressure or by heating. Residual diazonium compounds in filtrates formed during diazotizations should be analyzed by ^{1}H and ^{19}F NMR spectroscopy. Any **residual diazonium compound should be quenched** using triethylamine. After dediazotization of the diazonium salt, the crude reaction mixture is treated with saturated aqueous $NaHCO_3$ solution to neutralize unreacted diazonium salt.

13.2. Differential Scanning Calorimetry of Aryl Diazonium Tetrafluoroborate Salts

The **thermal stability** of aryl diazonium tetrafluoroborate salts **S1** to **S13** have been determined by DSC.

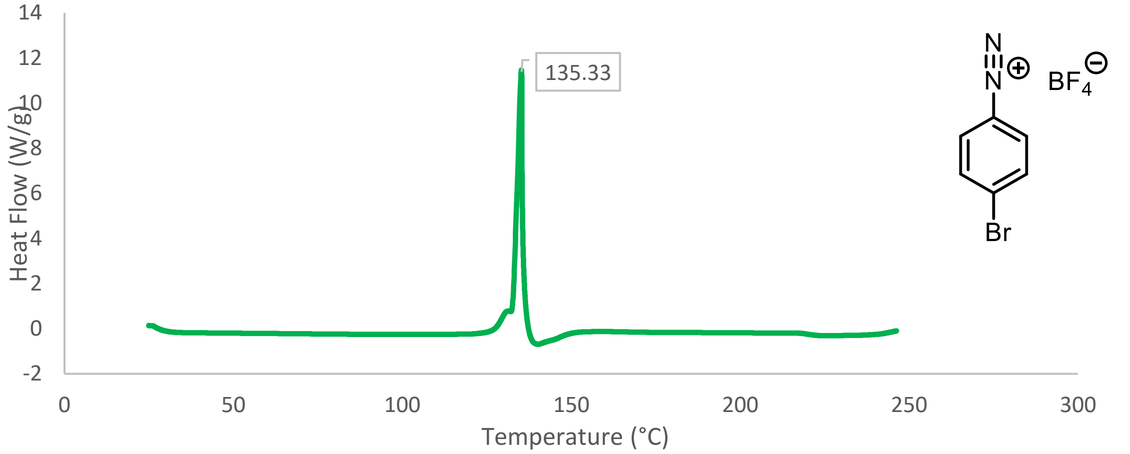

Figure S43. DSC profile of **S1** thermal decomposition under N_2. Sharp exotherm starting at ca. 123 °C consistent with decomposition.

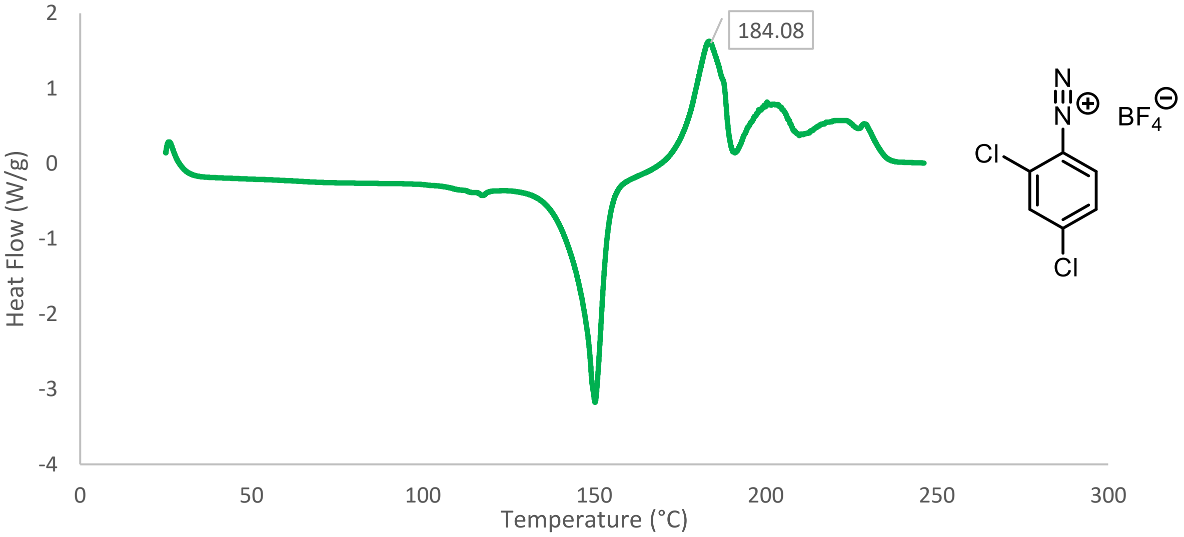

Figure S44. DSC profile of **S2** thermal decomposition under N_2. Melting endotherm followed by decomposition starting at ca. 170 °C over a wider temperature range.

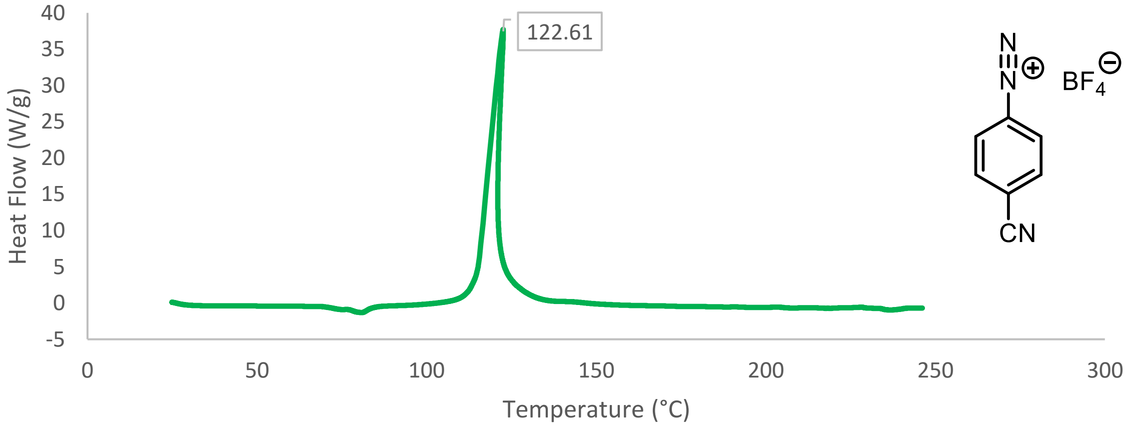

Figure S45. DSC profile of **S3** thermal decomposition under N_2. Sharp exotherm starting at ca. 110 °C consisted with decomposition.

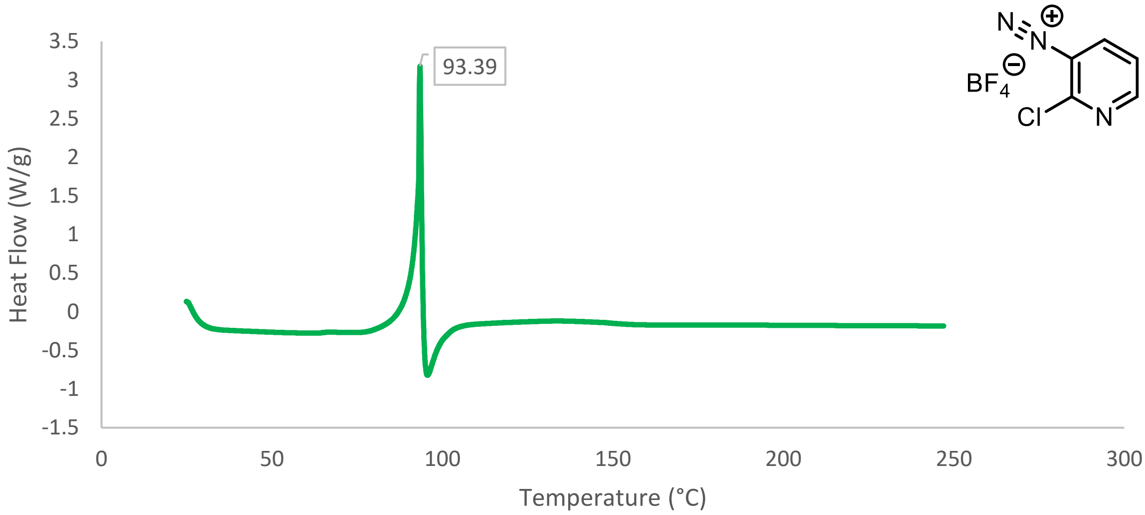

Figure S46. DSC profile of **S4** thermal decomposition under N_2. Sharp exotherm starting at ca. 81 °C consistent with decomposition followed by an endothermic event.

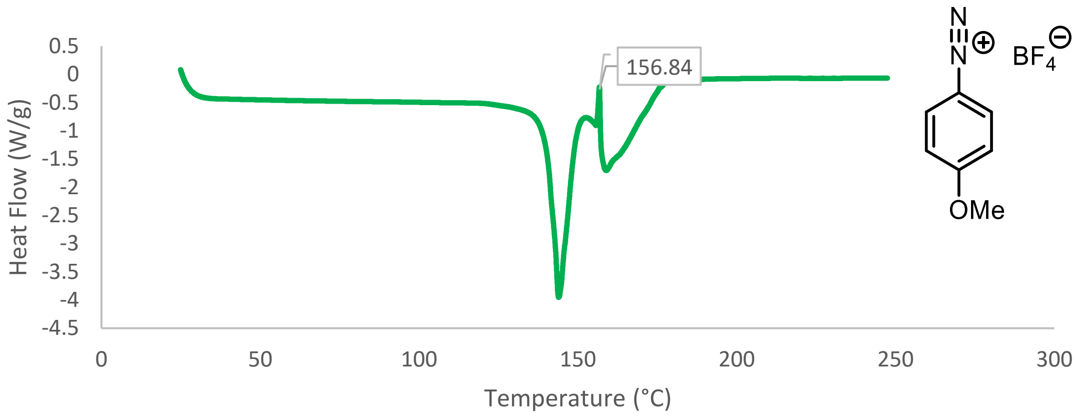

Figure S47. DSC profile of **S5** thermal decomposition under N_2. Melting endotherm followed by an overlapping weak exotherm between 153 °C and 157 °C due to decomposition.

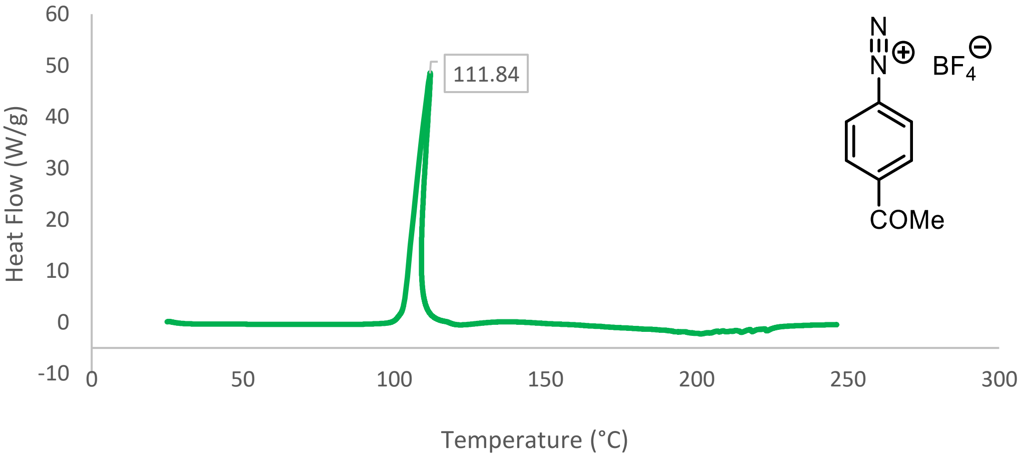

Figure S48. DSC profile of **S6** thermal decomposition under N_2. Sharp exotherm starting at ca. 100 °C consistent with decomposition. No melting endotherm was observed.

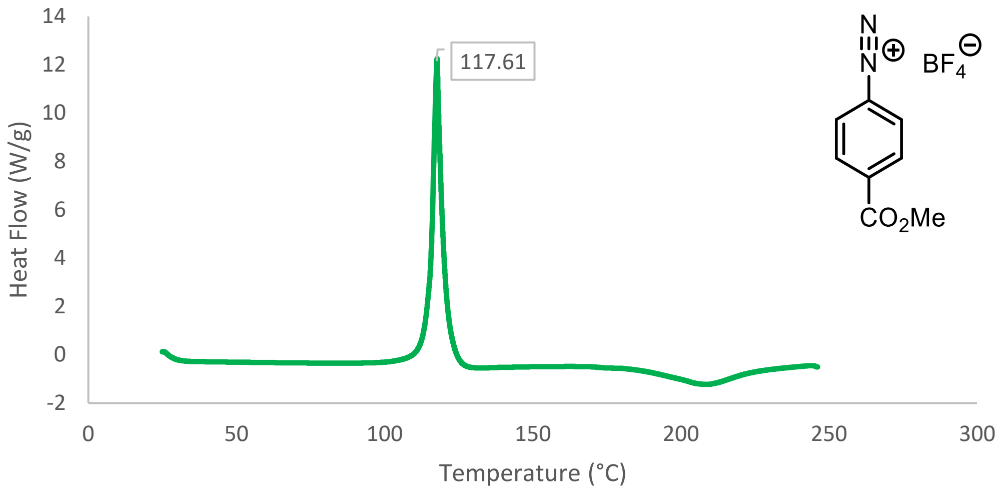

Figure S49. DSC profile of **S7** thermal decomposition under N_2. Sharp exotherm starting at ca. 107 °C consisted with decomposition. No melting endotherm was observed.

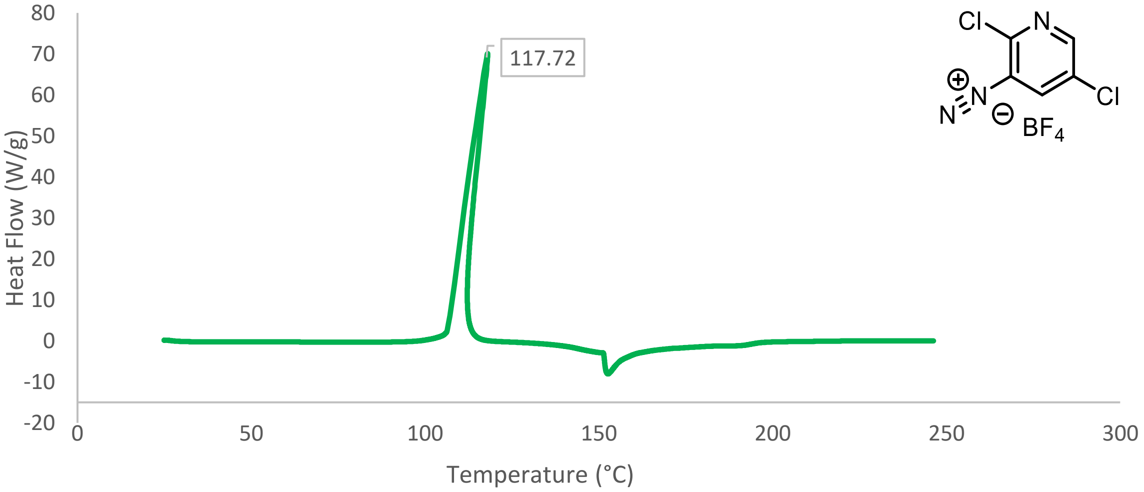

Figure S50. DSC profile of **S8** thermal decomposition under N_2. Sharp exotherm starting at ca. 105 °C consisted with decomposition. However, the exothermic peak in the DSC data is sharp and leans toward higher temperatures, indicative of a thermal runaway event during decomposition.

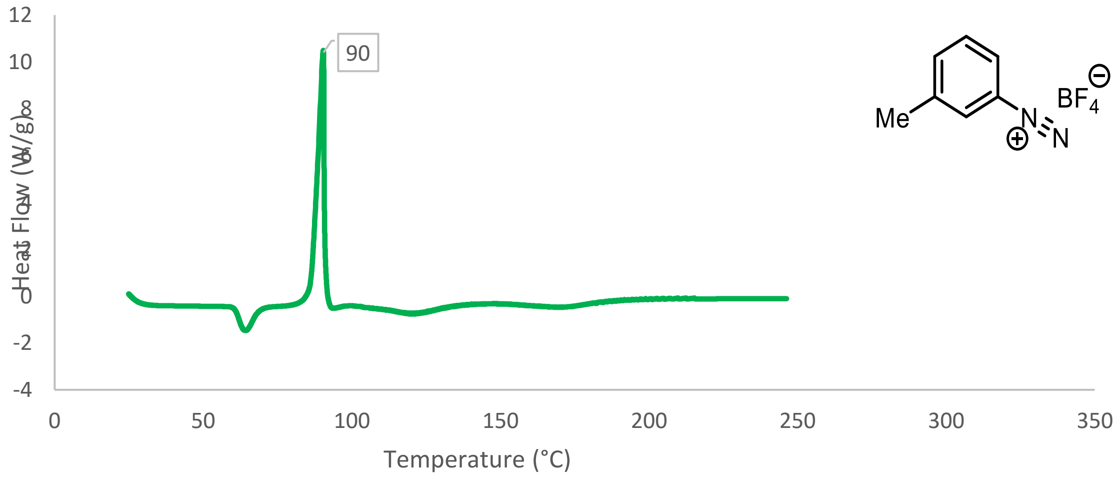

Figure S51. DSC profile of **S9** thermal decomposition under N_2. Melting endotherm followed by a Sharp exotherm starting at ca. 83 °C due to decomposition.

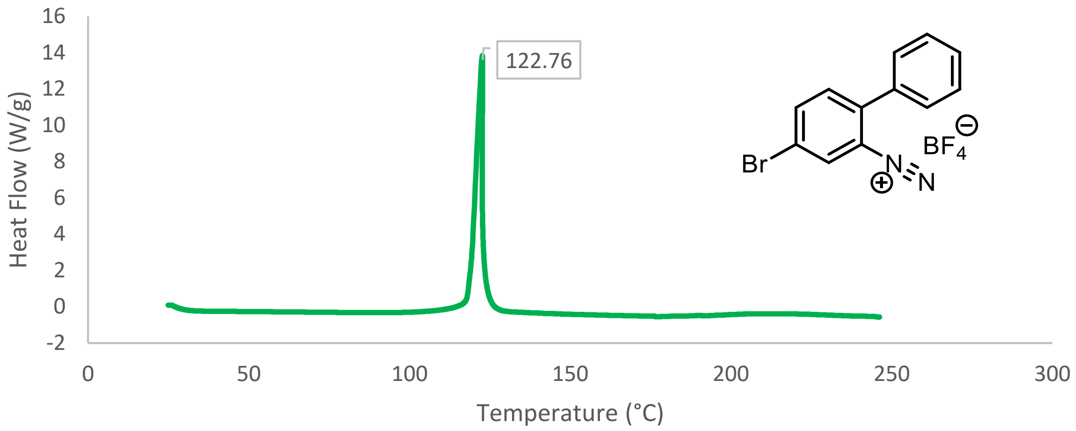

Figure S52. DSC profile of **S10** thermal decomposition under N_2. Sharp exotherm starting at ca. 114 °C due to decomposition. No melting endotherm was observed.

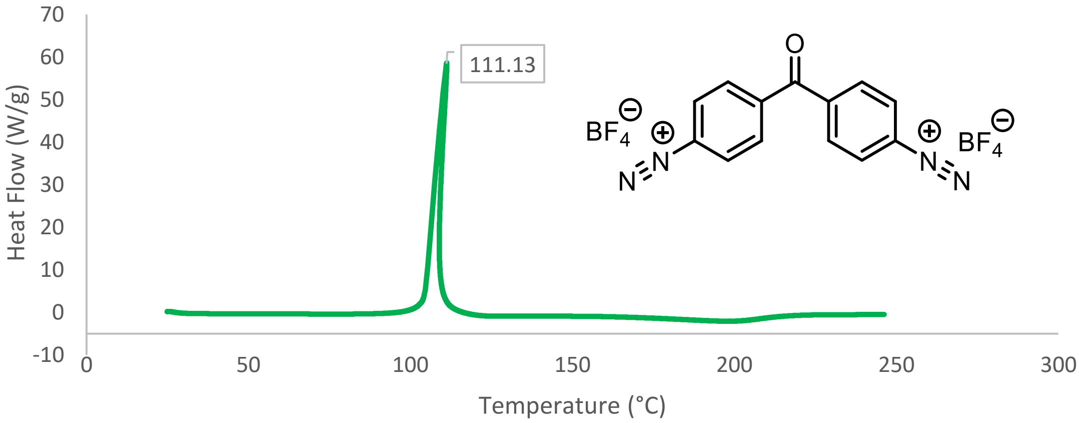

Figure S53. DSC profile of **S11** thermal decomposition under N_2. Sharp exotherm starting at ca. 102 °C due to decomposition. The exothermic peak in the DSC data is sharp and leans toward higher temperatures, indicative of a thermal runaway event during decomposition. No melting endotherm was observed.

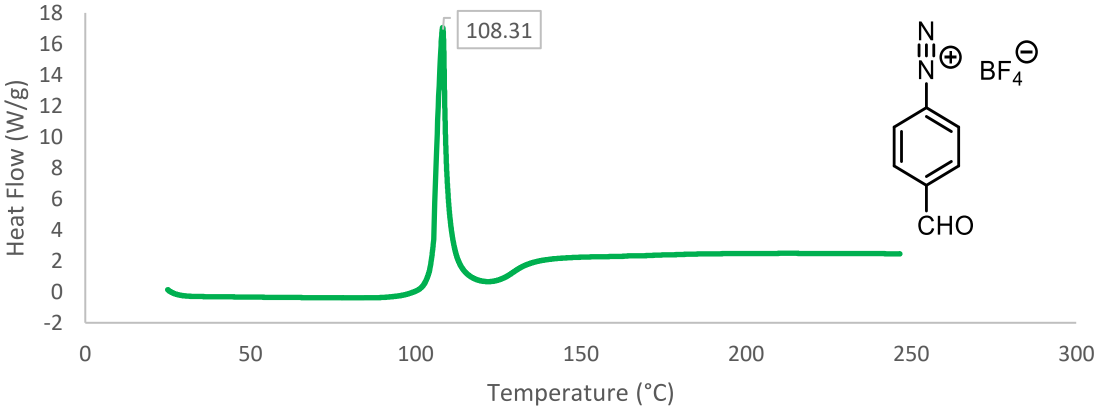

Figure S54. DSC profile of **S12** thermal decomposition under N_2. Sharp exotherm starting at ca. 101 °C due to decomposition. No melting endotherm was observed.

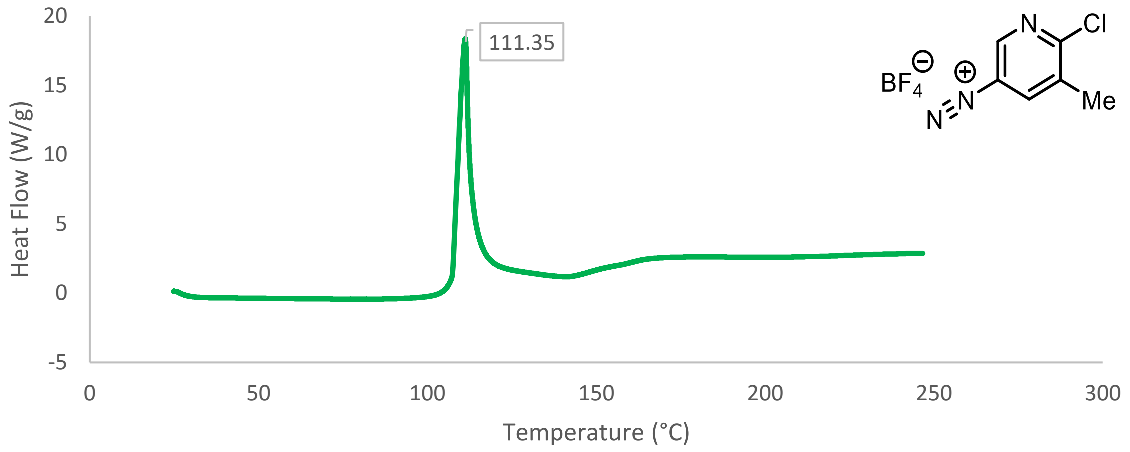

Figure S55. DSC profile of S13 thermal decomposition under N_2. Sharp exotherm starting at ca. 104 °C due to decomposition. No melting endotherm was observed.

13.3. Recommended Process Temperature (T_{D24}) of Aryl Diazonium Tetrafluoroborate Salts

The estimated maximum process temperature (T_{D24}) values for compounds **S1**-**S13**, calculated using T_{init} data (from DSC analysis) and equation (3), are presented in **Table S29** [41]. T_{D24} is defined as the temperature at which the time to maximum rate under adiabatic conditions (TMR_{ad}) becomes >24 h.[41] This calculation provides a quick and conservative estimate of a safe working temperature, above which decomposition may occur.

$$T_{D24} = (0.7 \text{ X } T_{init}) - 46 \quad (3)$$

The diazonium salts showed a thermal decomposition between 100 °C and 150 °C (10 examples), one required higher temperature (**S2**, >150 °C) and two decomposed in the range of 80−90 °C (**S4** and **S9**). Compounds **S4** and **S9** were prepared on a 1 mmol scale only and used immediately after isolation.

Table S29. Differential Scanning Calorimetry (DSC) Analysis and T_{D24} of Diazonium salts **S1**-**S13**.

	Initial decomp. Temp. (T_{init})	T_{D24}	Comments
**S1**	126 °C	42 °C	Sharp exotherm starting at *ca.* 126 °C consistent with decomposition.
**S2**	170 °C	73 °C	Melting endotherm followed by decomposition starting at *ca.* 170 °C and extending over a wide temperature range.
**S3**	104 °C	27 °C	Sharp exotherm starting at *ca.* 104 °C consisted with decomposition.
**S4**	80 °C	10 °C	Sharp exotherm starting at *ca.* 80 °C consistent with decomposition followed by an endothermic event.
**S5**	153 °C	61 °C	Sharp melting endotherm followed by an overlapping exotherm between 153 °C and 157 °C due to decomposition.
**S6**	100 °C	24 °C	Sharp exotherm starting at *ca.* 100 °C consistent with decomposition. No melting endotherm was observed.
**S7**	103 °C	26 °C	Sharp exotherm starting at ca. 103 °C consisted with decomposition. No melting endotherm was observed.
**S8**	103 °C	26 °C	Sharp exotherm starting at *ca.* 103 °C consisted with decomposition. However, the exothermic peak in the DSC data is sharp and leans toward higher temperatures, indicative of a thermal runaway event during decomposition[51].
**S9**	83 °C	12 °C	Melting endotherm followed by a Sharp exotherm starting at *ca.* 83 °C due to decomposition.
**S10**	110 °C	31 °C	Sharp exotherm starting at *ca.* 110 °C due to decomposition. No melting endotherm was observed.
**S11**	101 °C	25 °C	Sharp exotherm starting at *ca.* 101 °C due to decomposition. The exothermic peak is sharp and leans toward higher temperatures, indicative of a thermal runaway event during decomposition. No melting endotherm was observed[51].
**S12**	102 °C	25 °C	Sharp exotherm starting at *ca.* 102 °C due to decomposition. No melting endotherm was observed.
**S13**	104 °C	27 °C	Sharp exotherm starting at *ca.* 104 °C due to decomposition. No melting endotherm was observed.

Diazonium salts **S4** and **S9** have a maximum process temperature (T_{D24}) < 25 °C. A one pot protocol which bypasses the requirement to isolate these diazonium salts has been added to **Section 16** of **Supplementary Information**.

13.4. Impact Sensitivity and Explosive Propagation of Aryl Diazonium Tetrafluoroborate Salts

The mathematical correlations of Yoshida and Pfizer [equations (4 to 7)] have been widely used to predict whether a compound exhibits sensitivity to impact (IS) and/or explosive propagation (EP) [41]. Both correlations employ the values taken from the DSC experiments corresponding to the $T_{initial}$ (◦C), the T_{onset} (◦C), and the enthalpy of decomposition (ΔH_D). The enthalpy values are shown in the form of Q (cal/g) which represents the inverse of ΔH_D in cal/g. The IS and EP values are dimensionless.

$$\text{Yoshida IS: } \log_{10}(Q) - 0.72\,[\log_{10}(T_{onset} - 25)] - 0.98 \quad (4)$$

$$\text{Yoshida EP: } \log_{10}(Q) - 0.38\,[\log_{10}(T_{onset} - 25)] - 1.67 \quad (5)$$

$$\text{Pfizer IS: } \log_{10}(Q) - 0.54\,[\log_{10}(T_{initial} - 25)] - 0.98 \quad (6)$$

$$\text{Pfizer EP: } \log_{10}(Q) - 0.285\,[\log_{10}(T_{initial} - 25)] - 1.67 \quad (7)$$

Full DSC methods can be found in the **Materials and Methods** of **Supplementary Information**. Peak integrations, peak temperatures ($T_{initial}$ and T_{onset}) and enthalpy (Q) were calculated using the TRIOS™ software. Note: $T_{initial}$ vary with the heating rate. In all experiments, a heating rate of 20 °C /min was applied for comparable results and an accurate assessment of thermal stability.

For both correlations, positive values predict that a given compound will be marked as sensitive to impact (IS) and/or as explosive propagators (EP). Compounds may be flagged as hazardous by one correlation and not the other. By default, caution is paramount.

Table S30. Explosion Propagation (EP) and Impact Sensitivity (IS) of **S1-S13** using Yoshida/Pfizer correlations

				Yoshida Correlation		Pfizer Correlation	
**Comp.**	**T_{init}(°C)**	**T_{onset}(°C)**	**Q (Cal/g)**	**Impact sensitivity (IS)**	**Explosive Propagation (EP)**	**Impact Sensitivity (IS)**	**Explosive Propagation (EP)**
**S1**	126	130	39.9	-0.8341	-0.8369	-0.4612	-0.6401
**S2**	170	178	29.4	-1.0847	-1.0319	-0.6788	-0.8177
**S3**	104	123	119.3	-0.3359	-0.3495	**0.0718**	-0.1343
**S4**	80	93	8.1	-1.3913	-1.4574	-1.0099	-1.2561
**S5***	140	148	78.7	-0.5887	-0.5683	-0.1907	-0.3582
**S6**	100	106	119.3	-0.2776	-0.3187	**0.0840**	-0.1279
**S7**	103	103	39.9	-0.7412	-0.7879	-0.4006	-0.6081
**S8**	103	106	133.8	-0.2275	-0.2686	**0.1249**	-0.0827
**S9**	83	87	22.5	-0.9190	-0.9996	-0.5807	-0.8211
**S10**	110	117	29.4	-0.9256	-0.9479	-0.5536	-0.7516
**S11**	101	104	142.0	-0.1941	-0.2389	**0.1565**	-0.0538
**S12**	102	105	48.5	-0.6643	-0.7073	-0.3128	-0.5218
**S13**	104	107	73.4	-0.4924	-0.5317	-0.1392	-0.3453

Compound **S5*** Data is sourced from ref [41]

The Yoshida correlation indicates that none of the tested compounds are either IS or an EP. The Pfizer-modified correlation flagged compounds **S3**, **S6**, **S8**, and **S11** as IS. To ensure safe handling, these compounds are synthesized on a 1 mmol scale. They were then used in the dediazotization step immediately without storage. Alternatively, polyvinyl chloride (PVC) can be added as an inert material (phlegmatizing agent) to stabilize diazonium salts **S3**, **S6**, **S8** and **S11**. The presence of the phlegmatizing agent did not effect the dediazotization process (see **Section 13.7** of **Supplementary Information**).

13.5. General procedure for the synthesis of aryl diazonium tetrafluoroborate (Method A)

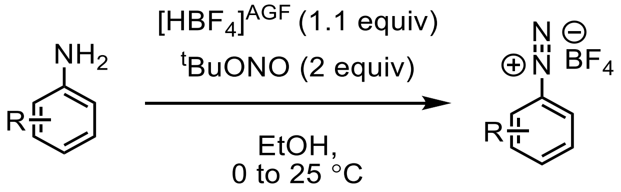

Note, the presence of nitrous acid can lower the decomposition temperature of aryl tetrafluoroborate diazonium salt Minimize the presence of nitrous acid by combining amine and acid first, before adding the tert-butyl nitrite. Check for the excess of nitrous acid by starch–potassium iodide paper and neutralization using sulfamic acid.

In a 25 mL round-bottom flask, the corresponding amine (5 mmol, 1 equiv) was suspended in 5 mL of ethanol. Subsequently, an aqueous solution containing $[HBF_4]^{AGF}$ (0.932 mL, 6 mmol, 5.9 M, 1.1 equiv) was added and the mixture was stirred for 5 minutes at 25 °C. Next, the solution was cooled to 0 °C using an ice bath, and ^{t}BuONO (1.19 mL, 10 mmol, 2 equiv) was added dropwise over a 5-minute period. Immediate formation of a precipitate was observed and reaction was then stirred at 25 °C (or 10 °C using a dry ice dioxane bath) for an additional 1 hour. After the reaction, cold diethyl ether (Et_2O, 20mL) was added to ensure complete precipitation. The precipitate was isolated by filtration and washed with an additional Et_2O (5 mL × 2). The compound obtained was used in the subsequent step without the need for further purification. *Note, after filtration, any remaining diazonium salt in the filtrate (as confirmed by analyzing the filtrate using ^{1}H and ^{19}F NMR) is quenched with triethylamine.*

4-Bromobenzenediazonium tetrafluoroborate (S1)

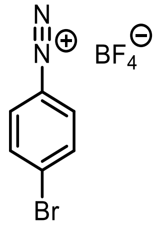

Prepared following **GP A** using 4-bromoaniline (860 mg, 5 mmol). White solid, 93% yield (1.25 g, 4.92 mmol).

^{1}H NMR (200 MHz, DMSO-*d*$_6$) 8.58 (d, *J* = 9.1 Hz, 2H), 8.26 (d, *J* = 9.1 Hz, 2H).
CAS 673-40-5
Spectroscopic data are in accordance with those in literature [52].

2,4-Dichlorobenzenediazonium tetrafluoroborate (S2)

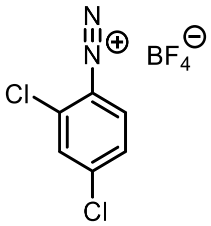

Prepared following **GP A** using 2,4-dichloroaniline (0.810 g, 5 mmol). White solid, 84% yield (1.1 g, 4.22 mmol).

^{1}H NMR (400 MHz, DMSO-*d*$_6$) δ 8.86 (d, *J* = 9.0 Hz, 1H), 8.56 (d, *J* = 2.1 Hz, 1H), 8.11 (dd, *J* = 9.0, 2.1 Hz, 1H).
CAS 27165-13-5
Spectroscopic data are in accordance with those in literature [53].

4-Cyanobenzenediazonium tetrafluoroborate (S3)

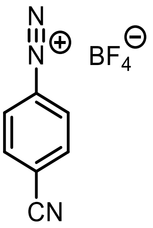

Prepared following **GP A** using 4-aminobenzonitrile (0.118 mg, 1 mmol). Yellow solid, 87% yield (160 mg, 0.737 mmol).

^{1}H NMR (200 MHz, DMSO-*d$_6$*) δ 8.84 (d, *J* = 8.5 Hz, 2H), 8.46 (d, *J* = 8.4 Hz, 2H).
CAS 2252-32-6
Spectroscopic data are in accordance with those in literature [54].

2-Chloropyridine-3-diazoniumtetrafluoroborate (**S4**)

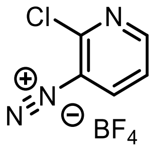

According to the data in **Table S29**, the estimated process temperatures for **S4** indicate low values (T_{D24} < 25 °C) due to their initial decomposition temperatures of 80 °C, respectively. To prepare **S4** safely, the reaction was stirred at 10 °C using a dry ice-dioxane bath instead of at room temperature. **The synthesized diazonium salt S4 was used immediately for fluoro-dediazotisation step.**

Prepared following a modified **GP A** using 2-chloropyridin-3-amine (128.5 mg, 1 mmol). The reaction mixture was initially stirred at 0 °C in an ice bath following the addition of ^{t}BuONO for 5 min, and then transferred to a dry ice-dioxane bath, maintaining a temperature of 10 °C for 1 hour. After the reaction, cold diethyl ether (Et_2O, 5 mL) was added to ensure complete precipitation. The precipitate was isolated by filtration and washed with an additional Et_2O (5 mL × 2). White solid, 60% yield (136 mg, 0.598 mmol).

^{1}H NMR (400 MHz, CD_3CN) δ 9.10 (d, *J* = 4.9 Hz, 1H), 8.98 – 8.87 (m, 1H), 7.94 (dd, *J* = 8.5, 4.9 Hz, 1H).
^{19}F NMR (377 MHz, CD_3CN) δ -151.24.
^{13}C NMR (101 MHz, CD_3CN) δ 161.1, 152.6, 144.7, 126.2, 115.5.

4-Methoxybenzenediazonium tetrafluoroborate (S5)

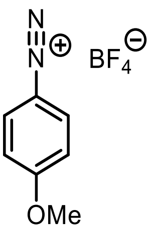

Prepared following **GP A** using 4-methoxyaniline (616 mg, 5 mmol). Brown solid, 93% yield (980 mg, 4.92 mmol).

^{1}H NMR (200 MHz, DMSO-*d$_6$*) δ 8.61 (d, *J* = 9.4 Hz, 2H), 7.48 (d, *J* = 9.4 Hz, 2H), 4.04 (s, 3H).
CAS 459-64-3
Spectroscopic data are in accordance with those in literature [52].

4-Acetylbenzenediazonium tetrafluoroborate (S6)

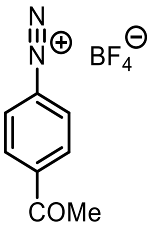

Prepared following **GP A** using 1-(4-aminophenyl)ethan-1-one (135 mg, 1 mmol). White solid, 89% yield (210 mg, 0.897 mmol).

^{1}H NMR (400 MHz, DMSO-*d$_6$*) δ 8.80 (d, *J* = 8.9 Hz, 2H), 8.41 (d, *J* = 8.9 Hz, 2H), 2.71 (s, 3H).
CAS 350-47-0
Spectroscopic data are in accordance with those in literature [55].

4-(Methoxycarbonyl)benzenediazonium tetrafluoroborate (S7)

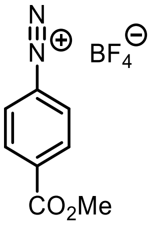

Prepared following **GP A** using methyl 4-aminobenzoate (756 mg, 5 mmol). White solid, 95% yield (1.25 g, 5 mmol).

^{1}H NMR (200 MHz, DMSO-*d$_6$*) δ 8.80 (d, *J* = 8.9 Hz, 2H), 8.44 (d, *J* = 9.0 Hz, 2H), 3.95 (s, 3H).
Spectroscopic data are in accordance with those in literature [56].

2,5-Dichloropyridine-3-diazonium tetrafluoroborate (S8)

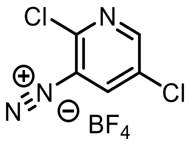

Prepared following **GP A** using 2,5-dichloropyridin-3-amine (163 mg, 1 mmol) and $[HBF_4]^{AGF}$ (2.2 equiv.). The reaction was stirred at 0 °C after addition of tBuONO throughout. Yellow solid, 62% yield (161 mg, 0.614 mmol).

^{1}H NMR (400 MHz, DMSO-*d$_6$*) Complete decomposition observed in DMSO.

3-Methylbenzenediazonium tetrafluoroborate (S9)

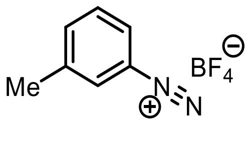

According to the data in **Table S29**, the estimated process temperatures for **S9** indicate low values (T_{D24} < 25 °C) due to their initial decomposition temperatures of 83 °C, respectively. To prepare **S9** safely, the reaction was stirred at 10 °C using a dry ice-dioxane bath instead of at room temperature. **The synthesized diazonium salt S9 was used immediately for fluoro-dediazotisation step.**

Prepared following a modified **GP A** using 3-methylaniline (107 mg, 1 mmol). The reaction mixture was initially stirred at 0 °C in an ice bath following the addition of ^{t}BuONO for 5 min, and then transferred to a dry ice-dioxane bath, maintaining a temperature of 10 °C for 1 hour. After the reaction, cold diethyl ether (Et_2O, 20 mL) was added to ensure complete precipitation. The precipitate was isolated by filtration and washed with an additional Et_2O (5 mL × 2). White solid, 75 % yield (155 mg, 0.752 mmol).

^{1}H NMR (200 MHz, CD_3CN) δ 8.31 (d, *J* = 8.8 Hz, 2H), 8.07 (d, *J* = 7.9 Hz, 1H), 7.80 (t, *J* = 8.0 Hz, 1H), 2.52 (s, 3H).
CAS 1422-76-0
Spectroscopic data are in accordance with those in literature [57].

4-bromo-[1,1'-biphenyl]-2-diazonium tetrafluoroborate (S10)

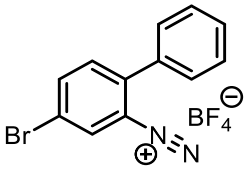

Prepared following **GP A** using 4-bromo-[1,1'-biphenyl]-2-amine (0.3 g, 1.21 mmol) and HBF_4 (1.1 equiv.). Yellow solid, 71% yield (301 mg, 0.868 mmol).

^{1}H NMR (200 MHz, DMSO-*d$_6$*) δ 9.24 (d, *J* = 2.1 Hz, 1H), 8.57 (dd, *J* = 8.5, 2.1 Hz, 1H), 8.02 (d, *J* = 8.5 Hz, 1H), 7.96 – 7.75 (m, 2H), 7.74 – 7.60 (m, 3H).
^{19}F NMR (376 MHz, DMSO-*d$_6$*) δ -148.19 (br s), -148.24 (br s).
^{13}C NMR (101 MHz, DMSO-*d$_6$*) δ 143.9, 143.0, 134.8, 133.6, 132.2, 131.3, 129.9, 128.6, 121.4, 116.2, 40.1, 39.9, 39.7, 39.5, 39.3, 39.1, 38.9.
HRMS (ESI) m/z not found

4,4'-carbonyldibenzenediazonium ditetrafluoroborate (S11)

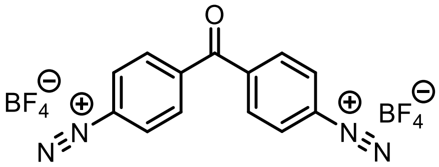

Prepared following **GP A** using bis(4-aminophenyl)methanone (212 mg, 1 mmol), tBuONO (4 equiv.) and $[HBF_4]^{AGF}$ (3.3 equiv.). Brown solid, 95% yield (390 mg, 0.951 mmol).

^{1}H NMR (400 MHz, DMSO-*d$_6$*) Complete decomposition observed in DMSO.

Note: *The Use of excess $[HBF_4]^{AGF}$ is necessary to prevent the formation of mixed diazonium salt species.*

4-Formylbenzenediazonium tetrafluoroborate (S12)

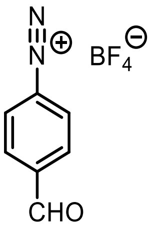

Prepared following modified **GP A.** In a 25 mL round-bottom flask, the 4-aminobenzaldehyde polymer (0.616 mg, 5 mmol) and $[HBF_4]^{AGF}$ (0.932 mL, 6 mmol, 5.9 M, 1.1 equiv) was suspended in 5 ml of ethanol and heated at 60 °C for 15 min to initiate the depolymerisation. Next, the reaction was cooled to 25 °C and additional $[HBF_4]^{AGF}$ (0.932 mL, 6 mmol, 5.9 M, 1.1 equiv) was added. The red solution was cooled to 0 °C using an ice bath and ^{t}BuONO (1.19 mL, 10 mmol, 2 equiv) was then added dropwise over 5 min. The reaction was then stirred at 25 °C for 1 h after which cold ether (Et_2O, 20 mL) was added. The clear solution was kept in the freezer at -25 °C for two days to allow full precipitation of 4-formylbenzenediazonium tetrafluoroborate. White solid, 86% yield (948 mg, 4.31 mmol).

^{1}H NMR (400 MHz, CD_3CN) δ 10.19 (s, 1H), 8.66 (d, *J* = 8.4 Hz, 2H), 8.34 (d, *J* = 8.8 Hz, 2H).
Spectroscopic data are in accordance with those in literature [58]

6-chloro-5-methylpyridine-3-diazonium tetrafluoroborate (S13)

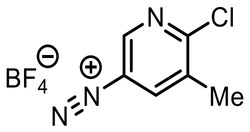

Prepared following **GP A** using 6-chloro-5-methylpyridin-3-amine (713 mg, 5 mmol). Pale orange solid, 73% yield (875 mg, 3.62 mmol).

^{1}H NMR (400 MHz, DMSO) δ 9.42 (d, *J* = 2.9 Hz, 1H), 7.93 (dd, *J* = 2.9, 1.4 Hz, 1H), 2.05 (d, *J* = 1.2 Hz, 3H).
^{19}F NMR (377 MHz, DMSO) δ -148.19 (br s), -148.24 (br s).
^{13}C NMR (101 MHz, DMSO) δ 161.1, 153.5, 130.7, 130.0, 90.2, 16.4.
HRMS (ESI) m/z not found

13.6. Example procedure for the synthesis of aryl diazonium tetrafluoroborate using 1 equivalent of *tert*-butyl nitrite

4-Bromobenzenediazonium tetrafluoroborate (S1)

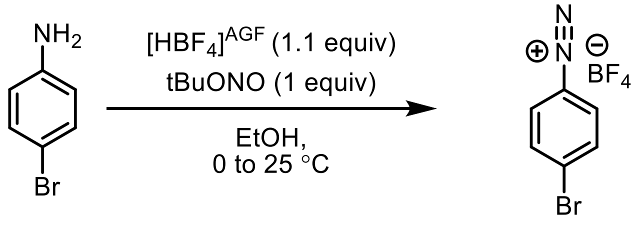

S1

In a 25 mL round-bottom flask, 4-bromoaniline (0.860 g, 5 mmol, 1.0 equiv) was suspended in 5 mL of ethanol. Subsequently, an aqueous solution containing $[HBF_4]^{AGF}$ (1.2 mL, 5.1 mmol, 4.6 M, 1.1 equiv) was added and the mixture was stirred for 5 minutes at 25 °C. Next, the solution was cooled to 0 °C using an ice bath, and *t*BuONO (0.595 mL, 5 mmol, 1 equiv) was added dropwise over a 5-minute period. Immediate formation of a precipitate was observed and reaction was subsequently stirred at 25 °C. After 1 h, cold diethyl ether (Et_2O, 20

mL) was added to ensure complete precipitation. The precipitate was isolated by filtration and washed with an additional Et_2O (2 x 5 mL) obtain **S1** as white solid (951 mg, 3.51 mmol, 71% yield).

13.7. Example procedure for the synthesis of aryl diazonium tetrafluoroborate with the addition of PVC as a phlegmatizing agent.

4,4'-carbonyldibenzenediazonium ditetrafluoroborate (S11) 66.5 wt% with PVC

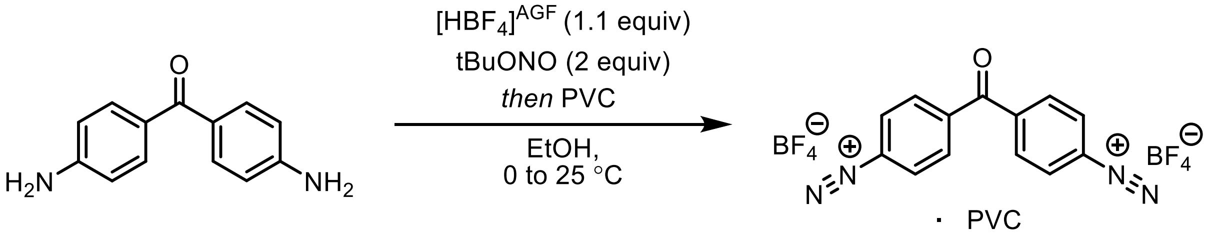

In a 25 mL round-bottom flask, bis(4-aminophenyl)methanone (212 mg, 1.0 mmol, 1 equiv) was suspended in 10 mL of ethanol. Aqueous $[HBF_4]^{AGF}$ (0.660 mL, 3.3 mmol, 5 M, 3.3 equiv) was added and the mixture was stirred for 5 minutes at 25 °C, behind a blast shield. Subsequently, the solution was cooled to 0 °C in an ice bath, and ^{t}BuONO (0.476 mL, 4 mmol, 4 equiv) was added dropwise over a 5-minute period. Immediate formation of a precipitate was observed and reaction was stirred at 25 °C for an additional 1 hour. PVC (1 g, 0.2 g/mmol) was added followed by cold diethyl ether (Et_2O, 10 mL). The precipitate was collected by filtration and washed with an additional cold Et_2O (5 mL × 2). The resulting diazonium tetrafluoroborate stabilized with the phlegmatizing agent was obtained as Brown solid. The total mass of the solid, containing 4,4'-carbonyldibenzenediazonium ditetrafluoroborate (S11) and PVC, was 0.602 g. Based on the known amount of PVC, the yield of S11 was calculated to be 98% (0.976 mmol, 66.6 wt% with PVC).

14. Synthesis of Aryl Fluorides by Balz-Schiemann Reaction

14.1. Safety Statement

CAUTION! **Aryl diazonium tetrafluoroborate salts (ArN_2BF_4)** are considered highly energetic compounds and are presumed to be thermally unstable, sensitive to friction, and shock-prone. It cannot be generalized that these salts are always stable due to the presence of the tetrafluoroborate counter-ion. All dediazotization reactions should be conducted in the presence of a **blast shield**. A **plastic spatula** should be used when handling the aryl diazonium tetrafluoroborate salt and never a metal spatula. After dediazotization of the diazonium salt, the crude reaction mixture is treated with saturated aqueous $NaHCO_3$ solution to neutralize unreacted diazonium salt.

14.2. General procedure for the dediazotization (Method B)

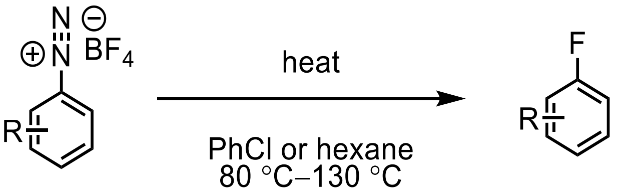

Under an air atmosphere, a screwcap sealed tube was charged with aryldiazonium tetrafluoroborate (0.5 mmol) and PhCl or hexane as solvent. The mixture was heated to specified temperature for specified time, after which the lid was unscrewed and white smoke emerged the vial. The mixture was diluted with Et_2O (10 ml) and quenched with saturated aqueous $NaHCO_3$ (2 mL). Organic layer was separated and the aqueous phase was extracted twice with Et_2O (2 x 5 mL). The combined organic fractions were dried over anhydrous Na_2SO_4, filtered, and concentrated *in vacuo*. The residue was purified by column chromatography on silica to afford the title compound.

Remove the solvent at 700 mbar and 40°C or at 100 mbar and 0 °C, *depending on the volatility of the product.*

Safety note: Dediazotization leads to the formation of BF_3 gas which increases the internal pressure of the reaction tube. After the reaction is finished the unscrewing must be performed inside the fume hood to safely release the BF_3 gas.

1-Bromo-4-fluorobenzene (1)

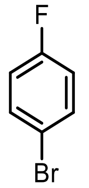

Prepared using 4-bromobenzenediazonium tetrafluoroborate **S1** (135 mg, 0.5 mmol) in PhCl (2 mL) following procedure **GP B**. The mixture was heated at 90 °C for 16 h, after which the lid was unscrewed and four drops of H_2O was added.

Yield: 98% (determined by ^{19}F NMR against 4-fluoroanisole as an internal standard).
^{19}F NMR (377 MHz, $CDCl_3$) δ, -114.97.
CAS 460-00-4
Spectroscopic data are in accordance with those in literature [59].

2,4-Dichloro-1-fluorobenzene (2)

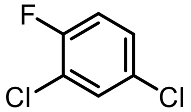

Under an air atmosphere, a screwcap sealed tube was charged with 2,4-dichlorobenzenediazonium tetrafluoroborate **S2** (260 mg, 1 mmol). The solid was heated at 190 °C for 2 h until all the solid melted, after which the lid was unscrewed and white smoke emerged the vial. The mixture was diluted with Et_2O (10 ml) and quenched with saturated aqueous $NaHCO_3$ (2 mL). Organic layer was separated and the aqueous phase was extracted twice with Et_2O (2 x 5 mL). The combined organic fractions were dried over anhydrous Na_2SO_4, filtered, and concentrated under reduced pressure. The residue was purified by column chromatography on silica gel using pentane as eluent to afford the title compound as colorless oil.

Note: Negligible decomposition (<10%) of 2,4-Dichlorobenzenediazonium tetrafluoroborate was observed at 80-100 °C either in hexane or PhCl or neat.

Yield: 120 mg, 0.73 mmol, 73%.
^{1}H NMR (400 MHz, $CDCl_3$) δ 7.40 (dd, *J* = 6.4, 2.5 Hz, 1H), 7.21 (ddd, *J* = 8.8, 4.1, 2.5 Hz, 1H), 7.10 – 7.06 (m, 1H).
^{13}C NMR (101 MHz, $CDCl_3$) δ 157.1 (d, *J* = 248.9 Hz), 130.5, 129.8 (d, *J* = 3.8 Hz), 128.3 (d, *J* = 7.1 Hz), 122.1 (d, *J* = 19.2 Hz), 117.6 (d, *J* = 22.6 Hz).
^{19}F NMR (377 MHz, $CDCl_3$) δ -117.75.
CAS 1435-48-9
Spectroscopic data are in accordance with those in literature [60].

4-Fluorobenzonitrile (3)

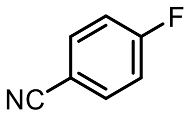

Under an air atmosphere, a screwcap sealed tube was charged with 4-cyanobenzenediazonium tetrafluoroborate **S3** (216 mg, 1 mmol) and hexane (4 mL). The mixture was heated at 80 °C for 16 h, after which the lid was unscrewed and white smoke emerged the vial. The mixture was diluted with Et_2O (10 ml) and quenched with saturated aqueous $NaHCO_3$ (2 mL). Organic layer was separated and the aqueous phase was extracted twice with Et_2O (2 x 5 mL). The combined organic fractions were dried over anhydrous Na_2SO_4, filtered, and concentrated under reduced pressure. The residue was purified by column chromatography on silica gel using pentane/Et_2O (95:5) as eluent to afford the title compound as white solid.

Note: Remove the solvent at 100 mbar and 0 °C, *due to the sublimation of the product.*

Yield: 94 mg, 0.78 mmol, 78%.
^{1}H NMR (400 MHz, $CDCl_3$) δ 7.70 – 7.63 (m, 2H), 7.21 – 7.12 (m, 2H).
^{13}C NMR (101 MHz, $CDCl_3$) δ 165.1 (d, *J* = 256.6 Hz), 134.8 (d, *J* = 9.4 Hz), 118.1, 116.9 (d, *J* = 22.6 Hz), 108.6 (d, *J* = 3.6 Hz).
^{19}F NMR (376 MHz, $CDCl_3$) δ -102.45.
CAS 1194-02-1
Spectroscopic data are in accordance with those in literature [61].

2-Chloro-3-fluoropyridine (4)

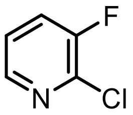

Under an air atmosphere, a screwcap sealed tube was charged with 2-chloropyridine-3-diazoniumtetrafluoroborate **S4** (227 mg, 1 mmol) and hexane (4 mL). The mixture was heated at 80 °C for 16 h, after which the lid was unscrewed and white smoke emerged the vial. The mixture was diluted with Et_2O (10 ml) and quenched with saturated aqueous $NaHCO_3$ (2 mL). Organic layer was separated and the aqueous phase was extracted twice with Et_2O (2 x 5 mL). The combined organic fractions were dried over anhydrous Na_2SO_4, filtered, and concentrated under reduced pressure. The residue was purified by column chromatography on silica gel using pentane as eluent to afford the title compound as colorless liquid.

Note: Solvent was removed at 100 mbar and 0 °C, *due to the volatility of the product.*

Yield: 114 mg, 0.87 mmol, 87%.
^{1}H NMR (400 MHz, $CDCl_3$) δ 8.27 – 8.18 (m, 1H), 7.54 – 7.43 (m, 1H), 7.34 – 7.22 (m, 1H).
^{13}C NMR (101 MHz, $CDCl_3$) δ 155.0 (d, *J* = 260.8 Hz), 144.9 (d, *J* = 5.9 Hz), 139.2 (d, *J* = 22.3 Hz), 124.6 (d, *J* = 18.7 Hz), 123.8 (d, *J* = 2.9 Hz).
^{19}F NMR (377 MHz, $CDCl_3$) δ -118.27.
CAS 17282-04-1
Spectroscopic data are in accordance with those in literature [62].

4-Fluoroanisole (5)

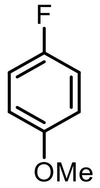

Under an air atmosphere, screwcap sealed tube was charged with 4-methoxybenzenediazonium tetrafluoroborate **S5** (221 mg, 1 mmol). The solid was slowly heated from 100 °C to 130 °C for 20 min, after which the lid was unscrewed and white smoke emerged the vial. The mixture was diluted with Et_2O (10 ml) and quenched with saturated aqueous $NaHCO_3$ (2 mL). Organic layer was separated and the aqueous phase was extracted twice with Et_2O. The combined organic fractions were dried over anhydrous Na_2SO_4, filtered, and concentrated under reduced pressure at 0 °C using a rotatory evaporator. The residue was purified by column chromatography on silica gel using pentane as eluent to afford the title compound as yellow oil.

Note: After the column chromatography solvent was removed at 100 mbar and 0 °C *due to the volatility of the product.*

Yield: 79 mg, 0.63 mmol, 63%.
^{1}H NMR (400 MHz, CDCl$_3$) δ 6.98 (dd, *J* = 9.2, 8.2 Hz, 2H), 6.84 (dd, *J* = 9.2, 4.3 Hz, 2H), 3.78 (s, 3H).
^{13}C NMR (101 MHz, CDCl$_3$) δ 157.4 (d, *J* = 237.8 Hz), 155.8 (d, *J* = 2.0 Hz), 115.9 (d, *J* = 23.1 Hz), 114.9 (d, *J* = 8.0 Hz), 55.9.
^{19}F NMR (377 MHz, CDCl$_3$) δ -124.39.
CAS 459-60-9
Spectroscopic data are in accordance with those in literature [63].

1-(4-fluorophenyl)ethan-1-one (6)

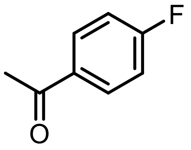

Under an air atmosphere, a screwcap sealed tube was charged with 4-acetylbenzenediazonium tetrafluoroborate **S6** (234 mg, 1 mmol) and PhCl (4 mL). The mixture was heated at 110 °C temperature for 4 h, after which the lid was unscrewed and white smoke emerged the vial. The mixture was diluted with Et_2O (10 ml) and quenched with saturated aqueous $NaHCO_3$ (2 mL). Organic layer was separated and the aqueous phase was extracted twice with Et_2O (2 x 5 mL). The combined organic fractions were dried over anhydrous Na_2SO_4, filtered, and concentrated under reduced pressure. The residue was purified by column chromatography on silica gel using pentane/Et_2O (90:10) as eluent to afford the title compound as yellow oil.

Yield: 91 mg, 0.66 mmol, 66%.
^{1}H NMR (400 MHz, CDCl$_3$) δ 7.98 (dd, *J* = 8.9, 5.4 Hz, 2H), 7.13 (t, *J* = 8.6 Hz, 2H), 2.59 (s, 3H).
^{13}C NMR (126 MHz, CDCl$_3$) δ 196.6, 165.9 (d, *J* = 254.5 Hz), 133.7 (d, *J* = 3.1 Hz), 131.1 (d, *J* = 9.2 Hz), 115.8 (d, *J* = 22.0 Hz), 26.7.
^{19}F NMR (377 MHz, CDCl$_3$) δ -105.33.
CAS 403-42-9
Spectroscopic data are in accordance with those in literature [61].

Methyl 4-fluorobenzoate (7)

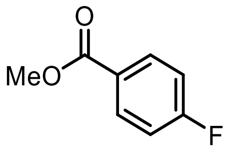

Under an air atmosphere, a screwcap sealed tube was charged with 4-(methoxycarbonyl)benzenediazonium tetrafluoroborate **S7** (250 mg, 1 mmol) and hexane (4 mL). The mixture was heated at 80 °C for 16h, after which the lid was unscrewed and white smoke emerged the vial. The mixture was diluted with Et_2O (10 ml) and quenched with saturated aqueous $NaHCO_3$ (2 mL). Organic layer was separated and the aqueous phase was extracted twice with Et_2O (2 x 5 mL). The combined organic fractions were dried over anhydrous Na_2SO_4, filtered, and concentrated under reduced pressure. The residue was purified by column chromatography on silica gel using pentane as eluent to afford the title compound.

Note: Remove the solvent at 100 mbar and 0 °C, *due to the volatility of the product.*

Yield: 131 mg, 0.5 mmol, 85%
^{1}H NMR (400 MHz, $CDCl_3$) δ 8.10 – 8.00 (m, 2H), 7.16 – 7.05 (m, 2H), 3.91 (s, 3H).
^{13}C NMR (101 MHz, $CDCl_3$) δ 167.2, 165.5 (d, *J* = 166.4 Hz), 132.3 (d, *J* = 9.4 Hz), 126.6 (d, *J* = 3.0 Hz), 115.7 (d, *J* = 21.9 Hz), 52.3.
^{19}F NMR (377 MHz, $CDCl_3$) δ -105.82.
CAS 403-33-8
Spectroscopic data are in accordance with those in literature [64].

2,5-Dichloro-3-fluoropyridine (8)

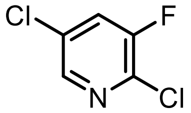

Under an air atmosphere, a screwcap sealed tube was charged with 2,5-dichloropyridine-3-diazonium tetrafluoroborate **S8** (250 mg, 1 mmol) and hexane (4 mL). The mixture was heated at 80 °C for 3 h, after which the lid was unscrewed and white smoke emerged the vial. The mixture was diluted with Et_2O (10 ml) and quenched with saturated aqueous $NaHCO_3$ (2 mL). Organic layer was separated and the aqueous phase was extracted twice with Et_2O (2 x 5 mL). The combined organic fractions were dried over anhydrous Na_2SO_4, filtered, and concentrated under reduced pressure. The residue was purified by column chromatography on silica gel using pentane as eluent to afford the title compound as white solid.

Note: Solvent was removed at 100 mbar and 0 °C, *due to the sublime nature of the product.*

Yield: 125 mg, 75%.
^{1}H NMR (500 MHz, $CDCl_3$) δ 8.22 (d, *J* = 2.2 Hz, 1H), 7.53 (dd, *J* = 7.5, 2.2 Hz, 1H).
^{13}C NMR (126 MHz, $CDCl_3$) δ 154.4 (d, *J* = 266.7 Hz), 143.8 (d, *J* = 5.6 Hz), 137.5 (d, *J* = 19.3 Hz), 131.3 (d, *J* = 2.5 Hz), 125.0 (d, *J* = 21.2 Hz).
^{19}F NMR (470 MHz, $CDCl_3$) δ -115.49.
CAS 103999-77-5
Spectroscopic data are in accordance with those in literature [65].

1-Fluoro-3-methylbenzene (9)

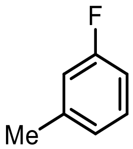

Prepared using 3-methylbenzenediazonium tetrafluoroborate **S9** (103 mg, 0.5 mmol) in hexane (2 mL). The mixture was heated at 80 °C for 1 h, after which the lid was unscrewed and four drops of H_2O was added.

Yield: 89% (determined by ^{19}F NMR against 4-fluoroanisole as an internal standard).
^{19}F NMR (377 MHz, $CDCl_3$) δ, -114.35.
CAS 352-70-5
Spectroscopic data are in accordance with those in literature [66].

4-Bromo-2-fluoro-1,1'-biphenyl (10)

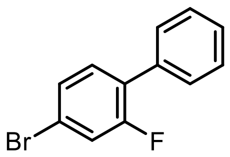

Under an air atmosphere, a screwcap sealed tube was charged with 4-bromo-[1,1'-biphenyl]-2-diazonium tetrafluoroborate **S10** (150 mg, 0.44 mmol) and hexane (2 mL). The mixture was heated at 90 °C for 1 h, after which the lid was unscrewed and white smoke emerged the vial. The mixture was diluted with Et_2O (10 ml) and quenched with saturated aqueous $NaHCO_3$ (2 mL). Organic layer was separated and the aqueous phase was extracted twice with Et_2O (5 mL× 2). The combined organic fractions were dried over anhydrous Na_2SO_4, filtered, and concentrated under reduced pressure. The residue was purified by column chromatography on silica gel using pentane as eluent to afford the title compound as a white solid.

Yield: 90 mg, 0.83 mmol, 83%.
^{1}H NMR (400 MHz, $CDCl_3$) δ 7.95 – 6.83 (m, 8H).
^{13}C NMR (101 MHz, $CDCl_3$) δ 159.5 (d, J = 252.5 Hz), 134.8 (d, J = 1.5 Hz), 131.8 (d, J = 4.0 Hz), 128.9 (d, J = 3.1 Hz), 128.6, 128.2 (d, J = 13.4 Hz), 128.1, 127.7 (d, J = 3.7 Hz), 121.3 (d, J = 9.5 Hz), 119.8 (d, J = 26.2 Hz).
^{19}F NMR (377 MHz, $CDCl_3$) δ -115.13.
CAS 41604-19-7
Spectroscopic data are in accordance with those in literature [67].

Bis(4-fluorophenyl)methanone (11)

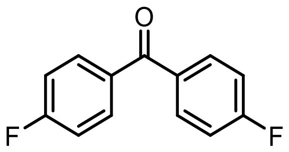

Under an air atmosphere, a screwcap sealed tube was charged with 4,4'-carbonyldibenzenediazonium ditetrafluoroborate **S11** (410 mg, 1 mmol) and PhCl (8 mL). The mixture was heated at 80 °C for 16 h, after which the lid was unscrewed and white smoke emerged the vial. The mixture was diluted with Et_2O (10 ml) and quenched with saturated aqueous $NaHCO_3$ (4 mL). Organic layer was separated and the aqueous phase was extracted twice with Et_2O (10 mL× 2). The combined organic fractions were dried over anhydrous Na_2SO_4, filtered, and concentrated under reduced pressure. The residue was purified by column chromatography on silica gel using pentane/Et_2O (90:10) as eluent to afford the title compound as white solid.

Heating **S11** in hexane at 80 °C for 16 h resulted in a 60% yield of bis(4-fluorophenyl)methanone.

Yield: 151 mg, 0.62 mmol, 69%.
^{1}H NMR (500 MHz, $CDCl_3$) δ 7.81 (m, 4H), 7.17 (m, 4H).
^{13}C NMR (126 MHz, $CDCl_3$) δ 194.0, 165.5 (d, J = 254.3 Hz), 133.8 (d, J = 3.2 Hz), 132.6 (d, J = 9.1 Hz), 115.7 (d, J = 21.8 Hz).
^{19}F NMR (470 MHz, $CDCl_3$) δ -105.76.
CAS 345-92-6
Spectroscopic data are in accordance with those in literature [68].

4-Fluorobenzaldehyde (12)

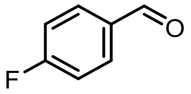

Under an air atmosphere, screwcap sealed tube was charged with 4-formylbenzenediazonium tetrafluoroborate **S12** (220 mg, 1 mmol) and hexane (4 mL). The mixture was heated 80 °C for 2 h, after which the lid was unscrewed and white smoke emerged the vial. The mixture was diluted with Et_2O (10 ml) and quenched with saturated aqueous $NaHCO_3$ (2 mL). Organic layer was separated and the aqueous phase was extracted twice with Et_2O. The combined organic fractions were dried over anhydrous Na_2SO_4, filtered, and concentrated under reduced pressure at 0 °C using a rotatory evaporator. The residue was purified by column chromatography on silica using pentane/Et_2O (98:2) as eluent to afford the title compound as yellow oil.

Note: After the column chromatography solvent was removed at 100 mbar and 0 °C *due to the volatility of the product.*

Yield: 89 mg, 0.72 mmol, 72%.
^{1}H NMR (400 MHz, CDCl$_3$) δ 9.95 (s, 1H), 7.90 (dd, *J* = 8.2, 5.4 Hz, 2H), 7.20 (dd, *J* = 9.2, 8.1 Hz, 2H).
^{13}C NMR (101 MHz, CDCl$_3$) δ 190.6, 166.6 (d, *J* = 256.7 Hz), 133.1 (d, *J* = 2.7 Hz), 132.3 (d, *J* = 9.8 Hz), 116.5 (d, *J* = 22.3 Hz).
^{19}F NMR (377 MHz, CDCl$_3$) δ, -102.41.
CAS 459-57-4
Spectroscopic data are in accordance with those in literature [63].

2-chloro-5-fluoro-3-methylpyridine (13)

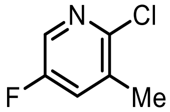

Under an air atmosphere, a screwcap sealed tube was charged with 6-chloro-5-methylpyridine-3-diazonium tetrafluoroborate **S13** (241 mg, 1 mmol) and hexane (4 mL). The mixture was heated at 95 °C for 30 min, after which the lid was unscrewed and white smoke emerged the vial. The mixture was diluted with Et_2O (10 ml) and quenched with saturated aqueous $NaHCO_3$ (2 mL). Organic layer was separated and the aqueous phase was extracted twice with Et_2O (2 x 5 mL). The combined organic fractions were dried over anhydrous Na_2SO_4, filtered, and concentrated under reduced pressure. The residue was purified by column chromatography on silica gel using pentane/EtOAc (99:1) as eluent to afford the title compound as a white solid.

Yield: 119 mg, 0.82 mmol, 82%.
^{1}H NMR (400 MHz, CDCl$_3$) δ 8.11 (d, *J* = 2.7 Hz, 1H), 7.32 (dd, *J* = 8.3, 2.7 Hz, 1H), 2.39 (s, 3H).
^{13}C NMR (101 MHz, CDCl$_3$) δ 160.0, 157.5, 146.3, 146.3, 135.0, 134.7, 134.4, 134.3, 126.6, 126.4, 19.7.
^{19}F NMR (377 MHz, CDCl$_3$) δ -130.36 (d, *J* = 8.3 Hz).
CAS 38186-84-4
Spectroscopic data are in accordance with those in literature [69].

14.3. Example procedure for the dediazotization of aryl diazonium tetrafluoroborate in the presence of PVC as a phlegmatizing agent.

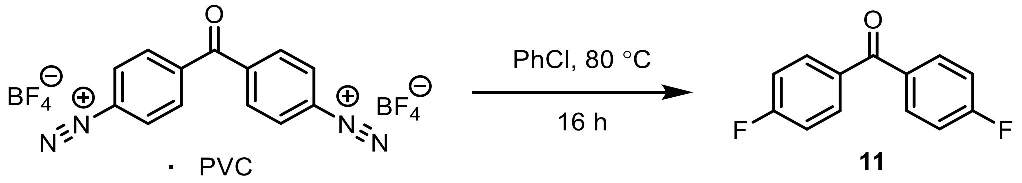

11

Under an air atmosphere, a screwcap sealed tube was charged with aryldiazonium tetrafluoroborate (313 mg, 0.5 mmol, 65.5 wt% in PVC) and PhCl (4 mL) as solvent. The mixture was heated at 80 °C for 16 h, after which the lid was unscrewed and four drops of H2O were added. The inert material was filtered off and the yield was determined using quantitative ^{19}F NMR with 4-fluoroanisole as an internal standard.

Yield: 74% (determined by ^{19}F NMR against 4-fluoroanisole as an internal standard). The overall yield over two steps, starting from bis(4-aminophenyl)methanone, was calculated to be 69%.
^{19}F NMR (470 MHz, $CDCl_3$) δ -105.76.
CAS 345-92-6.
Spectroscopic data are in accordance with those in literature [68].

15. Synthesis of Aryl Fluorides by S_NAr Reaction

15.1. Safety Statement

CAUTION! DMSO undergoes thermal decomposition at temperatures around its boiling point of 189 °C. The presence of impurities and/or acidic substances can cause it to decompose at significantly lower temperature and potentially result in uncontrollable autocatalytic decomposition of DMSO, leading to thermal runaway or even explosions. All S_NAr reactions using KF and DMSO as solvent (5 mL) were carried out ≤ 130 °C. All S_NAr reactions using Me_4NF·*t*AmOH and DMSO as solvent (5 mL) were carried out ≤ 80 °C. All S_NAr reactions using DMSO with heating should be conducted in the presence of a **blast shield**. For large scale experimentation, DMSO should be avoided and a suitable alternative should be used.

15.2. S_NAr reaction of chloroarenes using KF (Method C)

General procedure using acid grade fluorspar derived KF (KF^{AGF}) or metspar derived KF (KF^{M}) (Method C)

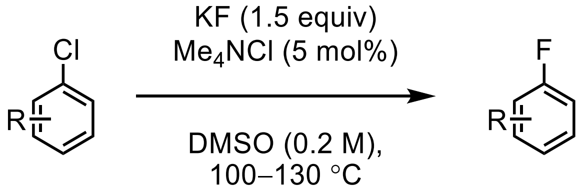

To an oven dried glass reaction vessel was added KF^{AGF} or KF^{M} (1.5 equiv). The KF was dried under high vacuum at 100 °C for at least 1 h. After this time, corresponding chloroarene (1.0 mmol, 1 equiv), Me_4NCl (5.5 mg, 0.05 mmol) and anhydrous DMSO (5 mL) was added to the reaction vessel. The reaction mixture was heated to specified temperature for specified time in a heating block. The resulting suspension was cooled to room temperature, filtered through a short plug of silica gel (washed with ~15 mL EtOAc) to remove insoluble by-products. The filtrate was washed with brine (10 mL). The organic extract was collected and the aqueous layer

was washed with EtOAc (2 x 5 mL). The combined organic extracts were dried over anhydrous Na_2SO_4, filtered, and concentrated *in vacuo*. The residue was purified by column chromatography on silica to afford the title compound.

1-Fluoro-2,4-dinitrobenzene (14)

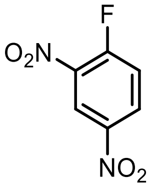

Prepared following **GP C** using 1-chloro-2,4-dinitrobenzene (203 mg, 1.0 mmol) and KF^{AGF} or KF^{M} (87 mg, 1.5 mmol, 1.5 equiv). The reaction is stirred at 70 °C for 1 h. Purification by flash column chromatography on silica using pentane/EtOAc (100:0 to 90:10) gave the title compound as a pale-yellow oil.

Yield using KF^{AGF}: 149 mg, 0.80 mmol, 80%
Yield using $KF^{M(I)}$: 137 mg, 0.74 mmol, 74%

^{1}H NMR (400 MHz, $CDCl_3$) δ 9.10 – 8.84 (m, 1H), 8.67 – 8.43 (m, 1H), 7.59 – 7.49 (m, 1H).
^{13}C NMR (101 MHz, $CDCl_3$) δ 158.80 (d, *J* = 275.2 Hz), 143.67, 137.18, 130.53 (d, *J* = 10.5 Hz), 122.45, 120.10 (d, *J* = 23.0 Hz).
^{19}F NMR (377 MHz, $CDCl_3$) δ -106.17 (ddd, *J* = 10.1, 6.6, 3.8 Hz).
CAS 350-30-1
Spectroscopic data are in accordance with those in literature [70].

2-chloro-1-fluoro-4-nitrobenzene (15)

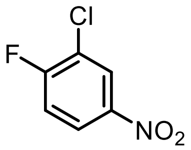

Prepared following **GP C** using 3,4-dichloronitrobenzene (192 mg, 1.0 mmol) and KF^{AGF} (87 mg, 1.5 mmol, 1.5 equiv). The reaction is stirred at 130 °C for 15 h. Purification by flash column chromatography on silica using pentane/Et_2O (100:0 to 95:5) gave the title compound as a pale-yellow solid.

Yield: 134 mg, 0.76 mmol, 76 %
^{1}H NMR (400 MHz, $CDCl_3$) δ 8.35 (dd, *J* = 9.2, 7.9 Hz, 1H), 8.18 (ddd, *J* = 9.2, 4.1, 2.6 Hz, 1H), 7.32 (dd, *J* = 9.2, 7.9 Hz, 1H).
^{13}C NMR (101 MHz, $CDCl_3$) δ 162.1 (d, *J* = 260.1 Hz), 144.4 (s), 126.9 (d, *J* = 1.6 Hz), 124.2 (d, *J* = 8.9 Hz), 122.7 (d, *J* = 19.6 Hz), 117.3 (d, *J* = 23.4 Hz).
^{19}F NMR (377 MHz, $CDCl_3$) δ -103.98 (m).
CAS 350-30-1
Spectroscopic data are in accordance with those in literature [71].

2-fluoro-5-nitrobenzonitrile (16)

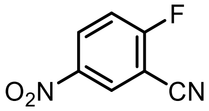

Prepared following **GP C** using 2-chloro-5-nitrobenzonitrile (183 mg, 1.0 mmol) and KF^{AGF} or KF^{M} (87 mg, 1.5 mmol, 1.5 equiv). The reaction is stirred at 100 °C for 6 h. Purification by flash column chromatography on silica using pentane/Et_2O (100:0 to 90:10) gave the title compound as as a pale-yellow solid.

Yield using KF^{AGF}: 157 mg, 0.95 mmol, 95 %
Yield using $KF^{M(I)}$: 148 mg, 0.89 mmol, 89 %

^{1}H NMR (400 MHz, $CDCl_3$) δ 8.58 (dd, *J* = 5.4, 2.7 Hz, 1H), 8.52 (ddd, *J* = 9.2, 4.4, 2.8 Hz, 1H), 7.45 (dd, *J* = 9.2, 7.7 Hz, 1H).
^{13}C NMR (101 MHz, $CDCl_3$) δ 166.2 (d, *J* = 269.8 Hz), 144.3, 130.6 (d, *J* = 10.3 Hz), 129.7 (d, *J* = 2.1 Hz), 118.0 (d, *J* = 21.9 Hz), 111.8, 103.2 (d, *J* = 17.8 Hz).
^{19}F NMR (377 MHz, $CDCl_3$) δ -95.78 (dt, *J* = 7.7, 4.4 Hz).
CAS 17417-09-3
Spectroscopic data are in accordance with those in literature [72].

3-chloro-4-fluorobenzonitrile (17)

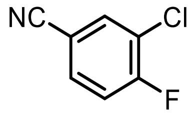

Prepared following **GP C** using 3,4-dichlorobenzonitrile (172 mg, 1.0 mmol) and KF^{AGF} (87 mg, 1.5 mmol, 1.5 equiv). The reaction is stirred at 130 °C for 48 h. Purification by flash column chromatography on silica using pentane/Et_2O (100:0 to 90:10) gave the title compound as as a white solid

Yield: 105 mg, 0.68 mmol, 68%
^{1}H NMR (400 MHz, $CDCl_3$) δ 7.74 (dd, *J* = 6.7, 2.1 Hz, 1H), 7.58 (ddd, *J* = 8.5, 4.4, 2.1 Hz, 1H), 7.27 (m, 1H).
^{13}C NMR (101 MHz, $CDCl_3$) δ 161.0 (d, *J* = 259.0 Hz), 134.8, 132.7 (d, *J* = 8.4 Hz), 123.0 (d, *J* = 18.8 Hz), 118.0 (d, *J* = 22.5 Hz), 117.0, 109.7 (d, *J* = 4.4 Hz).
^{19}F NMR (376 MHz, $CDCl_3$) δ -104.73 (ddd, *J* = 8.5, 6.7, 4.4 Hz).
CAS 117482-84-5
Spectroscopic data are in accordance with those in literature [73].

15.3. S_NAr reactions using acid grade fluorspar derived $Me_4NF\cdot{}^tAmOH$ (Method D)

General procedure for fluorodenitration using acid grade fluorspar derived Me_4NF (Method D)

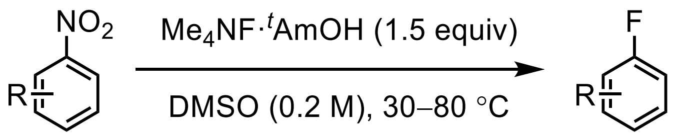

To an oven dried glass reaction vessel containing acid grade fluorspar derived $Me_4NF\cdot{}^tAmOH$ (1.5 or 2.5 equiv) was added the corresponding nitroarene (1.0 mmol, 1.0 equiv) and anhydrous DMSO (5 mL). The reaction mixture was heated to specified temperature for specified time in a heating block. The resulting suspension was cooled to room temperature, filtered through a short plug of silica gel (washed with ~15 mL EtOAc) to remove insoluble by-products. The filtrate was washed with brine (10 mL). The organic extract was collected and the aqueous layer was washed with EtOAc (2 x 5 mL). The combined organic extracts were dried over anhydrous Na_2SO_4, filtered, and concentrated *in vacuo*. The residue was purified by column chromatography on silica to afford the title compound.

2,6-difluorobenzonitrile (18)

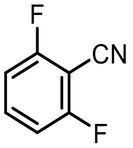

Prepared following **GP D** using 2,6-dinitrobenzonitrile (193 mg, 1.0 mmol) and $Me_4NF{\cdot}{}^tAmOH$ (544 mg, 3.0 mmol, 3.0 equiv). The reaction is stirred at 30 °C for 15 h. Purification by flash column chromatography on silica using pentane/Et_2O (100:0 to 98:2) gave the title compound as a white solid.

Yield: 117 mg, 0.84 mmol, 84%
^{1}H NMR (400 MHz, $CDCl_3$) δ 7.62 (tt, J = 8.5, 6.3 Hz, 1H), 7.11 – 7.02 (m, 2H).
^{13}C NMR (101 MHz, $CDCl_3$) δ 163.3 (dd, J = 261.6, 4.2 Hz), 135.8 (t, J = 10.1 Hz), 112.3 (dd, J = 19.4, 3.7 Hz), 109.2, 92.5 (t, J = 19.1 Hz).
^{19}F NMR (377 MHz, $CDCl_3$) δ -101.87 – -105.36 (m).
CAS 1897-52-5
Spectroscopic data are in accordance with those in literature [74].

4-fluoronitrobenzene (19)

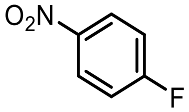

Prepared following **GP D** using 1,4-dinitrobenzene (168 mg, 1.0 mmol) and $Me_4NF{\cdot}{}^tAmOH$ (272 mg, 1.5 mmol, 1.5 equiv). The reaction is stirred at 80 °C for 15 min. Purification by flash column chromatography on silica using pentane/EtOAc (100:0 to 90:10) gave the title compound as a yellow oil.

Yield: 124 mg, 0.88 mmol, 88%
^{1}H NMR (400 MHz, $CDCl_3$) δ 8.32 – 8.23 (m, 2H), 7.25 – 7.16 (m, 2H).
^{13}C NMR (101 MHz, $CDCl_3$) δ 166.4 (d, J = 258.0 Hz), -144.5, 126.5 (d, J = 10.0 Hz), 116.6 (d, J = 23.7 Hz).
^{19}F NMR (377 MHz, $CDCl_3$) δ -101.35 – -103.81 (m).
CAS 350-46-9
Spectroscopic data are in accordance with those in literature [75].

2-fluorobenzonitrile (20)

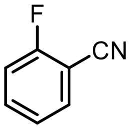

Prepared following **GP D** using 2-nitrobenzonitrile (148 mg, 1.0 mmol) and $Me_4NF{\cdot}{}^tAmOH$ (272 mg, 1.5 mmol, 1.5 equiv). The reaction is stirred at 80 °C for 18 h. Purification by flash column chromatography on silica using pentane/Et_2O (100:0 to 95:5) gave the title compound as a colorless oil.

Yield: 88 mg, 0.73 mmol, 73%
^{1}H NMR (400 MHz, $CDCl_3$) δ 7.68 – 7.57 (m, 2H), 7.30 – 7.25 (m, 1H), 7.25 – 7.19 (m, 1H).
^{13}C NMR (101 MHz, $CDCl_3$) δ 163.2 (d, J = 259.0 Hz), 135.2 (d, J = 8.3 Hz), 133.6, 125.0 (d, J = 3.8 Hz), 116.59 (d, J = 19.4 Hz), 114.1, 101.6 (d, J = 15.4 Hz).
^{19}F NMR (377 MHz, $CDCl_3$) δ -106.30 (m).
CAS 394-47-8
Spectroscopic data are in accordance with those in literature [76].

16. One-pot fluoro-dediazotization using acid grade fluorspar derived $LiBF_4$

16.1. Synthesis of $LiBF_4$ from acid grade fluorspar

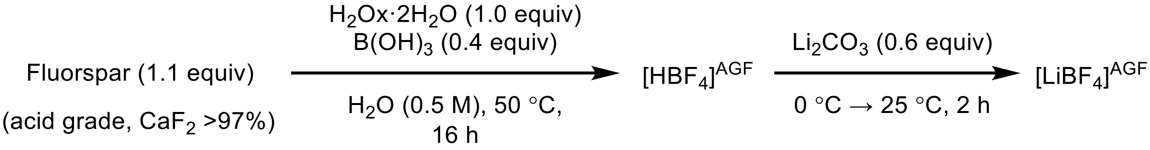

Acid grade fluorspar (> 97% CaF_2, 5.56 g, 71.16 mmol, 1.1 equiv), boric acid (2.00 g, 32.35 mmol, 0.5 equiv) and oxalic acid dihydrate (8.16 g, 64.69 mmol, 1.0 equiv) were weighed into a 100 mL PTFE round bottom flask. A stir bar and water (16 mL) were added and the mixture was heated to 50 °C for 16 h. The resulting suspension was then cooled to room temperature, the solids were filtered off using a Büchner funnel and further washed with water (2 x 5 mL) to obtain a solution containing $[HBF_4]^{AGF}$. Subsequently, Li_2CO_3 (1.43 g, 19.41 mmol, 0.6 equiv.) was gradually added to the $[HBF_4]^{AGF}$ solution over 15 min at 0 °C and the mixture was stirred at 25 °C for an additional 2 h. Next, the resulting solution was evaporated to dryness using the rotatory evaporator. The yellowish crystals obtained were redissolved in acetonitrile (20 mL) and solid impurities were separated by filtration. The filtrate was concentrated until small crystals formed and then stored in the freezer (-18 °C) overnight. The white crystals of $[LiBF_4]^{AGF}$ were filtered off and dried under high vacuum at 50 °C overnight to afford acid grade fluorspar derived $LiBF_4$ as a white solid (1.68 g, 17.92 mmol, 55 %).

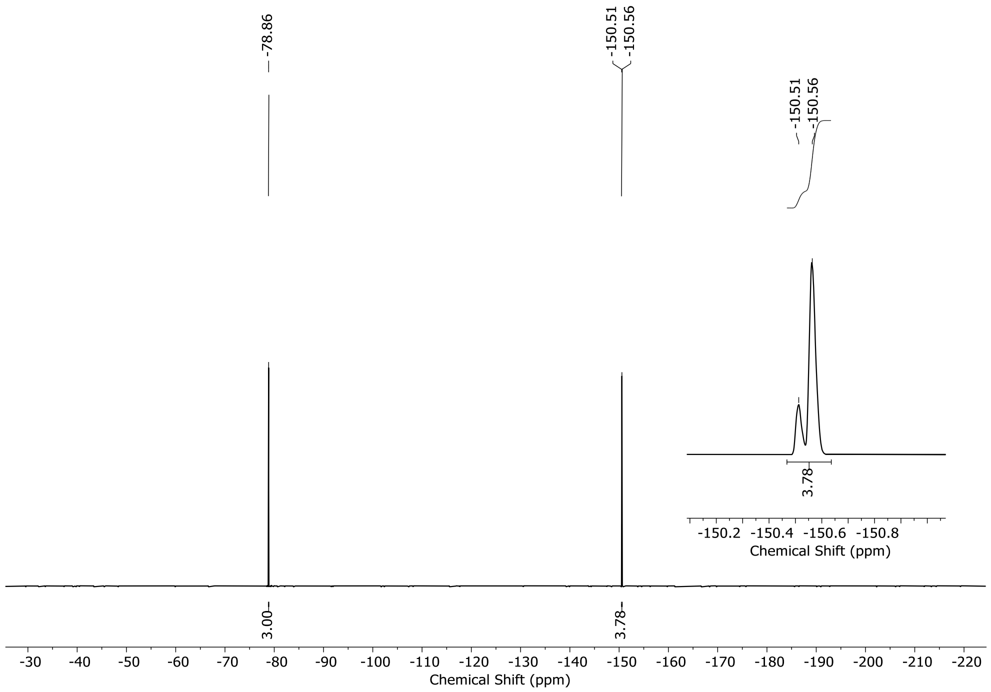

Figure S56. Determination of the purity of $[LiBF_4]^{AGF}$ by quantitative ^{19}F NMR against NaOTf as internal standard. A sample of $[LiBF_4]^{AGF}$ (15.0 mg) and sodium triflate as an NaOTf (27.5 mg) was dissolved in D_2O. The purity of $[LiBF_4]^{AGF}$ was calculated to be 96%.

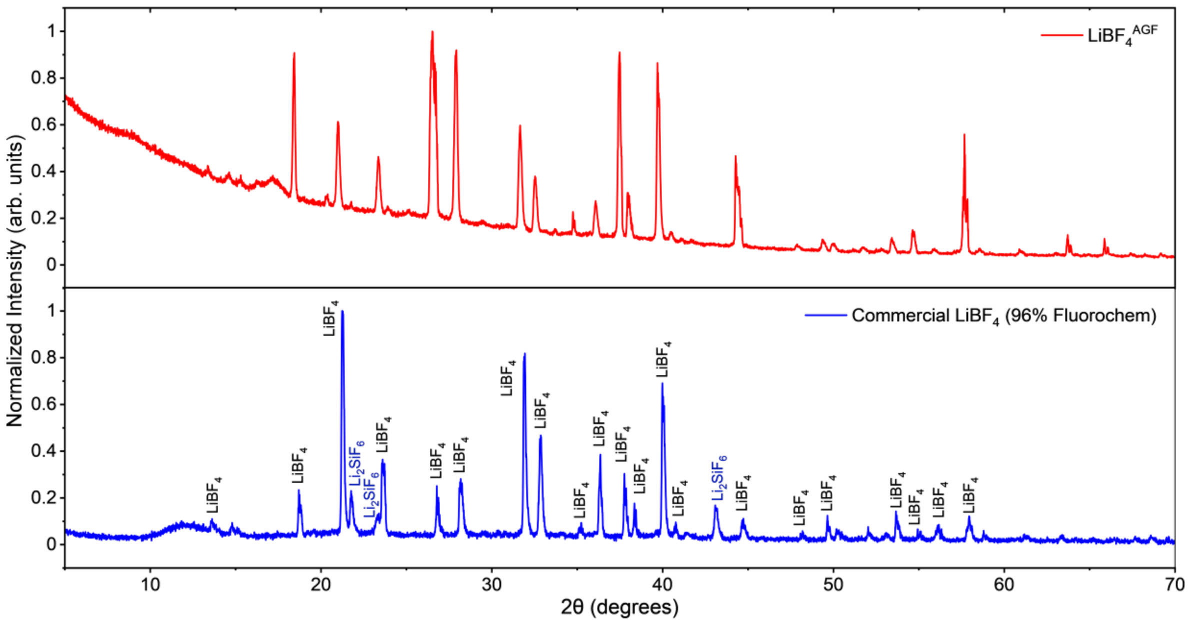

Figure S57. Powder X-ray diffraction pattern of $LiBF_4^{AGF}$ (top trace, red) and commercial $LiBF_4$ (bottom trace, blue). Commercial $LiBF_4$ contains Li_2SiF_6 impurity. All data collected at room temperature.

16.2. Application of $LiBF_4$ in one-pot Balz Schiemann reaction

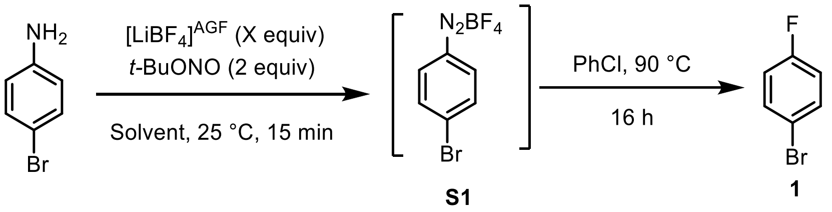

Following a modified procedure outlined in ref[77]. In a screwcap sealed tube, 4-bromoaniline (25.8 mg, 0.15 mmol, 1 equiv.), $[LiBF_4]^{AGF}$ (141 mg, 1.5 mmol, 10 equiv.), tert-butyl nitrite (38 µL, 0.3 mmol, 2 equiv.), and acetonitrile (0.50 mL) were combined. The reaction mixture was stirred at 25 °C for 15 min, followed by addition of chlorobenzene (0.5 mL). The tube was heated to 90 °C overnight under a blast shield, after which the lid was unscrewed and white smoke emerged the vial. Saturated $NaHCO_3$ (2 mL) was added to quench any potential remaining generated diazonium salt followed by the addition of 4-fluoroanisole (0.15 mmol) as an internal standard and $CDCl_3$ (1 mL). An aliquot of the organic layer was removed and analyzed by quantitative ^{19}F NMR.

Table S31. One-pot Balz-Schiemann reaction protocol with varying amounts of $[LiBF_4]^{AGF}$. [a] Yields refer to quantitative ^{19}F-NMR yields using 4-fluoroanisole an internal standard.

Entry	$[LiBF_4]^{AGF}$ equiv.	Solvent	Overall yield (%)
1	2	MeCN	8
2	5	MeCN	29
3	10	MeCN	47
4	10	*n*-butylacetate	66

The one-pot fluoro-dediazotization of diazonium salts was applied to the synthesis of **4** and **9** to avoid the precipitation of diazonium salts **S4** and **S9** (T_{D24} values < 25 °C, see **Table S29**). This method yielded 2-chloro-3-fluoropyridine (**4**) in 66% and 1-fluoro-3-methylbenzene (**9**) in 54% yield (as quantified by ^{19}F NMR spectroscopy).

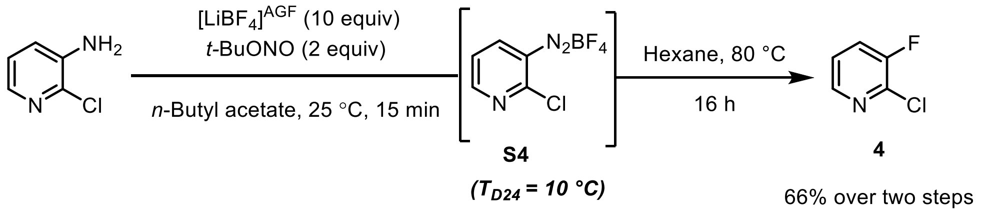

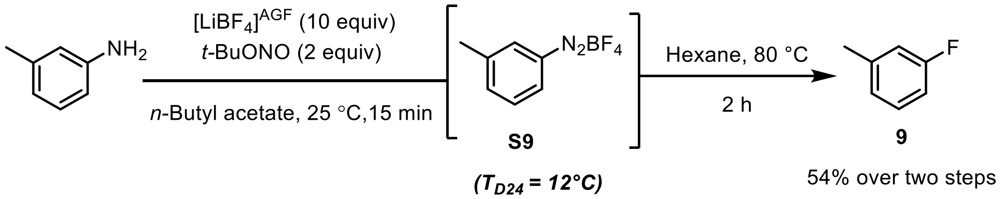

17. Stability of aqueous [B-F] and [Si-F] solutions prepared from acid grade fluorspar

Aqueous solutions of HBF_4 (48 wt. % in H_2O) are commercially available and sold at room temperature. In water, HBF_4 is in equilibrium with HBF_3OH with both species present in a ratio of 89:11 as determined by ^{19}F NMR spectroscopy (D_2O) (**Table S32**, entry 1). The composition of aqueous solutions of HBF_4 prepared from acid-grade fluorspar (AGF) upon treatment with oxalic acid and boric acid was studied by ^{19}F NMR. We did not observe species other than HBF_4, HBF_3OH and HBF_2OX (<0.5%) for samples prepared in house when stored at a 4.8M concentration for several months at room temperature (**Table S32**, entries 2 and 3).

Table S32. Stability studies on $[HBF_4]^{AGF}$

Entry	Time since initial preparation	HBF_4 conc.	HBF_4 (%)	HBF_3OH (%)
1	-	7.65 M [a]	89	11
2	0 months	4.80 M	83	17
3	3 months	4.80 M	86	14

[a] Measured using commercial HBF_4 solution (Sigma Aldrich)

A solution of [Si-F] was stored in a 50 mL conical polypropylene tubes at 25 °C for 4 weeks (**Table S33**). After this period, the distribution of [Si-F] species was analysed by quantitative ^{19}F NMR spectroscopy (D_2O) and was found to be H_2SiF_6 (54%), H_2SiF_5OH (38%) and H_2OxSiF_4 (3%). At week 0 it was H_2SiF_6 (39%), H_2SiF_5OH (56%) and H_2OxSiF_4 (2%). Using the aqueous solution of [Si-F] stored at 25 °C after 4 weeks, KF^{AGF} was prepared in 88% yield (purity 92% confirmed by ^{19}F NMR) [cf. using freshly prepared [Si-F] solution (0 weeks), KF^{AGF} was prepared in 85% yield (90% purity)].

Table S33. Stability studies on $[Si\text{-}F]^{AGF}$

Entry	Time since initial preparation	H_2SiF_6 (%)	H_2SiF_5OH (%)	H_2OxSiF_4 (%)	Total [Si-F] (%)
1	0 weeks	39	56	2	97
2	4 weeks	54	38	3	95

18. NMR Spectra

18.1. Spectra for isolated products

4-Bromobenzenediazonium tetrafluoroborate (S1) ^{1}H NMR (DMSO-d_6)

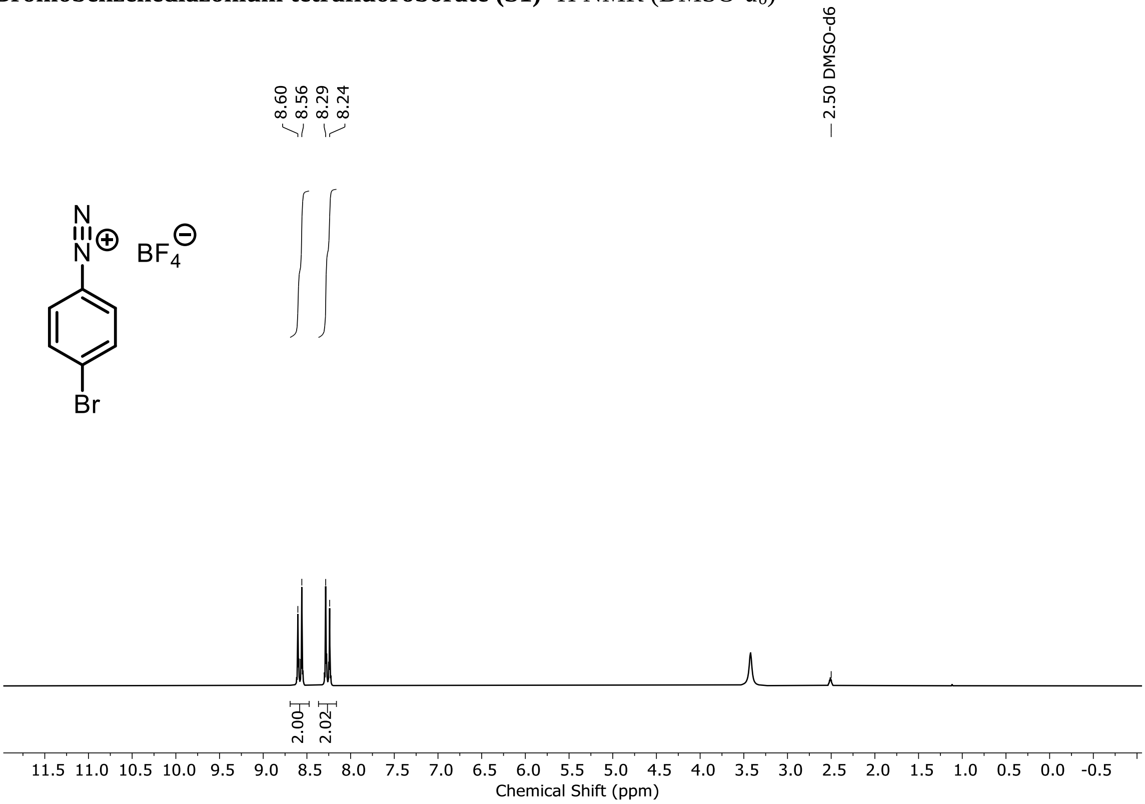

2,4-Dichlorobenzenediazonium tetrafluoroborate (S2) ^{1}H NMR (DMSO-d_6)

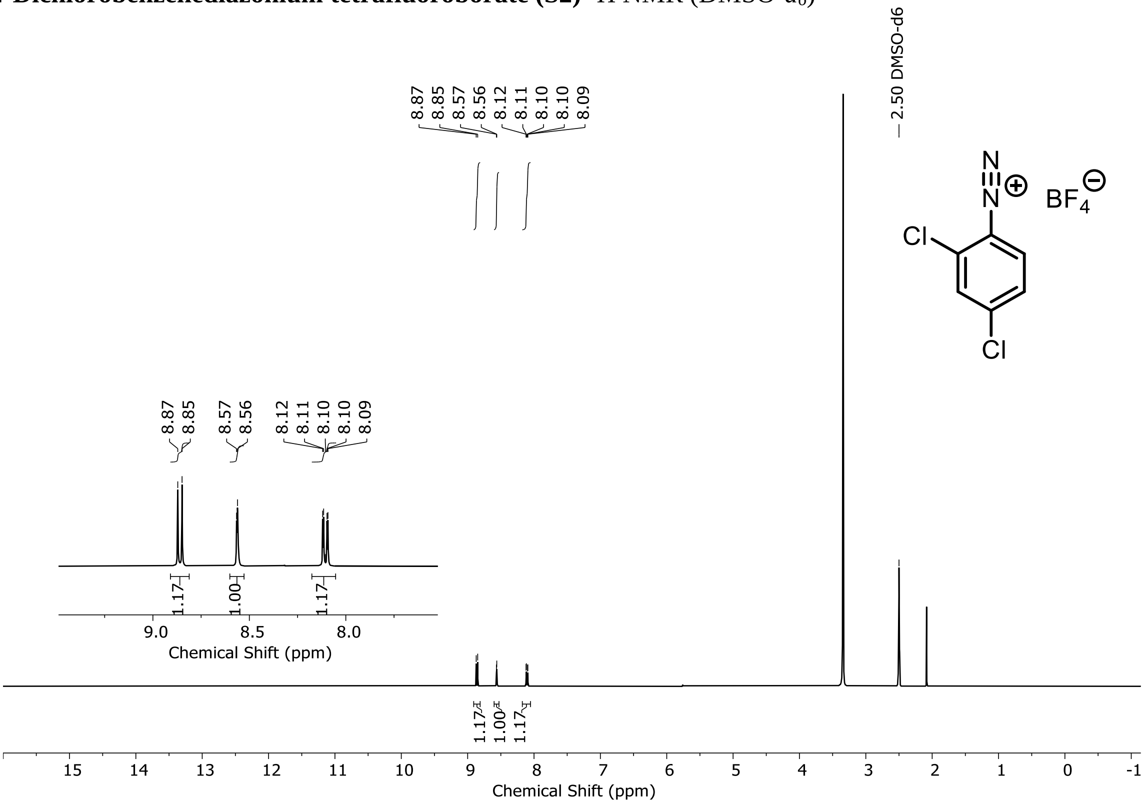

4-Cyanobenzenediazonium tetrafluoroborate (S3) ^{1}H NMR (DMSO-d_6)

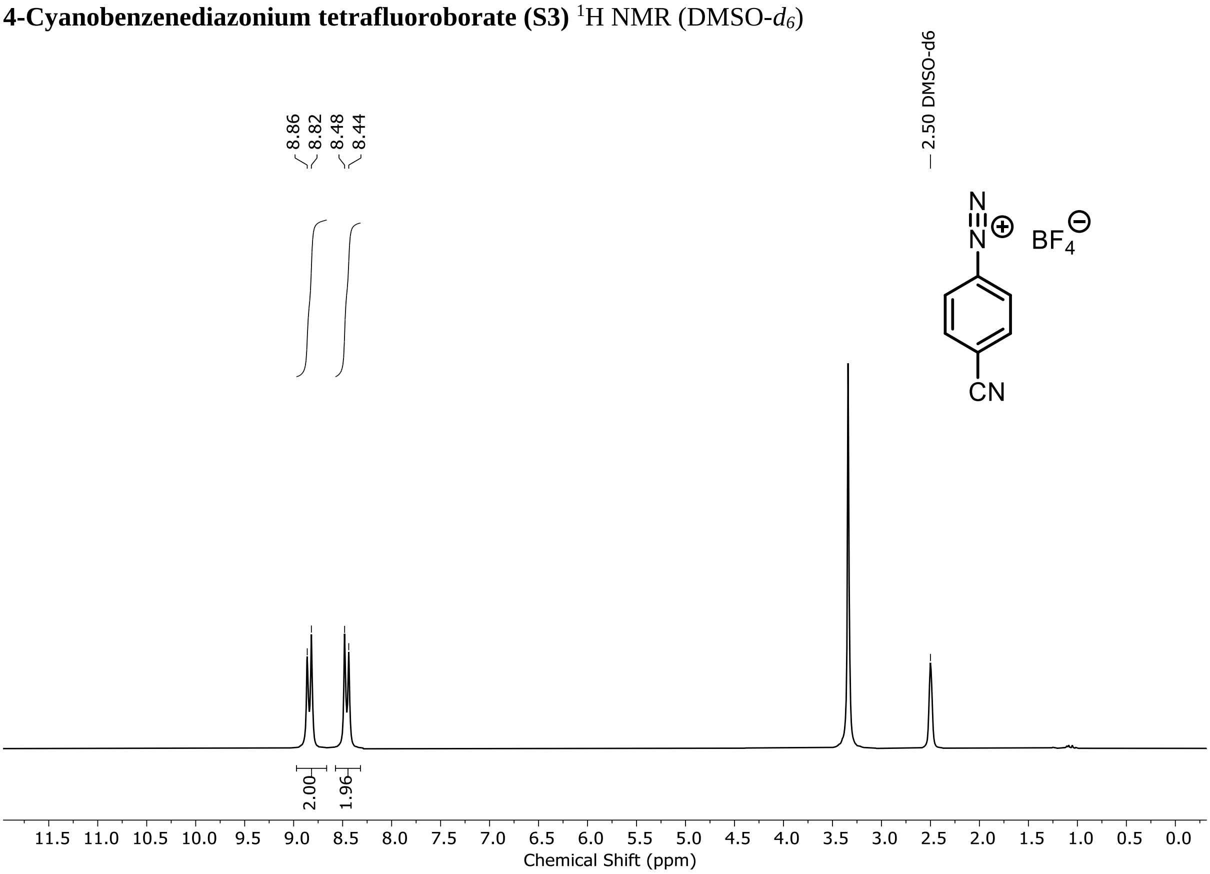

2-Chloropyridine-3-diazoniumtetrafluoroborate (S4) ^{1}H NMR (CD_3CN)

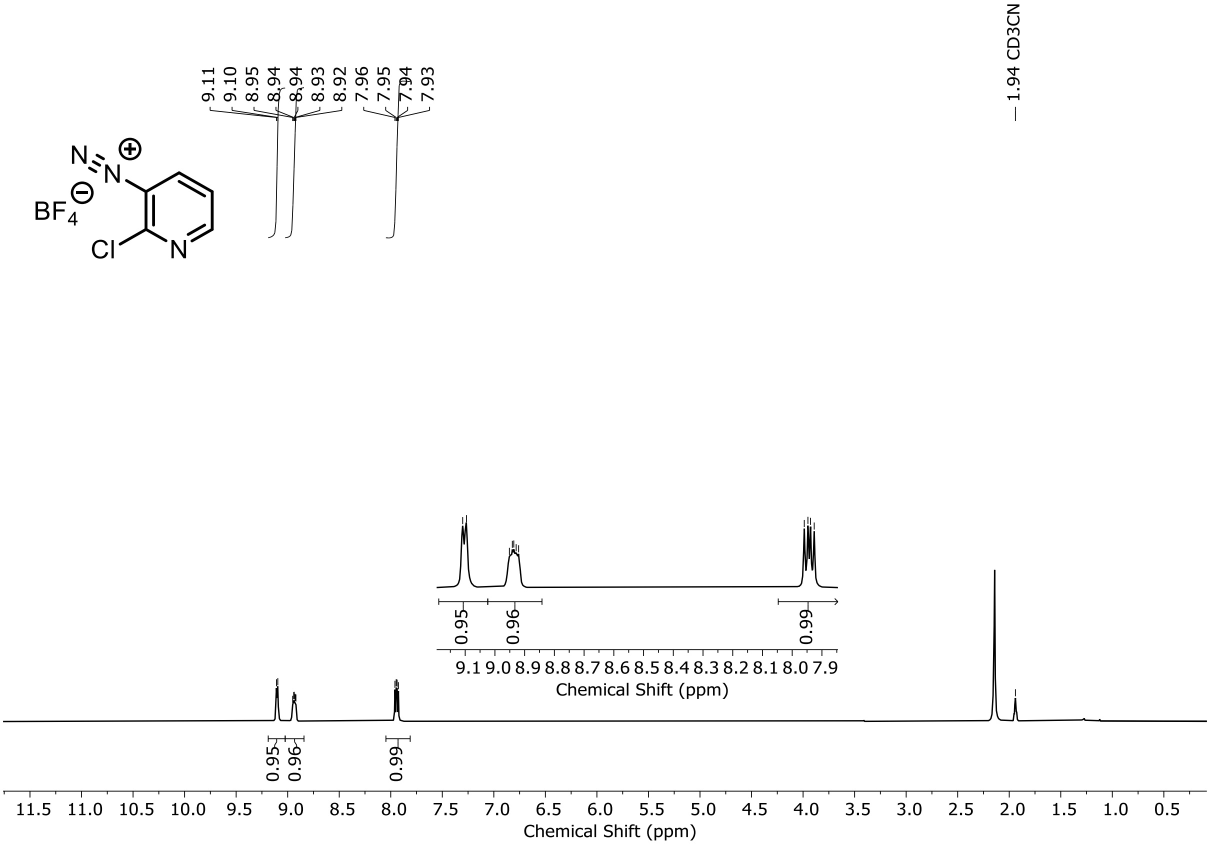

2-Chloropyridine-3-diazoniumtetrafluoroborate (S4) ^{19}F NMR (CD_3CN)

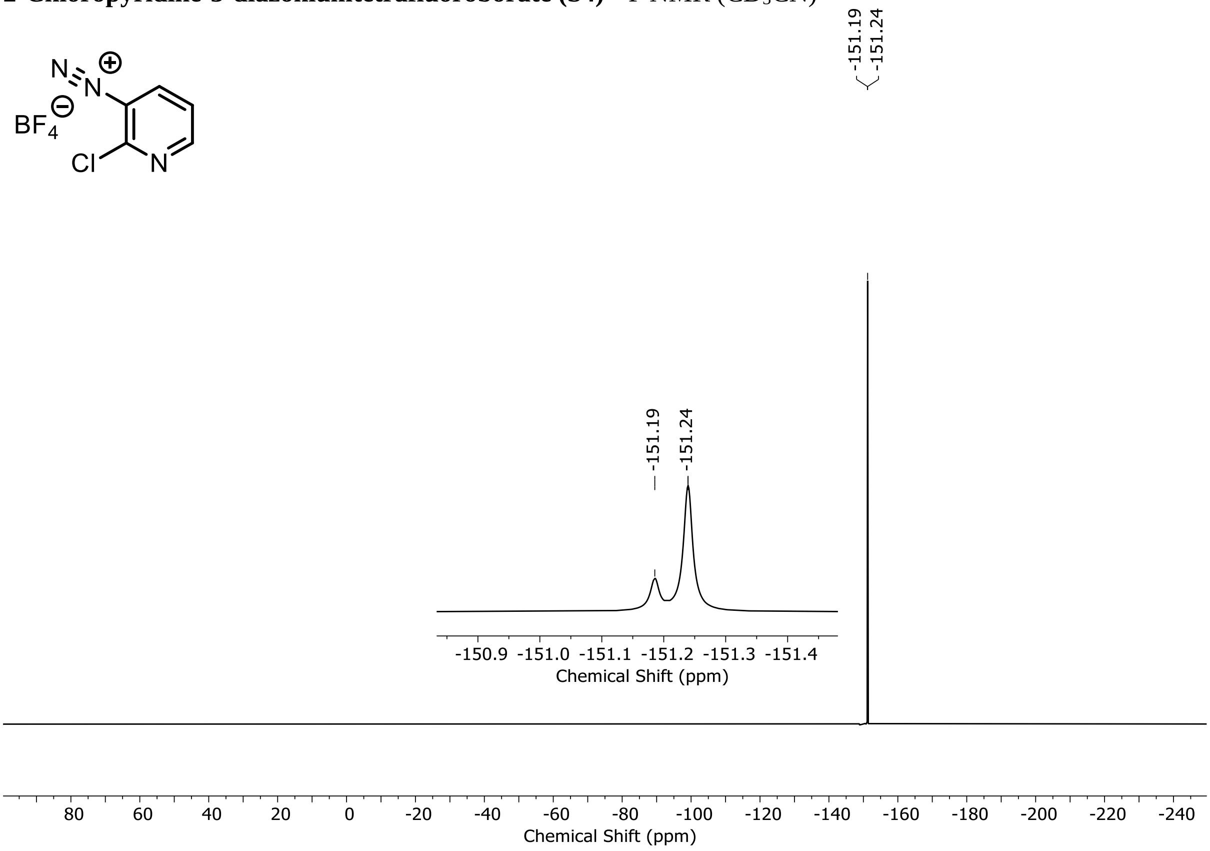

2-Chloropyridine-3-diazoniumtetrafluoroborate (S4) ^{13}C NMR (CD_3CN)

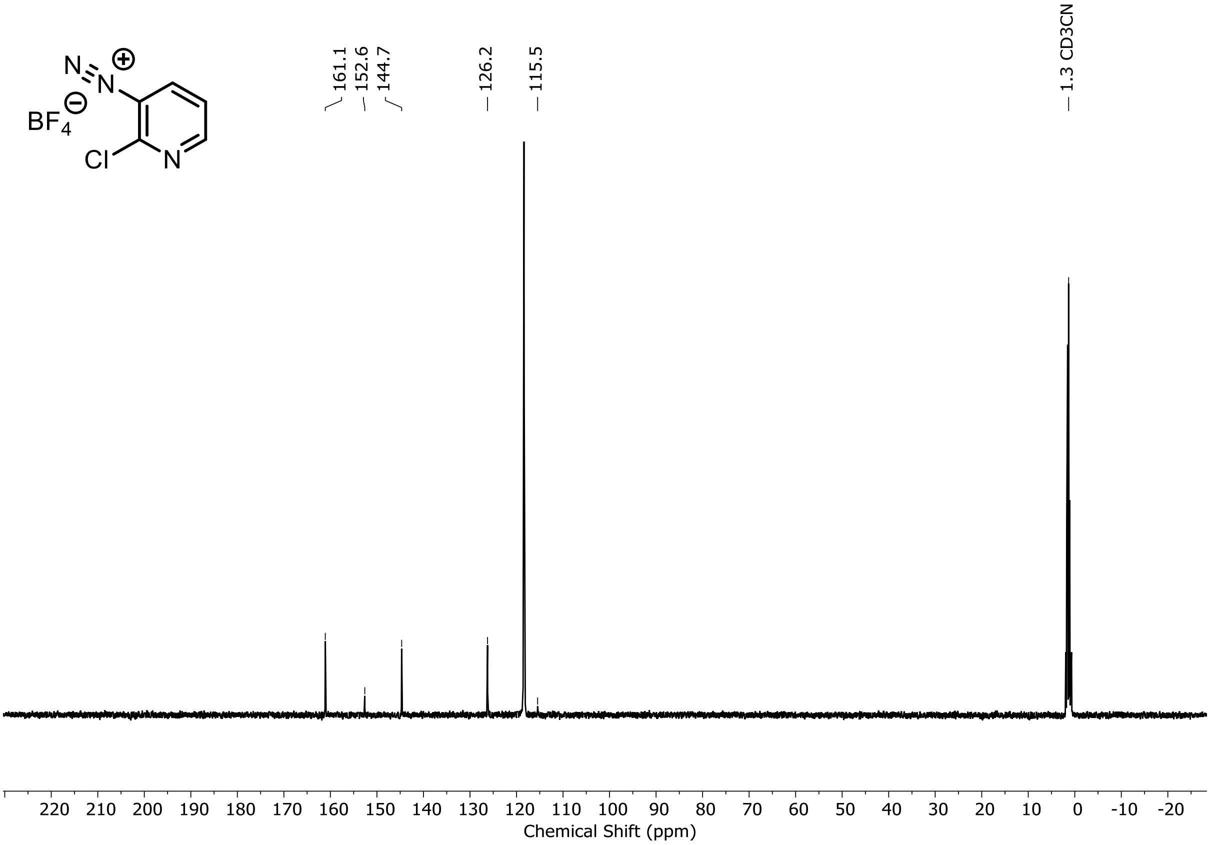

4-Methoxybenzenediazonium tetrafluoroborate (S5) ^{1}H NMR (DMSO-d_6)

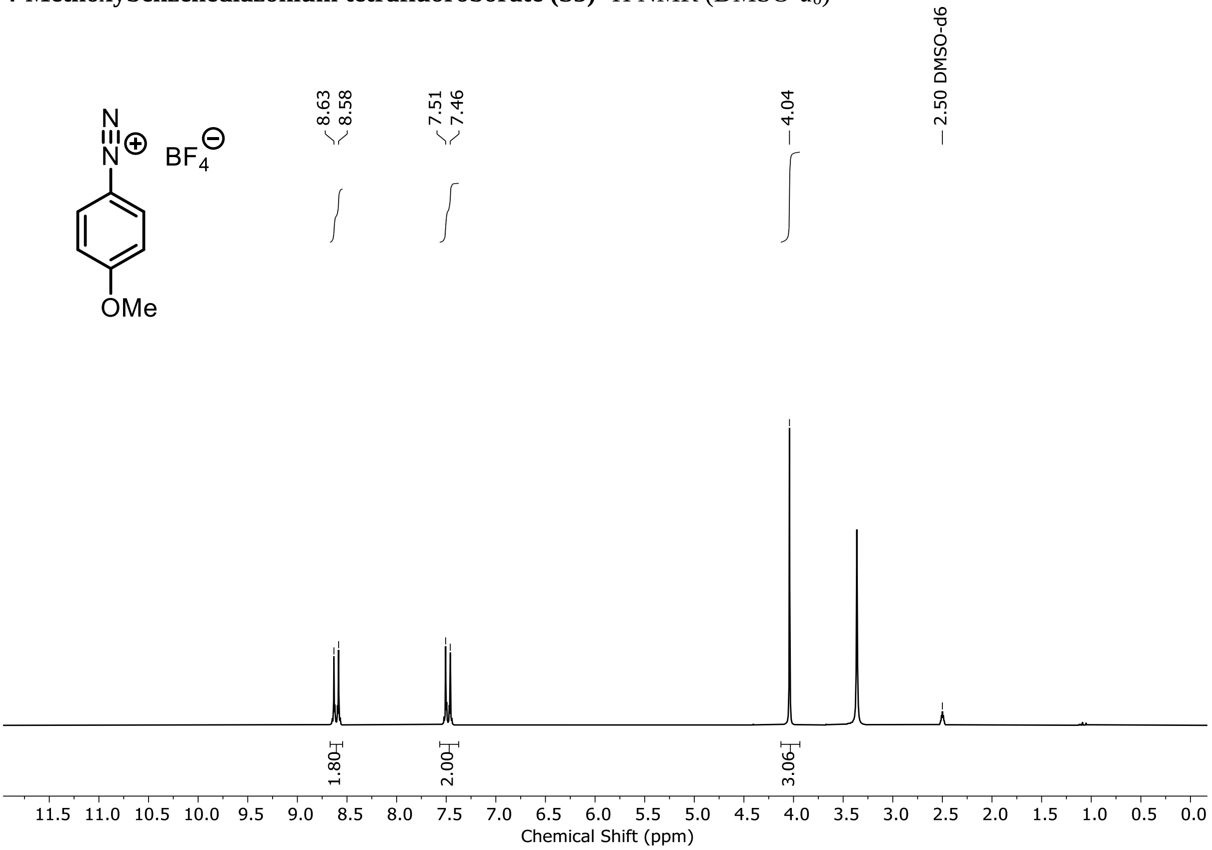

4-Acetylbenzenediazonium tetrafluoroborate (S6) ^{1}H NMR (DMSO-d_6)

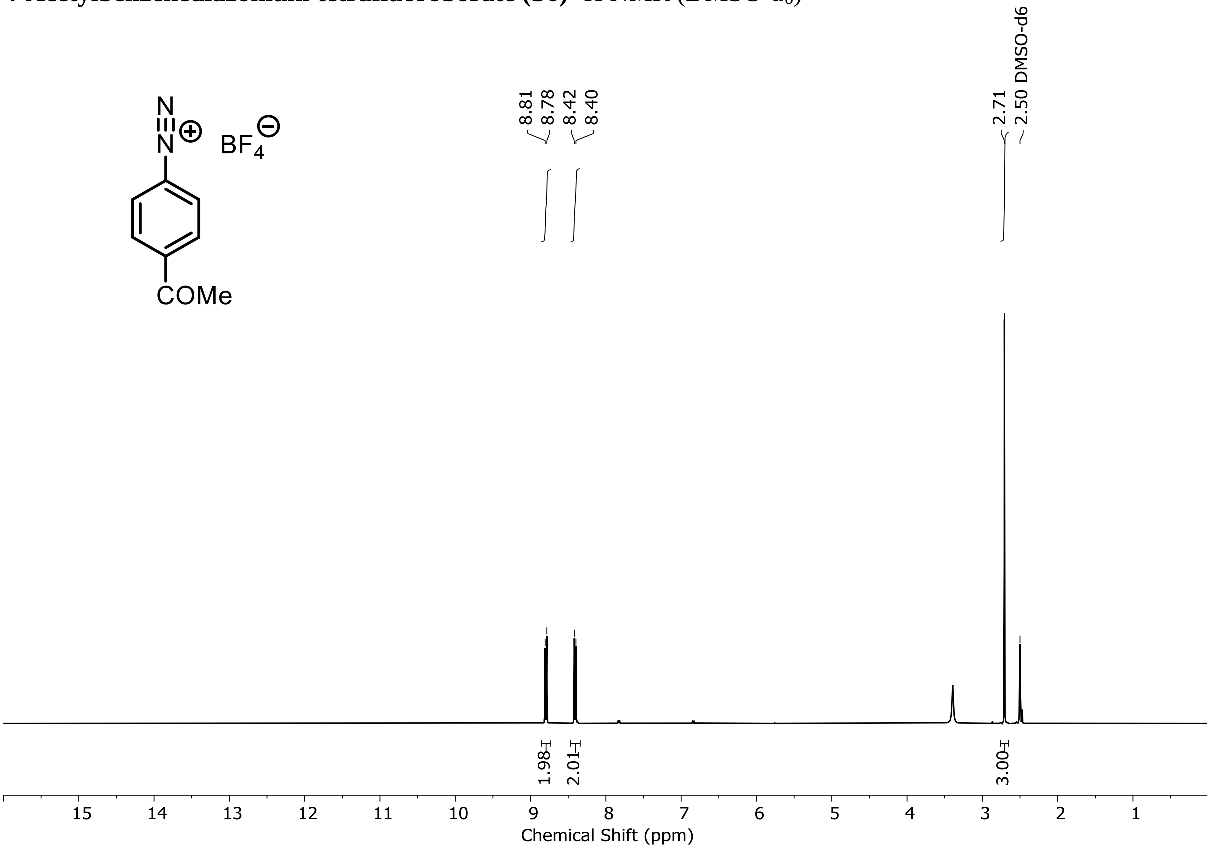

4-(Methoxycarbonyl)benzenediazonium tetrafluoroborate (S7) ^{1}H NMR (DMSO-d_6)

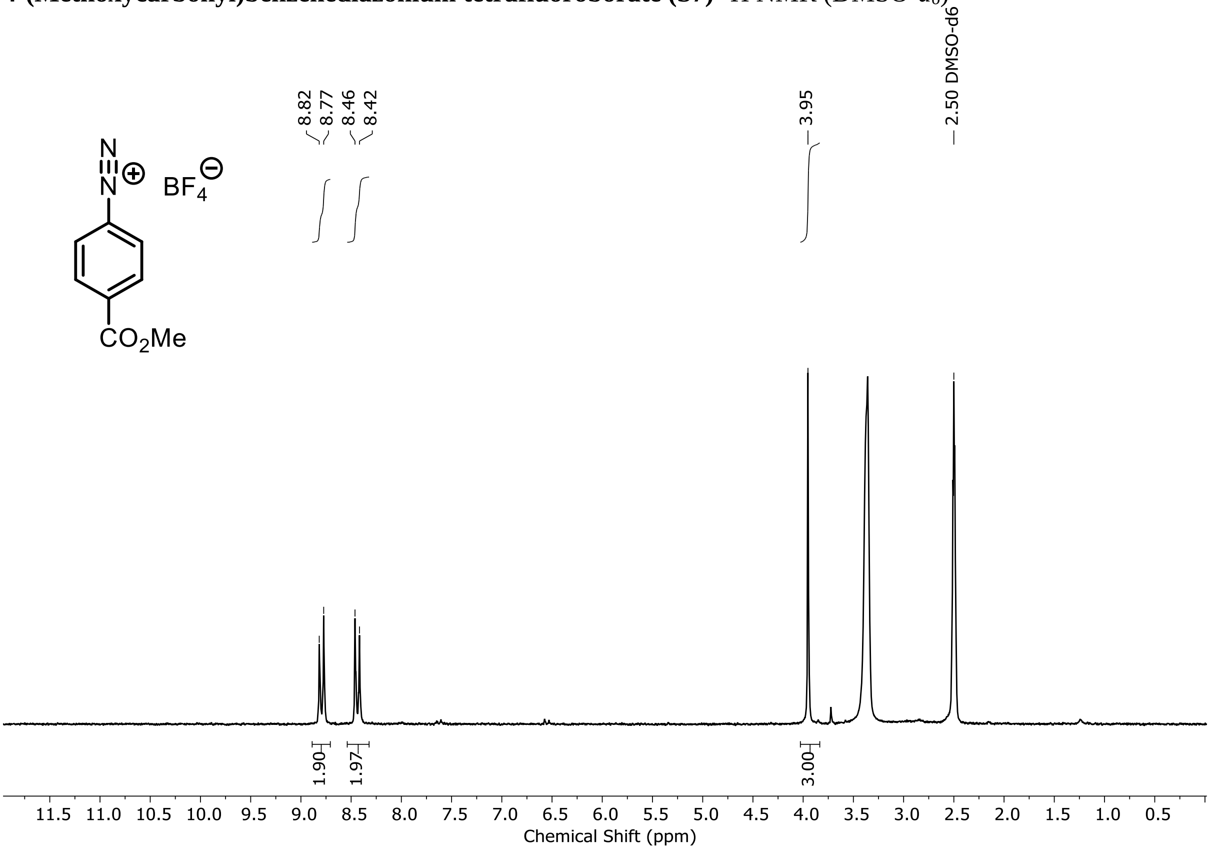

3-Methylbenzenediazonium tetrafluoroborate (S9) ^{1}H NMR (CD_3CN)

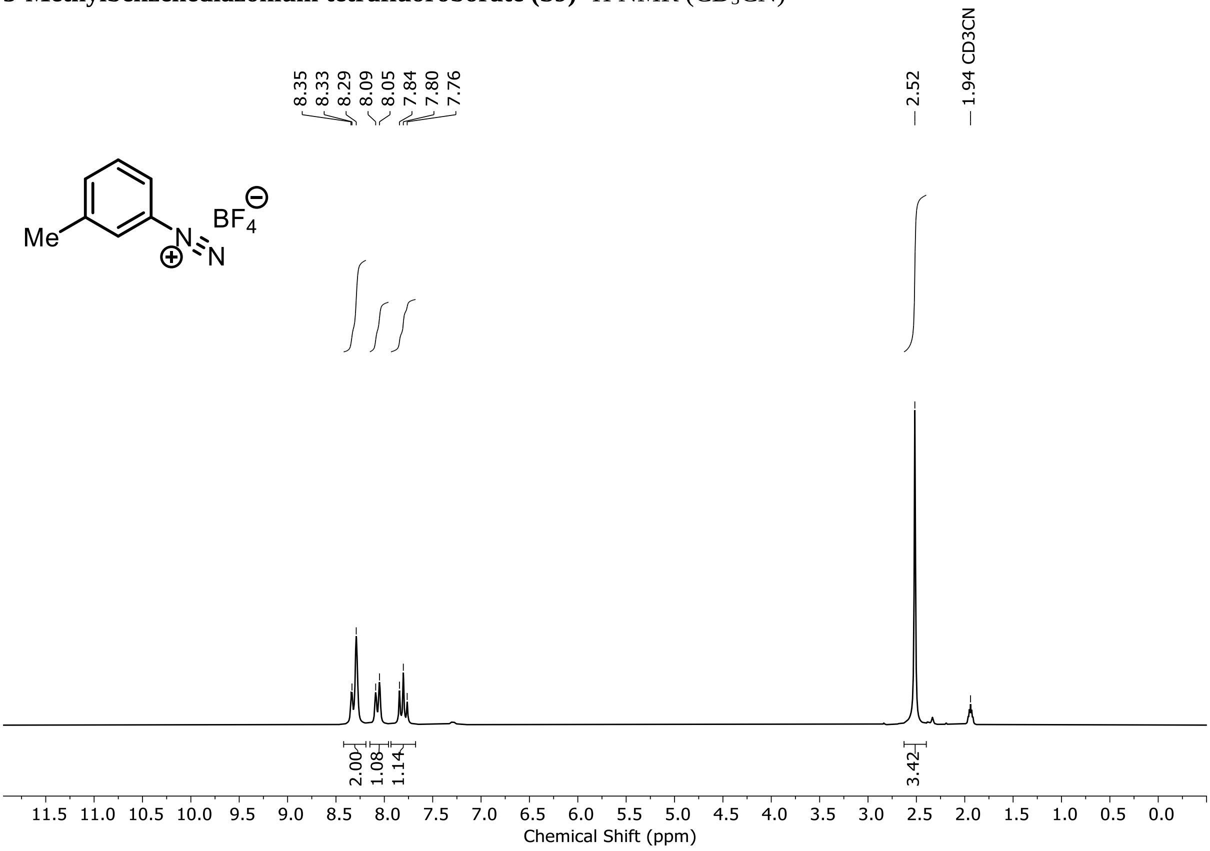

4-bromo-[1,1'-biphenyl]-2-diazonium tetrafluoroborate (S10) ^{1}H NMR (DMSO-d_6)

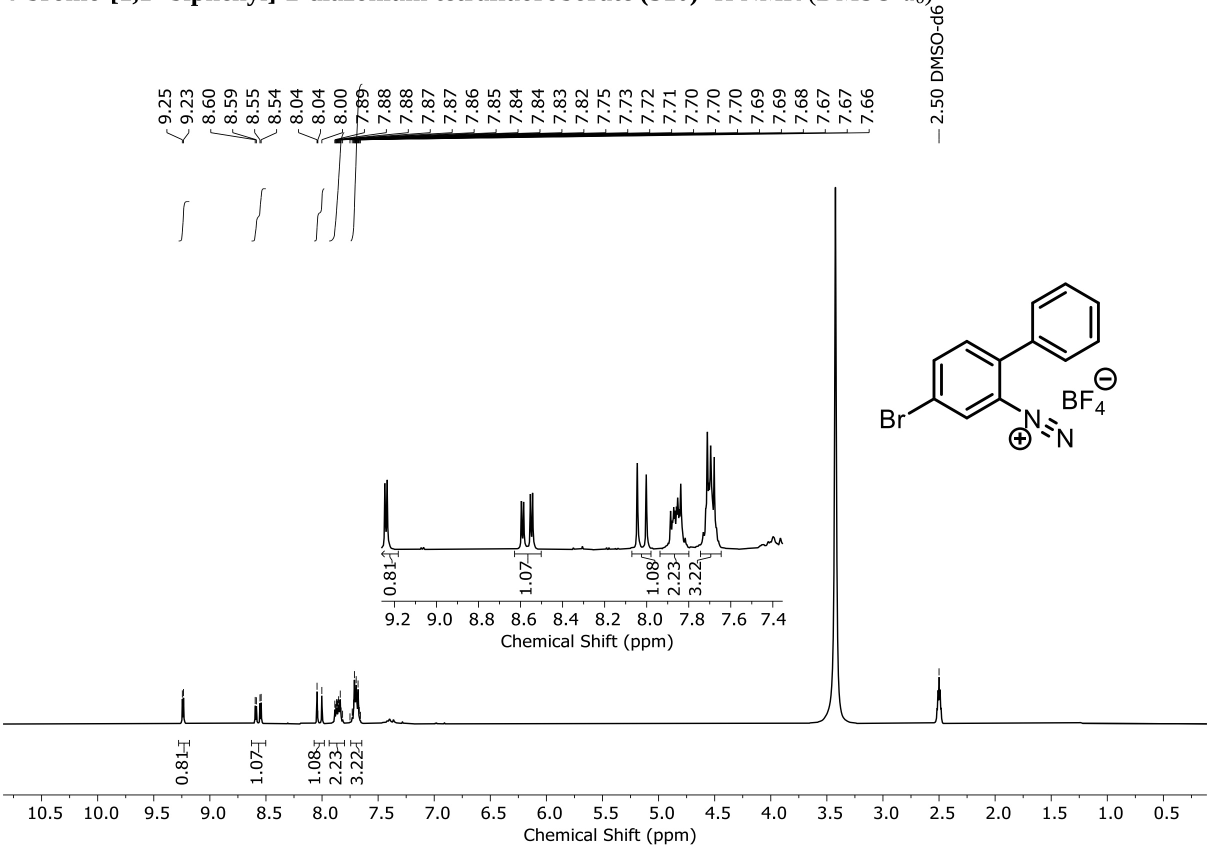

4-bromo-[1,1'-biphenyl]-2-diazonium tetrafluoroborate (S10) ^{19}F NMR (DMSO-d_6)

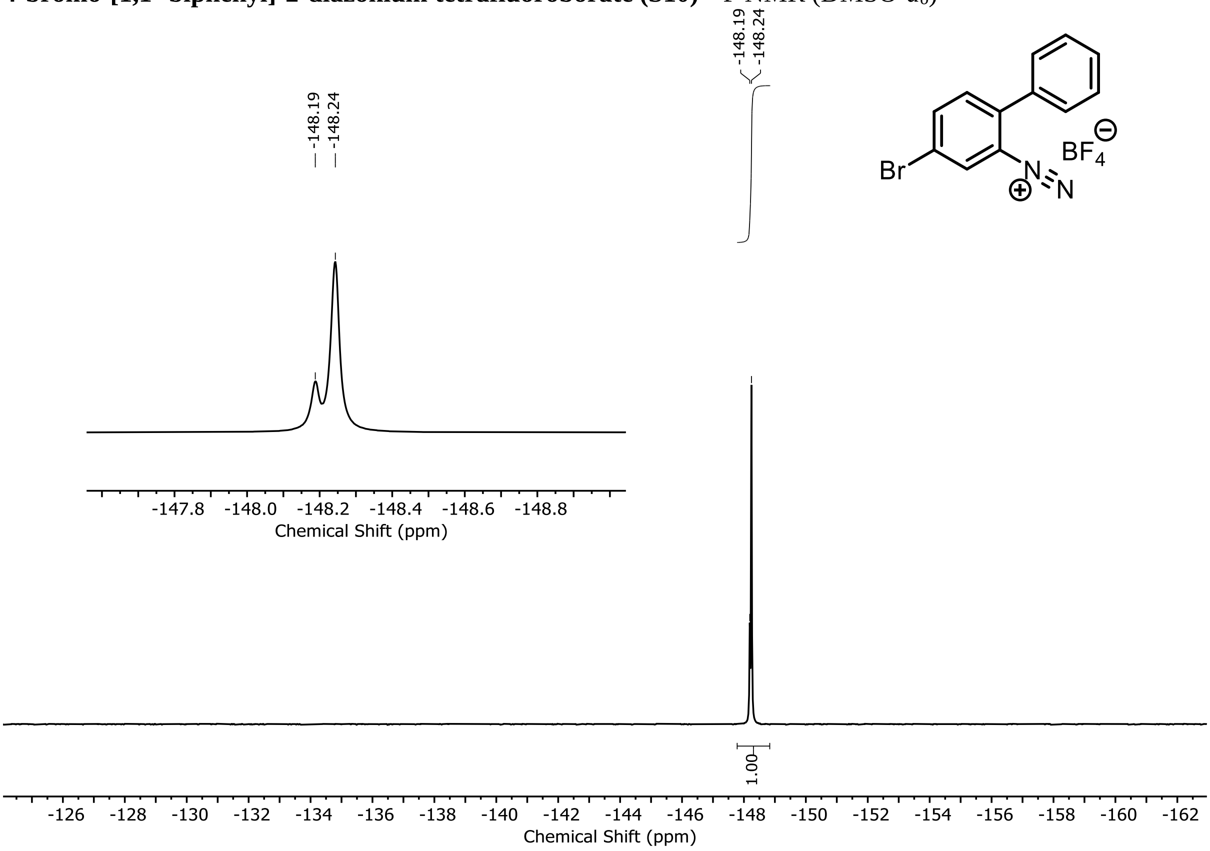

4-bromo-[1,1'-biphenyl]-2-diazonium tetrafluoroborate (S10) ^{13}C NMR (DMSO-d_6)

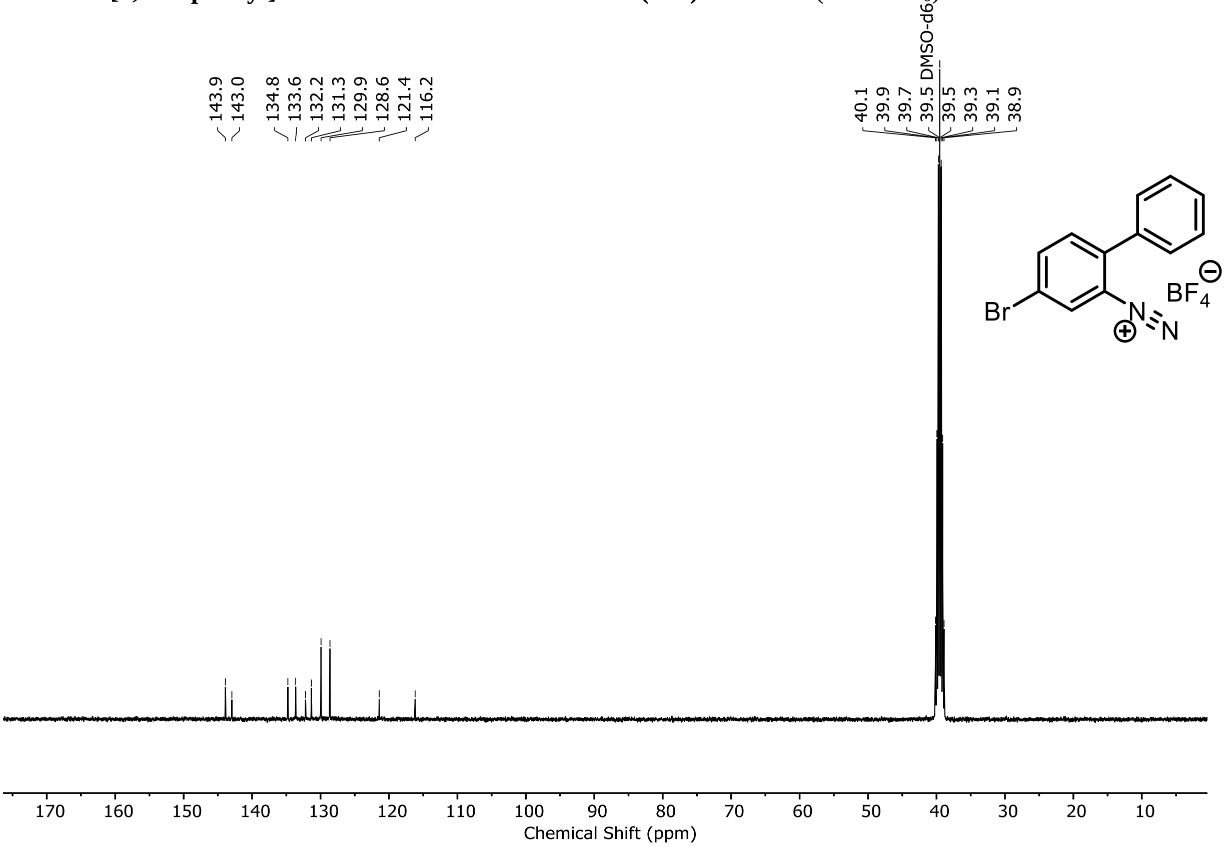

4-formylbenzenediazonium tetrafluoroborate (S12) ^{1}H NMR (CD_3CN)

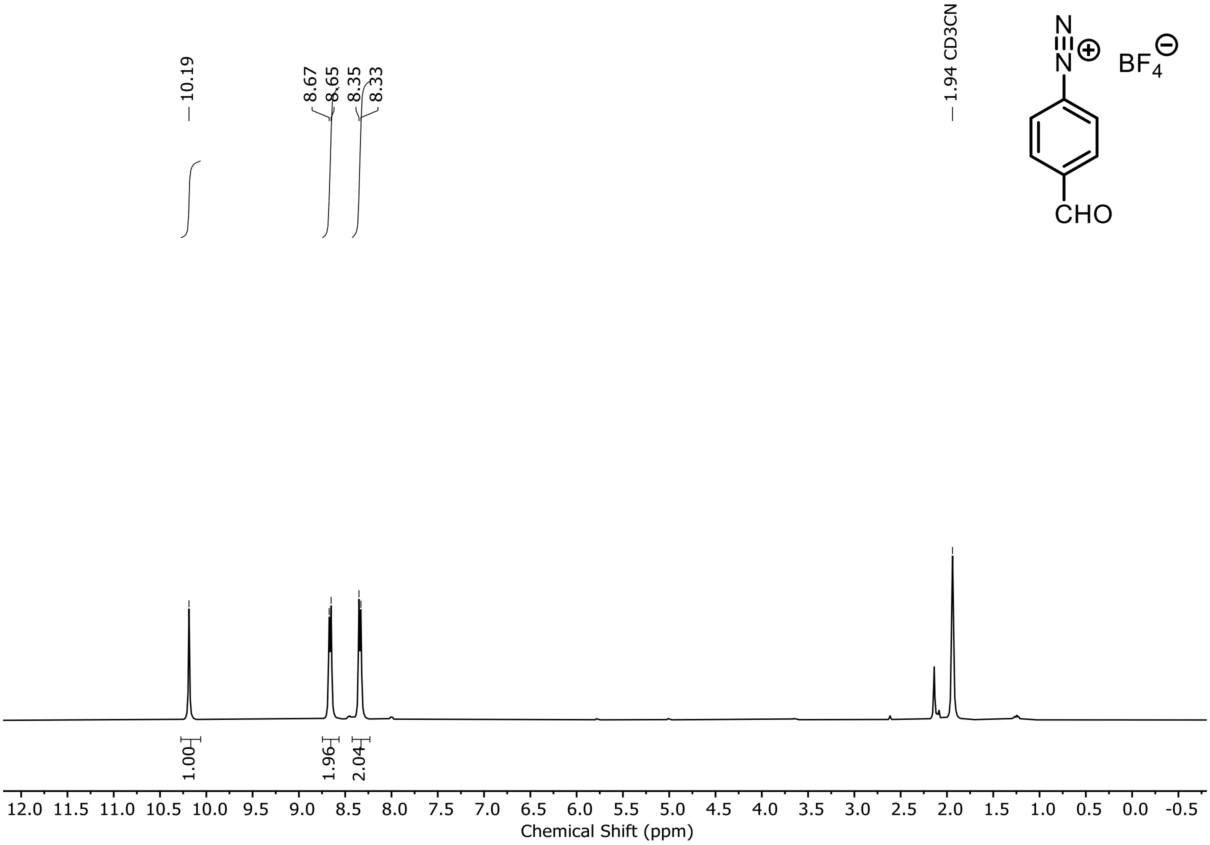

6-chloro-5-methylpyridine-3-diazonium tetrafluoroborate (S13) ^{1}H NMR (DMSO-d_6)

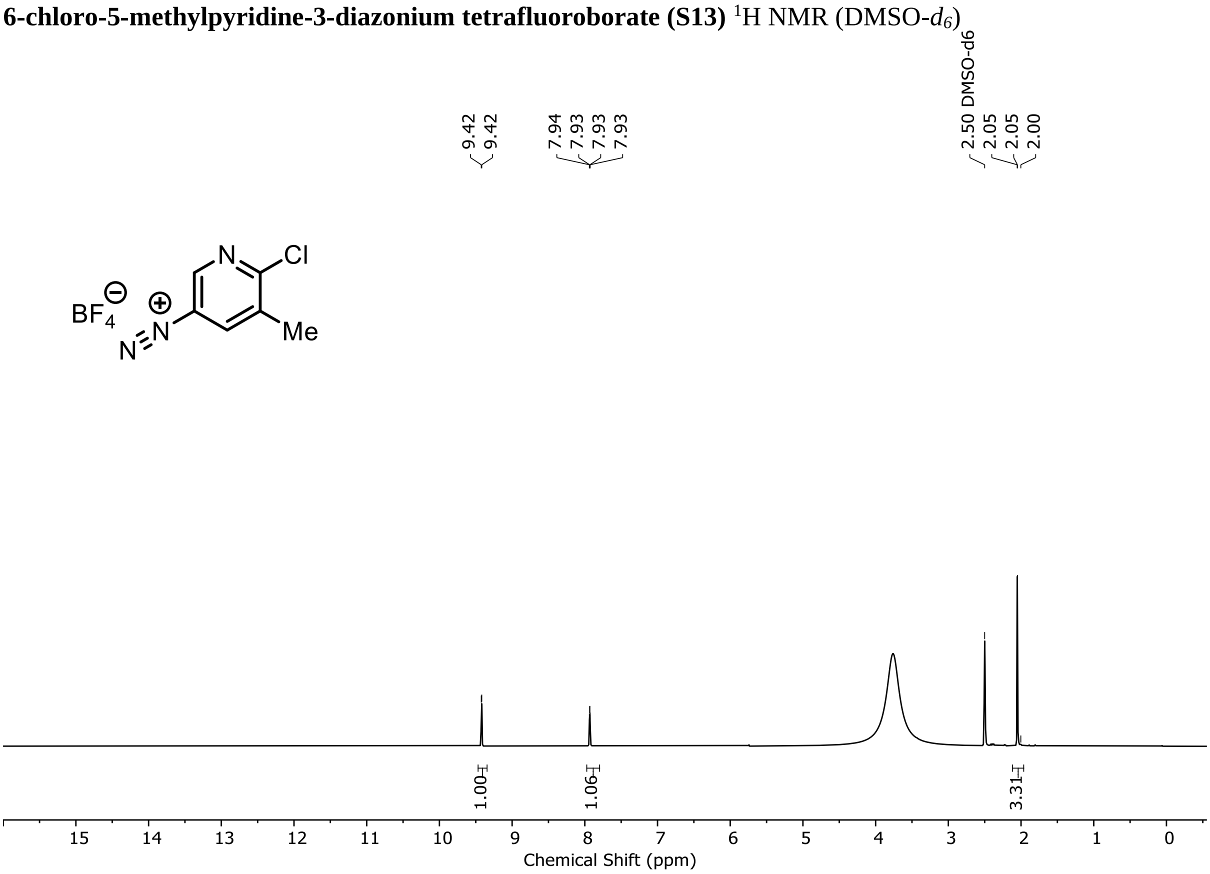

6-chloro-5-methylpyridine-3-diazonium tetrafluoroborate (S13) ^{19}F NMR (DMSO-d_6)

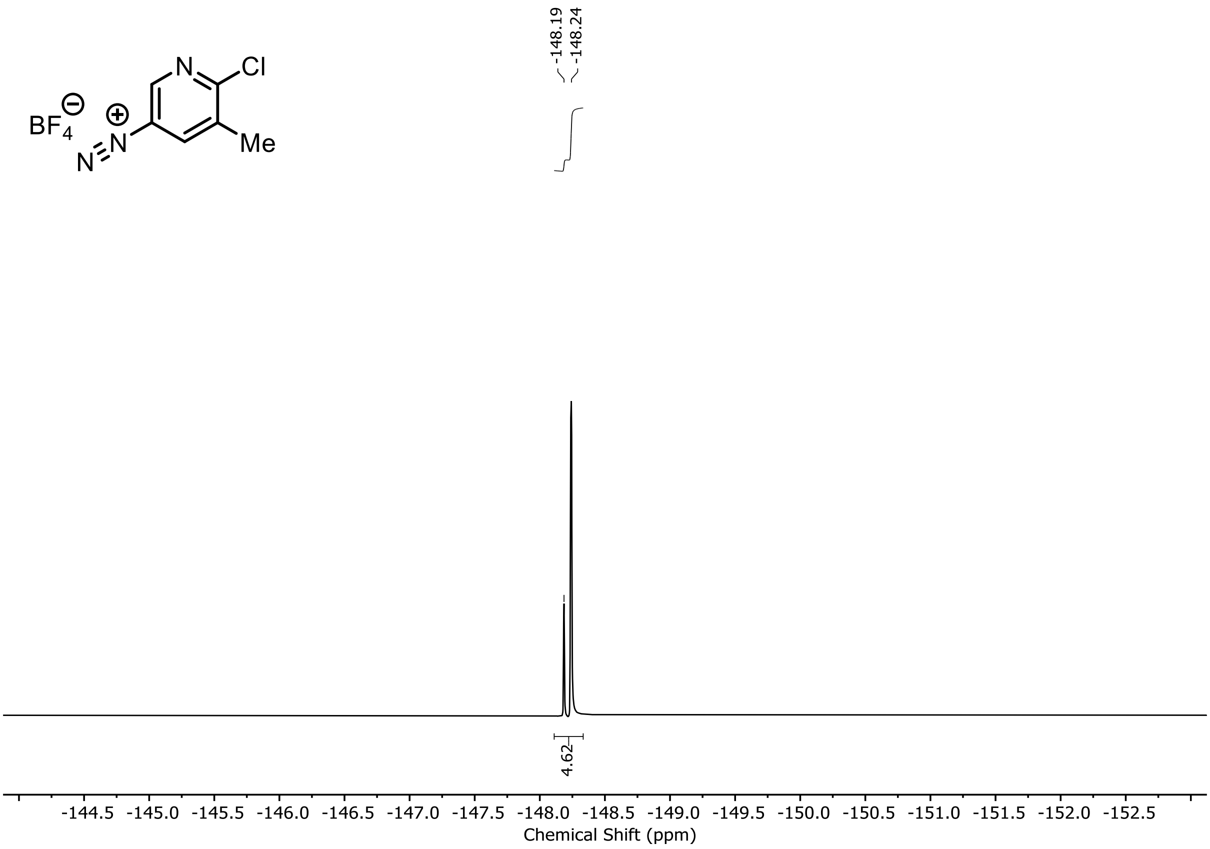

6-chloro-5-methylpyridine-3-diazonium tetrafluoroborate (S13) ^{13}C NMR (DMSO-d_6)

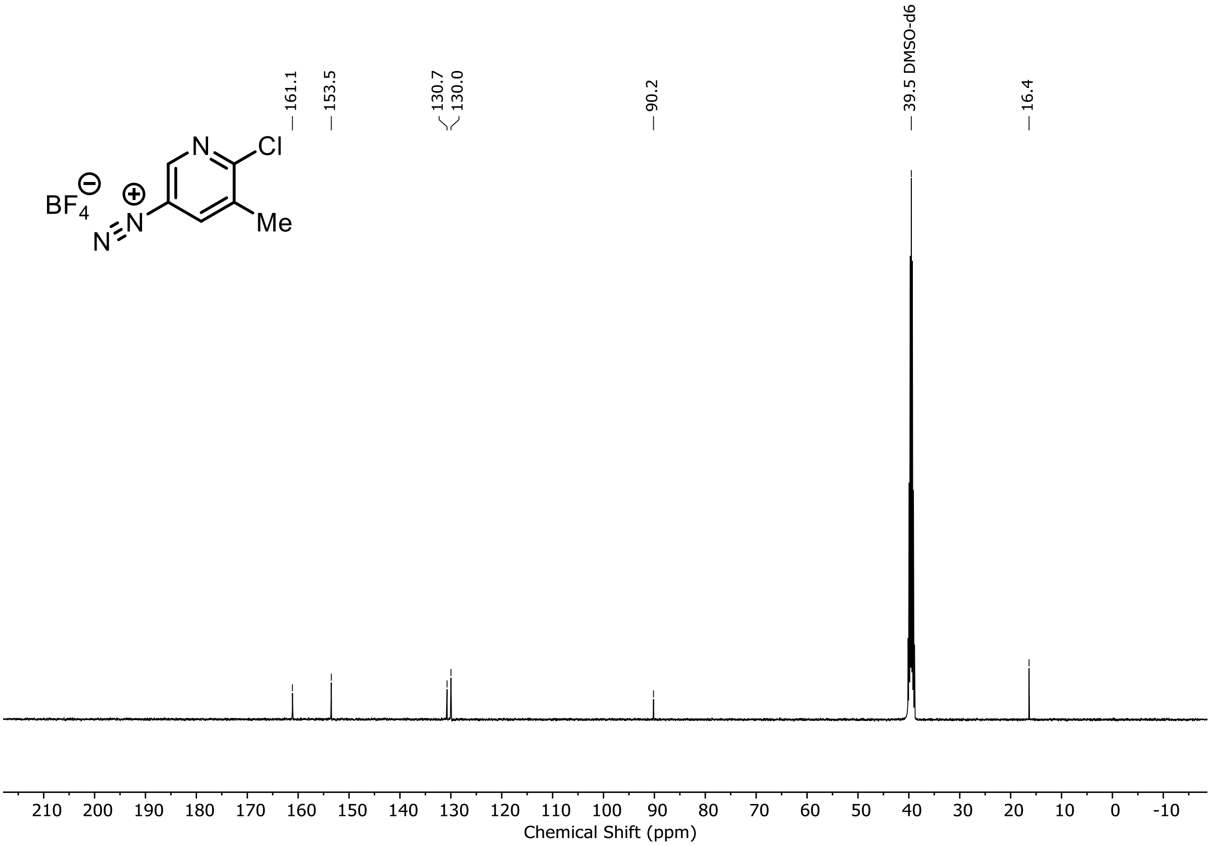

2,4-Dichloro-1-fluorobenzene (2) ^{1}H NMR ($CDCl_3$)

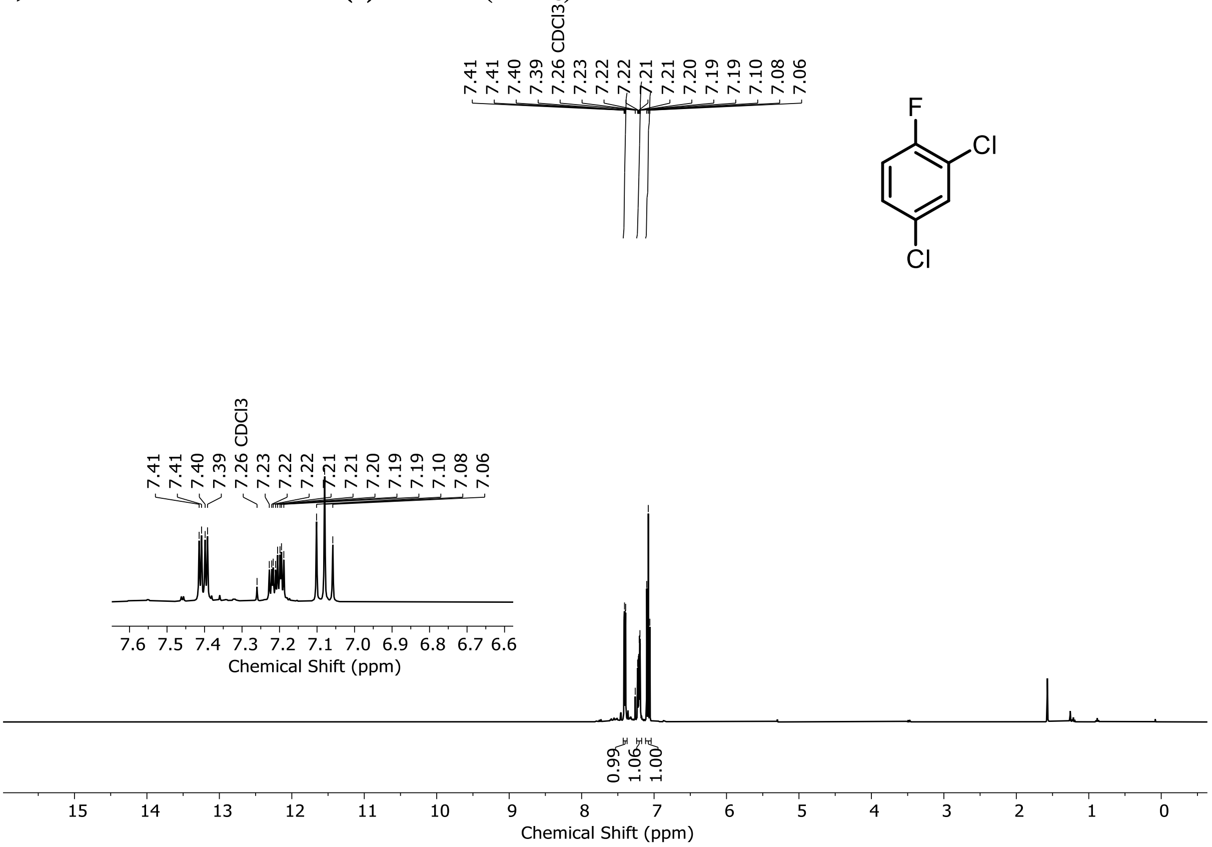

2,4-Dichloro-1-fluorobenzene (2) ^{13}C NMR ($CDCl_3$)

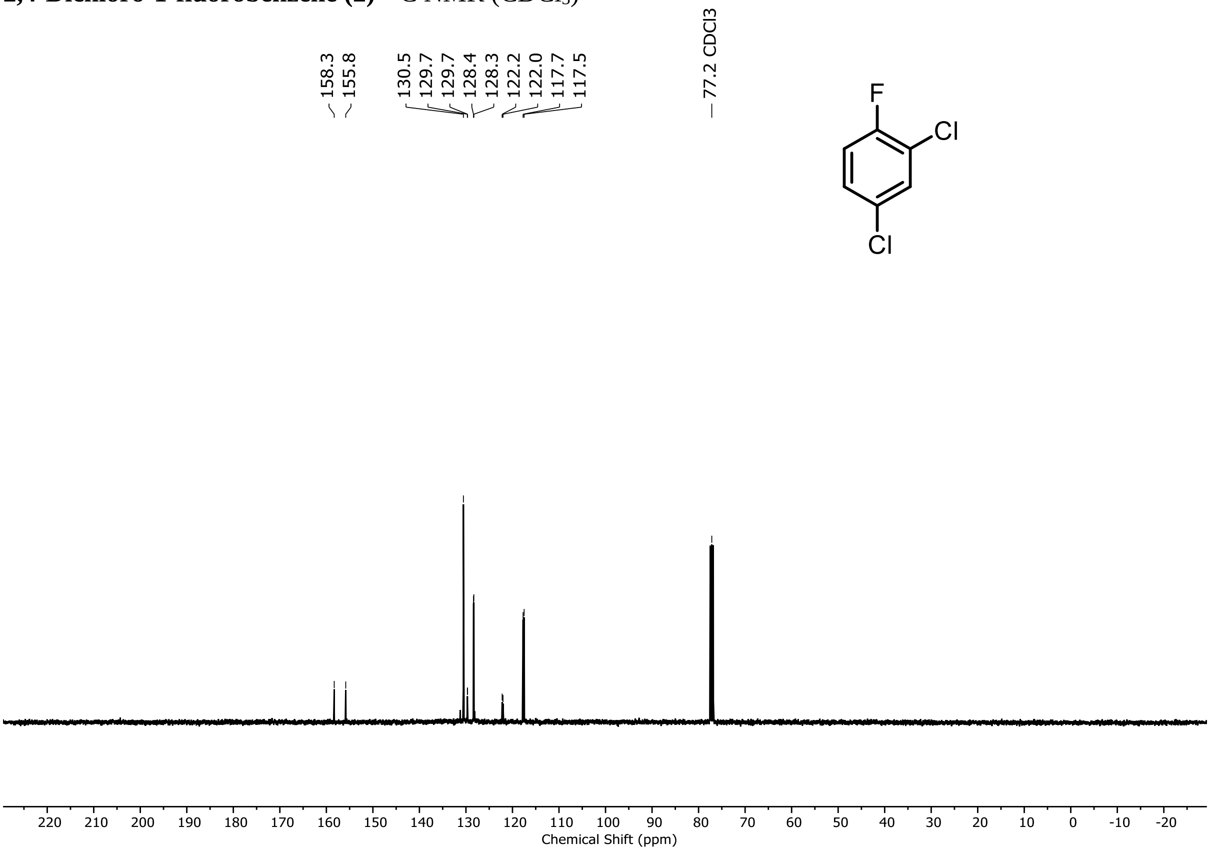

2,4-Dichloro-1-fluorobenzene (2) ^{19}F NMR ($CDCl_3$)

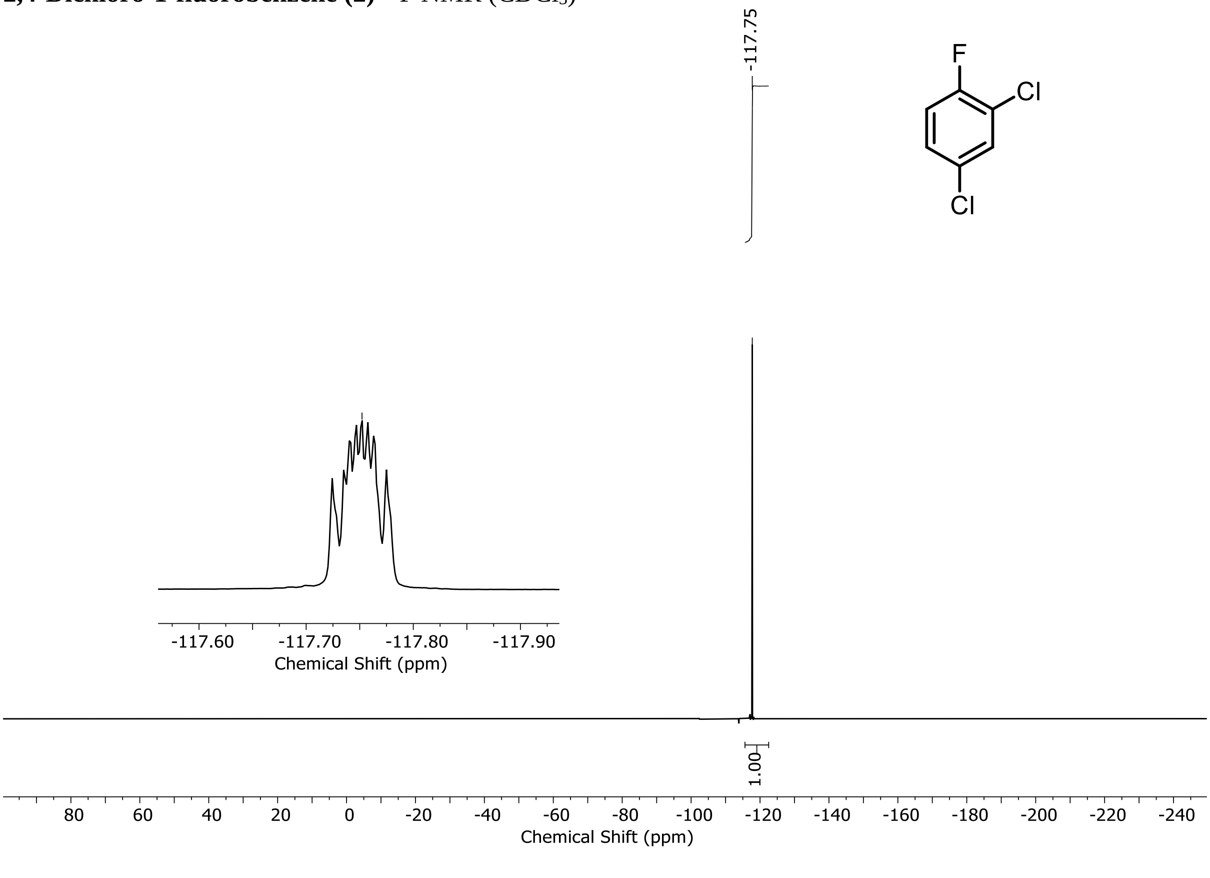

4-Fluorobenzonitrile (3) ^{1}H NMR ($CDCl_3$)

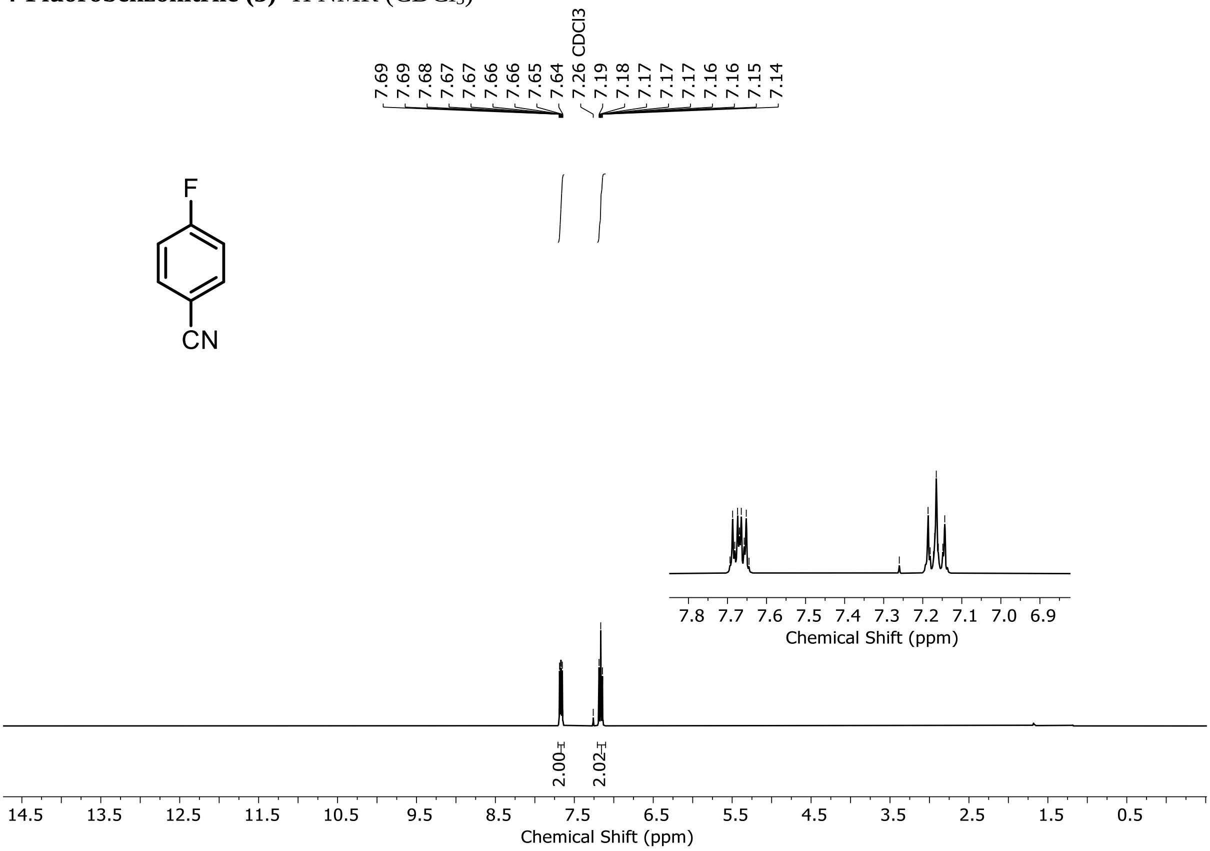

4-Fluorobenzonitrile (3) ^{13}C NMR ($CDCl_3$)

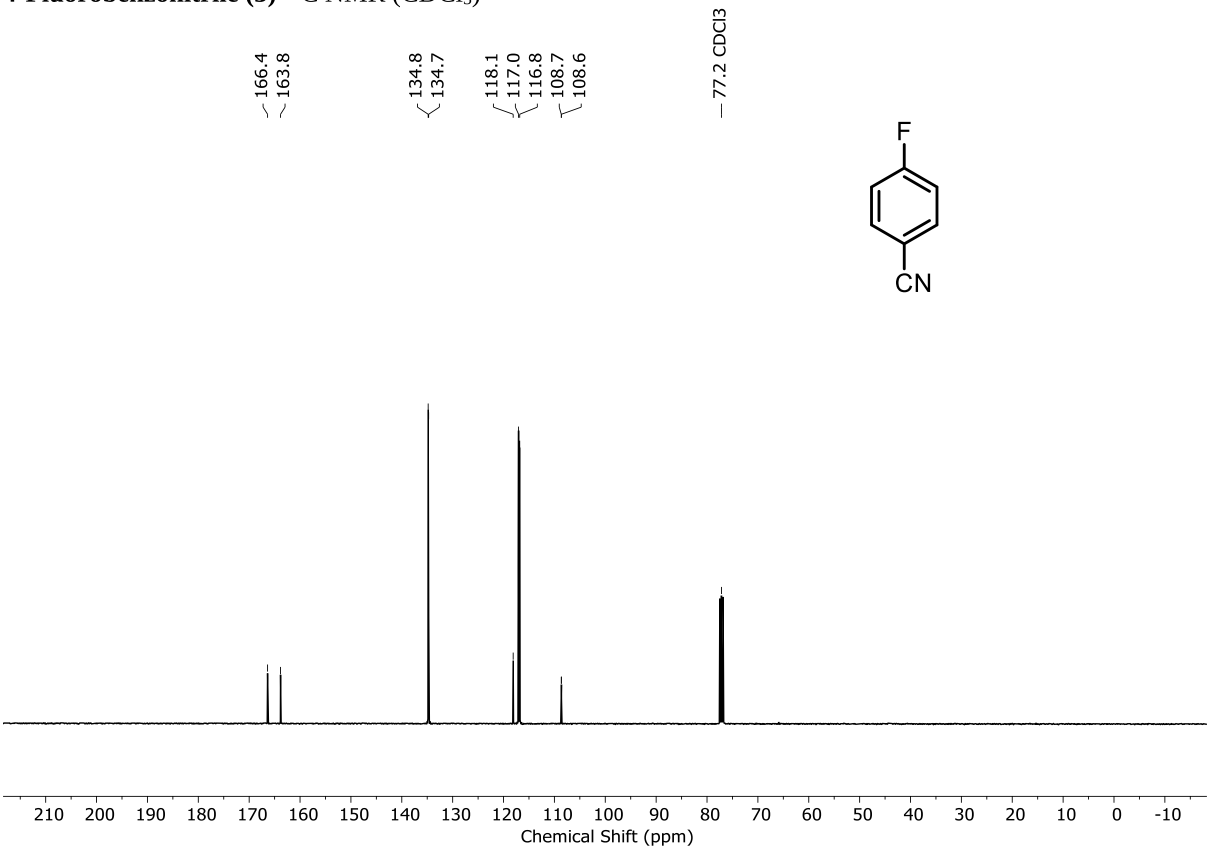

4-Fluorobenzonitrile (3) ^{19}F NMR ($CDCl_3$)

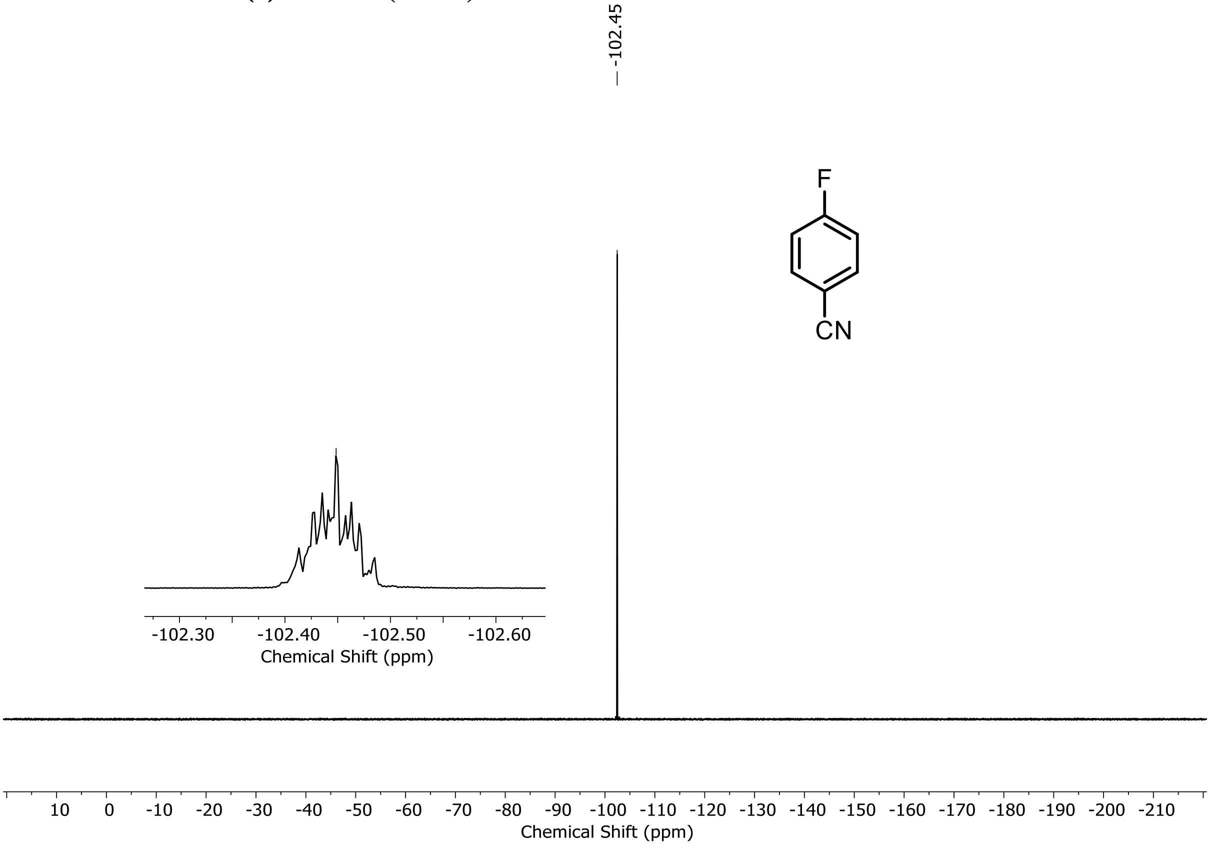

2-Chloro-3-fluoropyridine (4) ^{1}H NMR ($CDCl_3$)

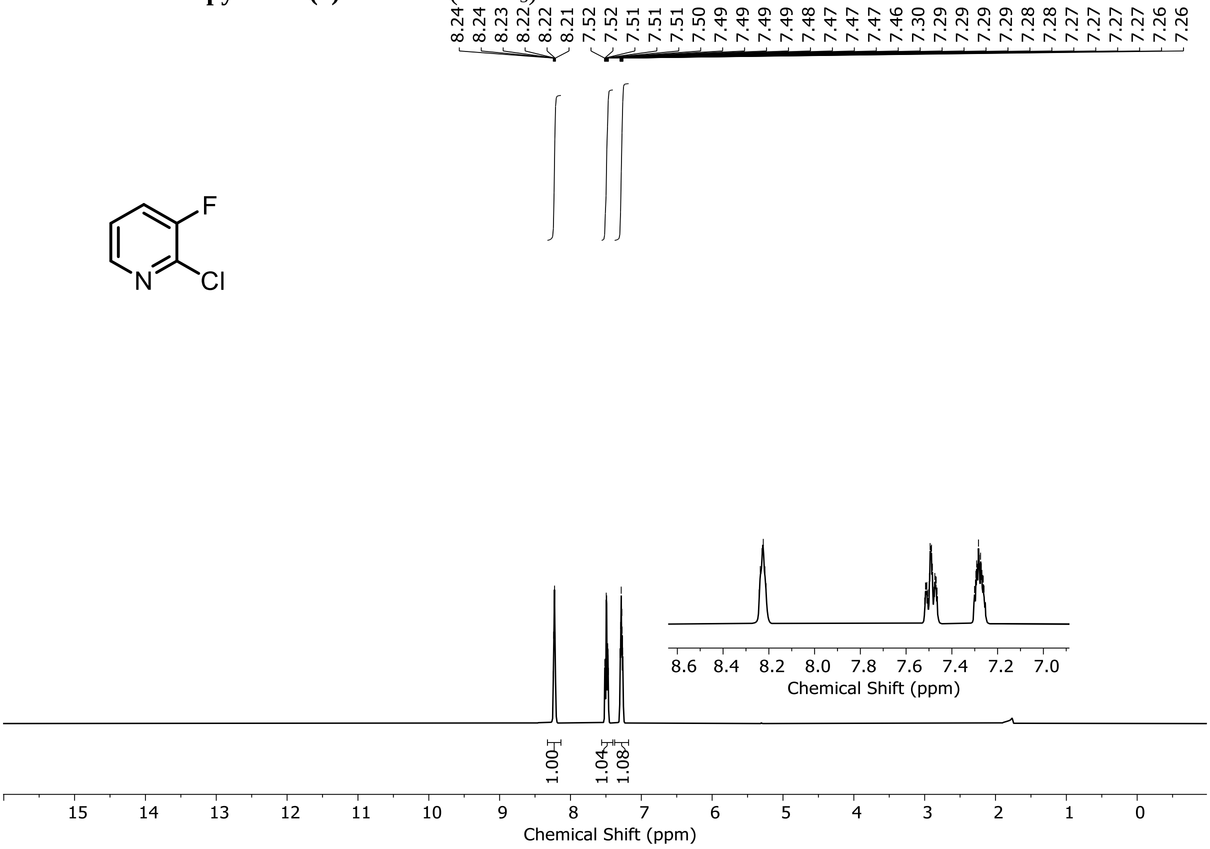

2-Chloro-3-fluoropyridine (4) ^{13}C NMR (CDCl$_3$)

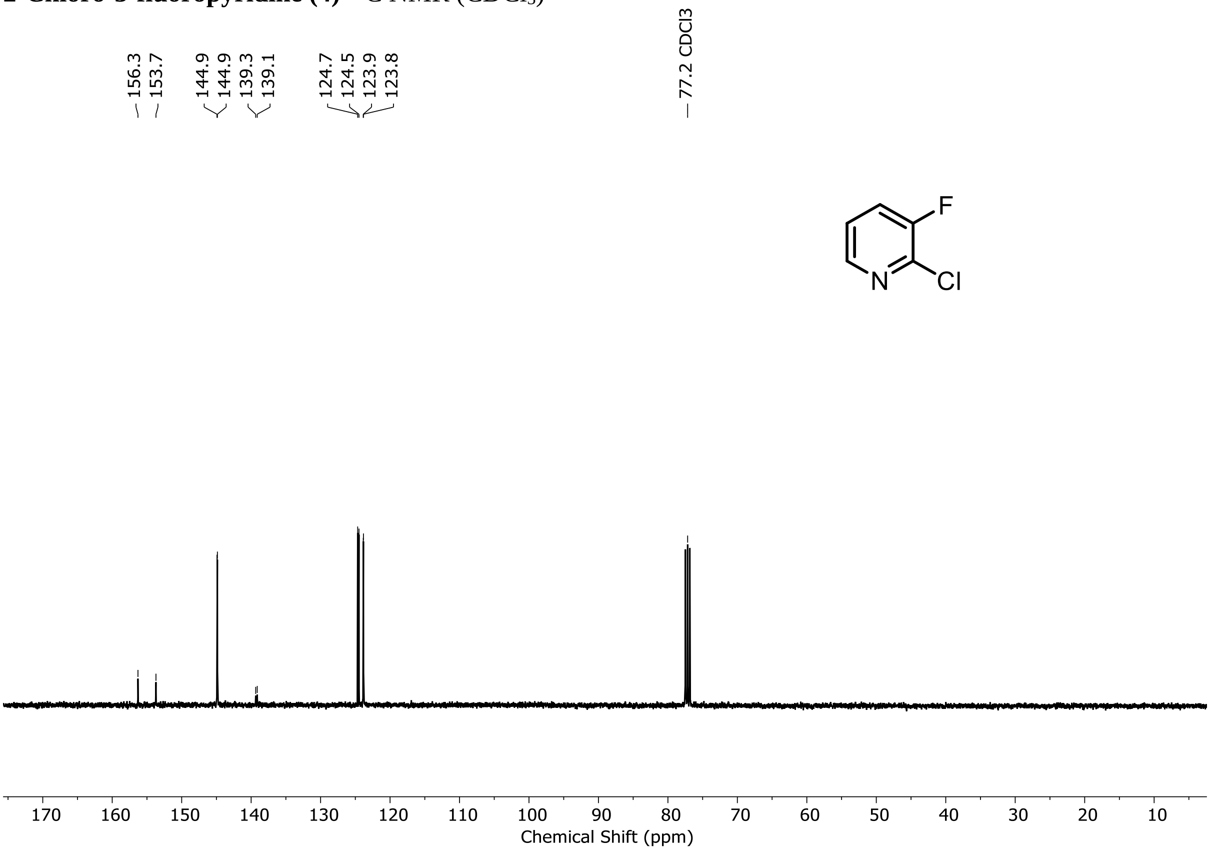

2-Chloro-3-fluoropyridine (4) ^{19}F NMR (CDCl$_3$)

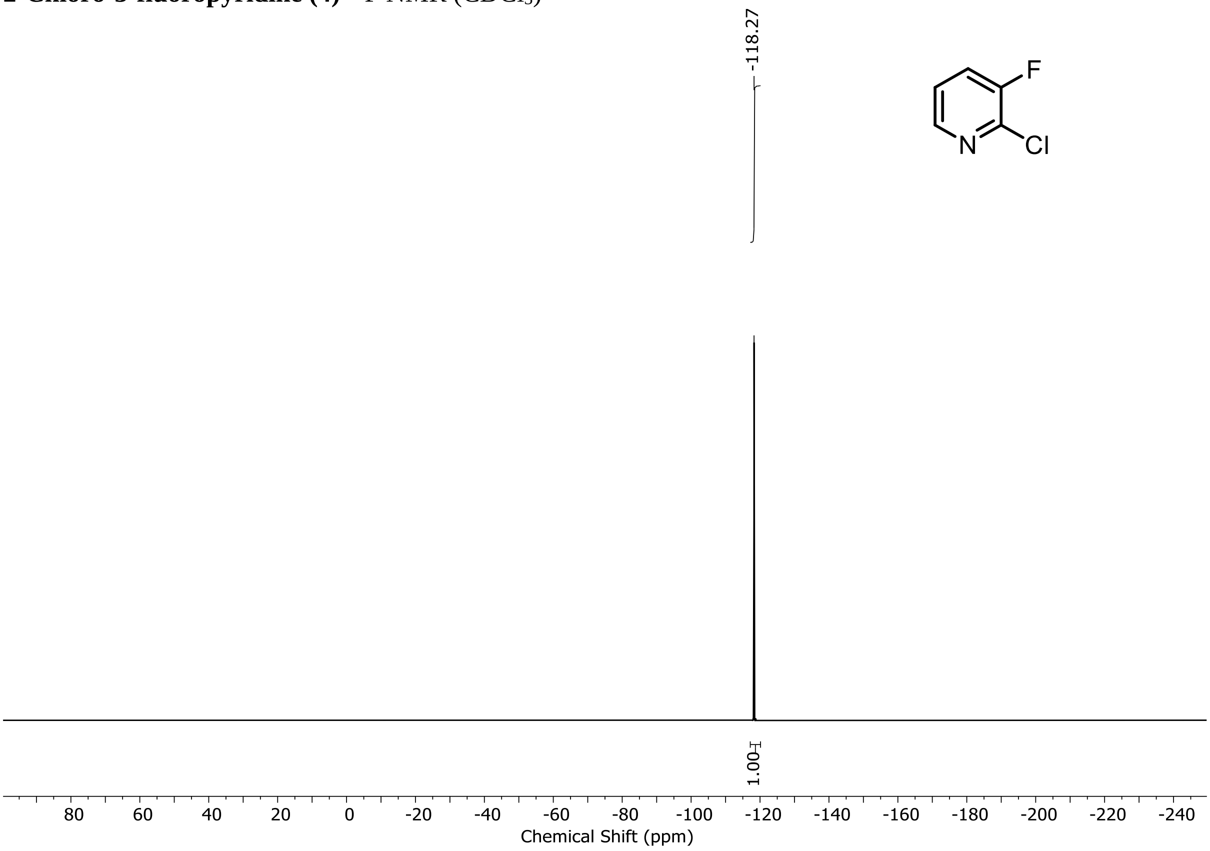

4-Fluoroanisole (5) ^{1}H NMR ($CDCl_3$)

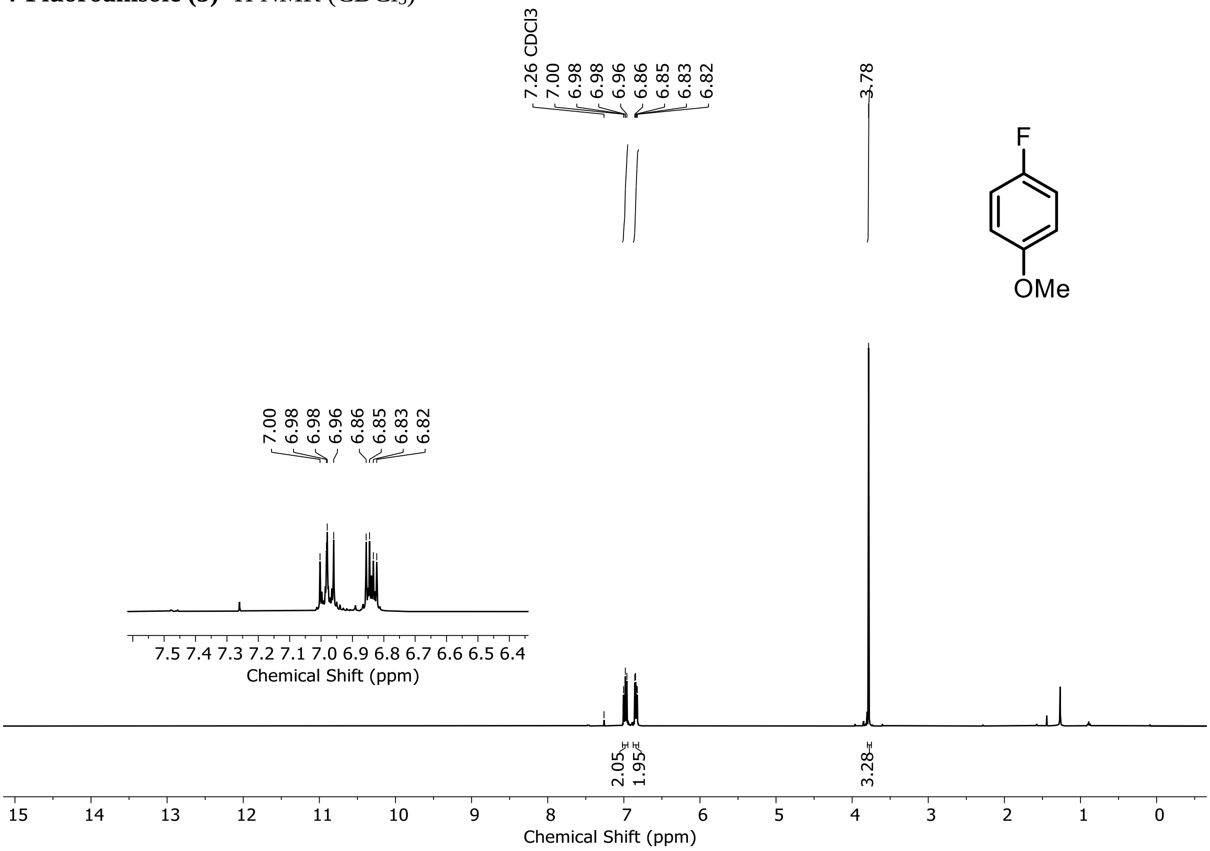

4-Fluoroanisole (5) ^{13}C NMR ($CDCl_3$)

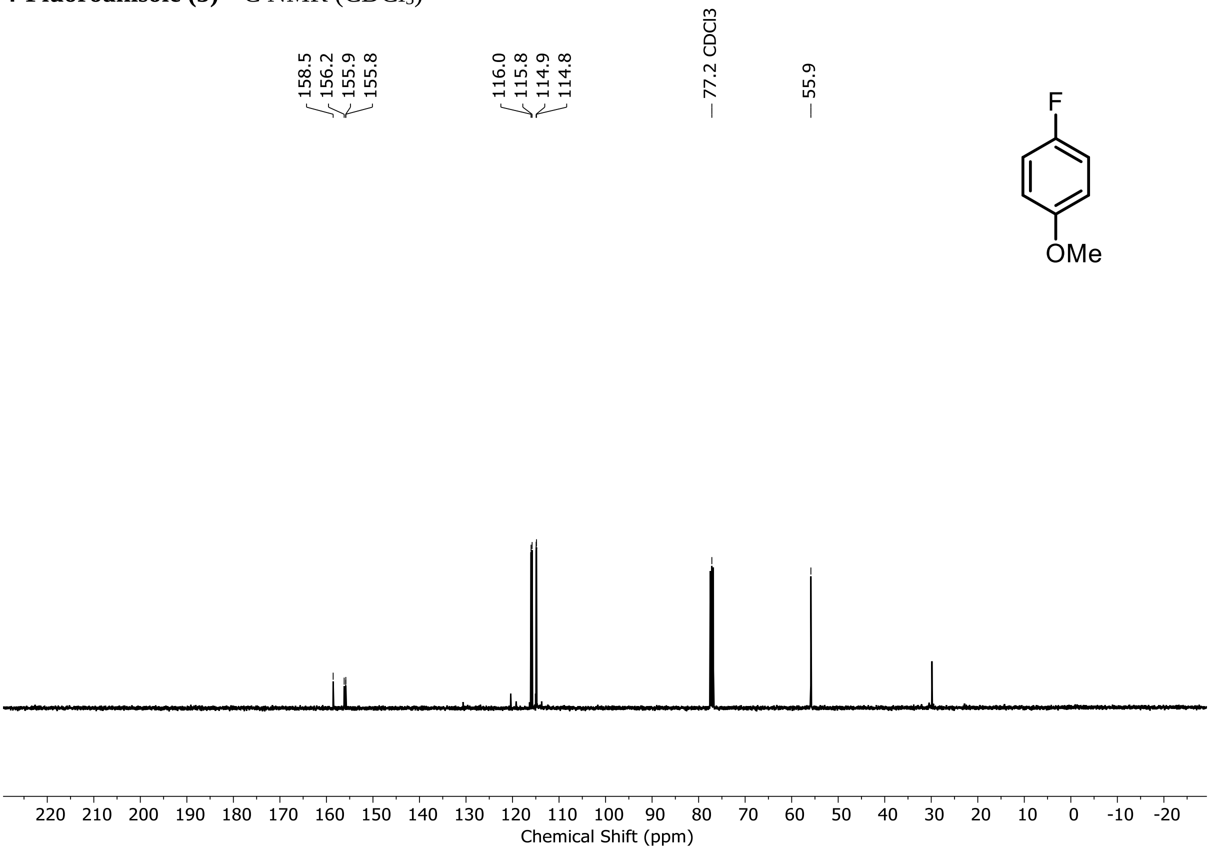

4-Fluoroanisole (5) ^{19}F NMR ($CDCl_3$)

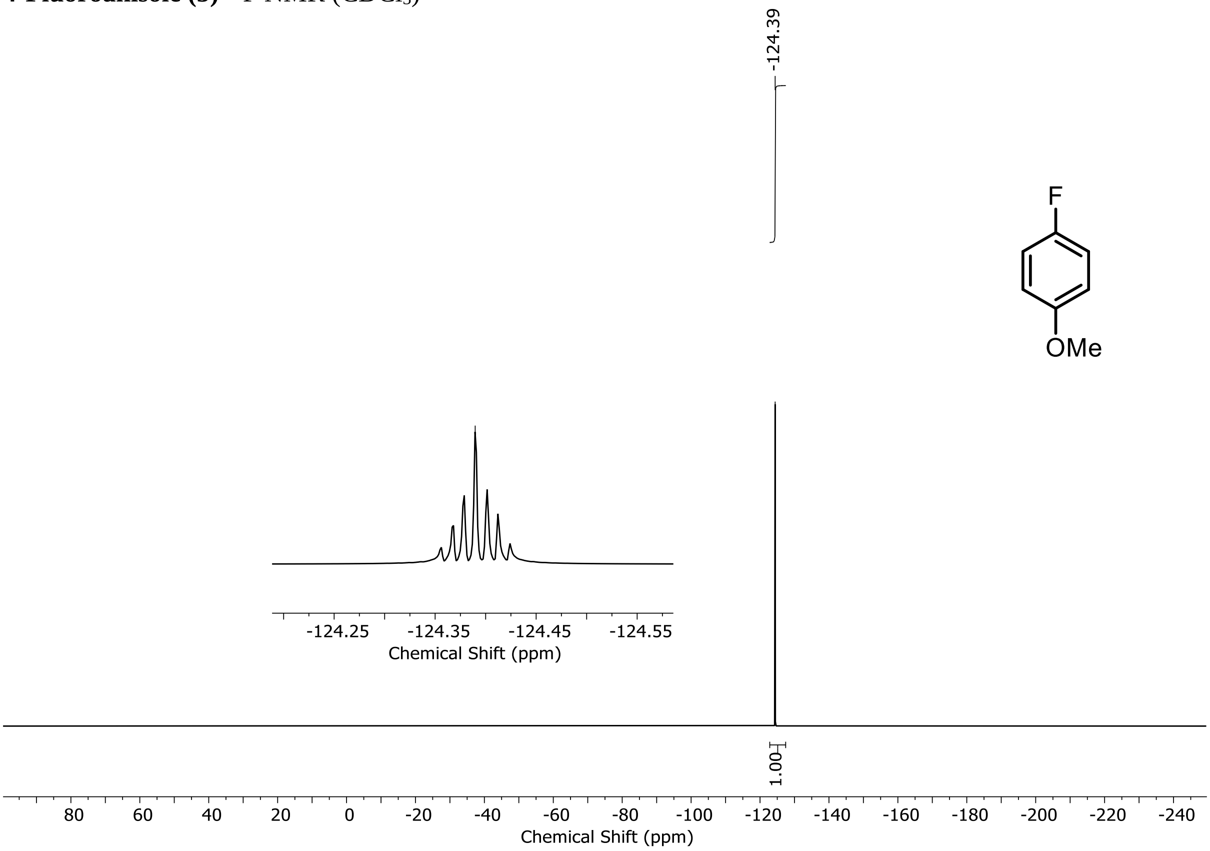

1-(4-fluorophenyl)ethan-1-one (6) ^{1}H NMR ($CDCl_3$)

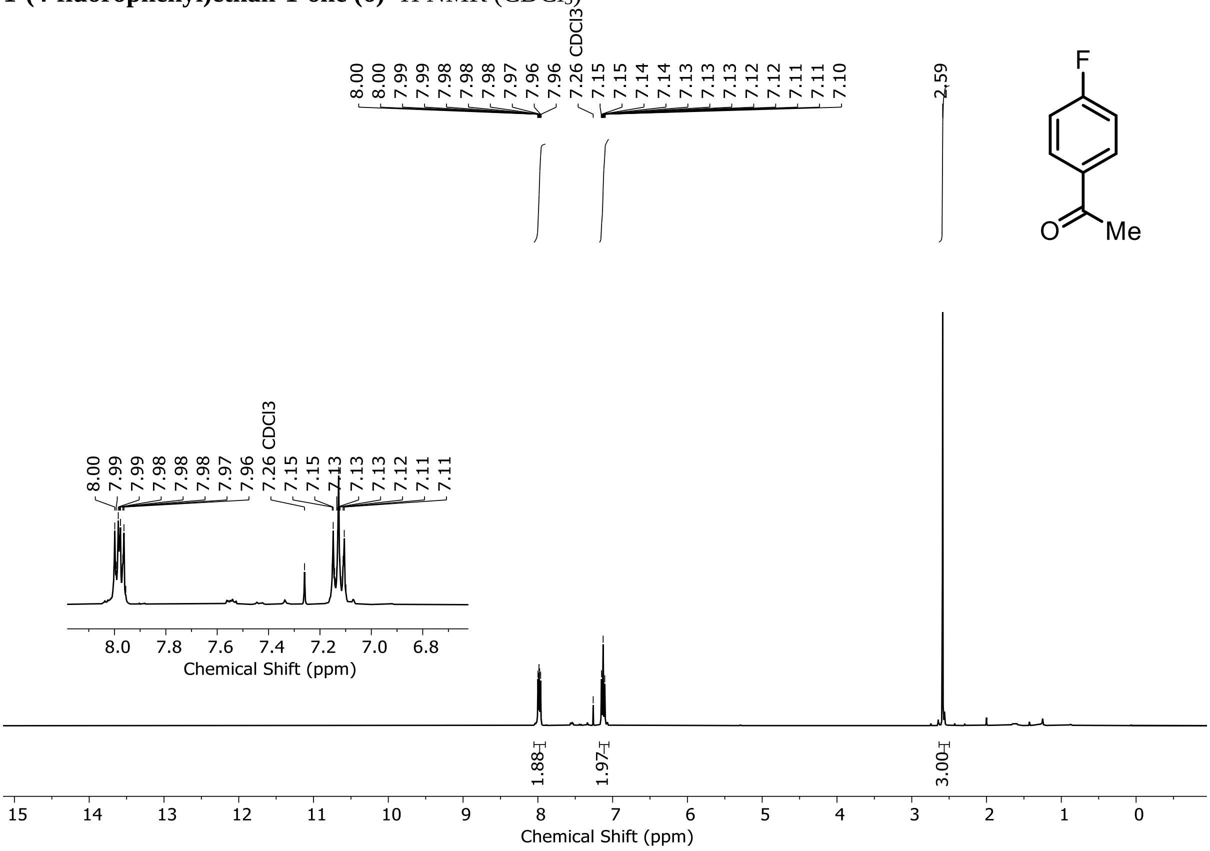

1-(4-fluorophenyl)ethan-1-one (6) ^{13}C NMR ($CDCl_3$)

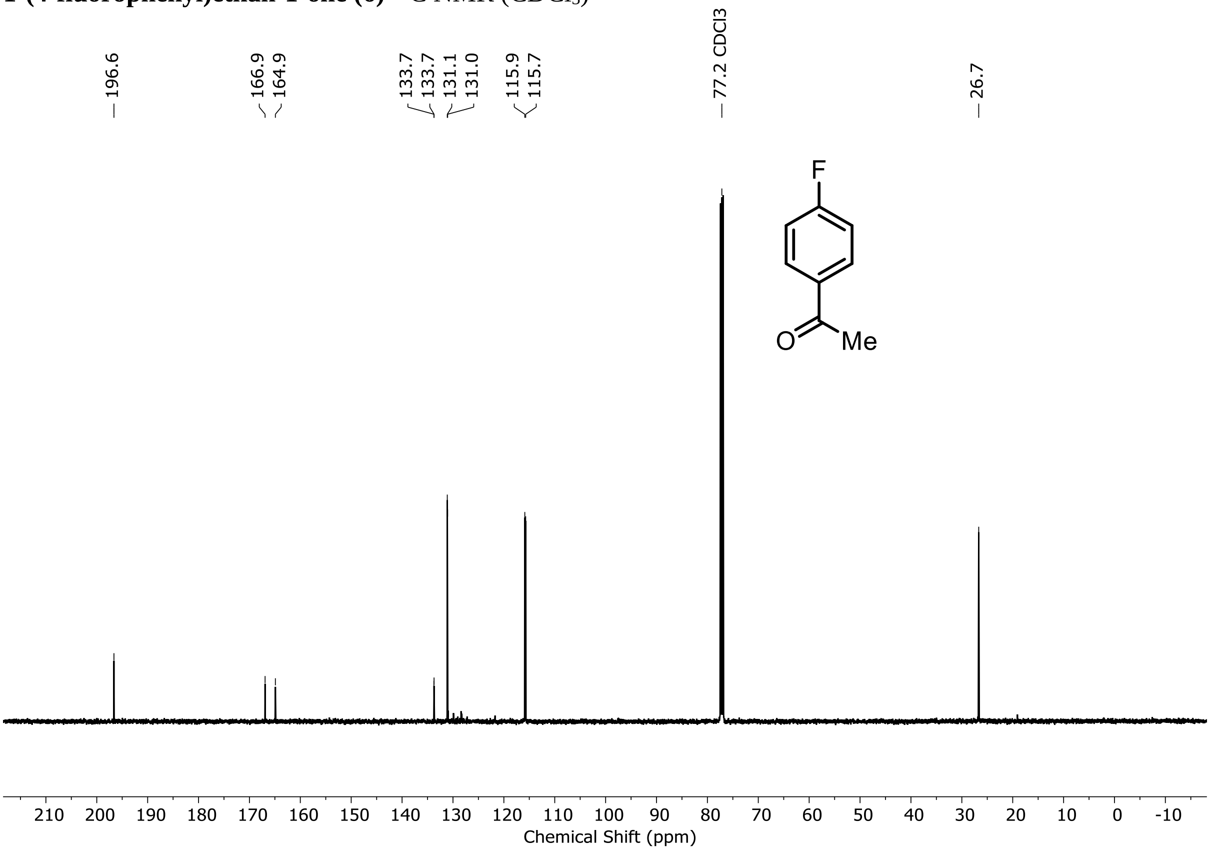

1-(4-fluorophenyl)ethan-1-one (6) ^{19}F NMR ($CDCl_3$)

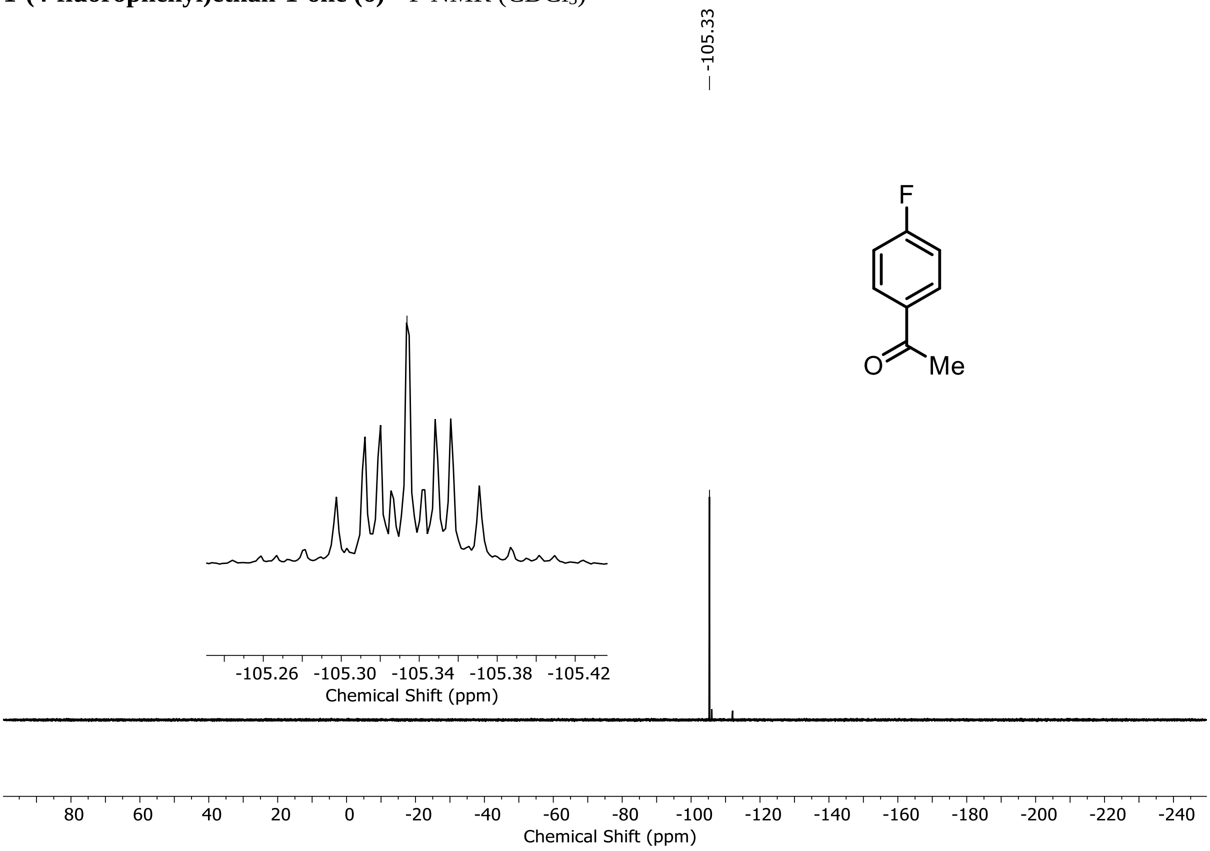

Methyl 4-fluorobenzoate (7) ^{1}H NMR ($CDCl_3$)

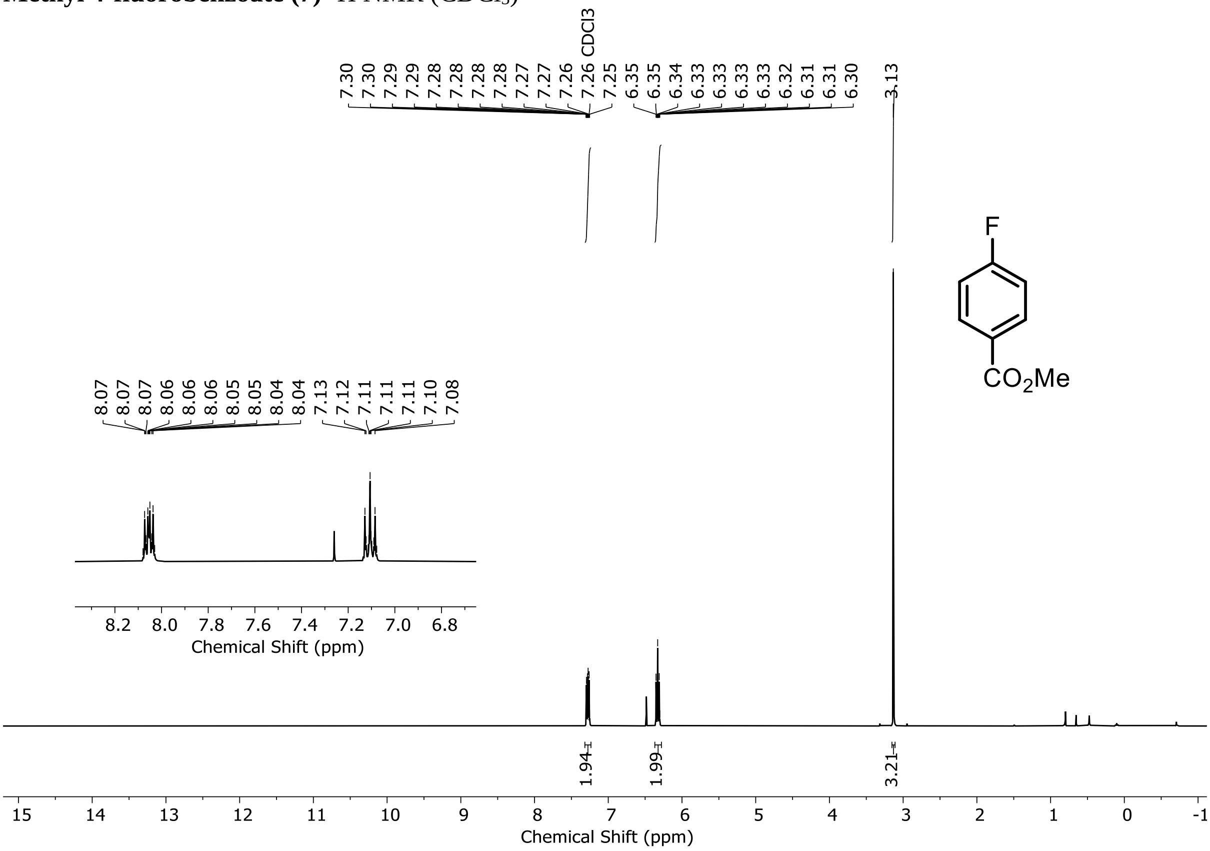

Methyl 4-fluorobenzoate (7) ^{13}C NMR ($CDCl_3$)

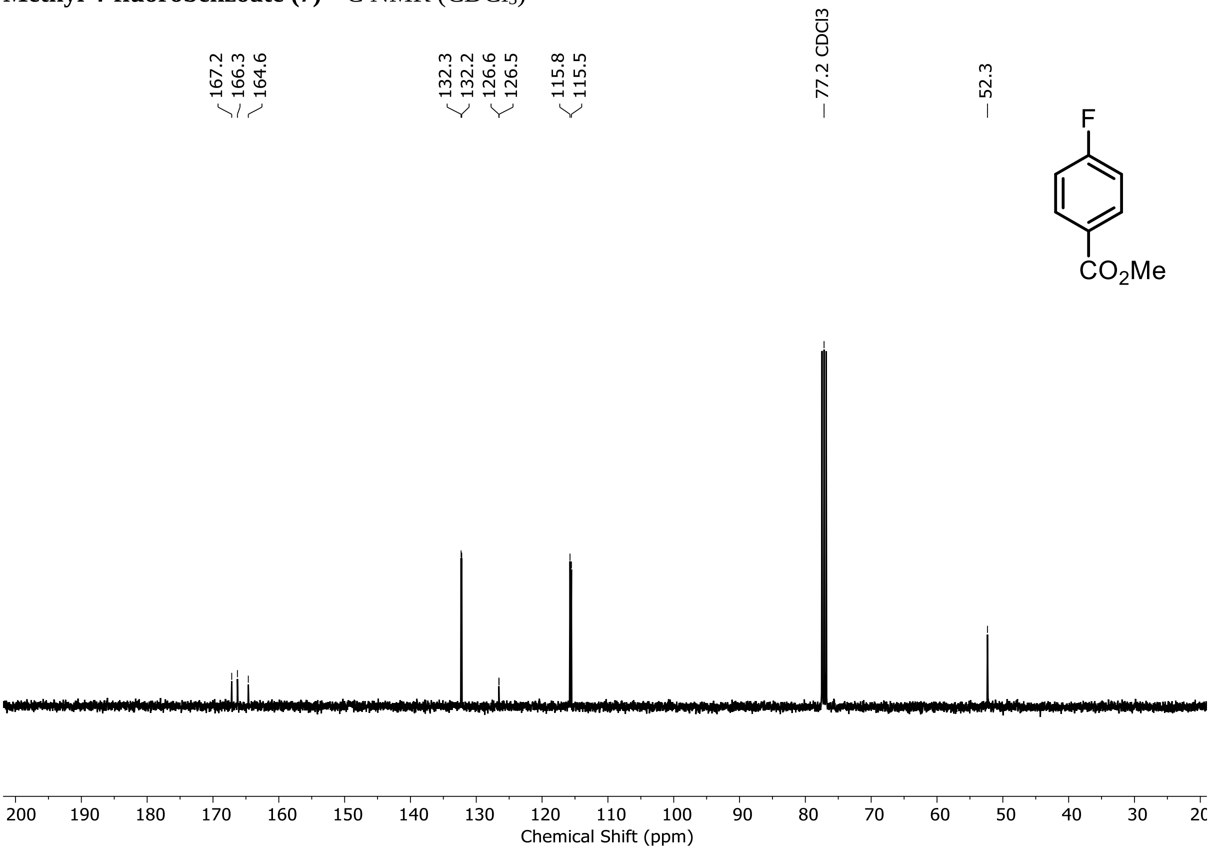

Methyl 4-fluorobenzoate (7) ^{19}F NMR ($CDCl_3$)

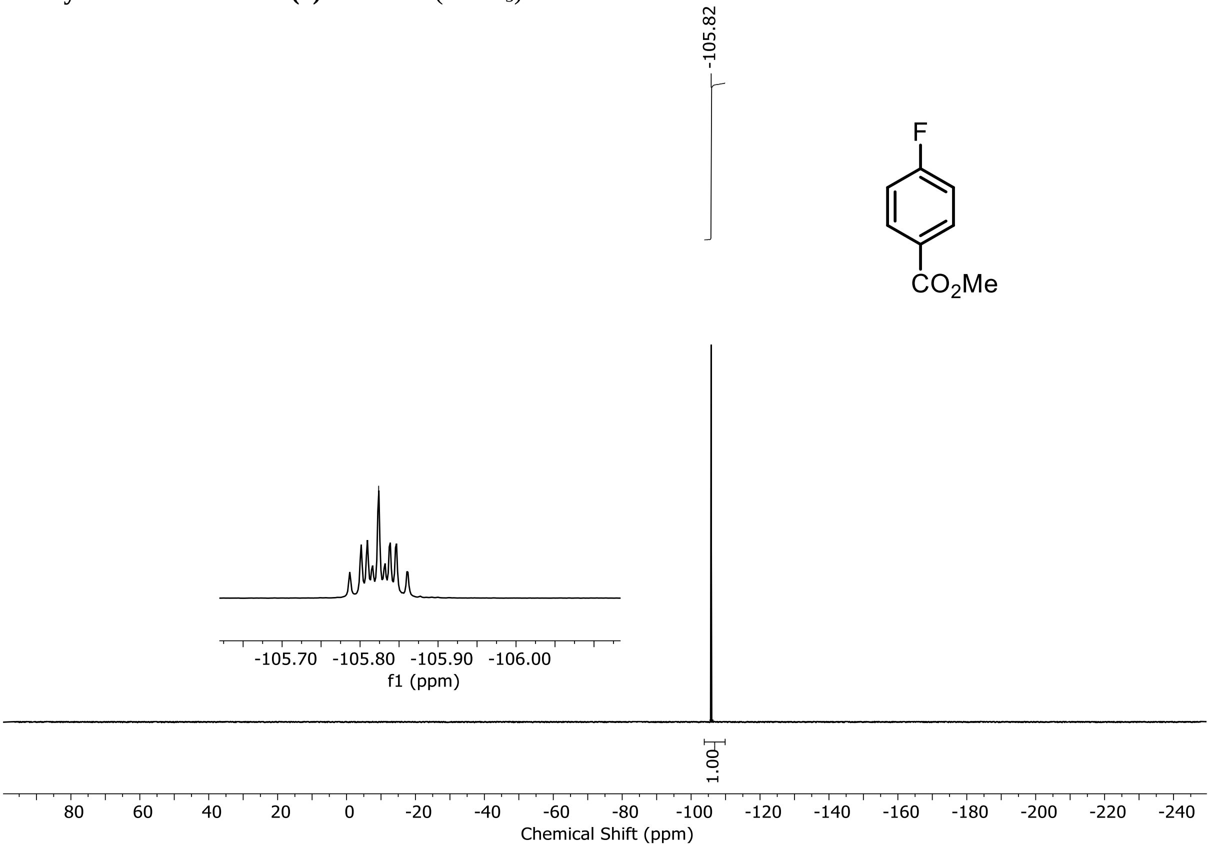

2,5-Dichloro-3-fluoropyridine (8) ^{1}H NMR ($CDCl_3$)

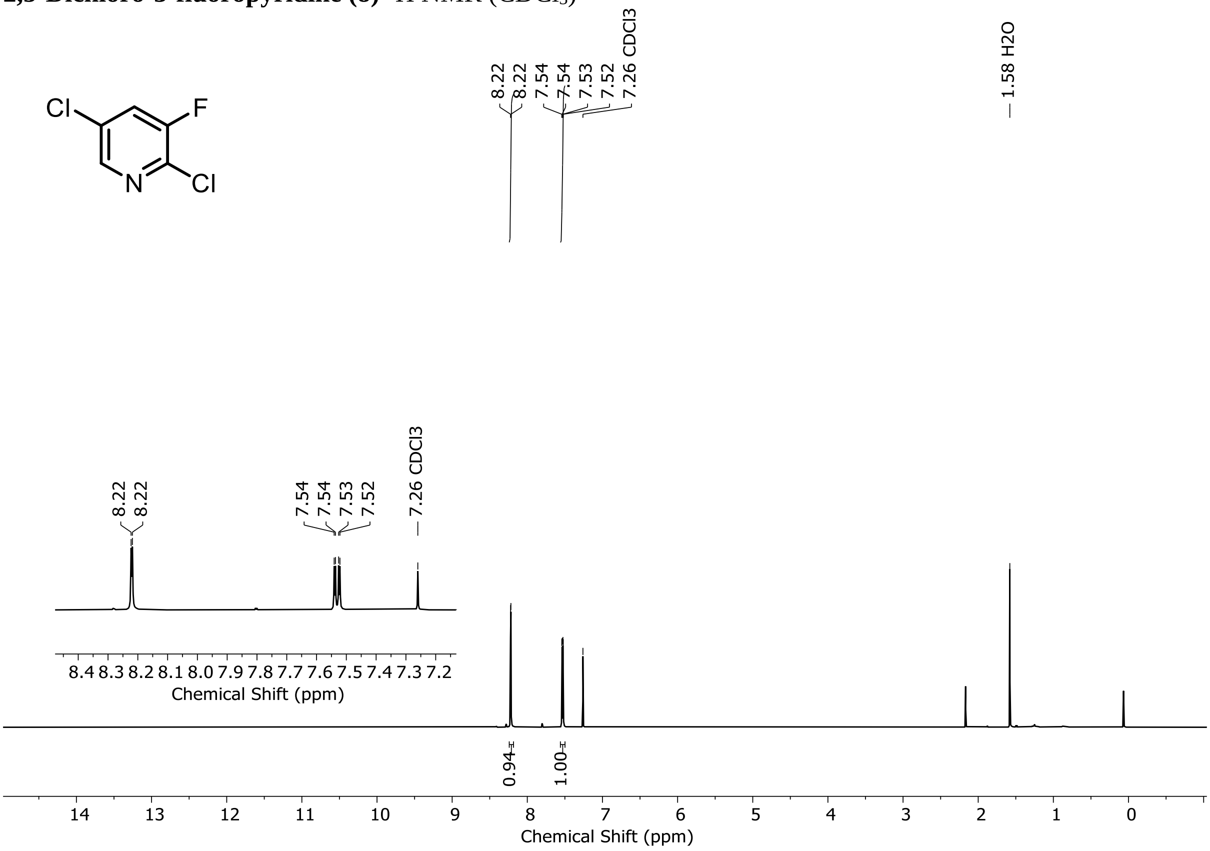

2,5-Dichloro-3-fluoropyridine (8) ^{13}C NMR ($CDCl_3$)

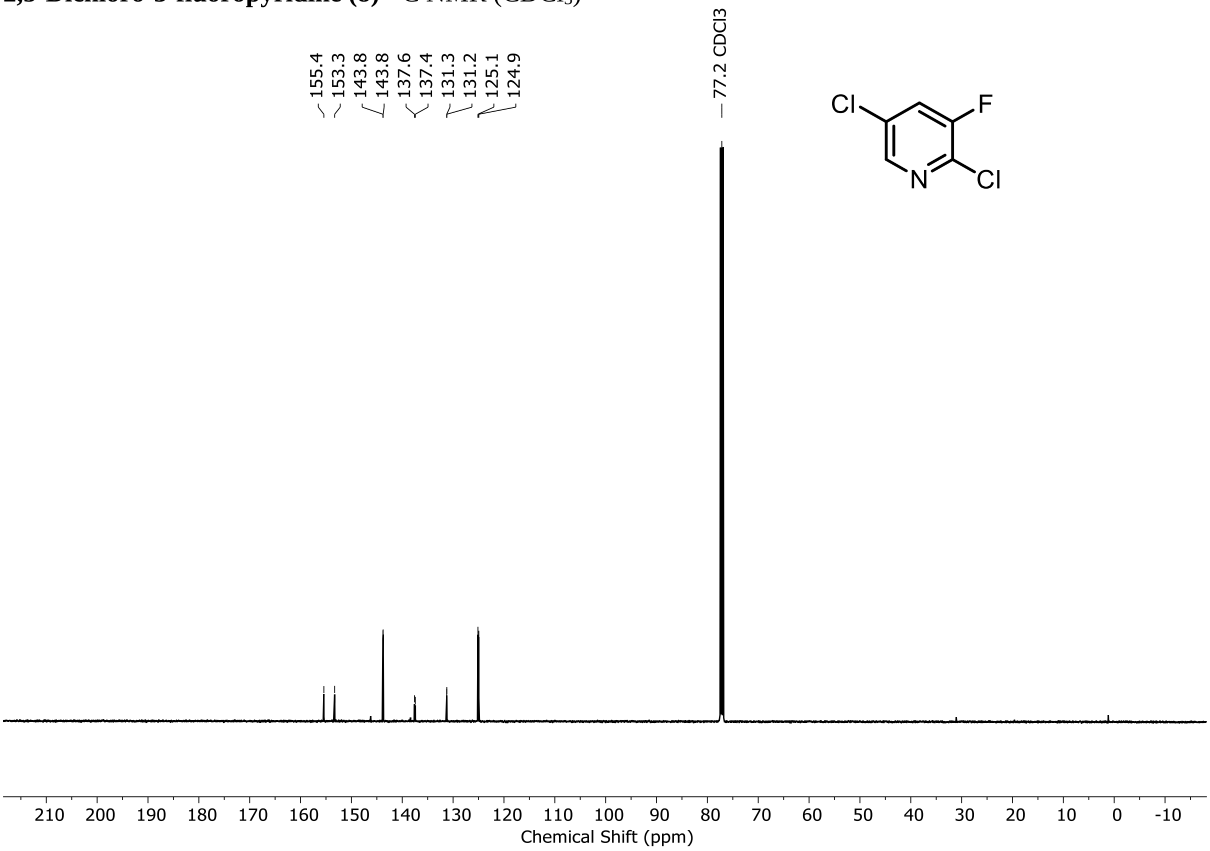

2,5-Dichloro-3-fluoropyridine (8) ^{19}F NMR ($CDCl_3$)

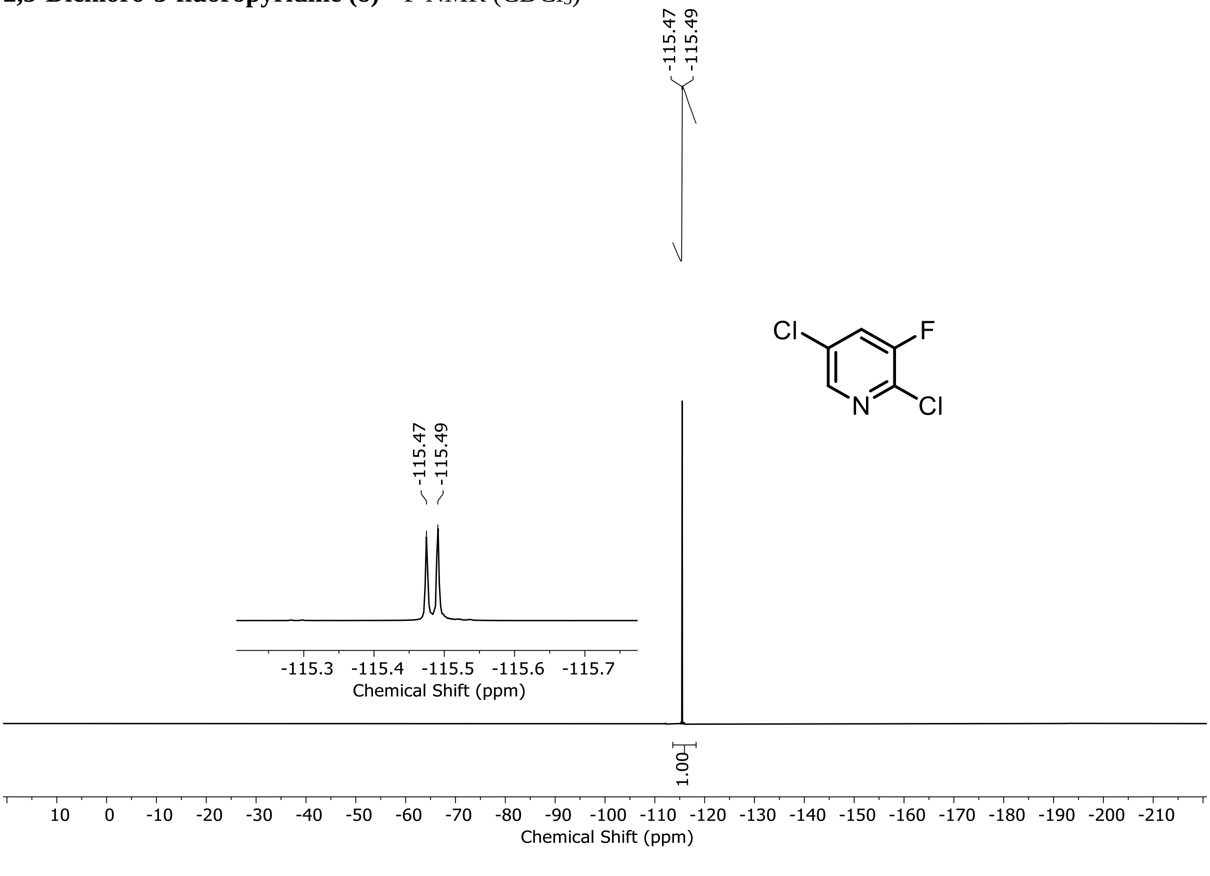

4-Bromo-2-fluoro-1,1'-biphenyl (10) ^{1}H NMR ($CDCl_3$)

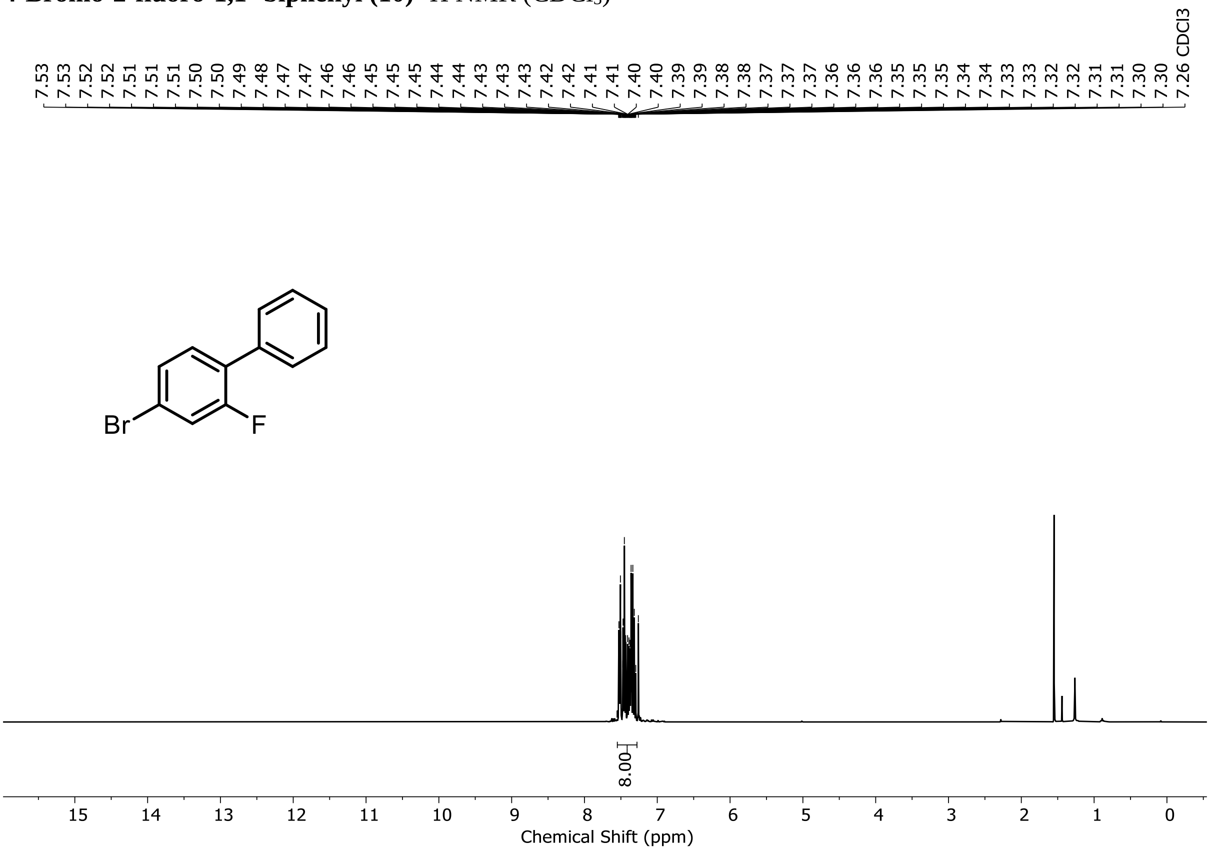

4-Bromo-2-fluoro-1,1'-biphenyl (10) ^{13}C NMR ($CDCl_3$)

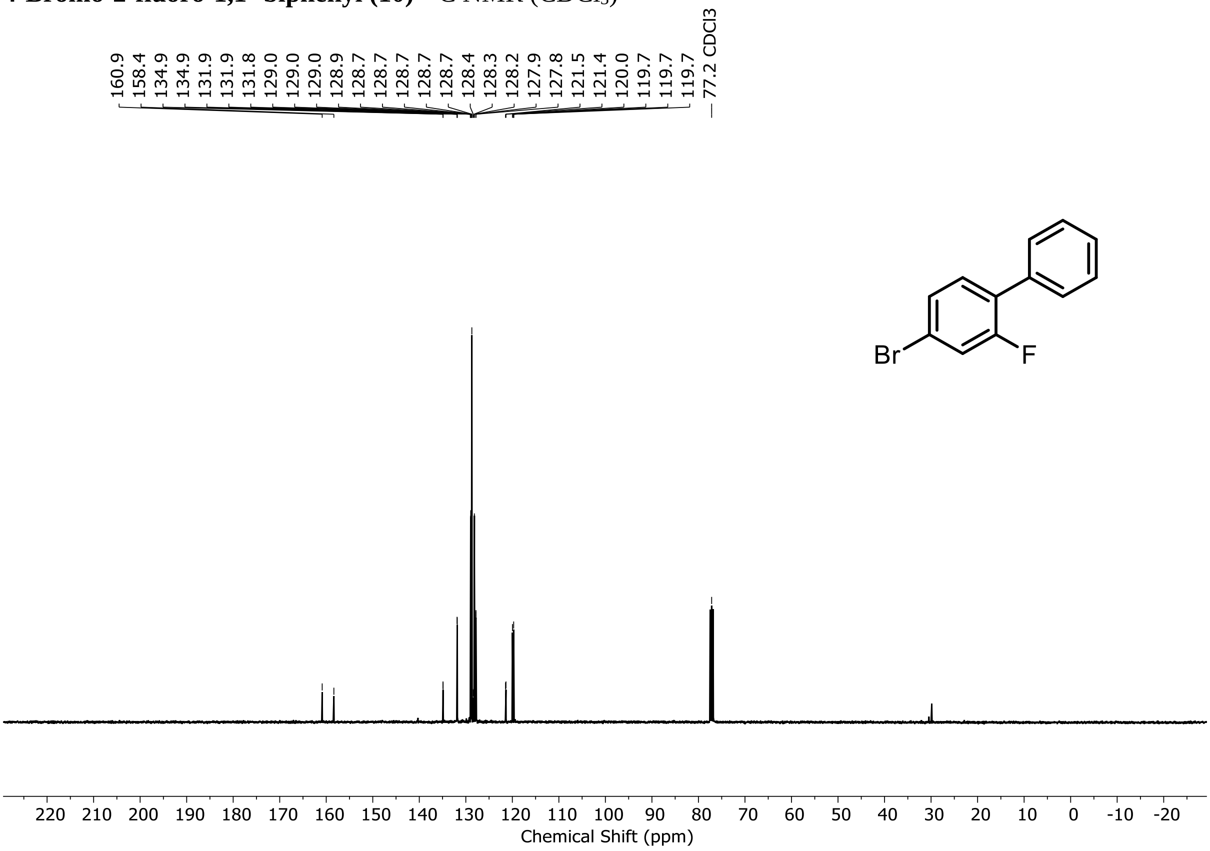

4-Bromo-2-fluoro-1,1'-biphenyl (10) ^{19}F NMR ($CDCl_3$)

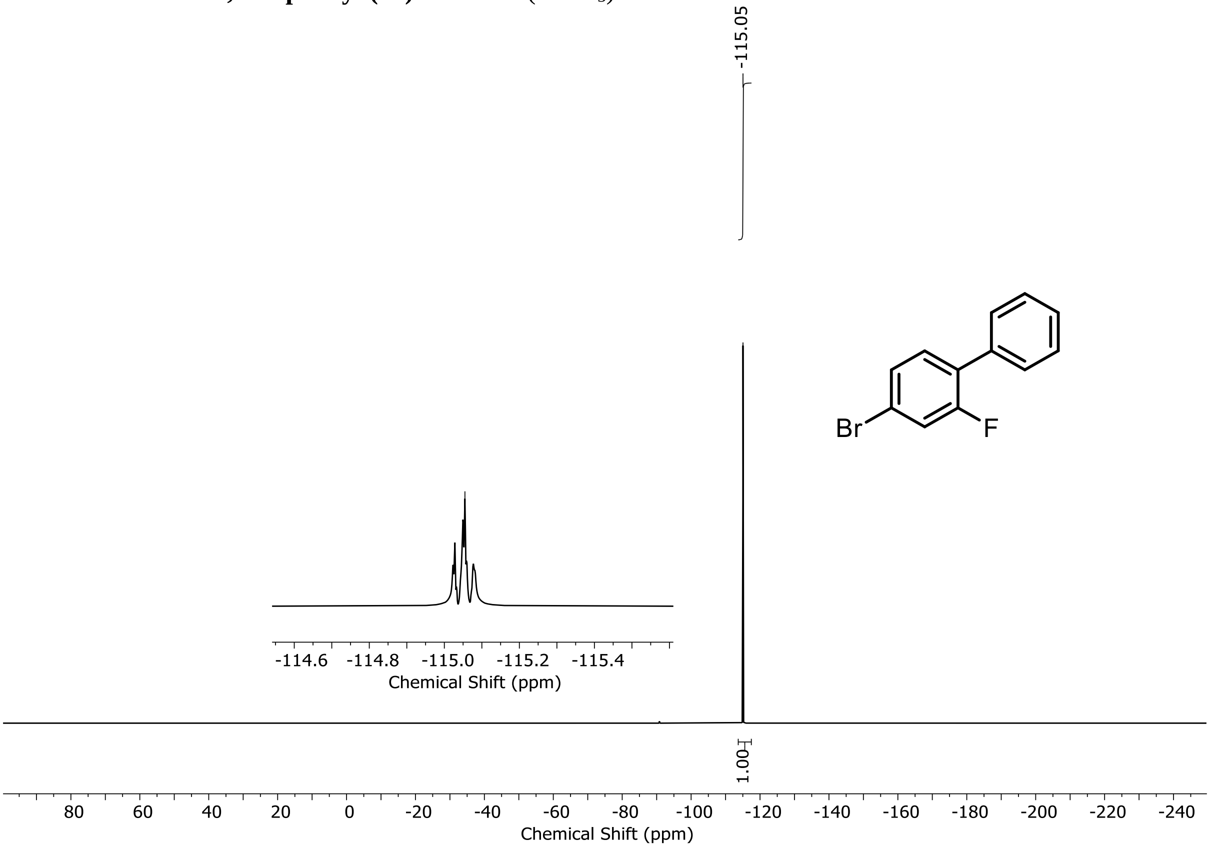

Bis(4-fluorophenyl)methanone (11) ^{1}H NMR ($CDCl_3$)

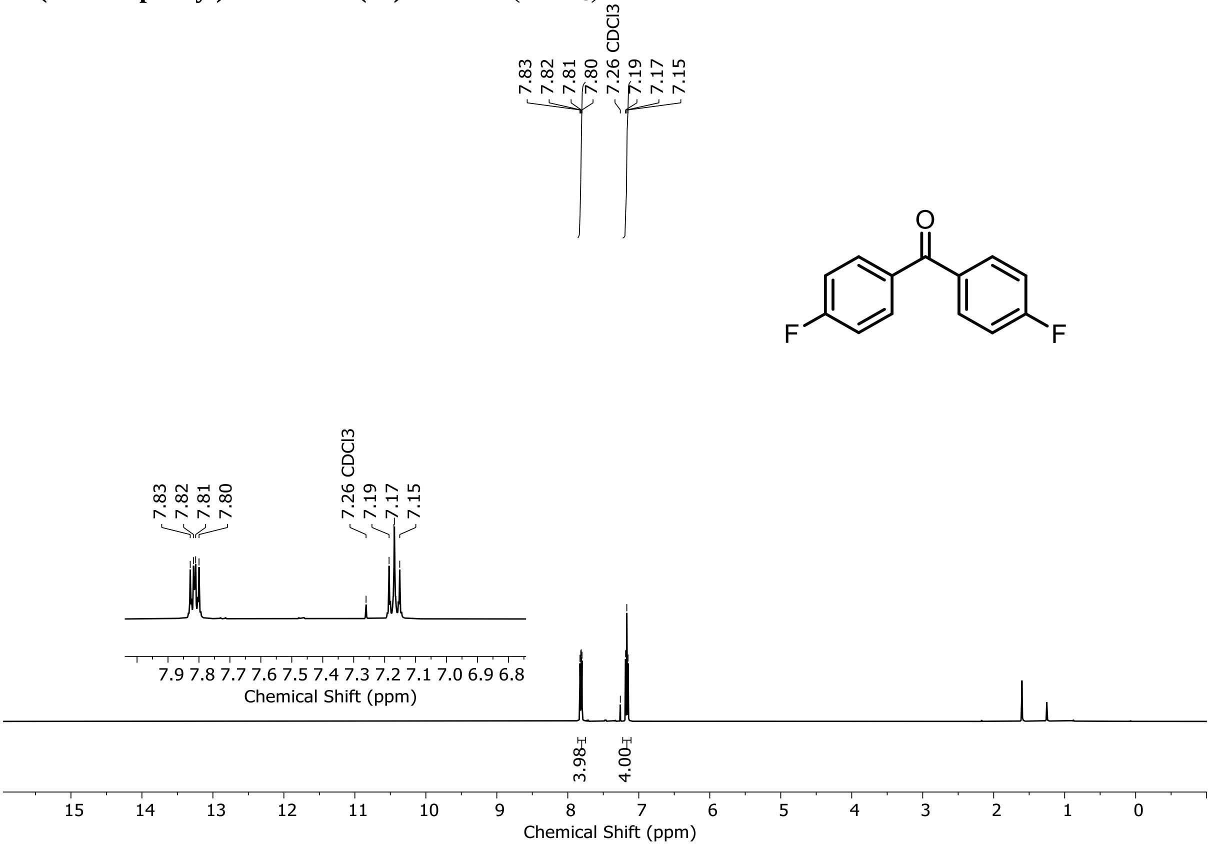

Bis(4-fluorophenyl)methanone (11) ^{13}C NMR ($CDCl_3$)

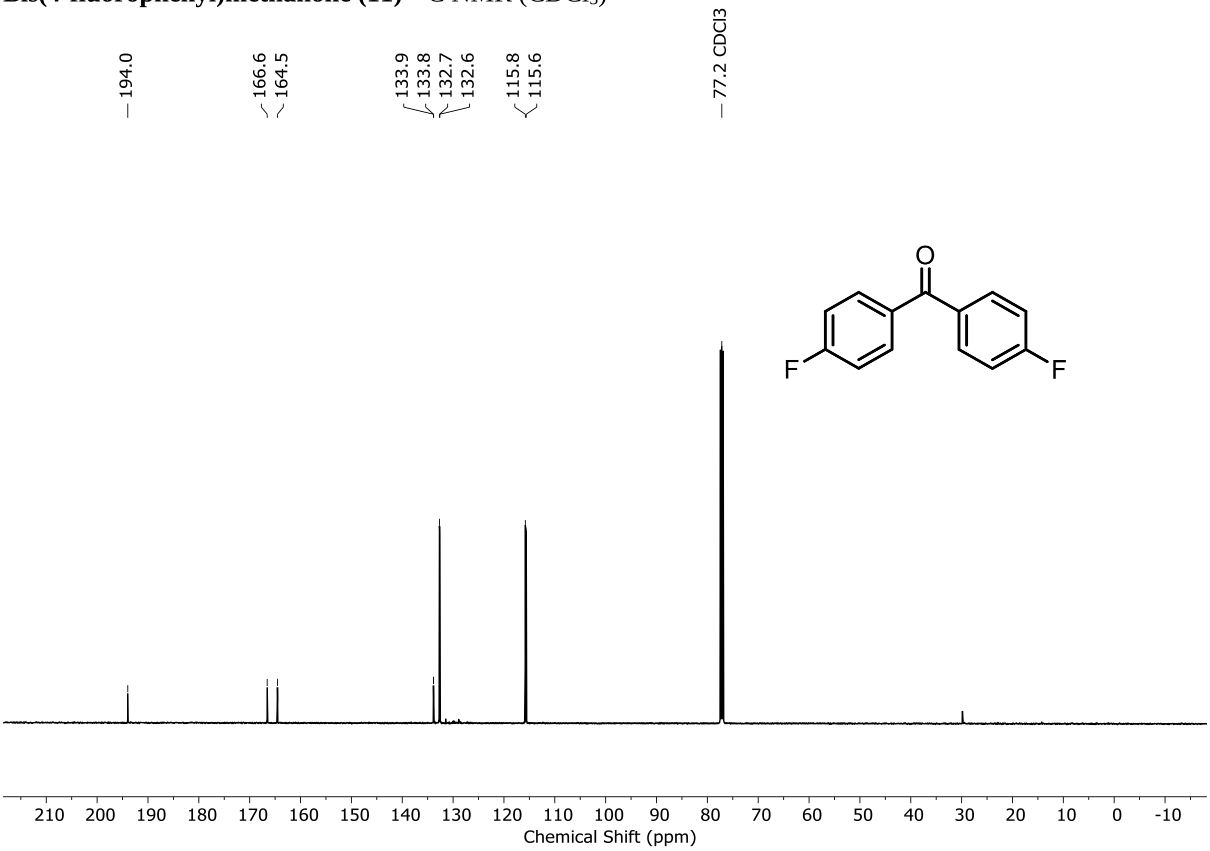

Bis(4-fluorophenyl)methanone (11) ^{19}F NMR ($CDCl_3$)

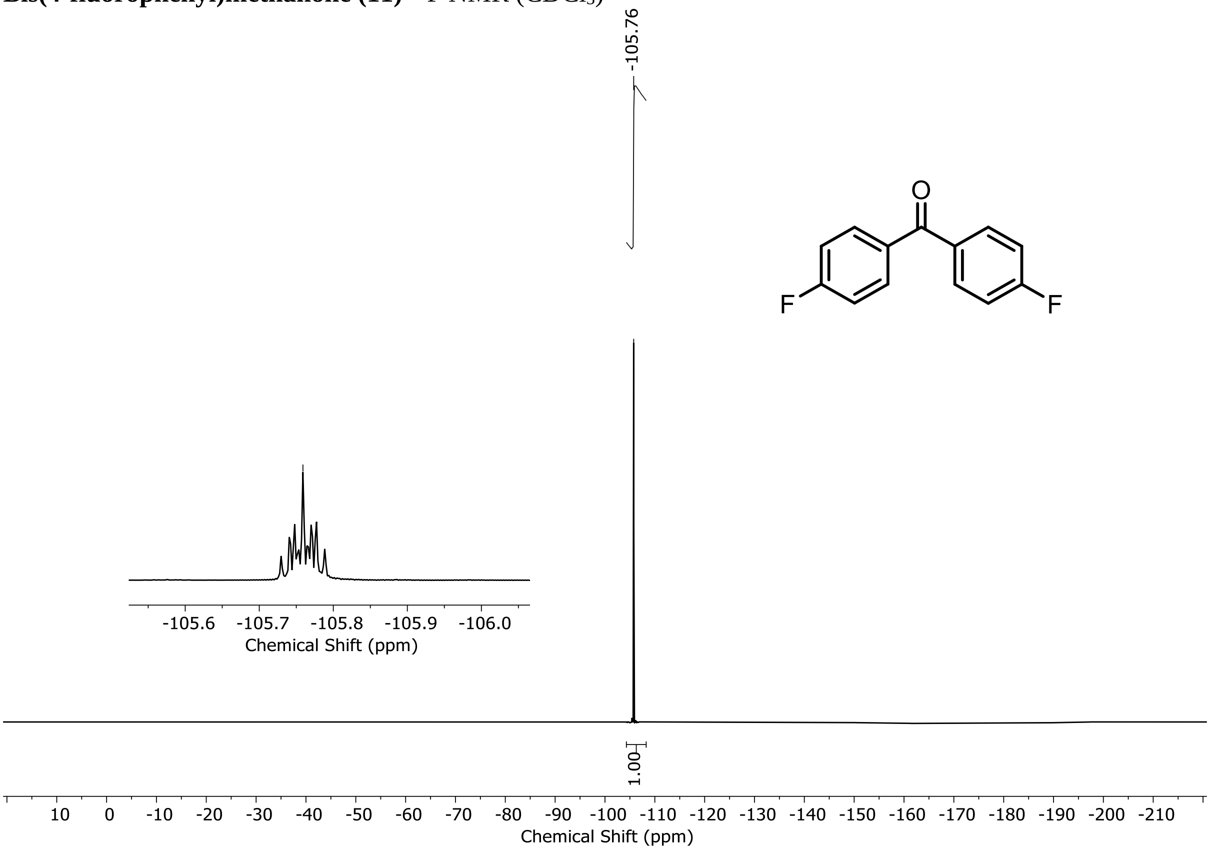

4-Fluorobenzaldehyde (12) ^{1}H NMR ($CDCl_3$)

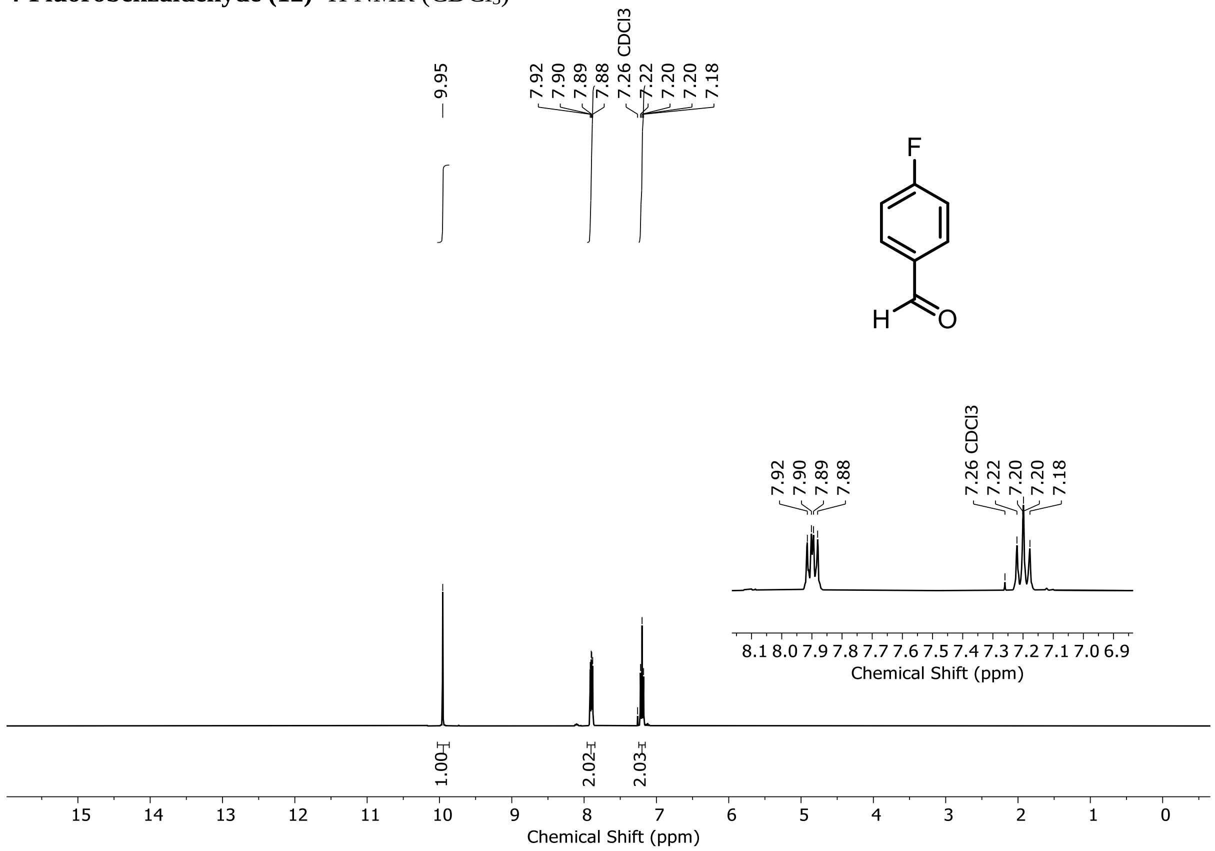

4-Fluorobenzaldehyde (12) ^{13}C NMR ($CDCl_3$)

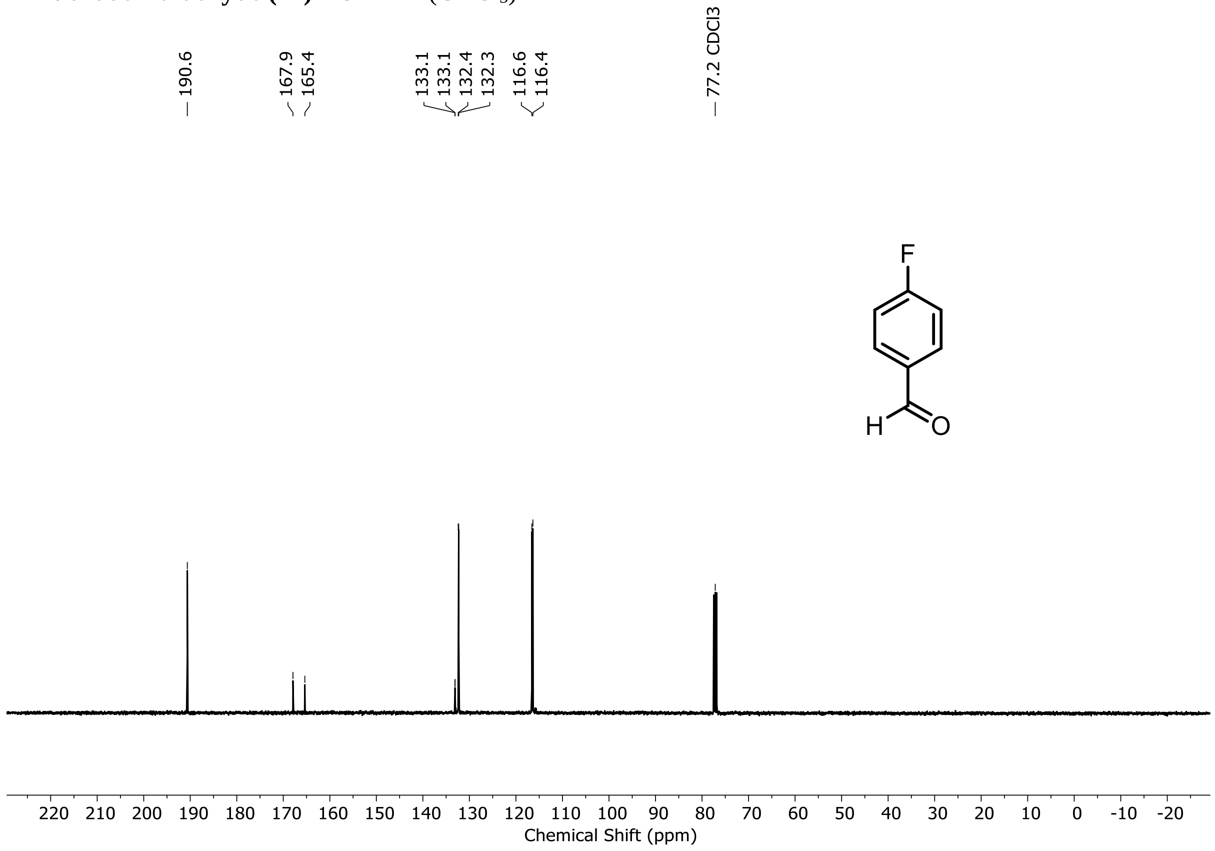

4-Fluorobenzaldehyde (12) ^{19}F NMR ($CDCl_3$)

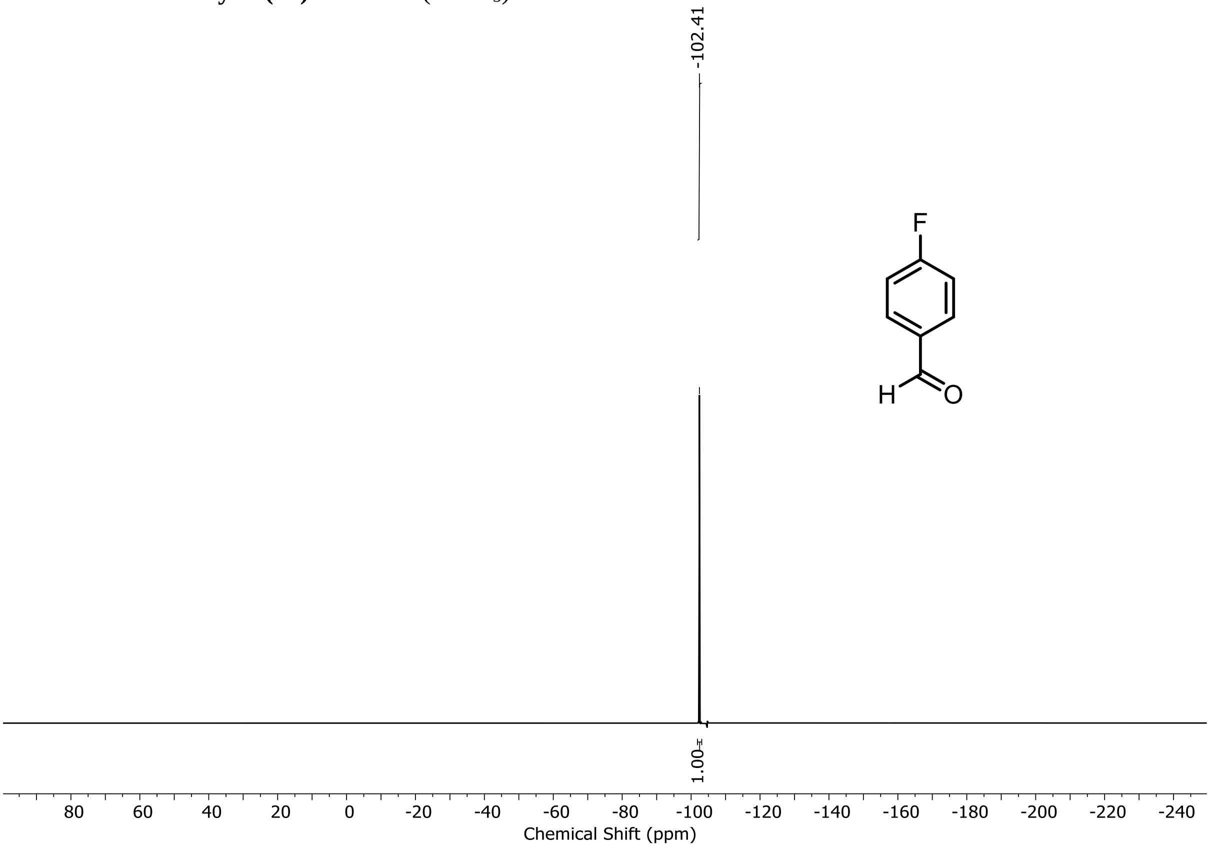

2-chloro-5-fluoronicotinonitrile (13) ^{1}H NMR ($CDCl_3$)

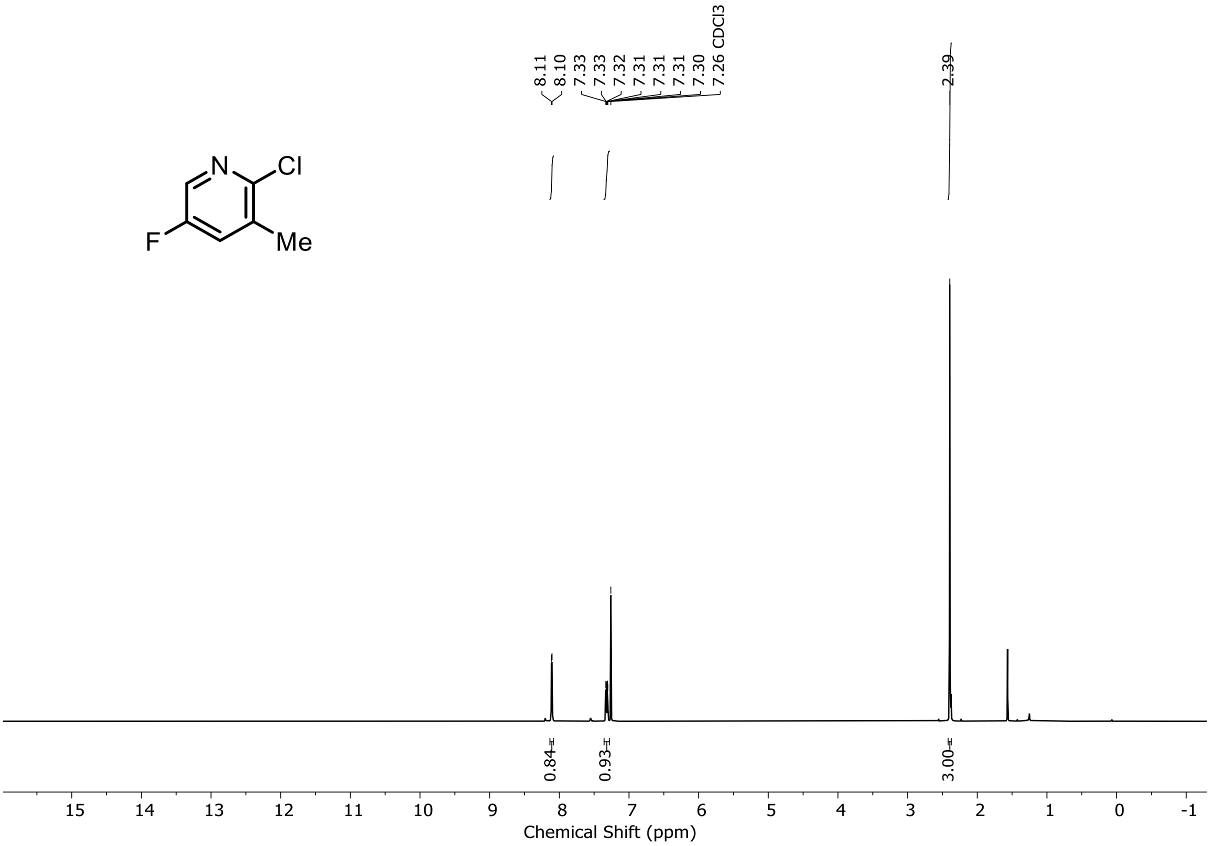

2-chloro-5-fluoronicotinonitrile (13) ^{13}C NMR ($CDCl_3$)

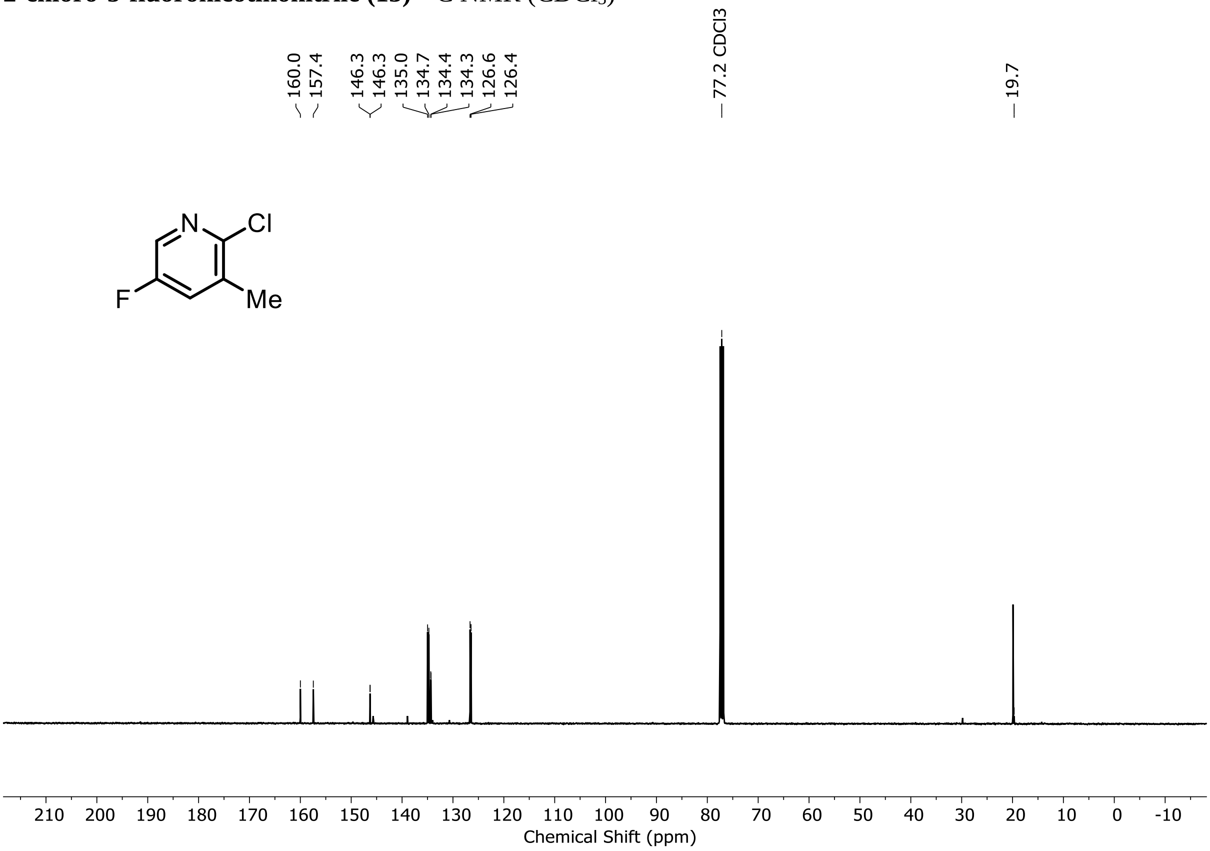

2-chloro-5-fluoronicotinonitrile (13) ^{19}F NMR ($CDCl_3$)

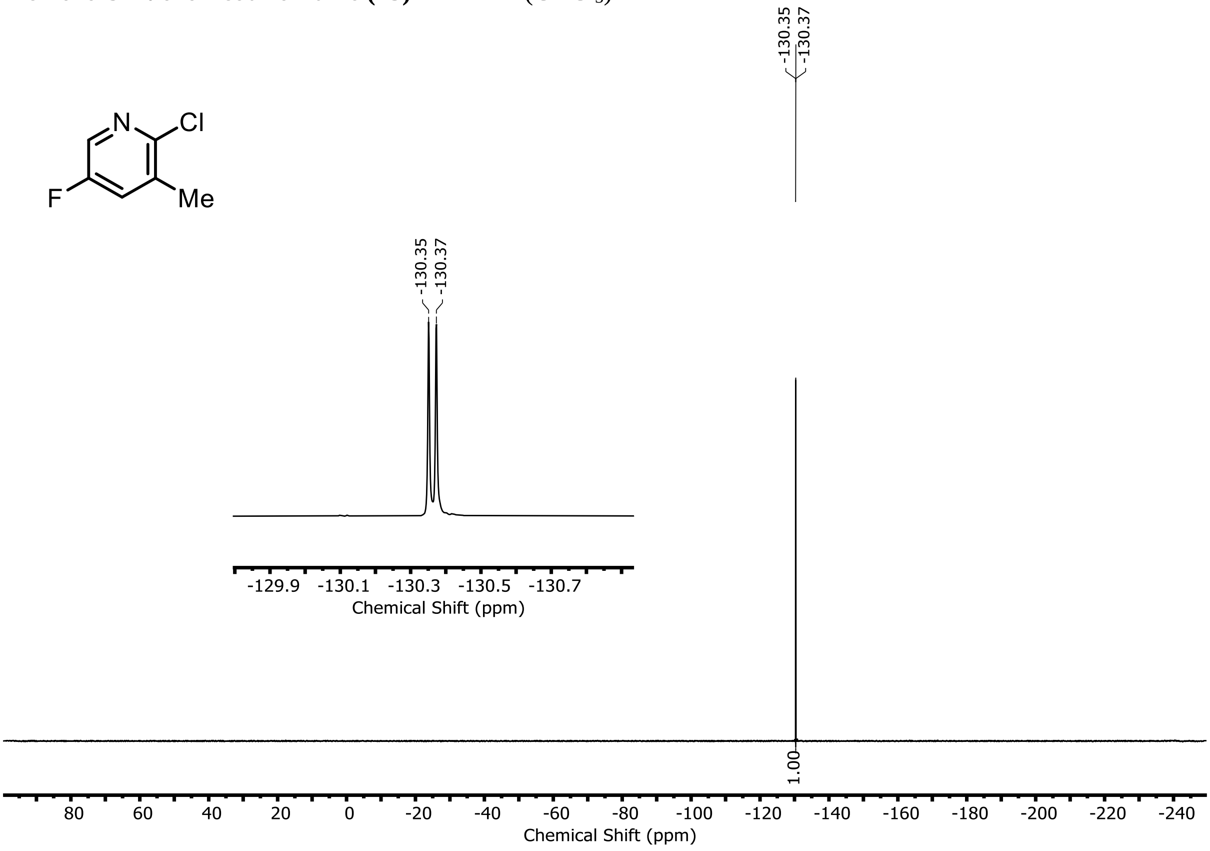

1-Fluoro-2,4-dinitrobenzene (14) ^{1}H NMR ($CDCl_3$)

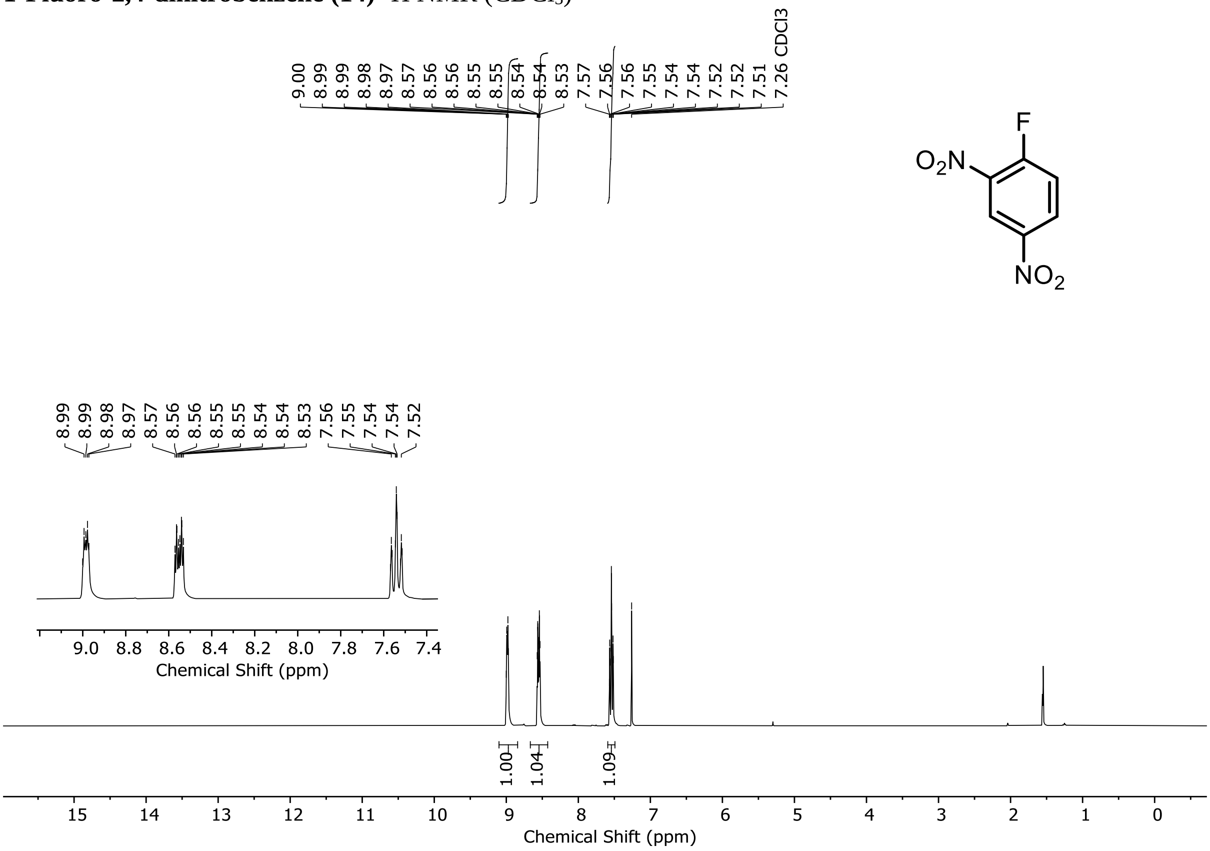

1-Fluoro-2,4-dinitrobenzene (14) ^{13}C NMR ($CDCl_3$)

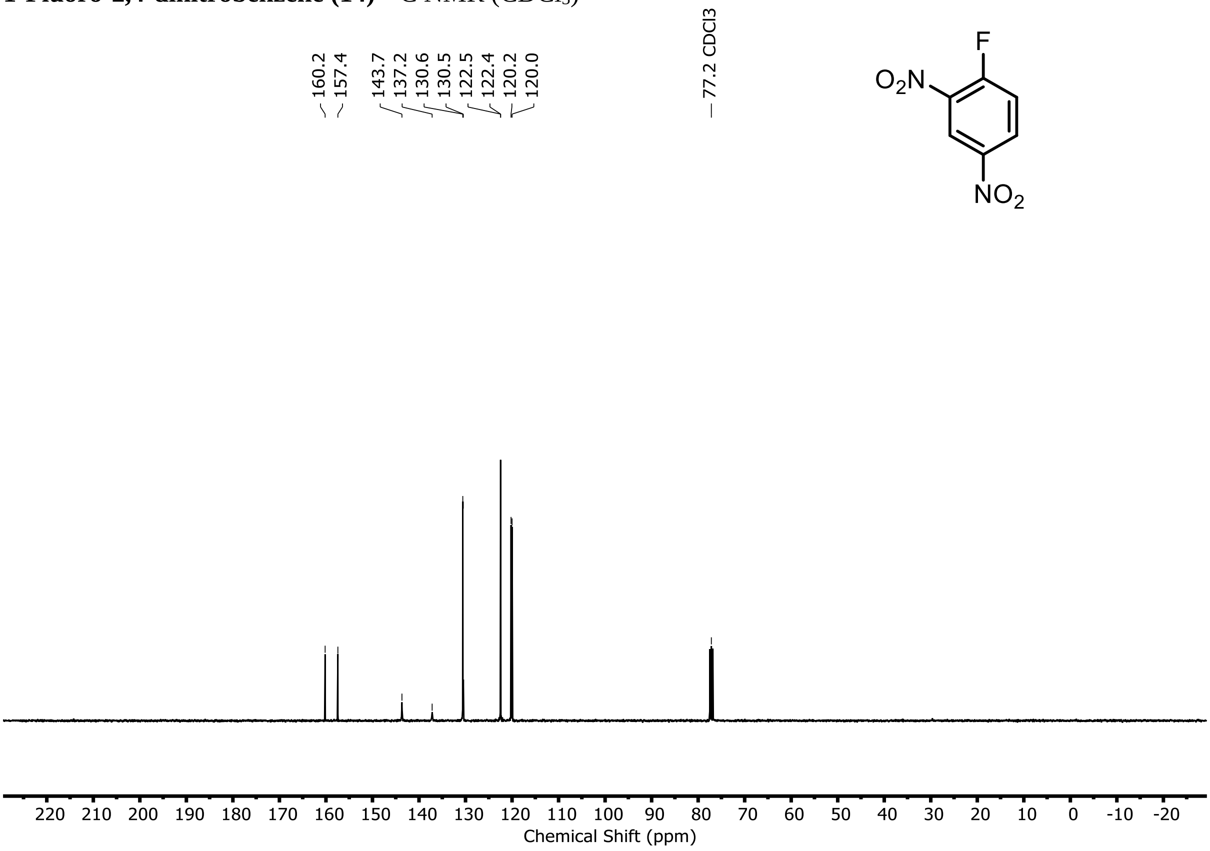

1-Fluoro-2,4-dinitrobenzene (14) ^{19}F NMR ($CDCl_3$)

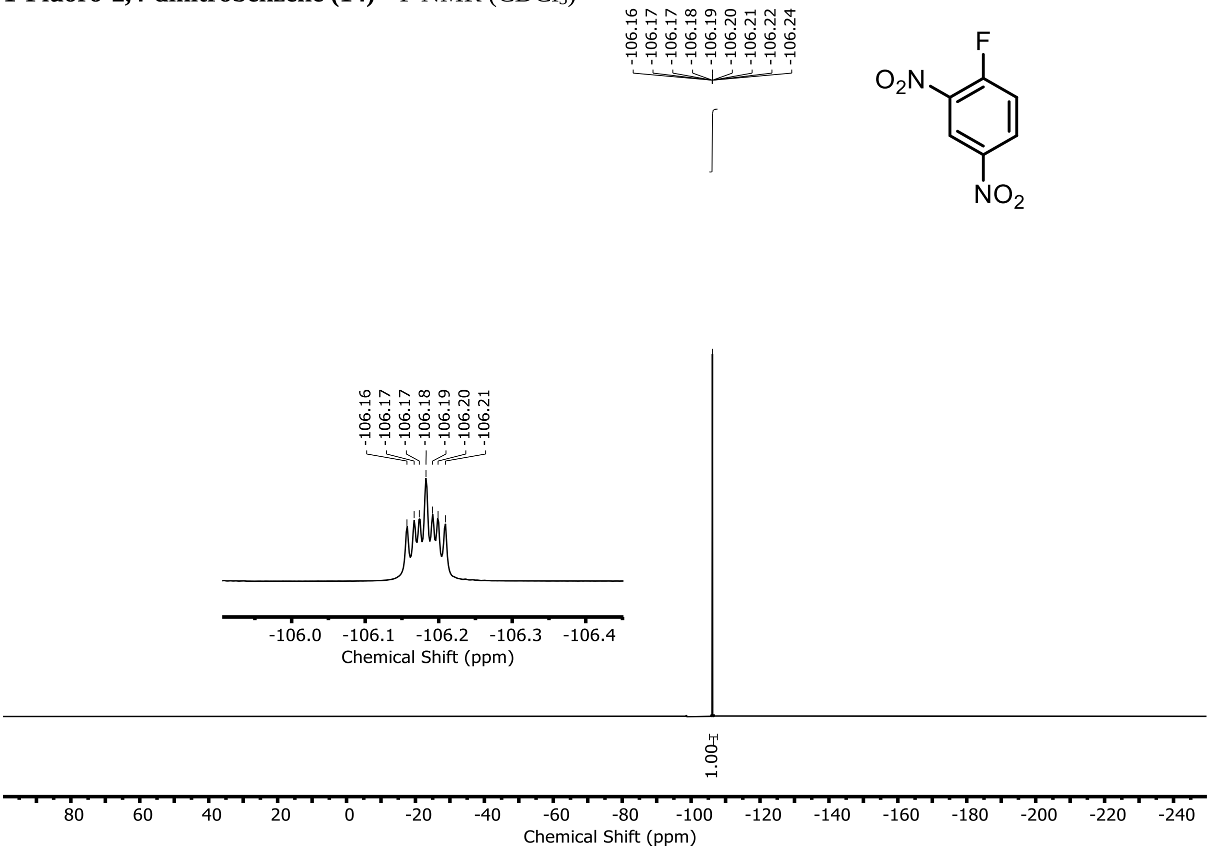

2-chloro-1-fluoro-4-nitrobenzene (15) ^{1}H NMR ($CDCl_3$)

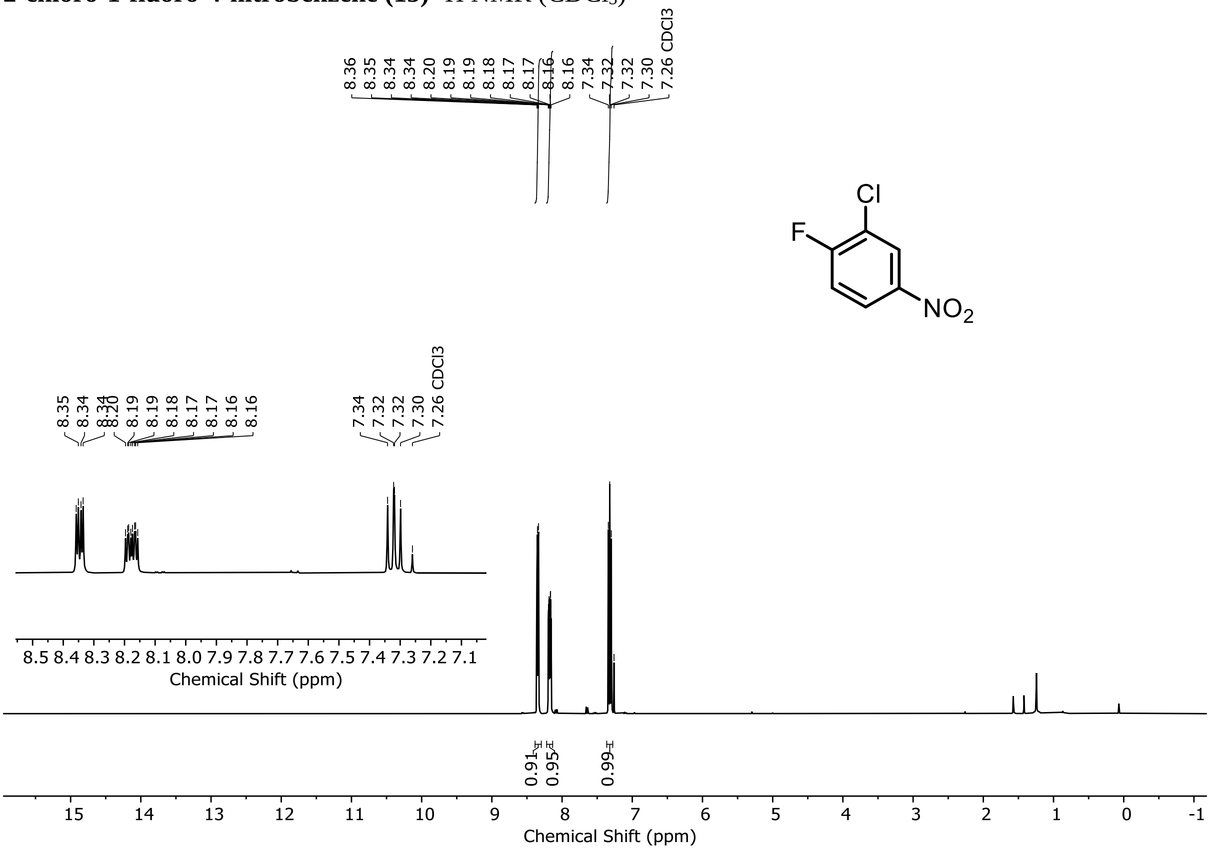

2-chloro-1-fluoro-4-nitrobenzene (15) ^{13}C NMR ($CDCl_3$)

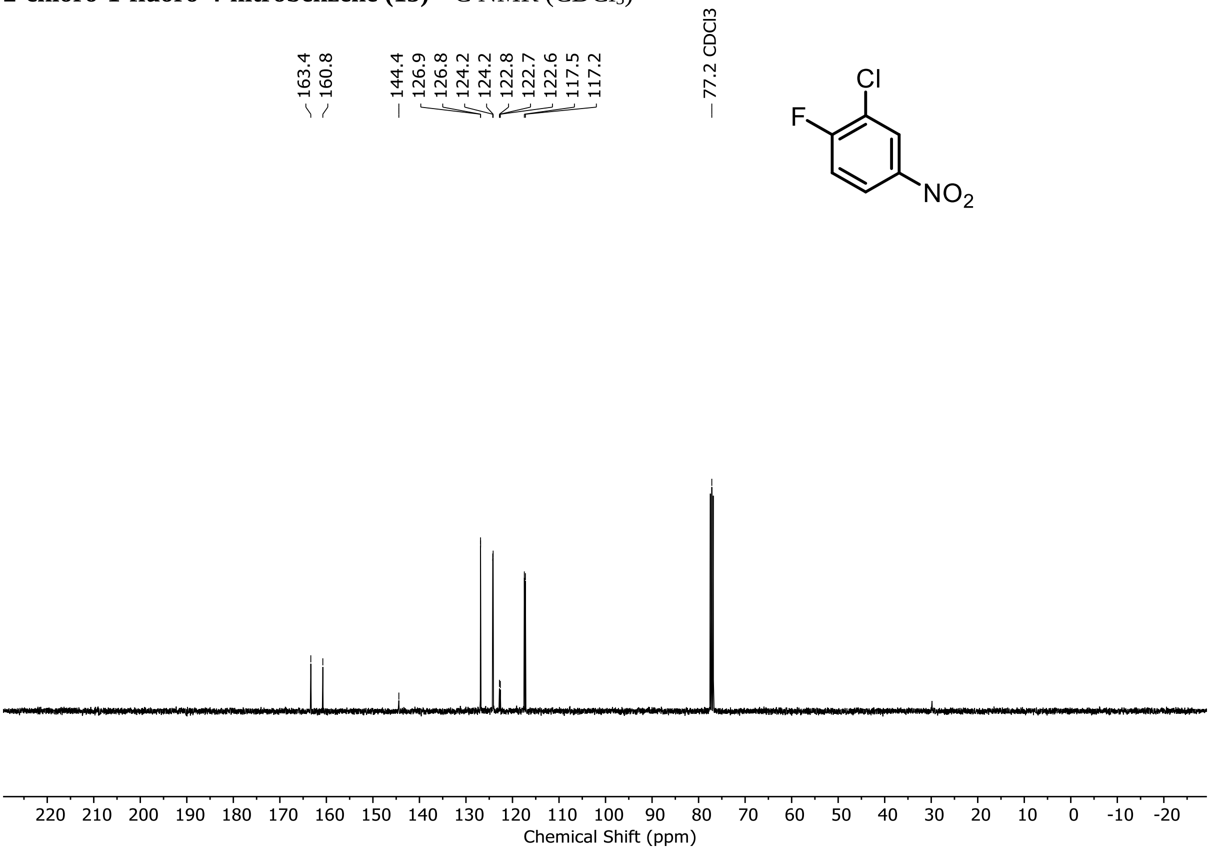

2-chloro-1-fluoro-4-nitrobenzene (15) ^{19}F NMR ($CDCl_3$)

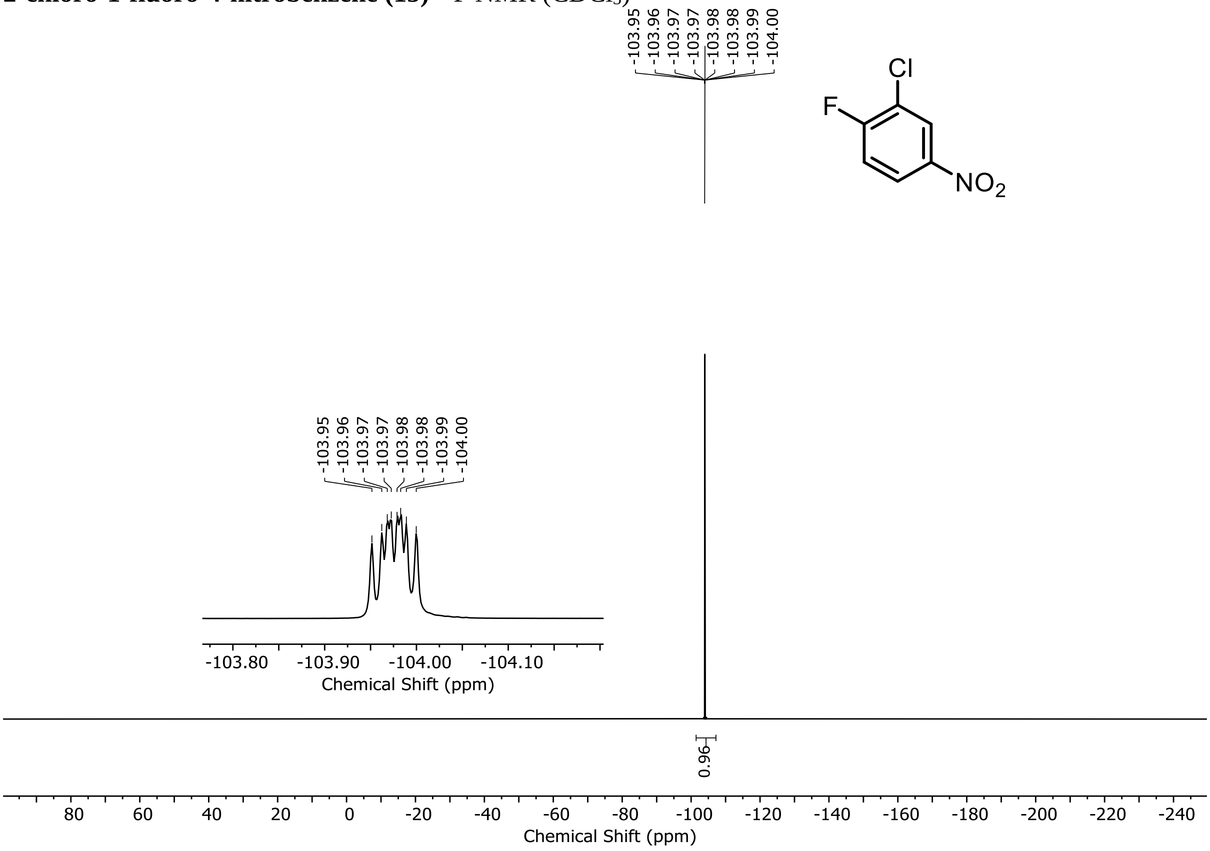

2-fluoro-5-nitrobenzonitrile (16) ^{1}H NMR ($CDCl_3$)

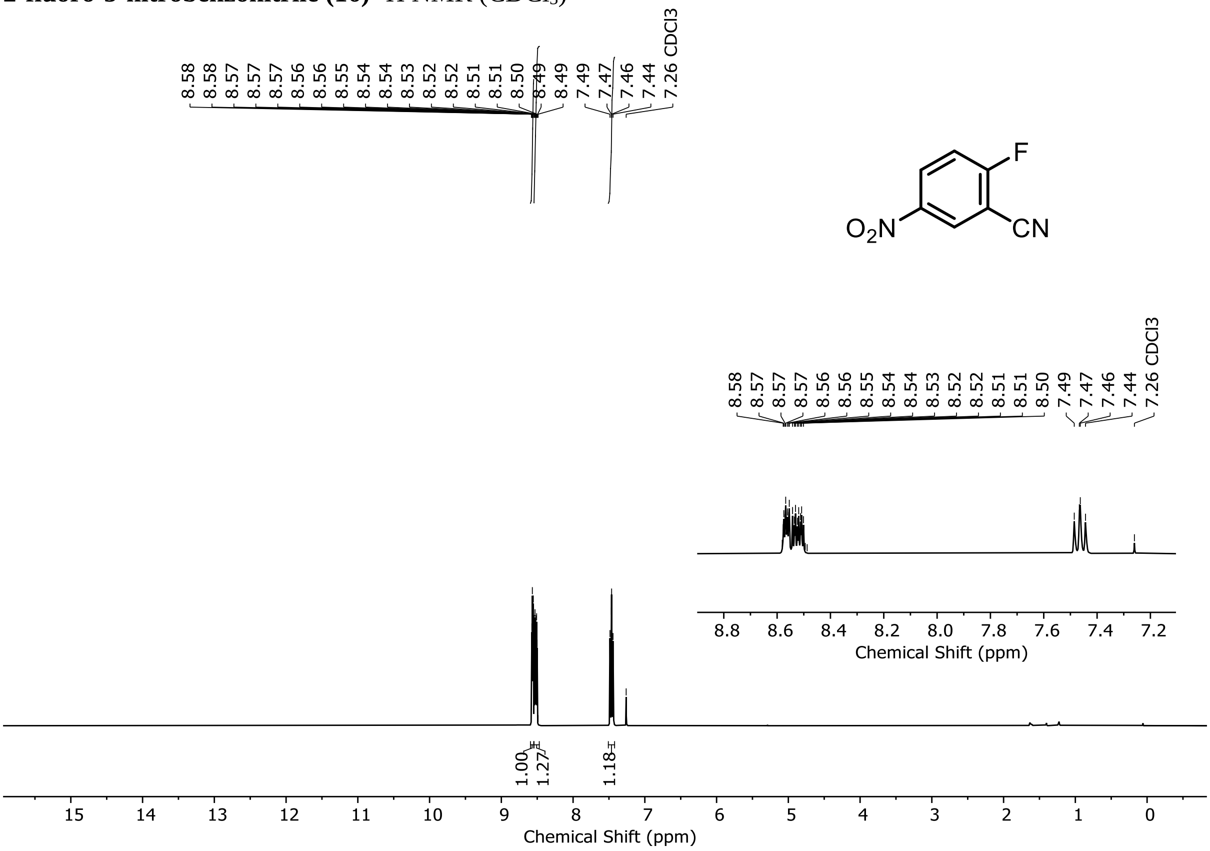

2-fluoro-5-nitrobenzonitrile (16) ^{13}C NMR ($CDCl_3$)

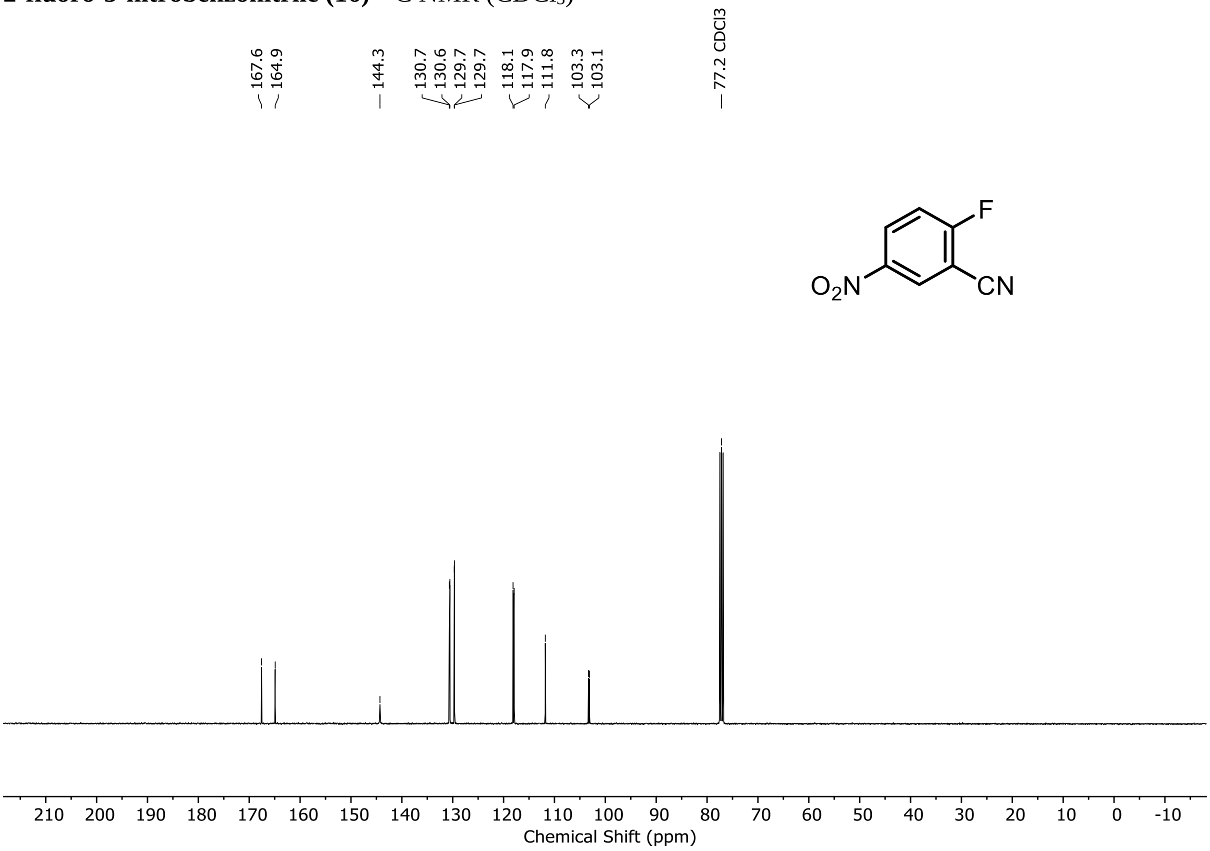

2-fluoro-5-nitrobenzonitrile (16) ^{19}F NMR ($CDCl_3$)

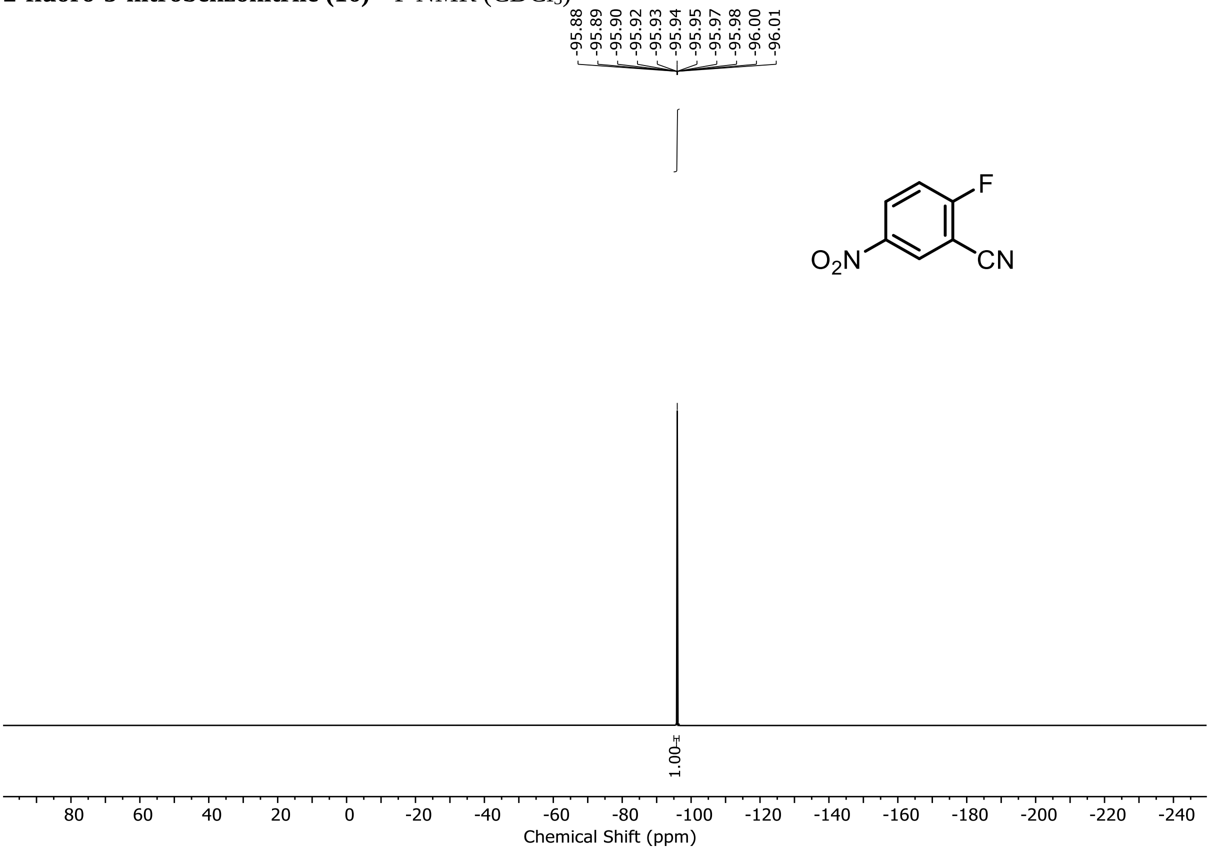

3-chloro-4-fluorobenzonitrile (17) ^{1}H NMR ($CDCl_3$)

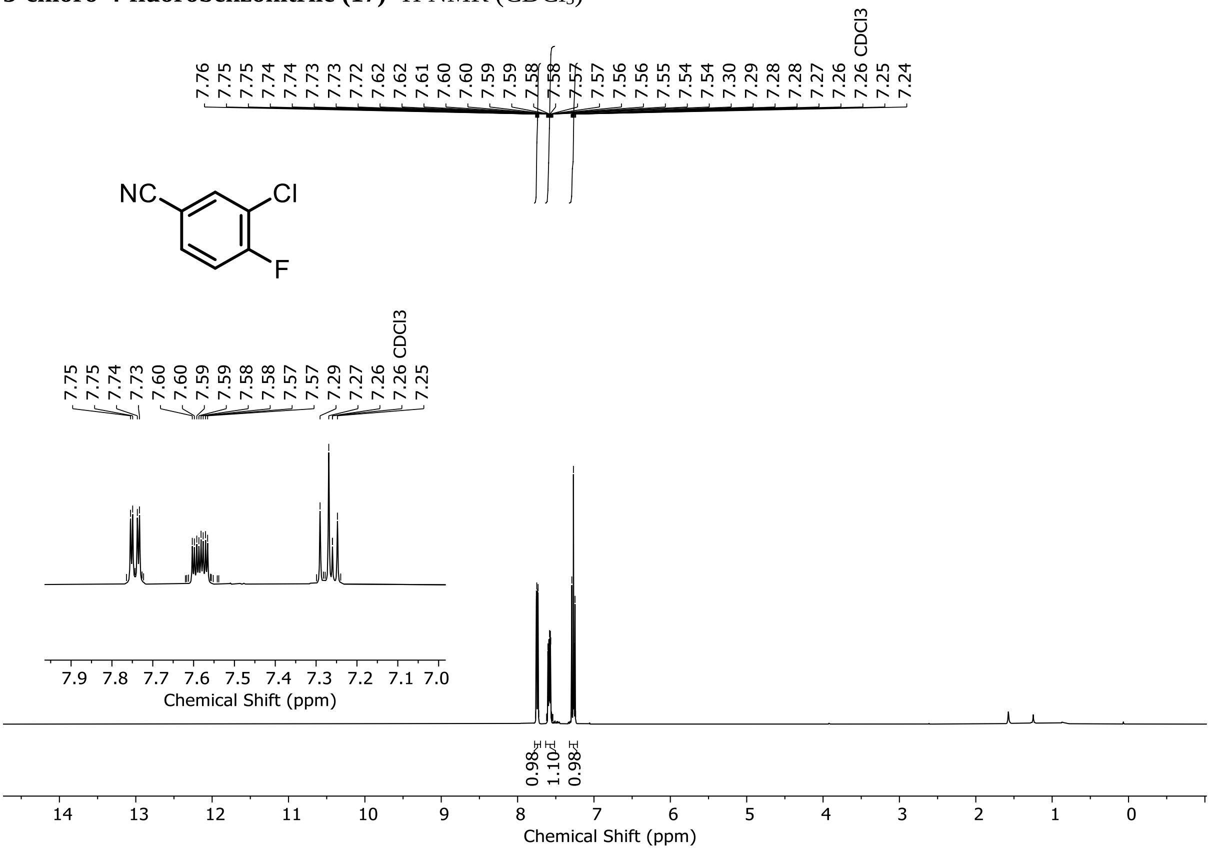

3-chloro-4-fluorobenzonitrile (17) ^{13}C NMR ($CDCl_3$)

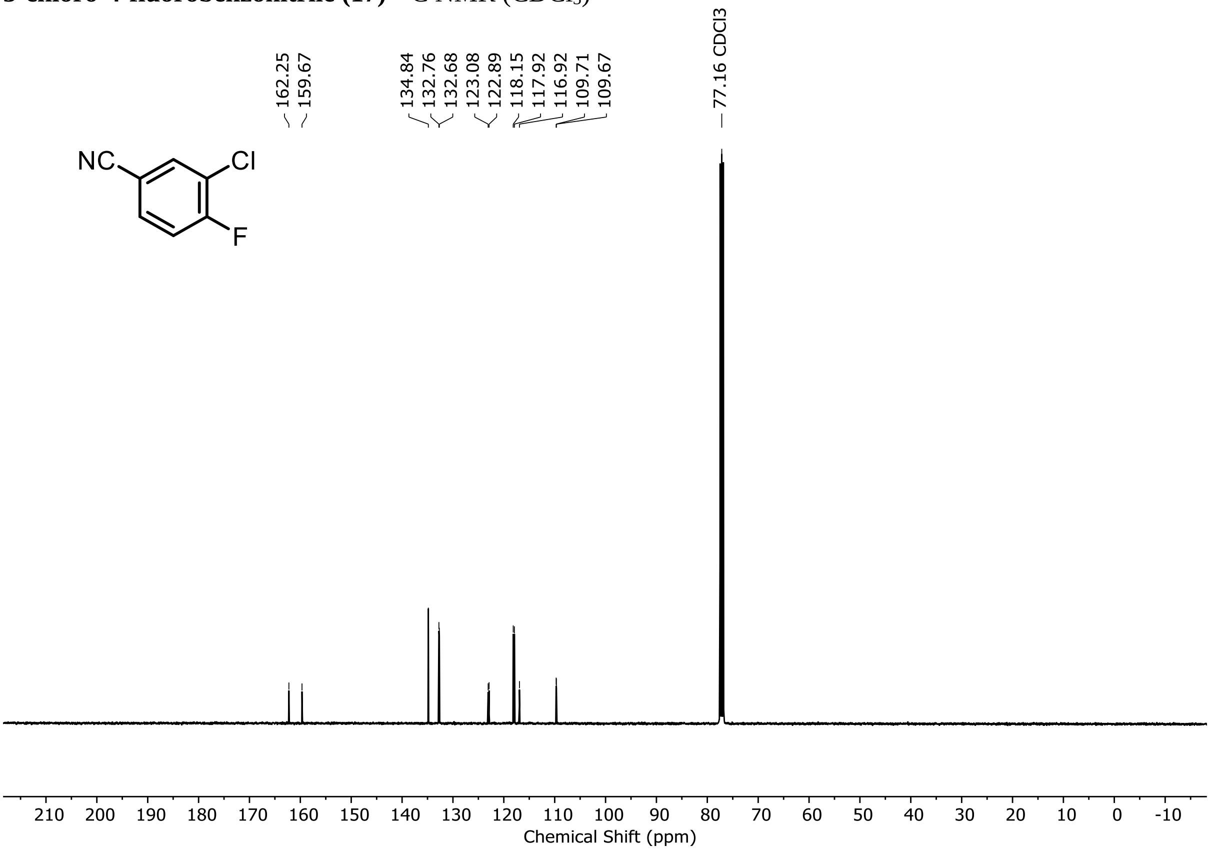

3-chloro-4-fluorobenzonitrile (17) ^{19}F NMR ($CDCl_3$)

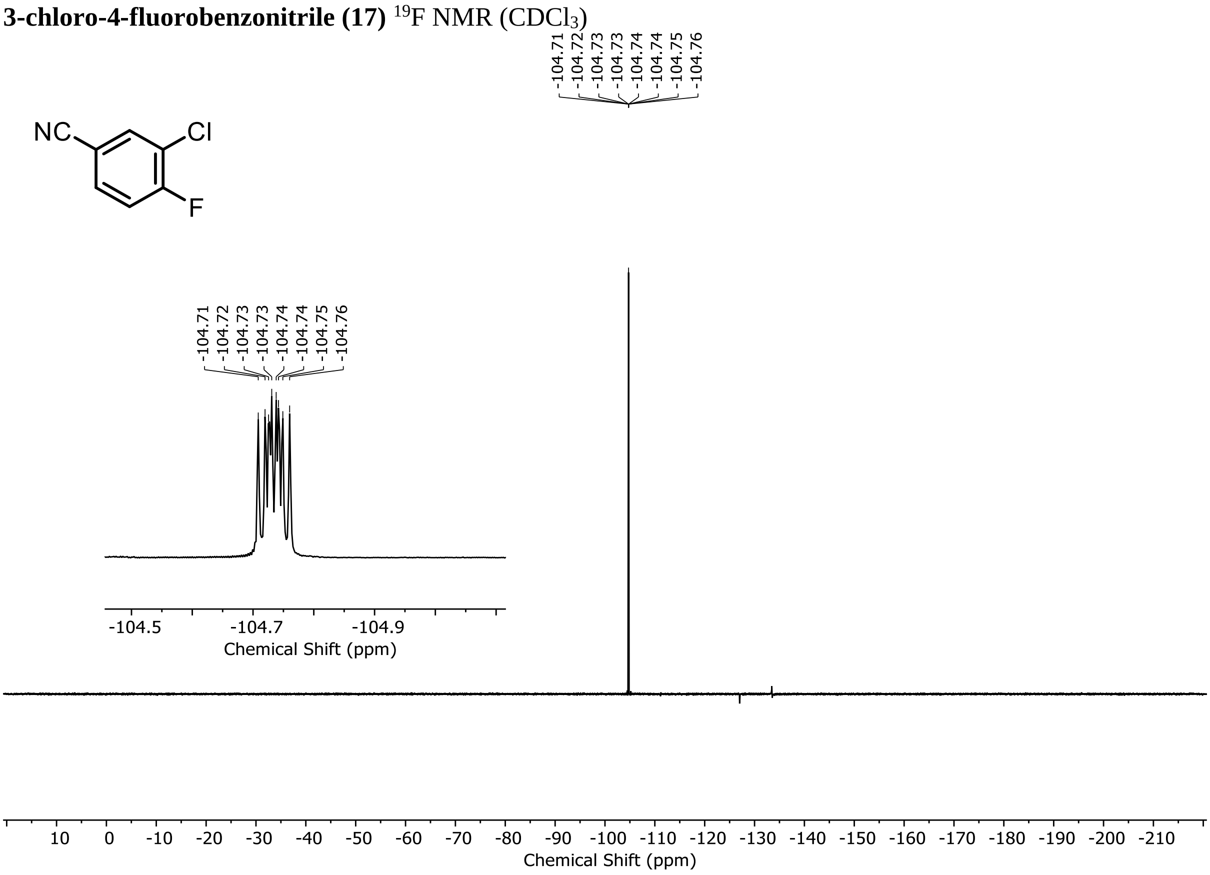

2,6-difluorobenzonitrile (18) ^{1}H NMR ($CDCl_3$)

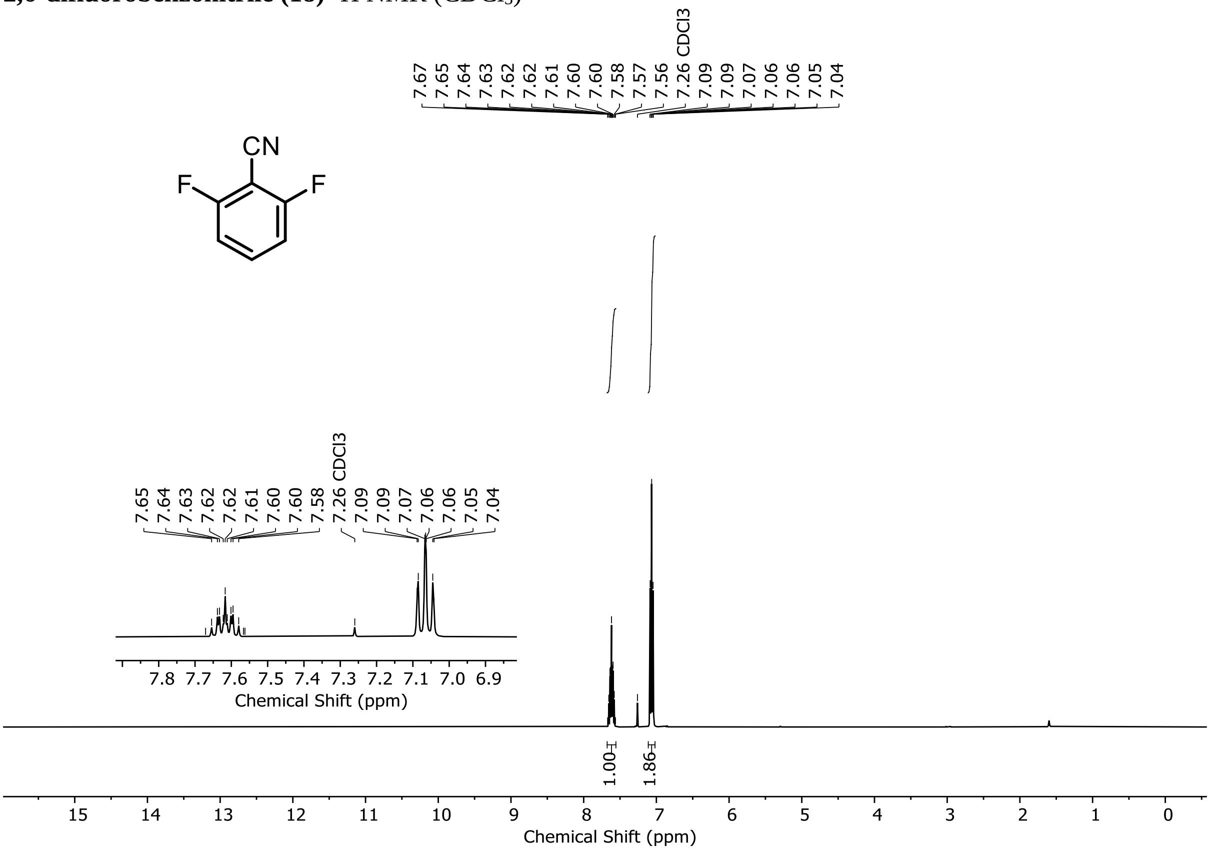

2,6-difluorobenzonitrile (18) ^{13}C NMR ($CDCl_3$)

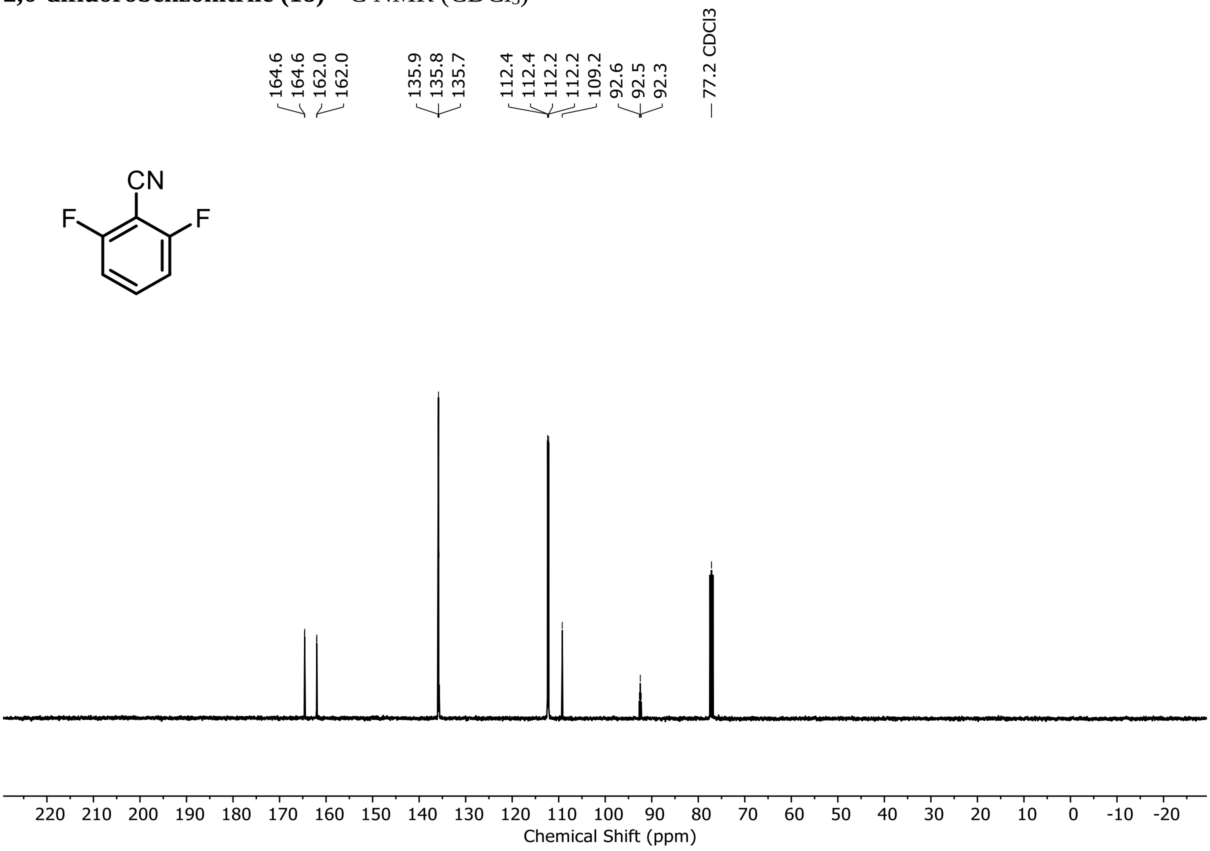

2,6-difluorobenzonitrile (18) ^{19}F NMR ($CDCl_3$)

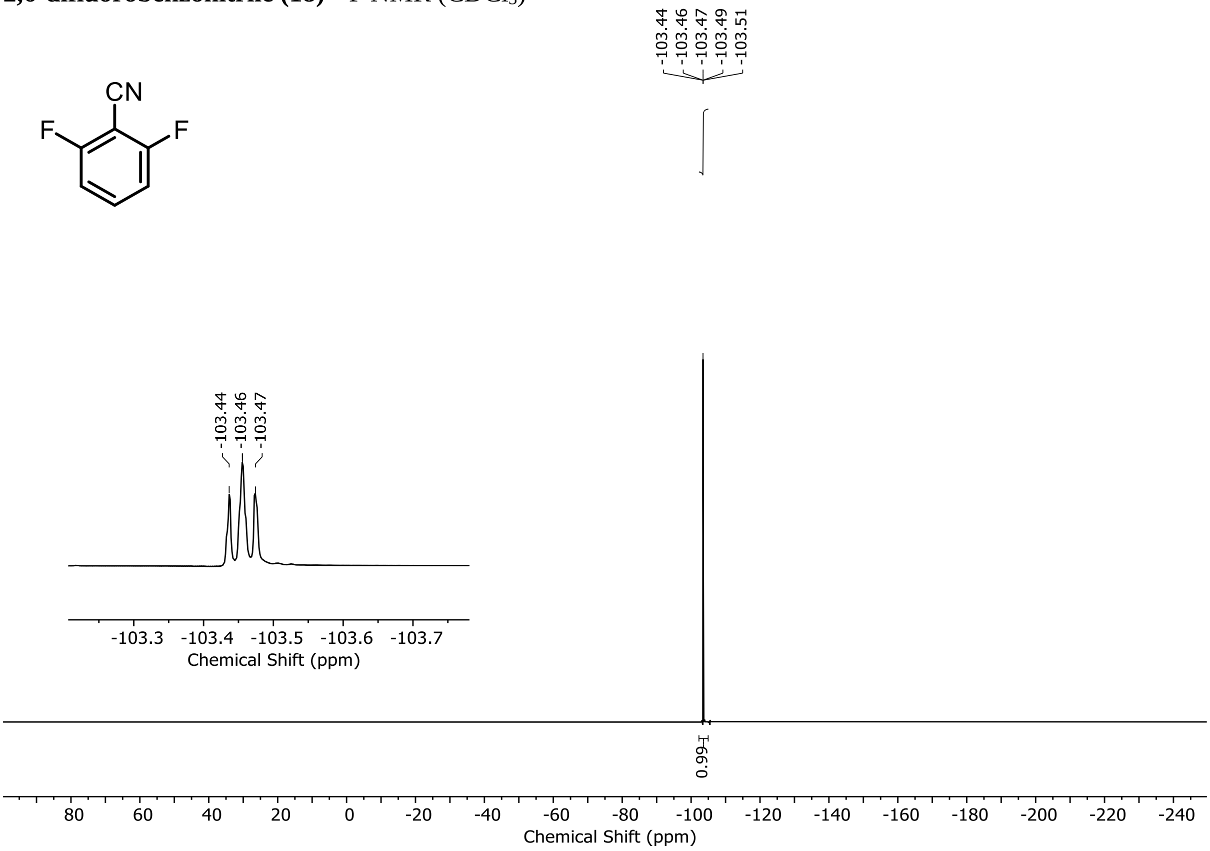

4-fluoronitrobenzene (19) ^{1}H NMR ($CDCl_3$)

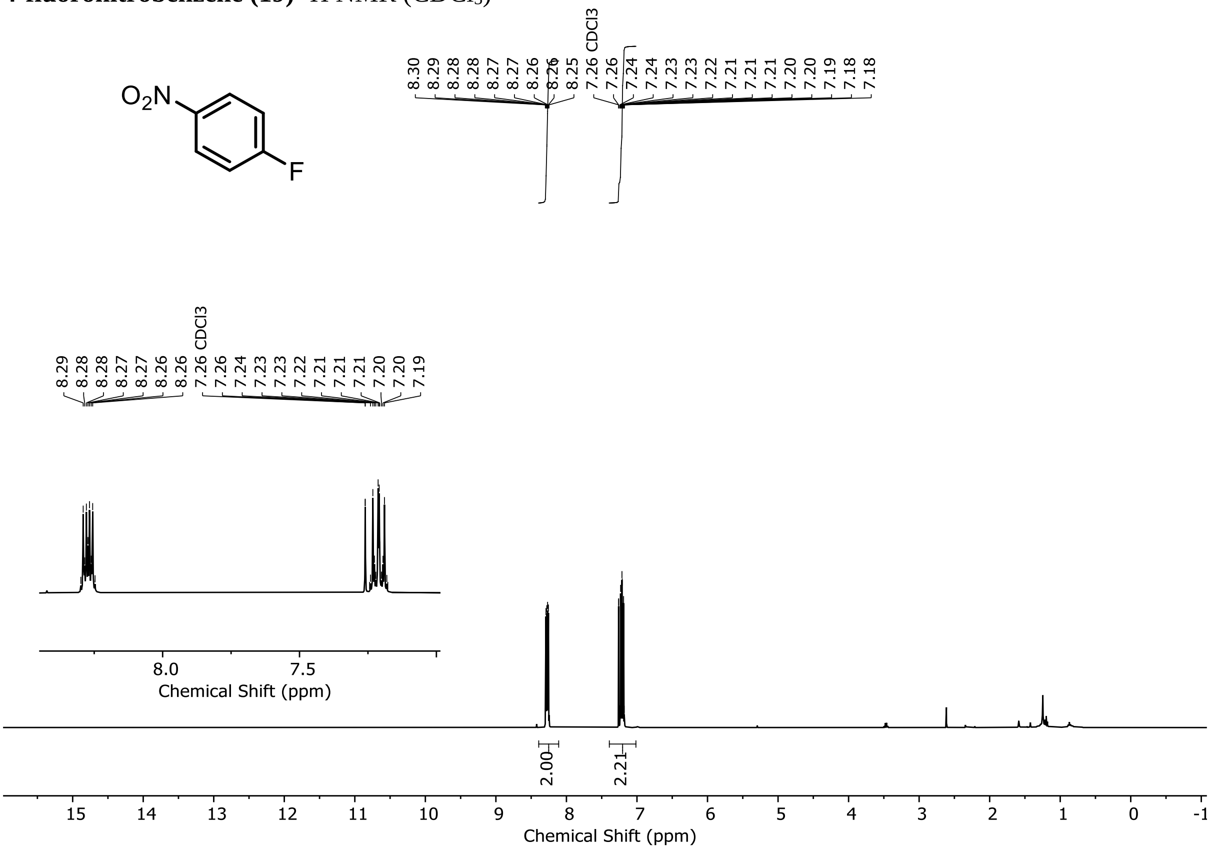

4-fluoronitrobenzene (19) ^{13}C NMR ($CDCl_3$)

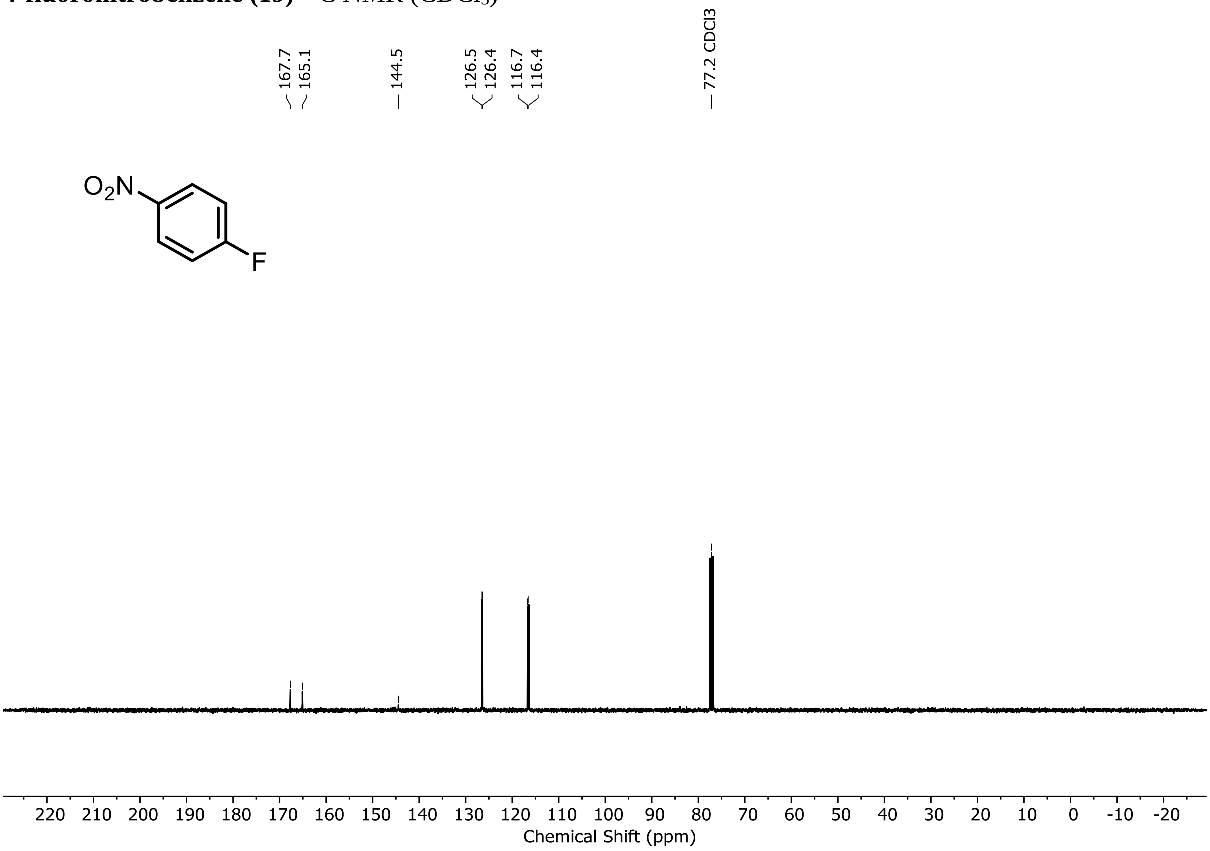

4-fluoronitrobenzene (19) ^{19}F NMR ($CDCl_3$)

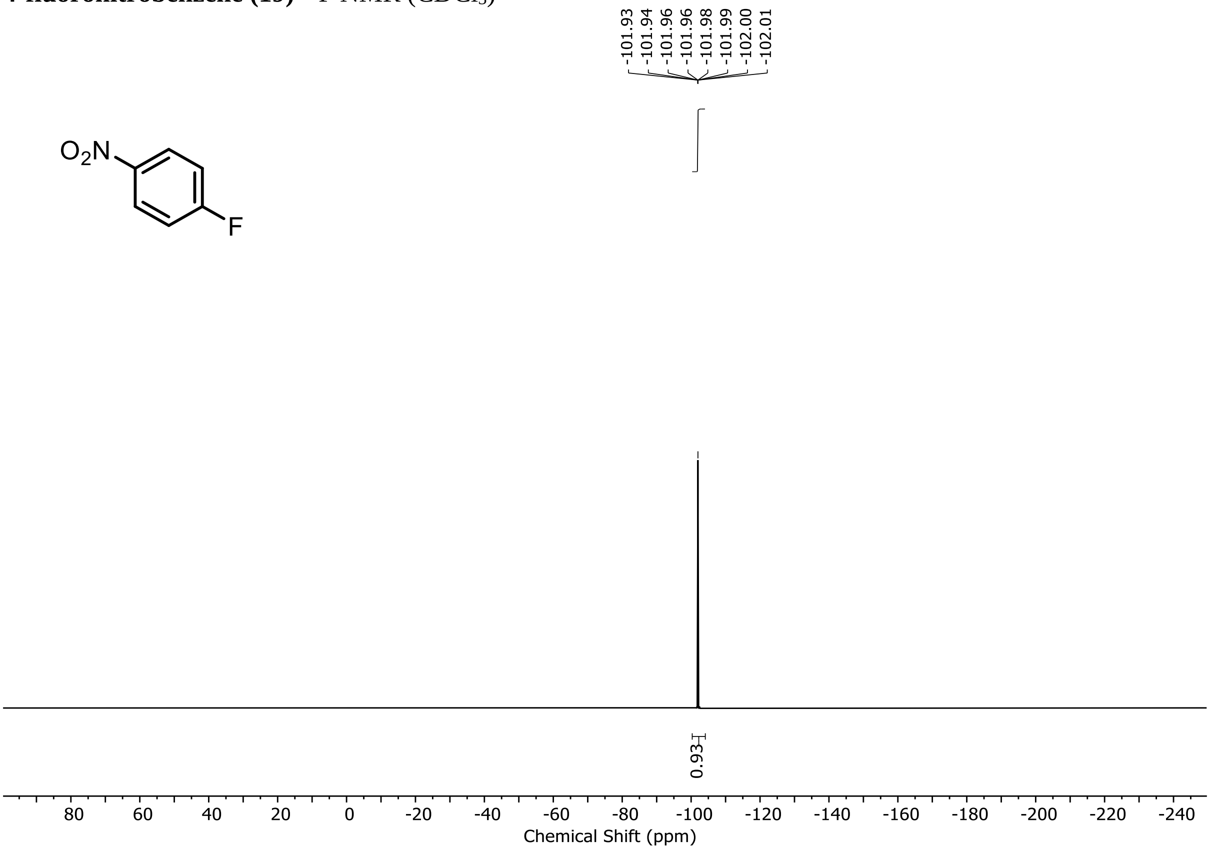

2-fluorobenzonitrile (20) ^{1}H NMR ($CDCl_3$)

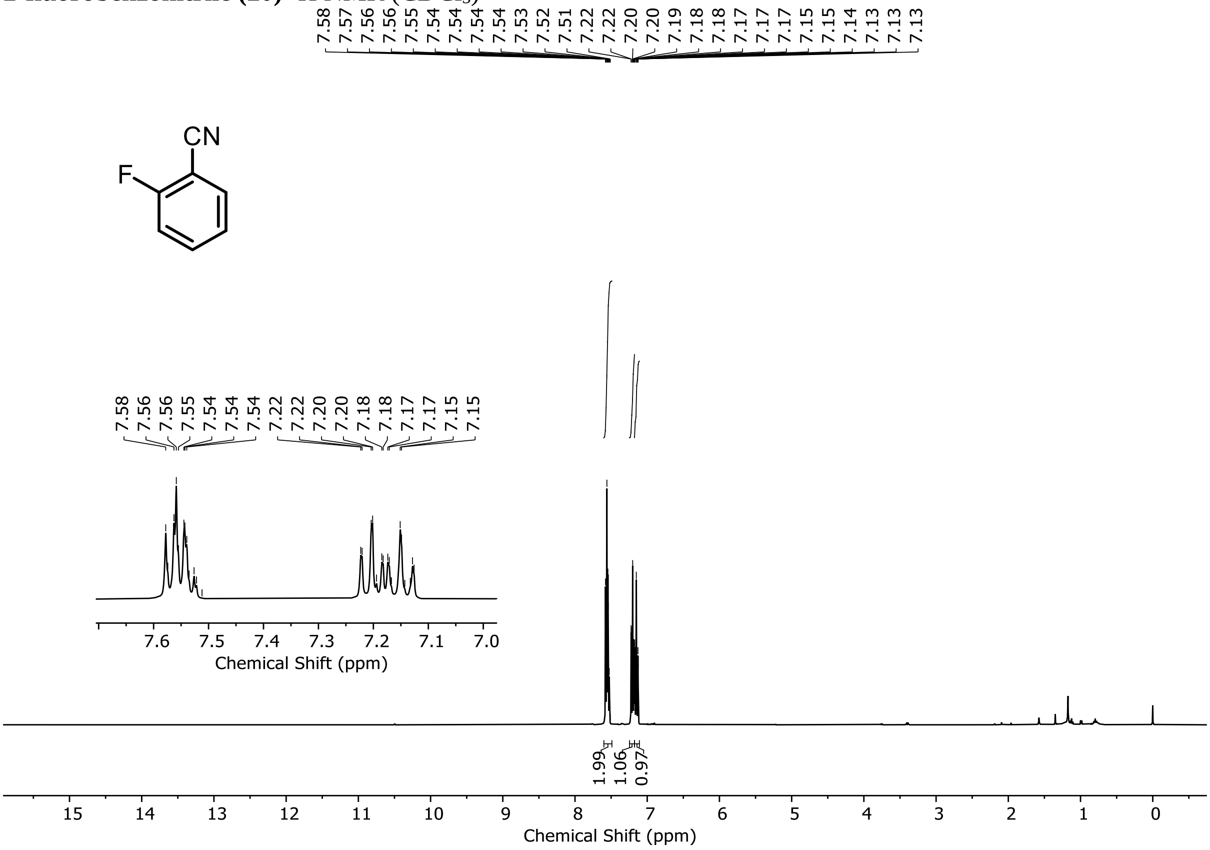

2-fluorobenzonitrile (20) ^{13}C NMR ($CDCl_3$)

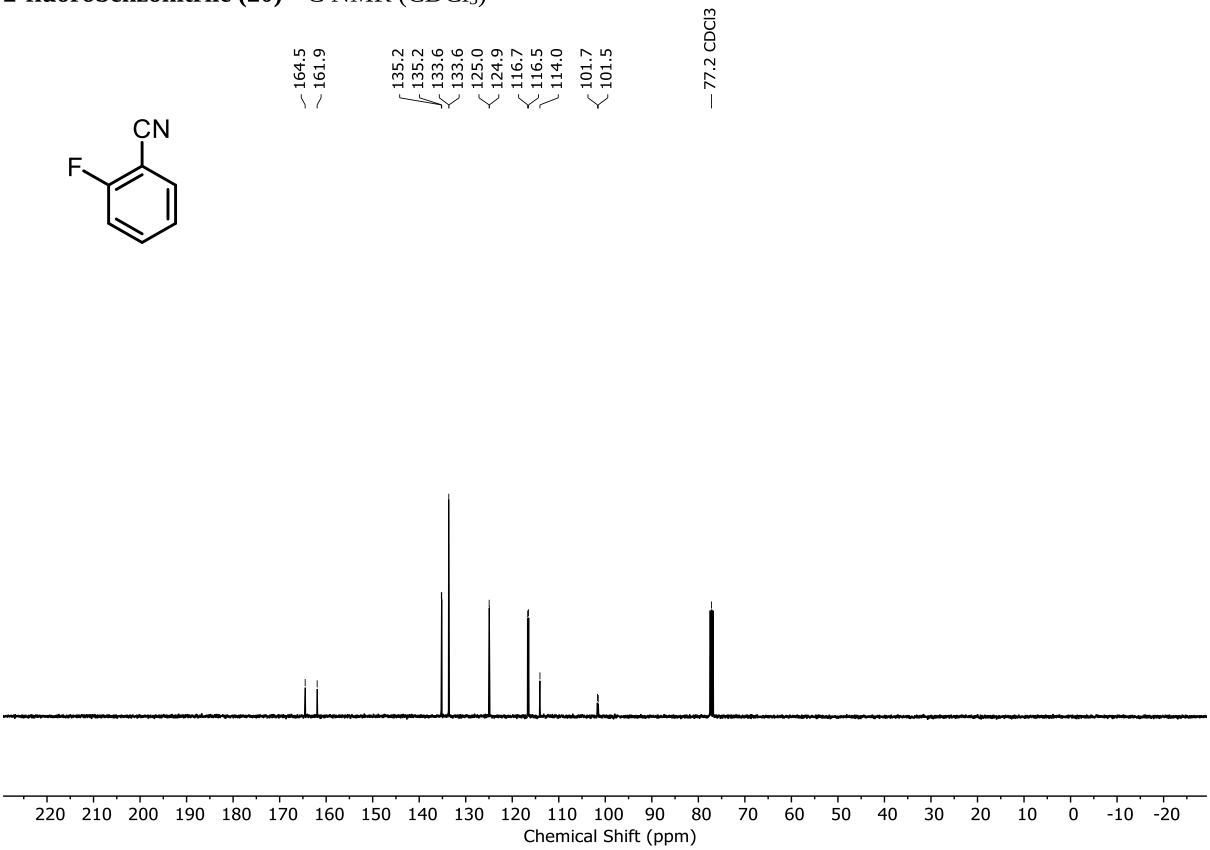

2-fluorobenzonitrile (20) ^{19}F NMR ($CDCl_3$)

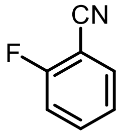

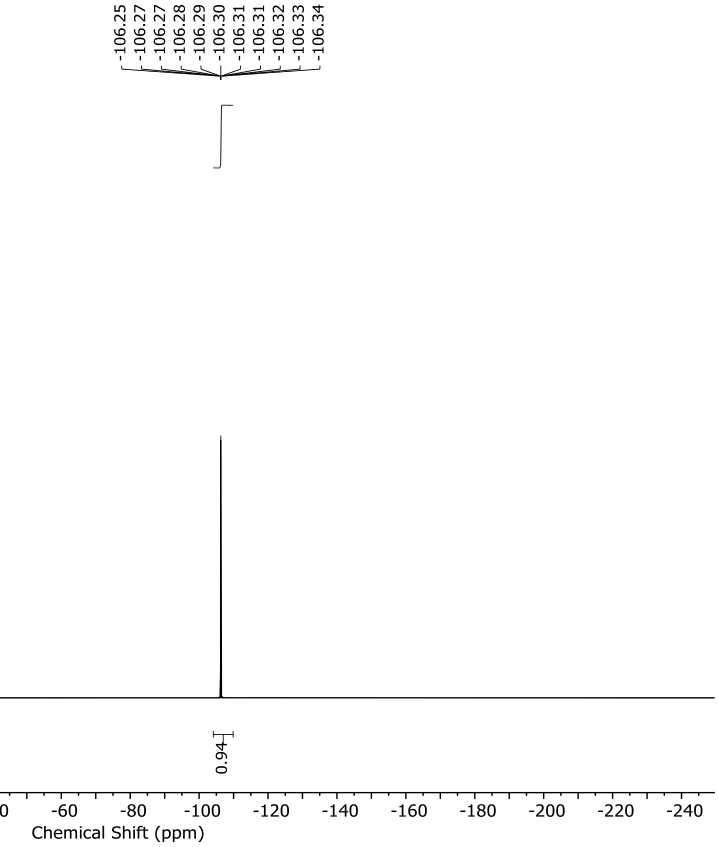

Acid grade fluorspar derived Me4NF·tAmOH ^{1}H NMR (D_2O)

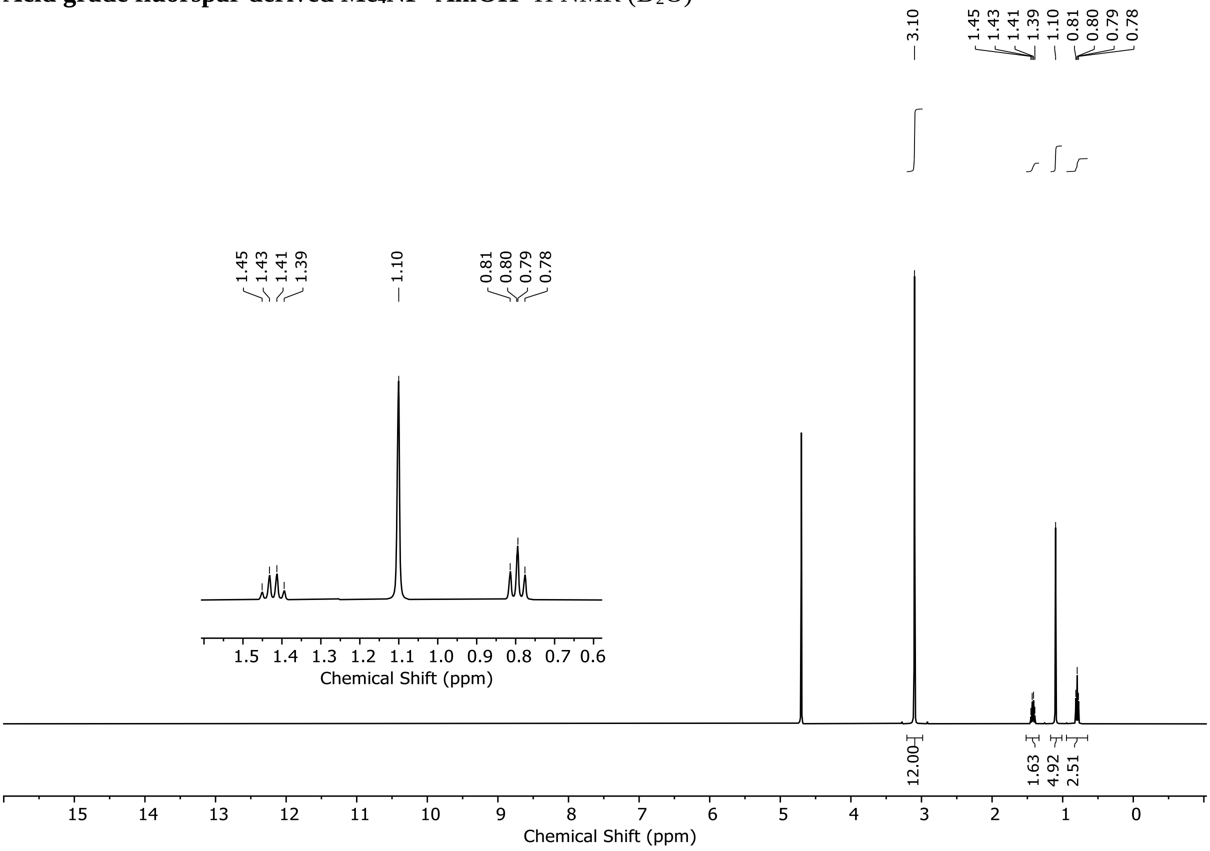

Acid grade fluorspar derived $Me_4NF \cdot {}^tAmOH$ ^{13}C NMR (D_2O)

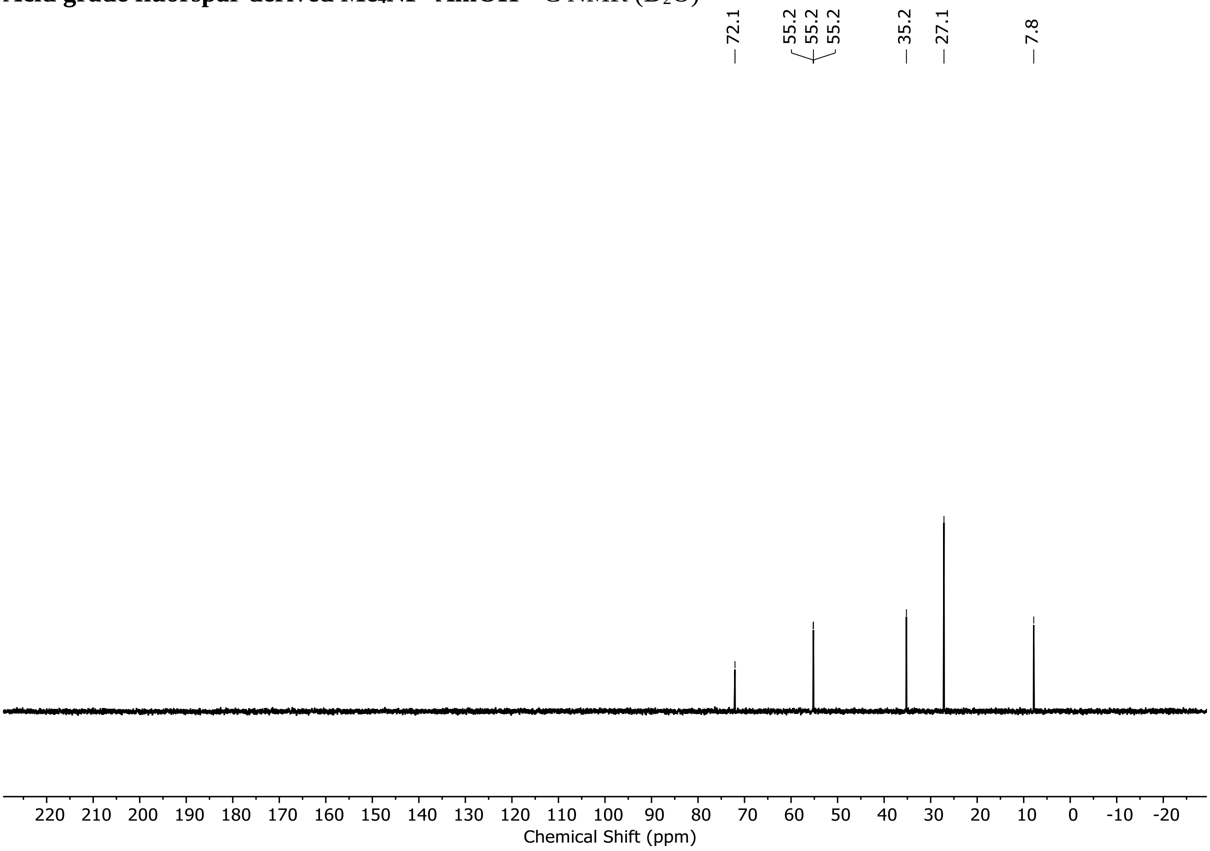

Acid grade fluorspar derived $Me_4NF \cdot {}^tAmOH$ ^{19}F NMR (D_2O)

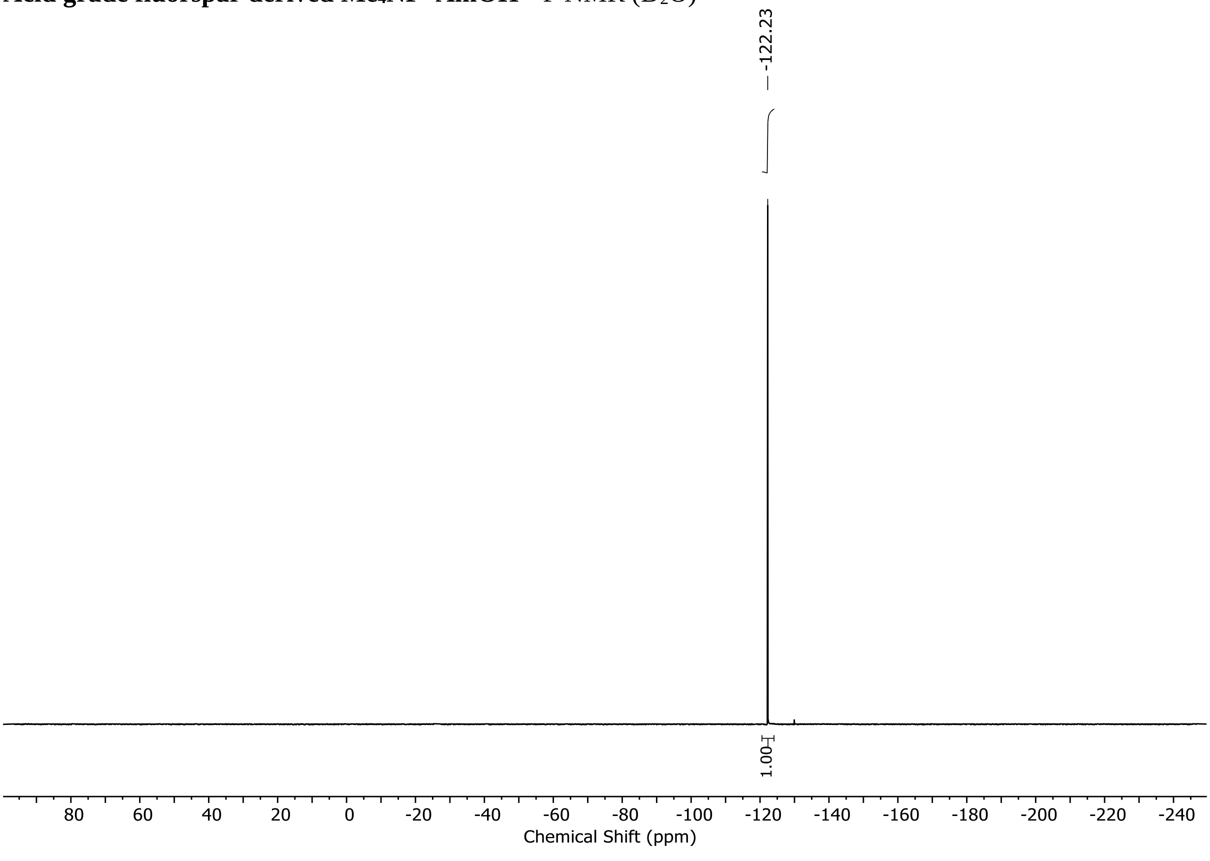

Acid grade fluorspar derived ^{n}Bu$_4$NF·(^{t}BuOH)$_4$ ^{1}H NMR (CDCl$_3$)

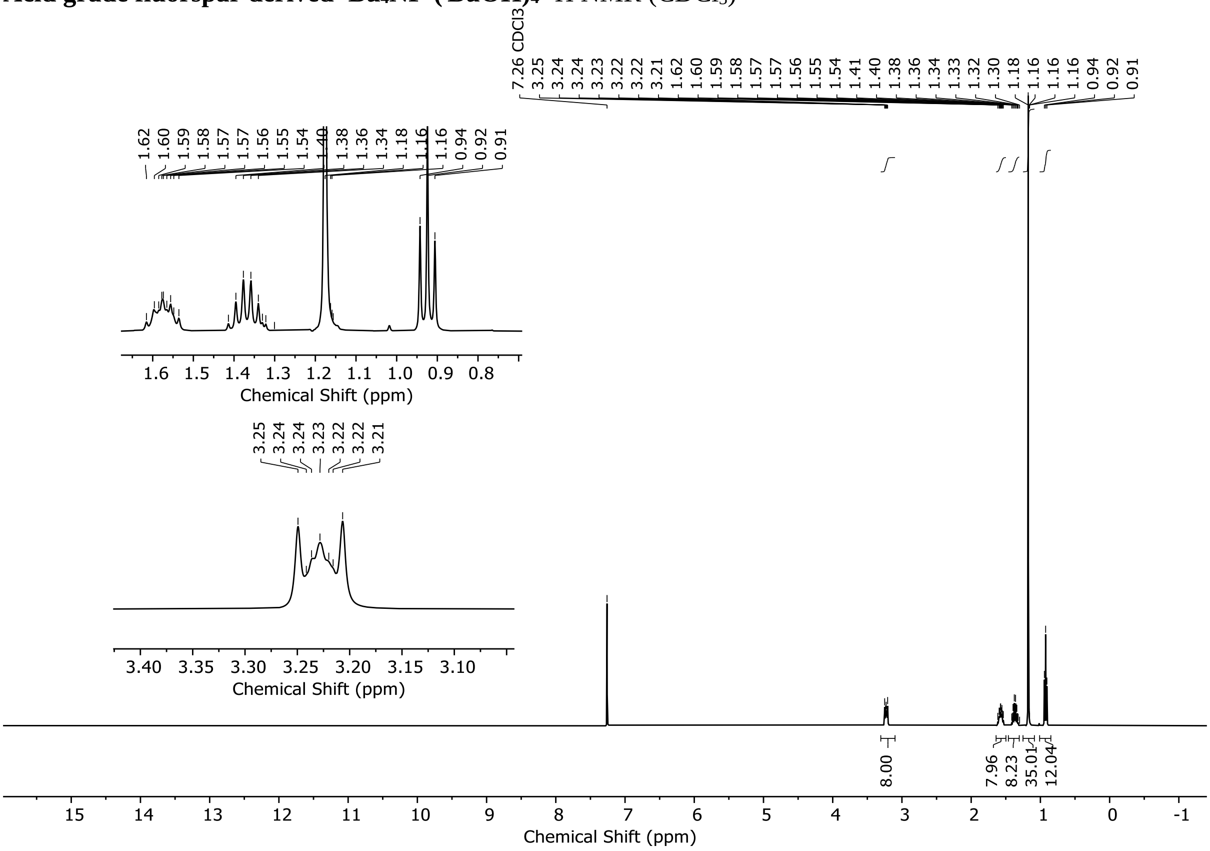

Acid grade fluorspar derived ^{n}Bu$_4$NF·(^{t}BuOH)$_4$ ^{13}C NMR (CDCl$_3$)

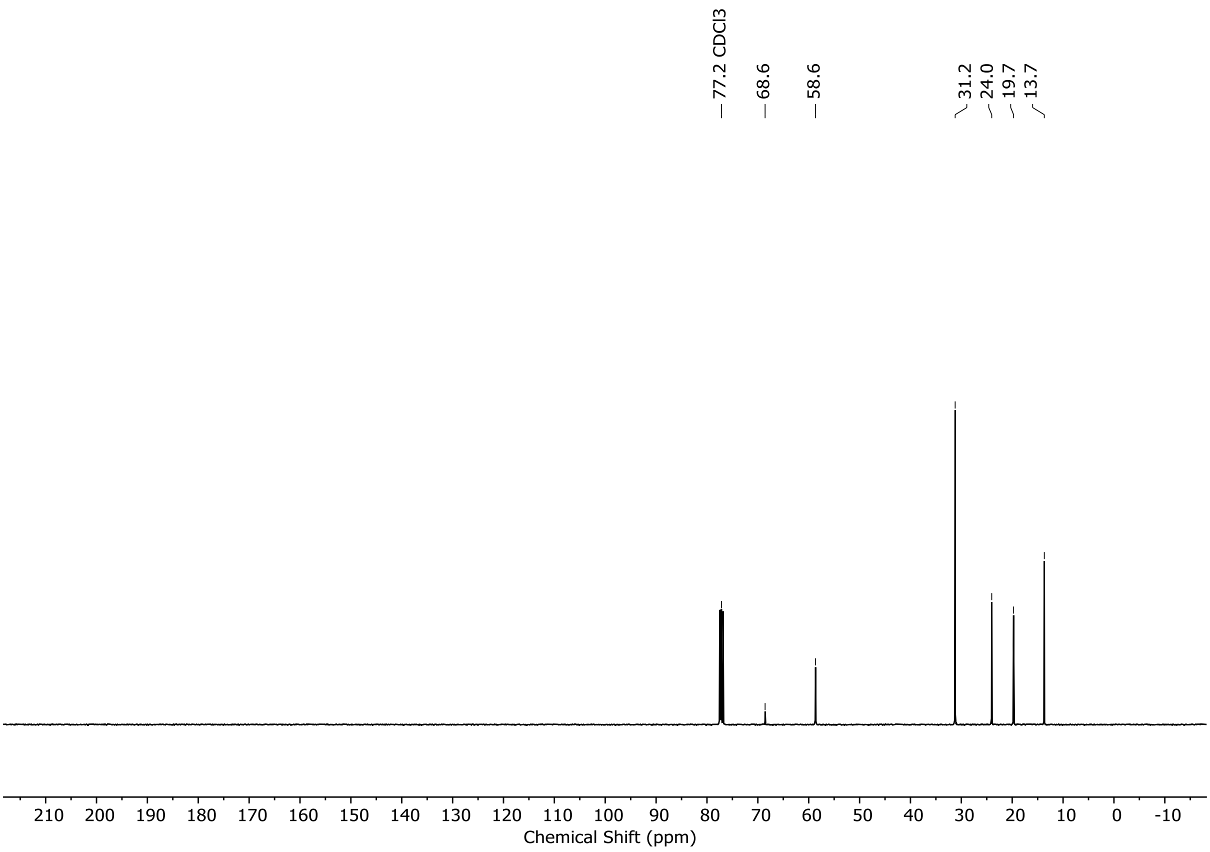

Acid grade fluorspar derived $^{n}Bu_4NF\cdot(^{t}BuOH)_4$ ^{19}F NMR ($CDCl_3$)

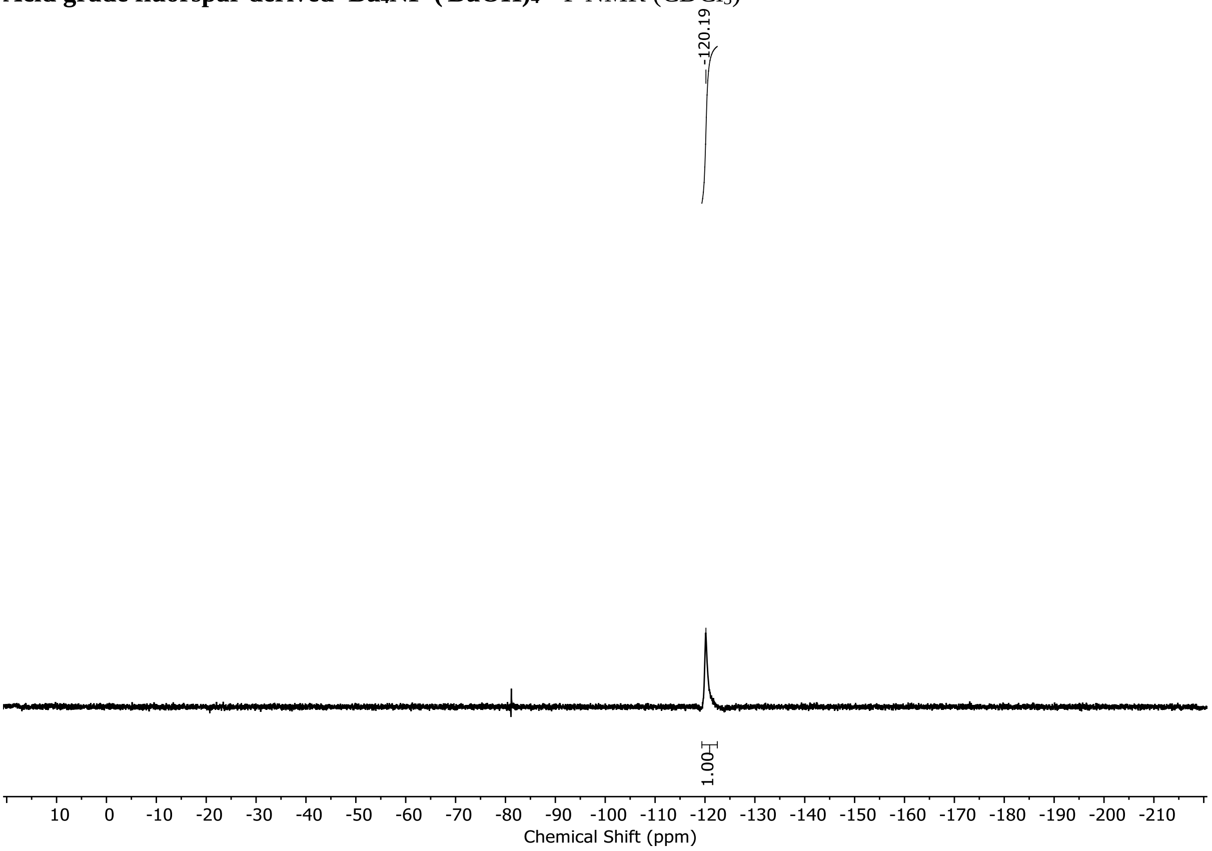

18.2. Yields determined by internal standard

Quantitative ^{19}F NMR showing conversion from **S1** to **1-Bromo-4-fluorobenzene** (**1**).

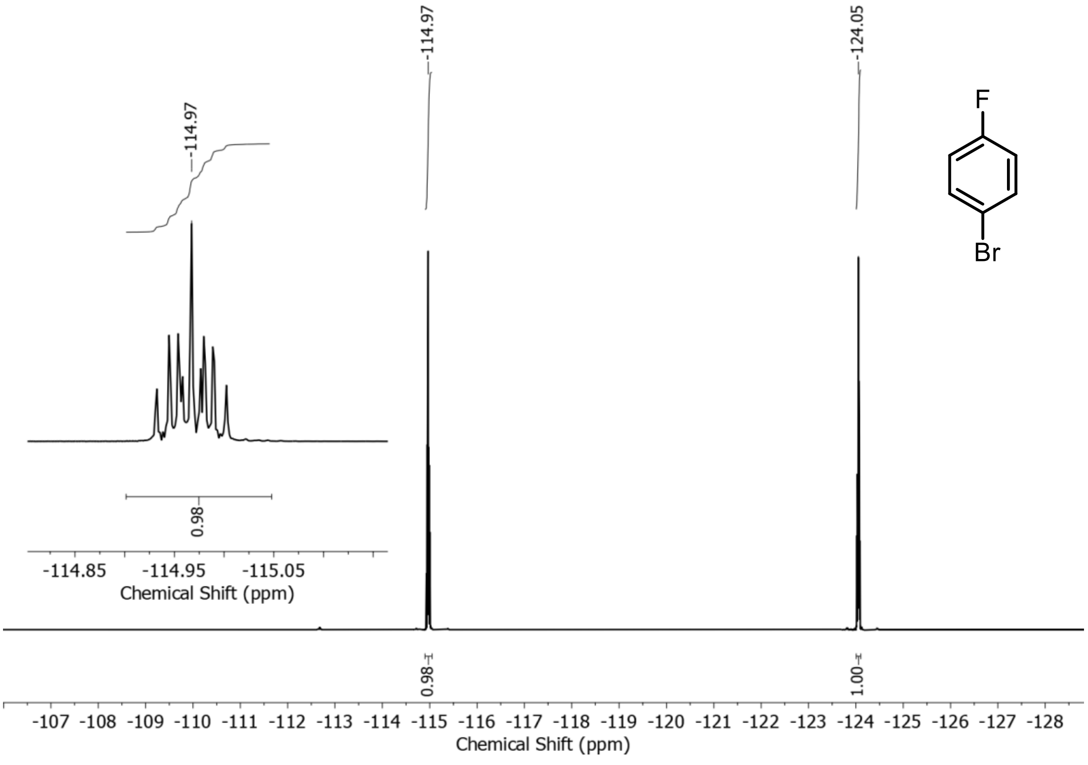

Quantitative ^{19}F NMR showing conversion of **S9** to **1-fluoro-3-methylbenzene (9)**

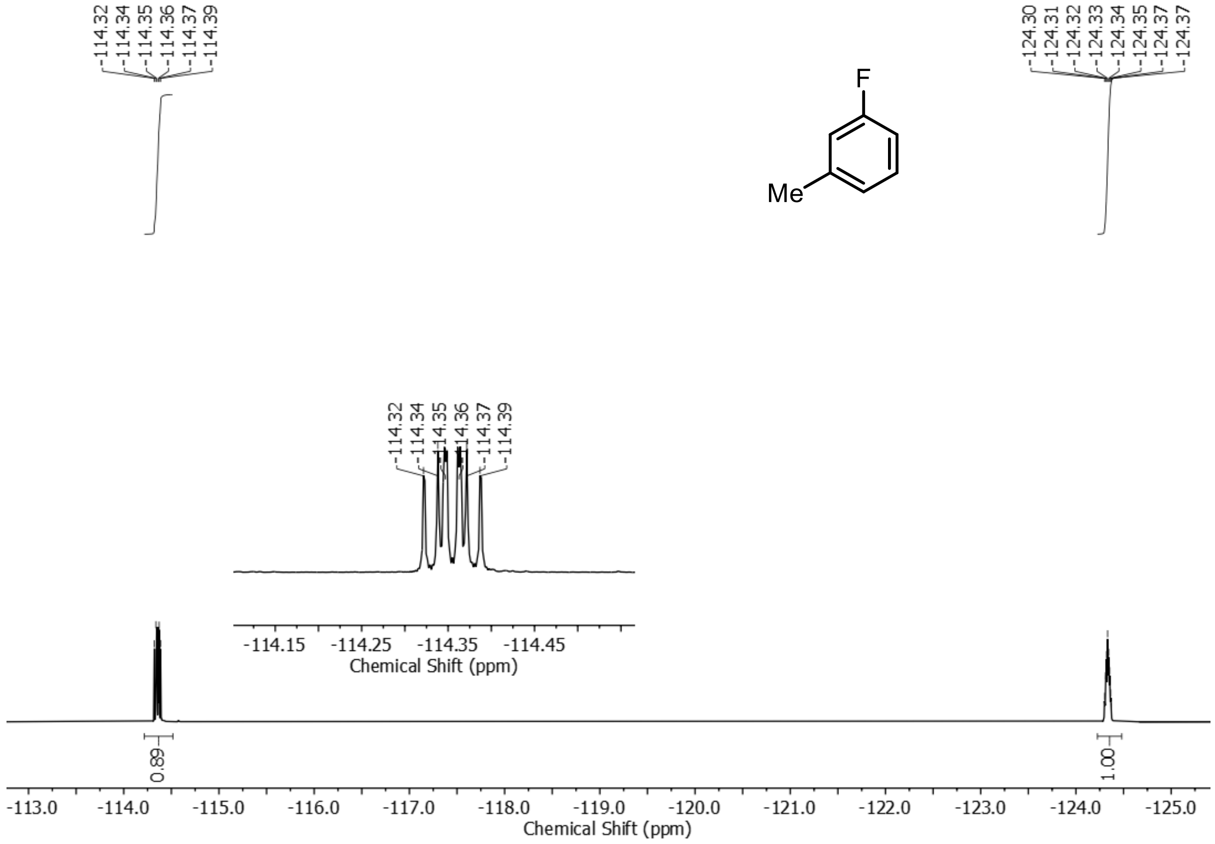

19. References

1. Britton, R. *et al.* Contemporary synthetic strategies in organofluorine chemistry. *Nat Rev Methods Primers* **1**, 47 (2021).
2. Ogawa, Y., Tokunaga, E., Kobayashi, O., Hirai, K. & Shibata, N. Current Contributions of Organofluorine Compounds to the Agrochemical Industry. *iScience* **23**, 101467 (2020).
3. Inoue, M., Sumii, Y. & Shibata, N. Contribution of Organofluorine Compounds to Pharmaceuticals. *ACS Omega* **5**, 10633–10640 (2020).
4. Harsanyi, A. & Sandford, G. Organofluorine chemistry: applications, sources and sustainability. *Green Chem.* **17**, 2081–2086 (2015).
5. Siegemund, G. *et al.* Fluorine Compounds, Organic. in *Ullmann's Encyclopedia of Industrial Chemistry* (ed. Wiley) 1–56 (Wiley, 2016). doi:10.1002/14356007.a11_349.pub2.
6. Schuler, E., Demetriou, M., Shiju, N. R. & Gruter, G.-J. M. Towards Sustainable Oxalic Acid from CO_2 and Biomass. *ChemSusChem* **14**, 3636–3664 (2021).
7. Angamuthu, R., Byers, P., Lutz, M., Spek, A. L. & Bouwman, E. Electrocatalytic CO_2 Conversion to Oxalate by a Copper Complex. *Science* **327**, 313–315 (2010).
8. Maslin, M., Van Heerde, L. & Day, S. Sulfur: A potential resource crisis that could stifle green technology and threaten food security as the world decarbonises. *Geogr. J.* **188**, 498–505 (2022).
9. Day, S., Alexander, P. & Maslin, M. Energy decarbonization threatens food security by reducing the availability of cheap sulfur. *Nat Food* **4**, 442–444 (2023).
10. Keijer, T., Bakker, V. & Slootweg, J. C. Circular chemistry to enable a circular economy. *Nature Chem* **11**, 190–195 (2019).
11. Blois, M. Electrolyte makers chase opportunities in US battery industry. *Chemical & Engineering News*.
12. Petrov, V. A. & Thrasher, J. S. From alternate routes to fluorspar (CaF_2) or anhydrous HF (aHF) to conversion of fluorspar into a nucleophilic fluorinating agent. *Journal of Fluorine Chemistry* **275**, 110274 (2024).
13. Patel, C. *et al.* Fluorochemicals from fluorspar via a phosphate-enabled mechanochemical process that bypasses HF. *Science* **381**, 302–306 (2023).
14. Lennox, A. J. J. & Lloyd-Jones, G. C. Preparation of Organotrifluoroborate Salts: Precipitation-Driven Equilibrium under Non-Etching Conditions. *Angew. Chem. Int. Ed.* **51**, 9385–9388 (2012).
15. Haynes, W. M. *CRC Handbook of Chemistry and Physics, 95th Edition*. (CRC Press, Hoboken, 2014).

16. Ihli, J. *et al.* Precipitation of Amorphous Calcium Oxalate in Aqueous Solution. *Chem. Mater.* **27**, 3999–4007 (2015).
17. Verma, A., Kore, R., Corbin, D. R. & Shiflett, M. B. Metal Recovery Using Oxalate Chemistry: A Technical Review. *Ind. Eng. Chem. Res.* **58**, 15381–15393 (2019).
18. Wamser, C. A. Hydrolysis of Fluoboric Acid in Aqueous Solution. *J. Am. Chem. Soc.* **70**, 1209–1215 (1948).
19. Ochoa, G., Pilgrim, C. D., Kerr, J., Augustine, M. P. & Casey, W. H. Aqueous geochemistry at gigapascal pressures: NMR spectroscopy of fluoroborate solutions. *Geochim. Cosmochim. Acta* **244**, 173–181 (2019).
20. Xu, Z. *et al.* Enhanced Performance of a Lithium–Sulfur Battery Using a Carbonate-Based Electrolyte. *Angew. Chem. Int. Ed.* **55**, 10372–10375 (2016).
21. Finney, W. F., Wilson, E., Callender, A., Morris, M. D. & Beck, L. W. Reexamination of Hexafluorosilicate Hydrolysis by 19F NMR and pH Measurement. *Environ. Sci. Technol.* **40**, 2572–2577 (2006).
22. W. Dean, P. A. & F. Evans, D. Spectroscopic studies of inorganic fluoro-complexes. Part III. Fluorine-19 nuclear magnetic resonance studies of silicon(IV), germanium(IV), and titanium(IV) fluoro-complexes. *J. Chem. Soc. A* **0**, 2569–2574 (1970).
23. Long, B., Wang, Z., Zhang, Q., Ke, W. & Ding, Y. Improved process to prepare high-purity anhydrous potassium fluoride from wet process phosphoric acid. *Chem. Eng. Commun.* **205**, 1342–1350 (2018).
24. Morales-Colón, M. T. *et al.* Tetramethylammonium Fluoride Alcohol Adducts for SNAr Fluorination. *Org. Lett.* **23**, 4493–4498 (2021).
25. Kim, D. W., Jeong, H.-J., Lim, S. T. & Sohn, M.-H. Tetrabutylammonium Tetra(tert-Butyl Alcohol)-Coordinated Fluoride as a Facile Fluoride Source. *Angewandte Chemie International Edition* **47**, 8404–8406 (2008).
26. Guendouzi, M. E., Faridi, J. & Khamar, L. Chemical speciation of aqueous hydrogen fluoride at various temperatures from 298.15 K to 353.15 K. *Fluid Phase Equilibria* **499**, 112244 (2019).
27. Yang, L. & Zhang, C.-P. Revisiting the Balz–Schiemann Reaction of Aryldiazonium Tetrafluoroborate in Different Solvents under Catalyst- and Additive-Free Conditions. *ACS Omega* **6**, 21595–21603 (2021).
28. Vedantham, R., Vetukuri, V. P. R., Boini, A., Khagga, M. & Bandichhor, R. Improved One-Pot Synthesis of Citalopram Diol and Its Conversion to Citalopram. *Org. Process Res. Dev.* **17**, 798–805 (2013).

29. Zarganes-Tzitzikas, T., Neochoritis, C. G. & Dömling, A. Atorvastatin (Lipitor) by MCR. *ACS Med. Chem. Lett.* **10**, 389–392 (2019).
30. Wu, Z. ‘Preparation method of norfloxacin, ciprofloxacin and enrofloxacin’, CN Patent CN104292159A (2014).
31. Caputo, F., Corbetta, S., Piccolo, O. & Vigo, D. Seeking for Selectivity and Efficiency: New Approaches in the Synthesis of Raltegravir. *Org. Process Res. Dev.* **24**, 1149–1156 (2020).
32. Xiao, X.-Y. *et al.* Fluorocyclines. 1. 7-Fluoro-9-pyrrolidinoacetamido-6-demethyl-6-deoxytetracycline: A Potent, Broad Spectrum Antibacterial Agent. *J. Med. Chem.* **55**, 597–605 (2012).
33. Balanov, A., Shenkar, N. & Niddam-Hildesheim, V. ‘Preparation of rosuvastatin’ US Patent US20070167625A1 (2006).
34. Otake, N. *et al.* ‘Method for producing optically active 2-(2-fluorobiphenyl-4-yl) propanoic acid’, US Patent US10207976B2 (2016).
35. Dai, M., Watts, V. J. & Ye, Z. ‘Adenylyl cyclase inhibitors for neuropathic and inflammatory pain’ US Patent US15044660. (2016).
36. Sasikala, C. H. V. A. *et al.* An Improved and Scalable Process for the Synthesis of Ezetimibe: An Antihypercholesterolemia Drug. *Org. Process Res. Dev.* **13**, 907–910 (2009).
37. Velcicky, J. *et al.* Modulating ADME Properties by Fluorination: MK2 Inhibitors with Improved Oral Exposure. *ACS Med. Chem. Lett.* **9**, 392–396 (2018).
38. Hirth-Dietrich, C. *et al.* ‘The use of sGC stimulators, sGC activators, alone and combinations with PDE5 inhibitors for the treatment of systemic sclerosis (SSc)’ WO Patent WO2011147810A1. (2011).
39. Z. C. ‘Preparation method of clodinafop propargyl’, CN105418494A CN Patent. (2016).
40. Mateos, J. *et al.* Nitrate reduction enables safer aryldiazonium chemistry. *Science* **384**, 446–452 (2024).
41. Souza, E. L. S. de, Chorro, T. H. D. & Correia, C. R. D. Thermal analysis of arenediazonium tetrafluoroborate salts: Stability and hazardous evaluation. *Process Safety and Environmental Protection* **177**, 69–81 (2023).
42. Firth, J. D. & Fairlamb, I. J. S. A Need for Caution in the Preparation and Application of Synthetically Versatile Aryl Diazonium Tetrafluoroborate Salts. *Org. Lett.* **22**, 7057–7059 (2020).
43. Sheng, M., Frurip, D. & Gorman, D. Reactive chemical hazards of diazonium salts. *Journal of Loss Prevention in the Process Industries* **38**, 114–118 (2015).

44. Yang, Q. *et al.* Potential Explosion Hazards Associated with the Autocatalytic Thermal Decomposition of Dimethyl Sulfoxide and Its Mixtures. *Org. Process Res. Dev.* **24**, 916–939 (2020).
45. National Research Council (US) Committee on Prudent Practices in the Laboratory. *Prudent Practices in the Laboratory: Handling and Management of Chemical Hazards: Updated Version*. (National Academies Press (US), Washington (DC), 2011).
46. Malz, F. & Jancke, H. Validation of quantitative NMR. *J. Pharm. Biomed. Anal.* **38**, 813–823 (2005).
47. MestreNova Manual https://mnova.pl/files/download/MestReNova-12-0-0_Manual.pdf (2017).
48. Burum, D. P. & Ernst, R. R. Net polarization transfer via a *J*-ordered state for signal enhancement of low-sensitivity nuclei. *J. Magn. Reson.* **39**, 163–168 (1980).
49. Morris, G. A. & Freeman, R. Enhancement of nuclear magnetic resonance signals by polarization transfer. *J. Am. Chem. Soc.* **101**, 760–762 (1979).
50. Hong, C. M., Whittaker, A. M. & Schultz, D. M. Nucleophilic Fluorination of Heteroaryl Chlorides and Aryl Triflates Enabled by Cooperative Catalysis. *J. Org. Chem.* **86**, 3999–4006 (2021).
51. Schotten, C. *et al.* Comparison of the Thermal Stabilities of Diazonium Salts and Their Corresponding Triazenes. *Org. Process Res. Dev.* **24**, 2336–2341 (2020).
52. Rodríguez-Fernández, L., Albarrán-Velo, J., Lavandera, I. & Gotor-Fernández, V. From Diazonium Salts to Optically Active 1-Arylpropan-2-ols Through a Sequential Photobiocatalytic Approach. *Adv. Synth. Catal.* **365**, 1883–1892 (2023).
53. Webb, E. W. *et al.* Room-Temperature Copper-Mediated Radiocyanation of Aryldiazonium Salts and Aryl Iodides via Aryl Radical Intermediates. *J. Am. Chem. Soc.* **145**, 6921–6926 (2023).
54. Mato, M. *et al.* Oxidative Addition of Aryl Electrophiles into a Red-Light-Active Bismuthinidene. *J. Am. Chem. Soc.* **145**, 18742–18747 (2023).
55. Sherborne, G. J. *et al.* Modular and Selective Arylation of Aryl Germanes (C−$GeEt_3$) over C−Bpin, C−SiR3 and Halogens Enabled by Light-Activated Gold Catalysis. *Angew. Chem. Int. Ed.* **59**, 15543–15548 (2020).
56. Andrejčák, S., Kisszékelyi, P., Májek, M. & Šebesta, R. Mechanochemical Radical Boronation of Aryl Diazonium Salts Promoted by Sodium Chloride. *Eur. J. Org. Chem.* **26**, e202201399 (2023).
57. Bremerich, M., Conrads, C. M., Langletz, T. & Bolm, C. Additions to N-Sulfinylamines as an Approach for the Metal-free Synthesis of Sulfonimidamides: O-Benzotriazolyl Sulfonimidates as Activated Intermediates. *Angew. Chem. Int. Ed.* **58**, 19014–19020 (2019).

58. Shiraki, T. *et al.* Multistep Wavelength Switching of Near-Infrared Photoluminescence Driven by Chemical Reactions at Local Doped Sites of Single-Walled Carbon Nanotubes. *Chem. Eur. J.* **24**, 19162–19165 (2018).

59. Wang, W. *et al.* Catalytic Electrophilic Halogenation of Arenes with Electron-Withdrawing Substituents. *J. Am. Chem. Soc.* **144**, 13415–13425 (2022).

60. Deng, M., Liu, K., Yuan, S., Luo, G. & Dian, L. Photoinduced $FeCl_3$-Catalyzed Chlorination of Aromatic Sulfonyl Chloride via Extrusion of SO2 at Room Temperature. *Org. Lett.* **25**, 4576–4580 (2023).

61. Ye, Y., Schimler, S. D., Hanley, P. S. & Sanford, M. S. $Cu(OTf)_2$-Mediated Fluorination of Aryltrifluoroborates with Potassium Fluoride. *J. Am. Chem. Soc.* **135**, 16292–16295 (2013).

62. Hebel, D. & Rozen, S. Utilizing acetyl hypofluorite for chlorination, bromination, and etherification of the pyridine system. *J. Org. Chem.* **56**, 6298–6301 (1991).

63. Furuya, T., Kaiser, H. M. & Ritter, T. Palladium-Mediated Fluorination of Arylboronic Acids. *Angew. Chem. Int. Ed.* **47**, 5993–5996 (2008).

64. Boehm, P., Roediger, S., Bismuto, A. & Morandi, B. Palladium-Catalyzed Chlorocarbonylation of Aryl (Pseudo)Halides Through In Situ Generation of Carbon Monoxide. *Angew. Chem. Int. Ed.* **59**, 17887–17896 (2020).

65. Schlosser, M. & Bobbio, C. Creating Structural Manifolds from a Common Precursor: Basicity Gradient-Driven Isomerization of Halopyridines. *Eur. J. Org. Chem.* **2002**, 4174–4180 (2002).

66. Yang, Y., Hammond, G. B. & Umemoto, T. Self-Sustaining Fluorination of Active Methylene Compounds and High-Yielding Fluorination of Highly Basic Aryl and Alkenyl Lithium Species with a Sterically Hindered N-Fluorosulfonamide Reagent. *Angew. Chem. Int. Ed.* **61**, e202211688 (2022).

67. Kutonova, K. V. *et al.* Arenediazonium Tosylates (ADTs) as Efficient Reagents for Suzuki–Miyaura Cross-Coupling in Neat Water. *Synthesis* **49**, 1680–1688 (2017).

68. Kolekar, Y. A., Saptal, V. B. & Bhanage, B. M. Carbonylative Self-Coupling of Aryl Boronic Acids Using a Confined Pd Catalyst within Melamine Dendron and Fibrous Nano-Silica: A CO Surrogate Approach. *Chem. Eur. J.* **29**, e202301381 (2023).

69. Ponticello, G. S., Engelhardt, E. L., Freedman, M. B. & Baldwin, J. J. Synthesis of 2-chloro-5-hydroxynicotinonitrile: The required intermediate in the total synthesis of a hydroxylated metabolite of (S)-2-(3-t-butylamino-2-hydroxypropoxy)-3-cyanopyridine. *J. Heterocycl. Chem.* **17**, 445–448 (1980).

70. Ung, G. & Bertrand, G. C-F Bond Activation with an Apparently Benign Ethynyl Dithiocarbamate, and Subsequent Fluoride Transfer Reactions. *Chem. Eur. J.* **18**, 12955–12957 (2012).
71. Lacour, M.-A., Zablocka, M., Duhayon, C., Majoral, J.-P. & Taillefer, M. Efficient Phosphorus Catalysts for the Halogen-Exchange (Halex) Reaction. *Adv. Synth. Catal.* **350**, 2677–2682 (2008).
72. Cohen, D. T. & Buchwald, S. L. Mild Palladium-Catalyzed Cyanation of (Hetero)aryl Halides and Triflates in Aqueous Media. *Org. Lett.* **17**, 202–205 (2015).
73. Cismesia, M. A., Ryan, S. J., Bland, D. C. & Sanford, M. S. Multiple Approaches to the In Situ Generation of Anhydrous Tetraalkylammonium Fluoride Salts for S_NAr Fluorination Reactions. *J. Org. Chem.* **82**, 5020–5026 (2017).
74. Sun, H. & DiMagno, S. G. Room-Temperature Nucleophilic Aromatic Fluorination: Experimental and Theoretical Studies. *Angew. Chem. Int. Ed.* **45**, 2720–2725 (2006).
75. Jelen, J. & Tavčar, G. Deoxyfluorination of Electron-Deficient Phenols. *Org. Lett.* **25**, 3649–3653 (2023).
76. Feng, Q. & Song, Q. Copper-Catalyzed Decarboxylative C-N Triple Bond Formation: Direct Synthesis of Benzonitriles from Phenylacetic Acids Under O_2 Atmosphere. *Adv. Synth. Catal.* **356**, 1697–1702 (2014).
77. Park, N. H., Senter, T. J. & Buchwald, S. L. Rapid Synthesis of Aryl Fluorides in Continuous Flow through the Balz-Schiemann Reaction. *Angew. Chem. Int. Ed.* **55**, 11907–11911 (2016).
